# Supplementary material for: AP-2α and AP-2β cooperatively function in the craniofacial surface ectoderm to regulate chromatin and gene expression dynamics during facial development
Source: eLife. 2022 Mar 25;11:e70511. doi: 10.7554/eLife.70511 (PMC9038197; doi:10.7554/eLife.70511)
Supplement: Figure 2—source data 1. [file elife-70511-fig2-data1.pdf]

Homer Known Motif Enrichment Results (output/)

Homer *de novo* Motif Results
Gene Ontology Enrichment Results
Known Motif Enrichment Results (txt file)
Total Target Sequences = 10362, Total Background Sequences = 33662

Rank	Motif	Name	P-value	log P-pvalue	q-value (Benjamini)	# Target Sequences with Motif	% of Targets Sequences with Motif	# Background Sequences with Motif	% of Background Sequences with Motif	Motif File	SVG
1	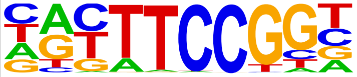	Elk4(ETS)/Hela-Elk4-ChIP-Seq(GSE31477)/Homer	1e-214	-4.950e+02	0.0000	6721.0	64.86%	16686.2	49.57%	motif file (matrix)	svg
2	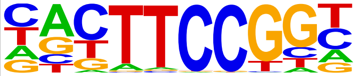	Elk1(ETS)/Hela-Elk1-ChIP-Seq(GSE31477)/Homer	1e-197	-4.557e+02	0.0000	6543.0	63.14%	16306.3	48.45%	motif file (matrix)	svg
3	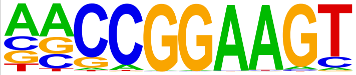	ETS(ETS)/Promoter/Homer	1e-171	-3.953e+02	0.0000	4340.0	41.88%	9751.4	28.97%	motif file (matrix)	svg
4	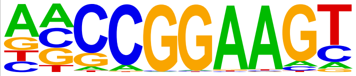	ELF1(ETS)/Jurkat-ELF1-ChIP-Seq(SRA014231)/Homer	1e-168	-3.878e+02	0.0000	6020.0	58.09%	14982.9	44.51%	motif file (matrix)	svg
5	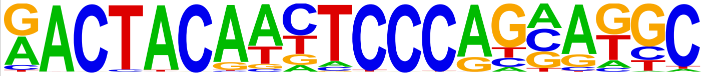	Ronin(THAP)/ES-Thap11-ChIP-Seq(GSE51522)/Homer	1e-159	-3.683e+02	0.0000	956.0	9.23%	1153.4	3.43%	motif file (matrix)	svg
6	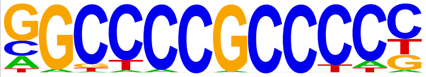	Sp1(Zf)/Promoter/Homer	1e-147	-3.387e+02	0.0000	6022.0	58.11%	15289.1	45.42%	motif file (matrix)	svg
7	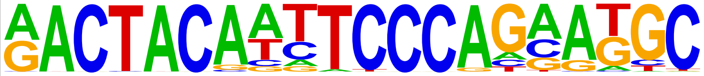	GFY-Staf(?,Zf)/Promoter/Homer	1e-114	-2.647e+02	0.0000	1097.0	10.59%	1684.8	5.01%	motif file (matrix)	svg
8	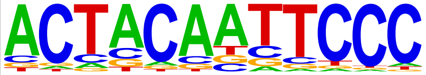	GFY(?)/Promoter/Homer	1e-101	-2.327e+02	0.0000	1135.0	10.95%	1865.4	5.54%	motif file (matrix)	svg
9	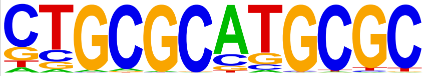	NRF1(NRF)/MCF7-NRF1-ChIP-Seq(Unpublished)/Homer	1e-86	-2.000e+02	0.0000	3208.0	30.96%	7577.4	22.51%	motif file (matrix)	svg
10	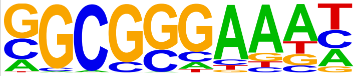	E2F4(E2F)/K562-E2F4-ChIP-Seq(GSE31477)/Homer	1e-77	-1.781e+02	0.0000	6058.0	58.46%	16594.6	49.30%	motif file (matrix)	svg
11	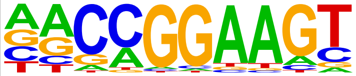	GABPA(ETS)/Jurkat-GABPa-ChIP-Seq(GSE17954)/Homer	1e-67	-1.565e+02	0.0000	6645.0	64.12%	18736.6	55.67%	motif file (matrix)	svg
12	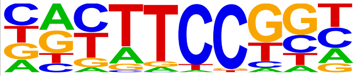	Fli1(ETS)/CD8-FLI-ChIP-Seq(GSE20898)/Homer	1e-65	-1.519e+02	0.0000	7708.0	74.38%	22409.6	66.58%	motif file (matrix)	svg
13	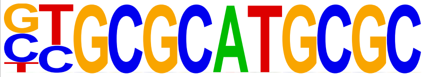	NRF(NRF)/Promoter/Homer	1e-65	-1.510e+02	0.0000	3187.0	30.75%	7870.5	23.38%	motif file (matrix)	svg
14	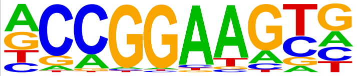	ETV4(ETS)/HepG2-ETV4-ChIP-Seq(ENCODE)/Homer	1e-51	-1.184e+02	0.0000	7885.0	76.09%	23352.5	69.38%	motif file (matrix)	svg

15	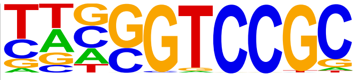	HINFP(Zf)/K562-HINFP.eGFP-ChIP-Seq(Encode)/Homer	1e-38	-8.879e+01	0.0000	5102.0	49.23%	14423.0	42.85%	motif file (matrix)	svg
16	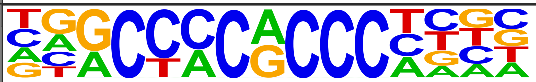	KLF3(Zf)/MEF-Klf3-ChIP-Seq(GSE44748)/Homer	1e-33	-7.780e+01	0.0000	6428.0	62.03%	18887.1	56.11%	motif file (matrix)	svg
17	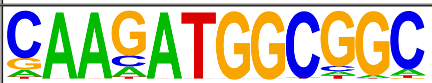	YY1(Zf)/Promoter/Homer	1e-31	-7.329e+01	0.0000	1301.0	12.55%	3044.4	9.04%	motif file (matrix)	svg
18	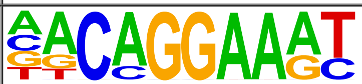	EWS:FLI1-fusion(ETS)/SK_N_MC-EWS:FLI1-ChIP-Seq(SRA014231)/Homer	1e-31	-7.144e+01	0.0000	4287.0	41.37%	12054.1	35.81%	motif file (matrix)	svg
19	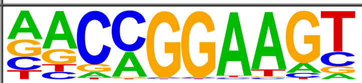	ETV1(ETS)/GIST48-ETV1-ChIP-Seq(GSE22441)/Homer	1e-27	-6.350e+01	0.0000	7812.0	75.38%	23746.5	70.55%	motif file (matrix)	svg
20	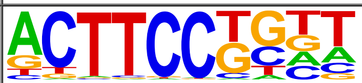	Elf4(ETS)/BMDM-Elf4-ChIP-Seq(GSE88699)/Homer	1e-24	-5.725e+01	0.0000	6481.0	62.54%	19360.2	57.52%	motif file (matrix)	svg
21	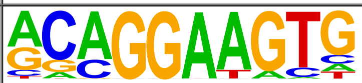	ETS1(ETS)/Jurkat-ETS1-ChIP-Seq(GSE17954)/Homer	1e-24	-5.632e+01	0.0000	6546.0	63.17%	19590.5	58.20%	motif file (matrix)	svg
22	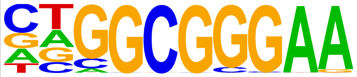	E2F1(E2F)/Hela-E2F1-ChIP-Seq(GSE22478)/Homer	1e-23	-5.443e+01	0.0000	3873.0	37.37%	10991.9	32.66%	motif file (matrix)	svg
23	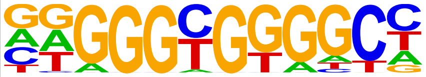	KLF1(Zf)/HUDEP2-KLF1-CutnRun(GSE136251)/Homer	1e-23	-5.443e+01	0.0000	8262.0	79.73%	25424.4	75.54%	motif file (matrix)	svg
24	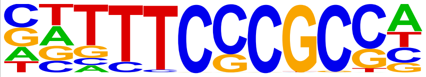	E2F7(E2F)/Hela-E2F7-ChIP-Seq(GSE32673)/Homer	1e-23	-5.388e+01	0.0000	2203.0	21.26%	5858.1	17.40%	motif file (matrix)	svg
25	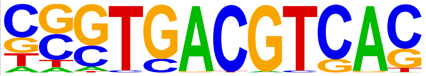	CRE(bZIP)/Promoter/Homer	1e-22	-5.223e+01	0.0000	2203.0	21.26%	5878.3	17.46%	motif file (matrix)	svg
26	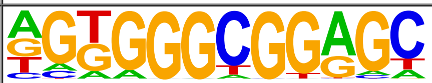	Sp5(Zf)/mES-Sp5.Flag-ChIP-Seq(GSE72989)/Homer	1e-20	-4.727e+01	0.0000	8769.0	84.62%	27294.7	81.09%	motif file (matrix)	svg
27	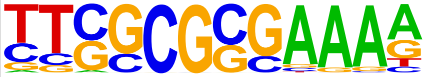	E2F(E2F)/Hela-CellCycle-Expression/Homer	1e-20	-4.643e+01	0.0000	1044.0	10.07%	2539.8	7.55%	motif file (matrix)	svg
28	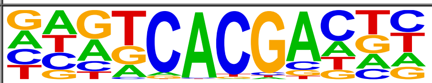	Npas4(bHLH)/Neuron-Npas4-ChIP-Seq(GSE127793)/Homer	1e-18	-4.341e+01	0.0000	5738.0	55.37%	17153.7	50.96%	motif file (matrix)	svg
29	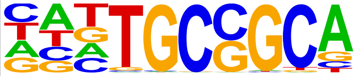	Zfp57(Zf)/H1-ZFP57.HA-ChIP-Seq(GSE115387)/Homer	1e-18	-4.338e+01	0.0000	4888.0	47.17%	14401.1	42.79%	motif file (matrix)	svg
30	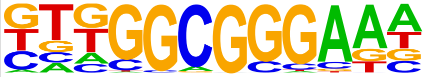	E2F3(E2F)/MEF-E2F3-ChIP-Seq(GSE71376)/Homer	1e-17	-4.030e+01	0.0000	6683.0	64.49%	20318.3	60.37%	motif file (matrix)	svg
31	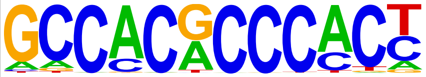	Klf9(Zf)/GBM-Klf9-ChIP-Seq(GSE62211)/Homer	1e-17	-3.976e+01	0.0000	5455.0	52.64%	16301.0	48.43%	motif file (matrix)	svg
32	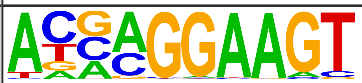	ELF5(ETS)/T47D-ELF5-ChIP-Seq(GSE30407)/Homer	1e-16	-3.783e+01	0.0000	4675.0	45.11%	13820.0	41.06%	motif file (matrix)	svg
33		Klf4(Zf)/mES-Klf4-ChIP-	1e-	-3.627e+01	0.0000	4265.0	41.16%	12539.0	37.25%	motif	svg

	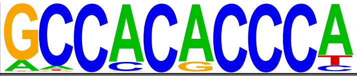	Seq(GSE11431)/Homer	15							file (matrix)	
34	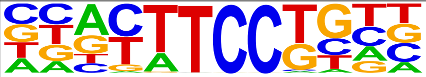	Etv2(ETS)/ES-ER71-ChIP-Seq(GSE59402)/Homer	1e-15	-3.597e+01	0.0000	5637.0	54.40%	16966.4	50.41%	motif file (matrix)	svg
35	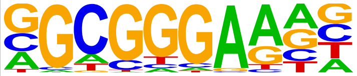	E2F6(E2F)/Hela-E2F6-ChIP-Seq(GSE31477)/Homer	1e-14	-3.318e+01	0.0000	6077.0	58.64%	18461.4	54.85%	motif file (matrix)	svg
36	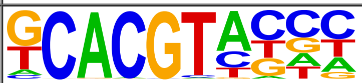	HIF2a(bHLH)/785_O-HIF2a-ChIP-Seq(GSE34871)/Homer	1e-13	-3.162e+01	0.0000	3158.0	30.47%	9129.7	27.12%	motif file (matrix)	svg
37	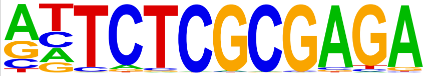	GFX(?)/Promoter/Homer	1e-13	-3.116e+01	0.0000	355.0	3.43%	756.9	2.25%	motif file (matrix)	svg
38	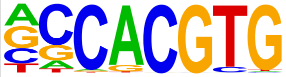	c-Myc(bHLH)/LNCAP-cMyc-ChIP-Seq(Unpublished)/Homer	1e-12	-2.967e+01	0.0000	4363.0	42.10%	12986.4	38.58%	motif file (matrix)	svg
39	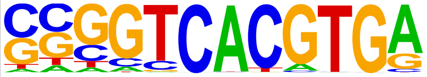	E-box(bHLH)/Promoter/Homer	1e-12	-2.905e+01	0.0000	984.0	9.50%	2540.8	7.55%	motif file (matrix)	svg
40	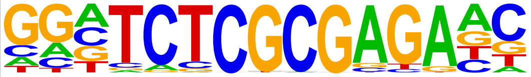	ZBTB33(Zf)/GM12878-ZBTB33-ChIP-Seq(GSE32465)/Homer	1e-12	-2.883e+01	0.0000	954.0	9.21%	2455.6	7.30%	motif file (matrix)	svg
41	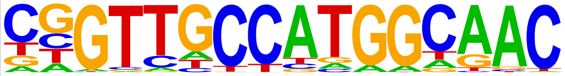	RFX(HTH)/K562-RFX3-ChIP-Seq(SRA012198)/Homer	1e-12	-2.850e+01	0.0000	951.0	9.18%	2451.0	7.28%	motif file (matrix)	svg
42	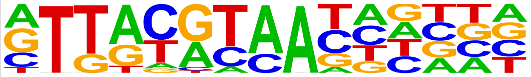	NFIL3(bZIP)/HepG2-NFIL3-ChIP-Seq(Encode)/Homer	1e-11	-2.563e+01	0.0000	2499.0	24.11%	7187.7	21.35%	motif file (matrix)	svg
43	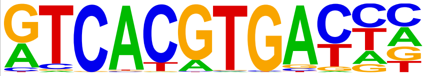	TFE3(bHLH)/MEF-TFE3-ChIP-Seq(GSE75757)/Homer	1e-11	-2.562e+01	0.0000	765.0	7.38%	1942.9	5.77%	motif file (matrix)	svg
44	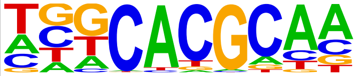	Arnt:Ahr(bHLH)/MCF7-Arnt-ChIP-Seq(Lo_et_al.)/Homer	1e-10	-2.437e+01	0.0000	5257.0	50.73%	15988.9	47.50%	motif file (matrix)	svg
45	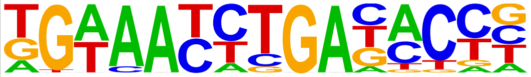	Six4(Homeobox)/MCF7-SIX4-ChIP-Seq(Encode)/Homer	1e-9	-2.298e+01	0.0000	373.0	3.60%	859.2	2.55%	motif file (matrix)	svg
46	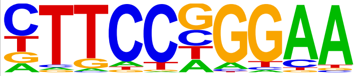	Stat3(Stat)/mES-Stat3-ChIP-Seq(GSE11431)/Homer	1e-9	-2.215e+01	0.0000	3625.0	34.98%	10804.4	32.10%	motif file (matrix)	svg
47	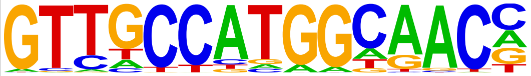	Rfx2(HTH)/LoVo-RFX2-ChIP-Seq(GSE49402)/Homer	1e-9	-2.155e+01	0.0000	973.0	9.39%	2601.6	7.73%	motif file (matrix)	svg
48	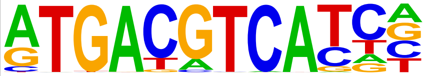	JunD(bZIP)/K562-JunD-ChIP-Seq/Homer	1e-9	-2.132e+01	0.0000	739.0	7.13%	1915.2	5.69%	motif file (matrix)	svg
49	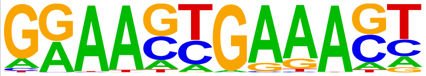	IRF8(IRF)/BMDM-IRF8-ChIP-Seq(GSE77884)/Homer	1e-9	-2.131e+01	0.0000	1991.0	19.21%	5697.7	16.93%	motif file (matrix)	svg
50	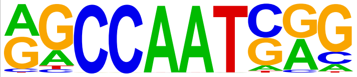	NFY(CCAAT)/Promoter/Homer	1e-8	-2.065e+01	0.0000	4913.0	47.41%	14970.7	44.48%	motif file (matrix)	svg
51		CEBP(bZIP)/ThioMac-CEBPb-ChIP-	1e-8	-2.048e+01	0.0000	2577.0	24.87%	7537.8	22.39%	motif	svg

	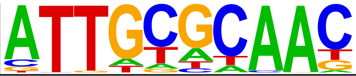	Seq(GSE21512)/Homer								file (matrix)	
52	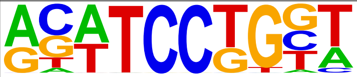	SPDEF(ETS)/VCaP-SPDEF-ChIP-Seq(SRA014231)/Homer	1e-8	-1.987e+01	0.0000	6010.0	57.99%	18557.8	55.13%	motif file (matrix)	svg
53	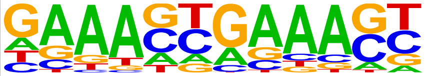	IRF2(IRF)/Erythroblas-IRF2-ChIP-Seq(GSE36985)/Homer	1e-8	-1.978e+01	0.0000	707.0	6.82%	1840.2	5.47%	motif file (matrix)	svg
54	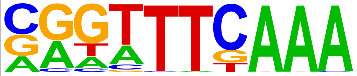	CHR(?)/Hela-CellCycle-Expression/Homer	1e-7	-1.725e+01	0.0000	2624.0	25.32%	7760.6	23.06%	motif file (matrix)	svg
55	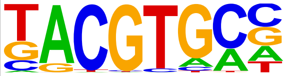	HIF-1a(bHLH)/MCF7-HIF1a-ChIP-Seq(GSE28352)/Homer	1e-7	-1.698e+01	0.0000	2296.0	22.16%	6738.3	20.02%	motif file (matrix)	svg
56	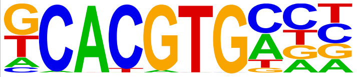	bHLHE40(bHLH)/HepG2-BHLHE40-ChIP-Seq(GSE31477)/Homer	1e-6	-1.497e+01	0.0000	2514.0	24.26%	7472.8	22.20%	motif file (matrix)	svg
57	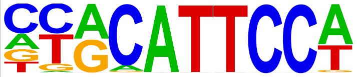	TEAD1(TEAD)/HepG2-TEAD1-ChIP-Seq(Encode)/Homer	1e-6	-1.431e+01	0.0000	4466.0	43.10%	13713.0	40.74%	motif file (matrix)	svg
58	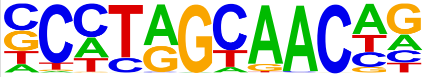	Rfx5(HTH)/GM12878-Rfx5-ChIP-Seq(GSE31477)/Homer	1e-5	-1.251e+01	0.0000	2242.0	21.63%	6683.8	19.86%	motif file (matrix)	svg
59	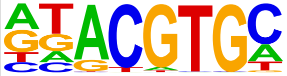	HIF-1b(HLH)/T47D-HIF1b-ChIP-Seq(GSE59937)/Homer	1e-5	-1.222e+01	0.0000	7030.0	67.84%	22141.4	65.78%	motif file (matrix)	svg
60	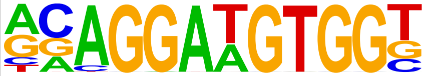	ETS:RUNX(ETS,Runt)/Jurkat-RUNX1-ChIP-Seq(GSE17954)/Homer	1e-5	-1.213e+01	0.0000	851.0	8.21%	2381.0	7.07%	motif file (matrix)	svg
61	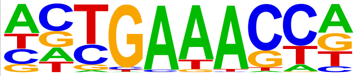	IRF4(IRF)/GM12878-IRF4-ChIP-Seq(GSE32465)/Homer	1e-5	-1.158e+01	0.0001	2179.0	21.03%	6511.6	19.35%	motif file (matrix)	svg
62	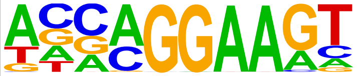	EHF(ETS)/LoVo-EHF-ChIP-Seq(GSE49402)/Homer	1e-4	-1.107e+01	0.0001	6613.0	63.81%	20810.7	61.83%	motif file (matrix)	svg
63	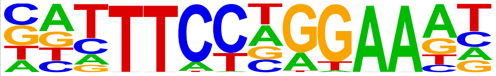	STAT1(Stat)/HelaS3-STAT1-ChIP-Seq(GSE12782)/Homer	1e-4	-1.050e+01	0.0002	1585.0	15.29%	4679.7	13.90%	motif file (matrix)	svg
64	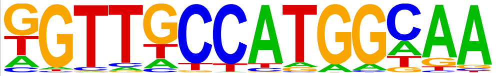	X-box(HTH)/NPC-H3K4me1-ChIP-Seq(GSE16256)/Homer	1e-4	-9.323e+00	0.0006	793.0	7.65%	2258.6	6.71%	motif file (matrix)	svg
65	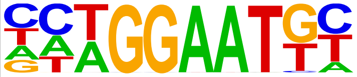	TEAD(TEA)/Fibroblast-PU.1-ChIP-Seq(Unpublished)/Homer	1e-3	-9.059e+00	0.0008	2874.0	27.73%	8796.0	26.13%	motif file (matrix)	svg
66	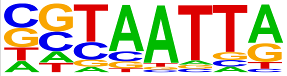	DLX5(Homeobox)/BasalGanglia-Dlx5-ChIP-seq(GSE124936)/Homer	1e-3	-9.017e+00	0.0008	2708.0	26.13%	8268.4	24.57%	motif file (matrix)	svg
67	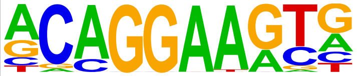	ERG(ETS)/VCaP-ERG-ChIP-Seq(GSE14097)/Homer	1e-3	-8.260e+00	0.0017	7621.0	73.54%	24238.9	72.01%	motif file (matrix)	svg
68	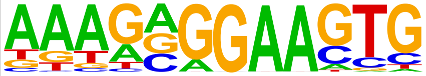	SpiB(ETS)/OCILY3-SPIB-ChIP-Seq(GSE56857)/Homer	1e-3	-8.211e+00	0.0017	1540.0	14.86%	4604.0	13.68%	motif file (matrix)	svg
69		STAT6(Stat)/Macrophage-Stat6-	1e-3	-8.103e+00	0.0019	2522.0	24.34%	7711.0	22.91%	motif	svg

	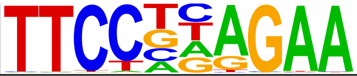	ChIP-Seq(GSE38377)/Homer								file (matrix)	
70	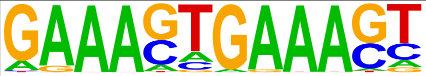	IRF1(IRF)/PBMC-IRF1-ChIP-Seq(GSE43036)/Homer	1e-3	-7.168e+00	0.0048	777.0	7.50%	2256.1	6.70%	motif file (matrix)	svg
71	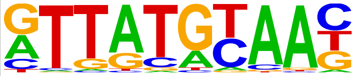	HLF(bZIP)/HSC-HLF.Flag-ChIP-Seq(GSE69817)/Homer	1e-3	-7.127e+00	0.0050	2807.0	27.09%	8657.4	25.72%	motif file (matrix)	svg
72	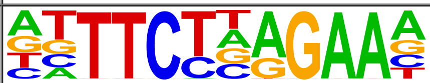	STAT5(Stat)/mCD4+-Stat5-ChIP-Seq(GSE12346)/Homer	1e-3	-6.923e+00	0.0060	1799.0	17.36%	5461.7	16.23%	motif file (matrix)	svg
73	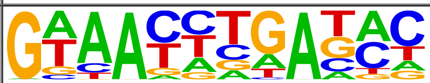	Six2(Homeobox)/NephronProgenitor-Six2-ChIP-Seq(GSE39837)/Homer	1e-2	-6.809e+00	0.0066	4492.0	43.35%	14088.7	41.86%	motif file (matrix)	svg
74	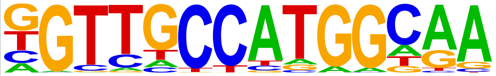	Rfx1(HTH)/NPC-H3K4me1-ChIP-Seq(GSE16256)/Homer	1e-2	-6.597e+00	0.0081	1553.0	14.99%	4696.1	13.95%	motif file (matrix)	svg
75	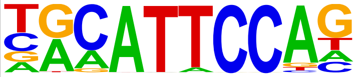	TEAD3(TEA)/HepG2-TEAD3-ChIP-Seq(Encode)/Homer	1e-2	-6.244e+00	0.0113	4912.0	47.40%	15476.6	45.98%	motif file (matrix)	svg
76	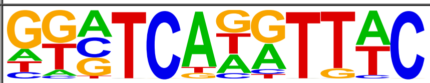	Six1(Homeobox)/Myoblast-Six1-ChIP-Chip(GSE20150)/Homer	1e-2	-5.768e+00	0.0180	1240.0	11.97%	3739.1	11.11%	motif file (matrix)	svg
77	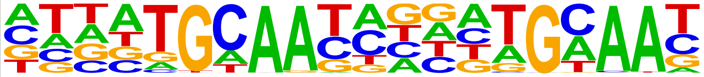	CEBP:CEBP(bZIP)/MEF-Chop-ChIP-Seq(GSE35681)/Homer	1e-2	-5.665e+00	0.0197	505.0	4.87%	1454.3	4.32%	motif file (matrix)	svg
78	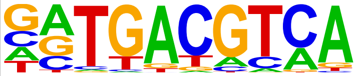	Atf1(bZIP)/K562-ATF1-ChIP-Seq(GSE31477)/Homer	1e-2	-5.441e+00	0.0243	3290.0	31.75%	10283.6	30.55%	motif file (matrix)	svg
79	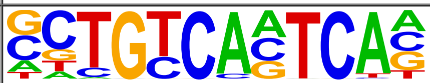	Pknox1(Homeobox)/ES-Prep1-ChIP-Seq(GSE63282)/Homer	1e-2	-5.406e+00	0.0249	1704.0	16.44%	5218.2	15.50%	motif file (matrix)	svg
80	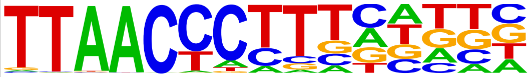	ZNF652/HepG2-ZNF652.Flag-ChIP-Seq(Encode)/Homer	1e-2	-5.386e+00	0.0251	1305.0	12.59%	3957.8	11.76%	motif file (matrix)	svg
81	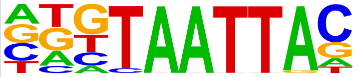	Dlx3(Homeobox)/Kerainocytes-Dlx3-ChIP-Seq(GSE89884)/Homer	1e-2	-5.210e+00	0.0295	2205.0	21.28%	6820.3	20.26%	motif file (matrix)	svg
82	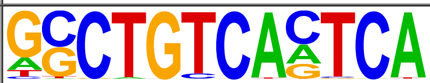	PBX1(Homeobox)/MCF7-PBX1-ChIP-Seq(GSE28007)/Homer	1e-2	-5.158e+00	0.0307	655.0	6.32%	1929.7	5.73%	motif file (matrix)	svg
83	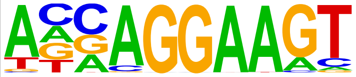	ELF3(ETS)/PDAC-ELF3-ChIP-Seq(GSE64557)/Homer	1e-2	-5.116e+00	0.0317	4029.0	38.88%	12681.8	37.68%	motif file (matrix)	svg
84	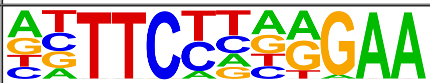	STAT6(Stat)/CD4-Stat6-ChIP-Seq(GSE22104)/Homer	1e-2	-5.049e+00	0.0335	2363.0	22.80%	7332.7	21.79%	motif file (matrix)	svg
85	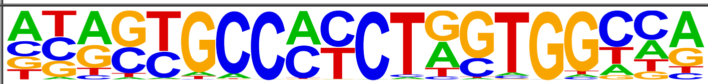	CTCF(Zf)/CD4+-CTCF-ChIP-Seq(Barski_et_al.)/Homer	1e-2	-4.830e+00	0.0412	1294.0	12.49%	3943.7	11.72%	motif file (matrix)	svg
