# Supplementary material for: AP-2α and AP-2β cooperatively function in the craniofacial surface ectoderm to regulate chromatin and gene expression dynamics during facial development
Source: eLife. 2022 Mar 25;11:e70511. doi: 10.7554/eLife.70511 (PMC9038197; doi:10.7554/eLife.70511)
Supplement: Figure 2—source data 2. [file elife-70511-fig2-data2.pdf]

Homer Known Motif Enrichment Results (cluster2/)

Homer *de novo* Motif Results
Gene Ontology Enrichment Results
Known Motif Enrichment Results (txt file)
Total Target Sequences = 54946, Total Background Sequences = 54329

Rank	Motif	Name	P-value	log P-pvalue	q-value (Benjamini)	# Target Sequences with Motif	% of Targets Sequences with Motif	# Background Sequences with Motif	% of Background Sequences with Motif	Motif File	SVG
1	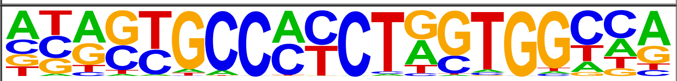	CTCF(Zf)/CD4+-CTCF-ChIP-Seq(Barski_et_al.)/Homer	1e-2528	-5.822e+03	0.0000	9588.0	17.45%	2640.4	4.86%	motif file (matrix)	svg
2	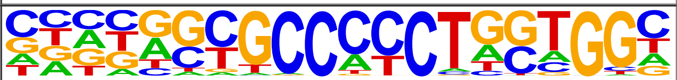	BORIS(Zf)/K562-CTCFL-ChIP-Seq(GSE32465)/Homer	1e-1970	-4.538e+03	0.0000	10985.0	20.00%	3997.6	7.36%	motif file (matrix)	svg
3	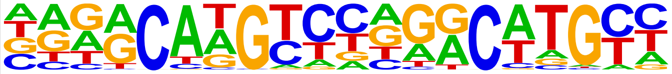	p73(p53)/Trachea-p73-ChIP-Seq(PRJNA310161)/Homer	1e-1505	-3.466e+03	0.0000	4165.0	7.58%	827.8	1.52%	motif file (matrix)	svg
4	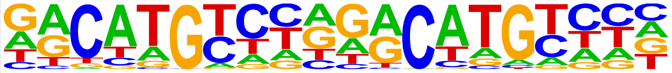	p53(p53)/Saos-p53-ChIP-Seq(GSE15780)/Homer	1e-1413	-3.254e+03	0.0000	5854.0	10.66%	1681.3	3.10%	motif file (matrix)	svg
5	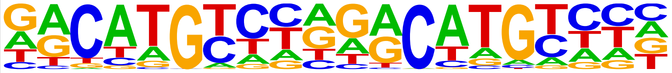	p53(p53)/Saos-p53-ChIP-Seq/Homer	1e-1413	-3.254e+03	0.0000	5854.0	10.66%	1681.3	3.10%	motif file (matrix)	svg
6	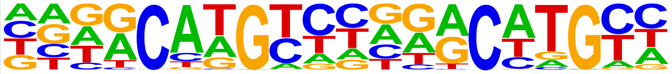	p63(p53)/Keratinocyte-p63-ChIP-Seq(GSE17611)/Homer	1e-1313	-3.025e+03	0.0000	12907.0	23.50%	6312.4	11.63%	motif file (matrix)	svg
7	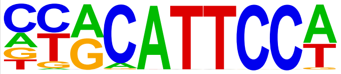	TEAD1(TEAD)/HepG2-TEAD1-ChIP-Seq(Encode)/Homer	1e-872	-2.010e+03	0.0000	19969.0	36.35%	13156.4	24.23%	motif file (matrix)	svg
8	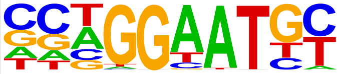	TEAD4(TEA)/Tropoblast-Tead4-ChIP-Seq(GSE37350)/Homer	1e-786	-1.811e+03	0.0000	18539.0	33.75%	12220.8	22.51%	motif file (matrix)	svg
9	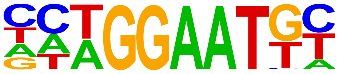	TEAD(TEA)/Fibroblast-PU.1-ChIP-Seq(Unpublished)/Homer	1e-740	-1.705e+03	0.0000	14546.0	26.48%	9013.1	16.60%	motif file (matrix)	svg
10	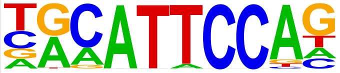	TEAD3(TEA)/HepG2-TEAD3-ChIP-Seq(Encode)/Homer	1e-710	-1.636e+03	0.0000	21819.0	39.72%	15401.6	28.36%	motif file (matrix)	svg
11	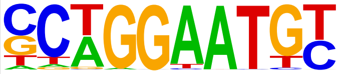	TEAD2(TEA)/Py2T-Tead2-ChIP-Seq(GSE55709)/Homer	1e-641	-1.478e+03	0.0000	12540.0	22.83%	7693.0	14.17%	motif file (matrix)	svg
12	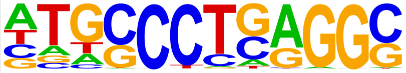	AP-2alpha(AP2)/Hela-AP2alpha-ChIP-Seq(GSE31477)/Homer	1e-620	-1.429e+03	0.0000	19624.0	35.72%	13809.3	25.43%	motif file (matrix)	svg
13	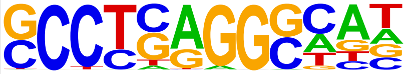	AP-2gamma(AP2)/MCF7-TFAP2C-ChIP-Seq(GSE21234)/Homer	1e-503	-1.160e+03	0.0000	23236.0	42.30%	17637.9	32.48%	motif file (matrix)	svg
14	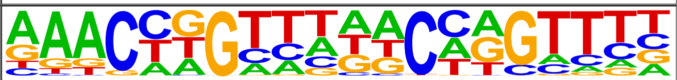	GRHL2(CP2)/HBE-GRHL2-ChIP-Seq(GSE46194)/Homer	1e-353	-8.132e+02	0.0000	9011.0	16.40%	5827.9	10.73%	motif file (matrix)	svg
15	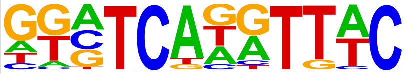	Six1(Homeobox)/Myoblast-Six1-ChIP-Chip(GSE20150)/Homer	1e-219	-5.057e+02	0.0000	5183.0	9.43%	3246.0	5.98%	motif file (matrix)	svg

16	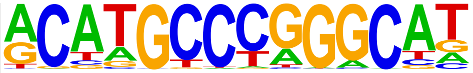	p53(p53)/mES-cMyc-ChIP-Seq(GSE11431)/Homer	1e-196	-4.518e+02	0.0000	980.0	1.78%	313.2	0.58%	motif file (matrix)	svg
17	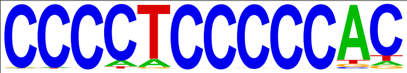	Zfp281(Zf)/ES-Zfp281-ChIP-Seq(GSE81042)/Homer	1e-182	-4.204e+02	0.0000	7366.0	13.41%	5207.1	9.59%	motif file (matrix)	svg
18	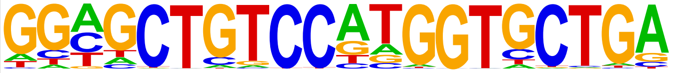	REST-NRSF(Zf)/Jurkat-NRSF-ChIP-Seq/Homer	1e-181	-4.187e+02	0.0000	608.0	1.11%	142.7	0.26%	motif file (matrix)	svg
19	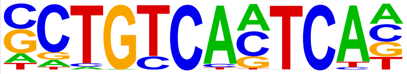	Pbx3(Homeobox)/GM12878-PBX3-ChIP-Seq(GSE32465)/Homer	1e-178	-4.103e+02	0.0000	6037.0	10.99%	4112.1	7.57%	motif file (matrix)	svg
20	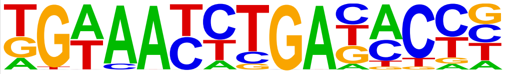	Six4(Homeobox)/MCF7-SIX4-ChIP-Seq(Encode)/Homer	1e-174	-4.019e+02	0.0000	1535.0	2.79%	675.6	1.24%	motif file (matrix)	svg
21	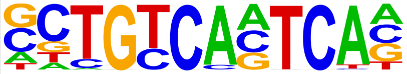	Pknox1(Homeobox)/ES-Prep1-ChIP-Seq(GSE63282)/Homer	1e-149	-3.438e+02	0.0000	5617.0	10.22%	3901.2	7.18%	motif file (matrix)	svg
22	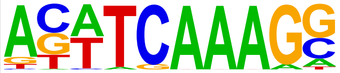	Tcf3(HMG)/mES-Tcf3-ChIP-Seq(GSE11724)/Homer	1e-148	-3.413e+02	0.0000	5375.0	9.78%	3705.0	6.82%	motif file (matrix)	svg
23	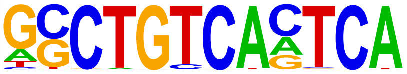	PBX1(Homeobox)/MCF7-PBX1-ChIP-Seq(GSE28007)/Homer	1e-148	-3.412e+02	0.0000	2522.0	4.59%	1430.9	2.64%	motif file (matrix)	svg
24	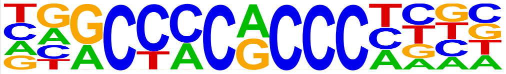	KLF3(Zf)/MEF-Klf3-ChIP-Seq(GSE44748)/Homer	1e-147	-3.403e+02	0.0000	11854.0	21.58%	9377.3	17.27%	motif file (matrix)	svg
25	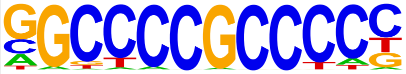	Sp1(Zf)/Promoter/Homer	1e-144	-3.334e+02	0.0000	6134.0	11.17%	4360.1	8.03%	motif file (matrix)	svg
26	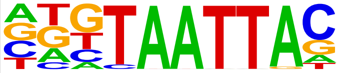	Dlx3(Homeobox)/Kerainocytes-Dlx3-ChIP-Seq(GSE89884)/Homer	1e-130	-3.000e+02	0.0000	9587.0	17.45%	7467.7	13.75%	motif file (matrix)	svg
27	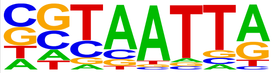	DLX5(Homeobox)/BasalGanglia-Dlx5-ChIP-seq(GSE124936)/Homer	1e-119	-2.742e+02	0.0000	11241.0	20.46%	9047.5	16.66%	motif file (matrix)	svg
28	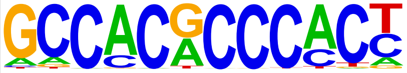	Klf9(Zf)/GBM-Klf9-ChIP-Seq(GSE62211)/Homer	1e-118	-2.732e+02	0.0000	10051.0	18.30%	7972.9	14.68%	motif file (matrix)	svg
29	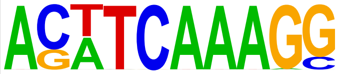	TCFL2(HMG)/K562-TCF7L2-ChIP-Seq(GSE29196)/Homer	1e-91	-2.098e+02	0.0000	2056.0	3.74%	1262.5	2.33%	motif file (matrix)	svg
30	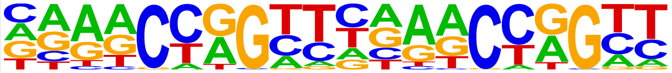	Tcfcp2l1(CP2)/mES-Tcfcp2l1-ChIP-Seq(GSE11431)/Homer	1e-90	-2.092e+02	0.0000	3838.0	6.99%	2712.4	5.00%	motif file (matrix)	svg
31	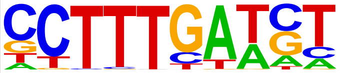	LEF1(HMG)/H1-LEF1-ChIP-Seq(GSE64758)/Homer	1e-90	-2.086e+02	0.0000	11586.0	21.09%	9620.5	17.72%	motif file (matrix)	svg
32	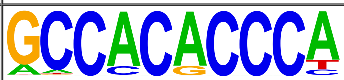	Klf4(Zf)/mES-Klf4-ChIP-Seq(GSE11431)/Homer	1e-83	-1.928e+02	0.0000	8989.0	16.36%	7304.4	13.45%	motif file (matrix)	svg
33	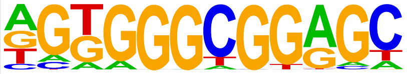	Sp5(Zf)/mES-Sp5.Flag-ChIP-Seq(GSE72989)/Homer	1e-83	-1.922e+02	0.0000	19405.0	35.32%	17071.1	31.44%	motif file (matrix)	svg
34	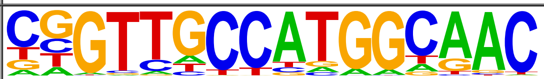	RFX(HTH)/K562-RFX3-ChIP-Seq(SRA012198)/Homer	1e-78	-1.800e+02	0.0000	2141.0	3.90%	1379.7	2.54%	motif file (matrix)	svg

35	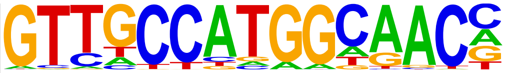	Rfx2(HTH)/LoVo-RFX2-ChIP-Seq(GSE49402)/Homer	1e-73	-1.689e+02	0.0000	2309.0	4.20%	1535.7	2.83%	motif file (matrix)	svg
36	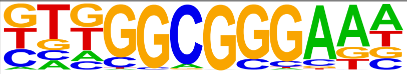	E2F3(E2F)/MEF-E2F3-ChIP-Seq(GSE71376)/Homer	1e-71	-1.642e+02	0.0000	9715.0	17.68%	8089.7	14.90%	motif file (matrix)	svg
37	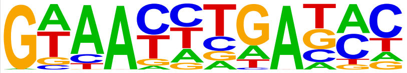	Six2(Homeobox)/NephronProgenitor-Six2-ChIP-Seq(GSE39837)/Homer	1e-63	-1.463e+02	0.0000	15354.0	27.95%	13464.8	24.80%	motif file (matrix)	svg
38	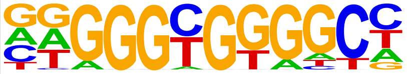	KLF1(Zf)/HUDEP2-KLF1-CutnRun(GSE136251)/Homer	1e-62	-1.443e+02	0.0000	17927.0	32.63%	15932.3	29.34%	motif file (matrix)	svg
39	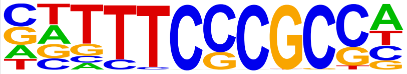	E2F7(E2F)/Hela-E2F7-ChIP-Seq(GSE32673)/Homer	1e-58	-1.351e+02	0.0000	2026.0	3.69%	1373.5	2.53%	motif file (matrix)	svg
40	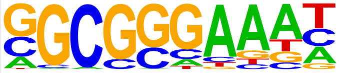	E2F4(E2F)/K562-E2F4-ChIP-Seq(GSE31477)/Homer	1e-58	-1.340e+02	0.0000	6368.0	11.59%	5163.3	9.51%	motif file (matrix)	svg
41	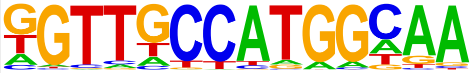	X-box(HTH)/NPC-H3K4me1-ChIP-Seq(GSE16256)/Homer	1e-54	-1.260e+02	0.0000	2386.0	4.34%	1692.1	3.12%	motif file (matrix)	svg
42	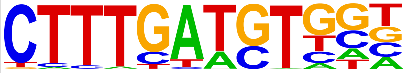	Tcf7(HMG)/GM12878-TCF7-ChIP-Seq(Encode)/Homer	1e-54	-1.248e+02	0.0000	6156.0	11.21%	5009.5	9.23%	motif file (matrix)	svg
43	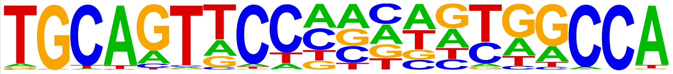	CTCF-SatelliteElement(Zf?)/CD4+-CTCF-ChIP-Seq(Barski_et_al.)/Homer	1e-53	-1.221e+02	0.0000	667.0	1.21%	341.3	0.63%	motif file (matrix)	svg
44	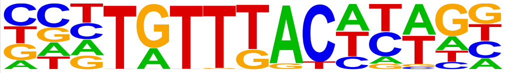	Foxa3(Forkhead)/Liver-Foxa3-ChIP-Seq(GSE77670)/Homer	1e-46	-1.082e+02	0.0000	5194.0	9.45%	4209.7	7.75%	motif file (matrix)	svg
45	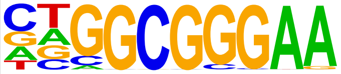	E2F1(E2F)/Hela-E2F1-ChIP-Seq(GSE22478)/Homer	1e-43	-1.009e+02	0.0000	3561.0	6.48%	2779.6	5.12%	motif file (matrix)	svg
46	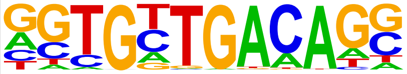	Tbx20(T-box)/Heart-Tbx20-ChIP-Seq(GSE29636)/Homer	1e-43	-9.922e+01	0.0000	4533.0	8.25%	3651.7	6.73%	motif file (matrix)	svg
47	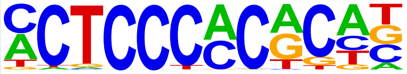	WT1(Zf)/Kidney-WT1-ChIP-Seq(GSE90016)/Homer	1e-35	-8.266e+01	0.0000	12080.0	21.99%	10762.8	19.82%	motif file (matrix)	svg
48	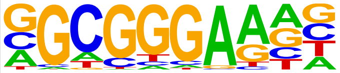	E2F6(E2F)/Hela-E2F6-ChIP-Seq(GSE31477)/Homer	1e-35	-8.218e+01	0.0000	8087.0	14.72%	7000.7	12.89%	motif file (matrix)	svg
49	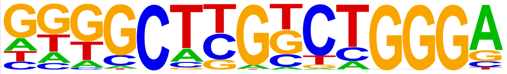	Zfp809(Zf)/ES-Zfp809-ChIP-Seq(GSE70799)/Homer	1e-34	-8.010e+01	0.0000	4299.0	7.83%	3521.1	6.48%	motif file (matrix)	svg
50	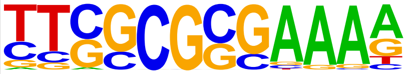	E2F(E2F)/Hela-CellCycle-Expression/Homer	1e-29	-6.740e+01	0.0000	860.0	1.57%	564.2	1.04%	motif file (matrix)	svg
51	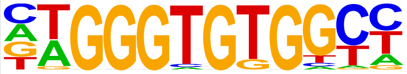	EKLF(Zf)/Erythrocyte-Klf1-ChIP-Seq(GSE20478)/Homer	1e-29	-6.714e+01	0.0000	4835.0	8.80%	4072.4	7.50%	motif file (matrix)	svg
52	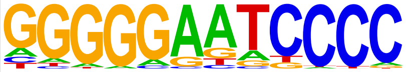	NFkB-p50,p52(RHD)/Monocyte-p50-ChIP-Chip(Schreiber_et_al.)/Homer	1e-27	-6.339e+01	0.0000	2211.0	4.02%	1722.5	3.17%	motif file (matrix)	svg
53	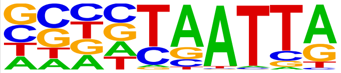	DLX1(Homeobox)/BasalGanglia-Dlx1-ChIP-seq(GSE124936)/Homer	1e-26	-6.022e+01	0.0000	17287.0	31.47%	15953.9	29.38%	motif file (matrix)	svg

54	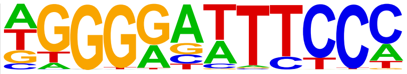	NFkB-p65(RHD)/GM12787-p65-ChIP-Seq(GSE19485)/Homer	1e-26	-6.006e+01	0.0000	8368.0	15.23%	7410.3	13.65%	motif file (matrix)	svg
55	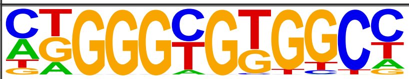	KLF6(Zf)/PDAC-KLF6-ChIP-Seq(GSE64557)/Homer	1e-25	-5.929e+01	0.0000	19346.0	35.22%	17962.1	33.08%	motif file (matrix)	svg
56	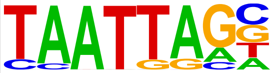	Lhx2(Homeobox)/HFSC-Lhx2-ChIP-Seq(GSE48068)/Homer	1e-21	-5.053e+01	0.0000	14252.0	25.94%	13114.2	24.15%	motif file (matrix)	svg
57	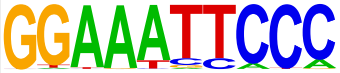	NFkB-p65-Rel(RHD)/ThioMac-LPS-Expression(GSE23622)/Homer	1e-21	-4.993e+01	0.0000	1108.0	2.02%	809.7	1.49%	motif file (matrix)	svg
58	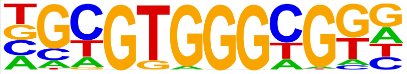	Egr2(Zf)/Thymocytes-Egr2-ChIP-Seq(GSE34254)/Homer	1e-20	-4.610e+01	0.0000	3541.0	6.45%	2997.9	5.52%	motif file (matrix)	svg
59	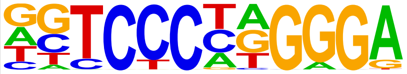	EBF(EBF)/proBcell-EBF-ChIP-Seq(GSE21978)/Homer	1e-19	-4.466e+01	0.0000	4244.0	7.73%	3654.0	6.73%	motif file (matrix)	svg
60	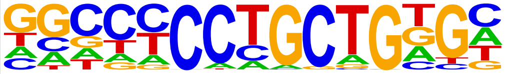	Zic3(Zf)/mES-Zic3-ChIP-Seq(GSE37889)/Homer	1e-18	-4.353e+01	0.0000	11701.0	21.30%	10728.5	19.76%	motif file (matrix)	svg
61	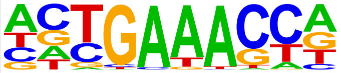	IRF4(IRF)/GM12878-IRF4-ChIP-Seq(GSE32465)/Homer	1e-17	-4.124e+01	0.0000	6520.0	11.87%	5809.5	10.70%	motif file (matrix)	svg
62	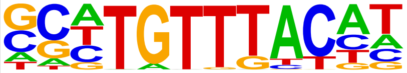	FOXK2(Forkhead)/U2OS-FOXK2-ChIP-Seq(E-MTAB-2204)/Homer	1e-17	-4.122e+01	0.0000	9192.0	16.73%	8347.9	15.37%	motif file (matrix)	svg
63	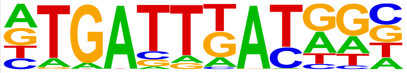	PBX2(Homeobox)/K562-PBX2-ChIP-Seq(Encode)/Homer	1e-16	-3.752e+01	0.0000	11189.0	20.37%	10298.8	18.97%	motif file (matrix)	svg
64	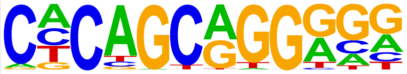	Zic2(Zf)/ESC-Zic2-ChIP-Seq(SRP197560)/Homer	1e-15	-3.661e+01	0.0000	9156.0	16.67%	8357.9	15.39%	motif file (matrix)	svg
65	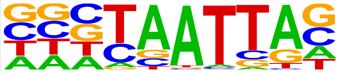	DLX2(Homeobox)/BasalGanglia-Dlx2-ChIP-seq(GSE124936)/Homer	1e-14	-3.287e+01	0.0000	19041.0	34.66%	17974.1	33.10%	motif file (matrix)	svg
66	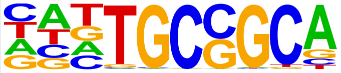	Zfp57(Zf)/H1-ZFP57.HA-ChIP-Seq(GSE115387)/Homer	1e-14	-3.265e+01	0.0000	5286.0	9.62%	4714.2	8.68%	motif file (matrix)	svg
67	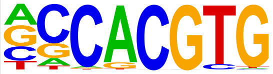	c-Myc(bHLH)/LNCAP-cMyc-ChIP-Seq(Unpublished)/Homer	1e-13	-3.180e+01	0.0000	7025.0	12.79%	6370.2	11.73%	motif file (matrix)	svg
68	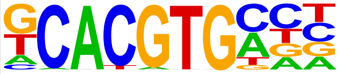	bHLHE40(bHLH)/HepG2-BHLHE40-ChIP-Seq(GSE31477)/Homer	1e-13	-3.152e+01	0.0000	4958.0	9.03%	4414.2	8.13%	motif file (matrix)	svg
69	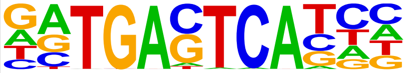	Jun-AP1(bZIP)/K562-cJun-ChIP-Seq(GSE31477)/Homer	1e-13	-2.998e+01	0.0000	3627.0	6.60%	3177.0	5.85%	motif file (matrix)	svg
70	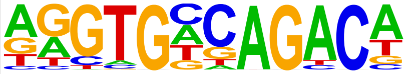	Tbox:Smad(T-box,MAD)/ESCd5-Smad2_3-ChIP-Seq(GSE29422)/Homer	1e-11	-2.612e+01	0.0000	3798.0	6.91%	3366.2	6.20%	motif file (matrix)	svg
71	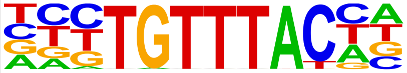	FOXP1(Forkhead)/H9-FOXP1-ChIP-Seq(GSE31006)/Homer	1e-11	-2.563e+01	0.0000	6322.0	11.51%	5761.4	10.61%	motif file (matrix)	svg
72	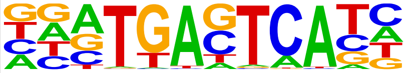	Fra1(bZIP)/BT549-Fra1-ChIP-Seq(GSE46166)/Homer	1e-11	-2.539e+01	0.0000	7657.0	13.94%	7040.2	12.97%	motif file (matrix)	svg

73	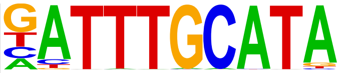	Oct11(POU,Homeobox)/NCIH1048-POU2F3-ChIP-seq(GSE115123)/Homer	1e-10	-2.514e+01	0.0000	4188.0	7.62%	3741.0	6.89%	motif file (matrix)	svg
74	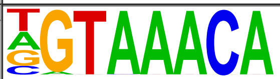	Foxo3(Forkhead)/U2OS-Foxo3-ChIP-Seq(E-MTAB-2701)/Homer	1e-10	-2.437e+01	0.0000	10146.0	18.47%	9447.2	17.40%	motif file (matrix)	svg
75	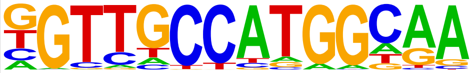	Rfx1(HTH)/NPC-H3K4me1-ChIP-Seq(GSE16256)/Homer	1e-10	-2.338e+01	0.0000	3838.0	6.99%	3426.8	6.31%	motif file (matrix)	svg
76	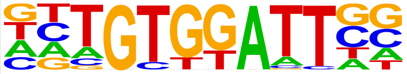	Foxh1(Forkhead)/hESC-FOXH1-ChIP-Seq(GSE29422)/Homer	1e-9	-2.265e+01	0.0000	8426.0	15.34%	7811.5	14.39%	motif file (matrix)	svg
77	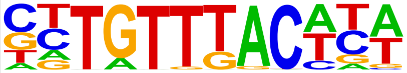	Foxa2(Forkhead)/Liver-Foxa2-ChIP-Seq(GSE25694)/Homer	1e-9	-2.168e+01	0.0000	11599.0	21.11%	10890.4	20.06%	motif file (matrix)	svg
78	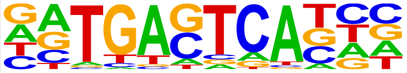	Fosl2(bZIP)/3T3L1-Fosl2-ChIP-Seq(GSE56872)/Homer	1e-9	-2.165e+01	0.0000	4947.0	9.01%	4492.1	8.27%	motif file (matrix)	svg
79	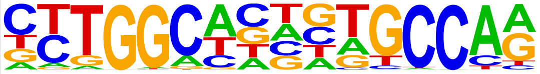	NF1(CTF)/LNCAP-NF1-ChIP-Seq(Unpublished)/Homer	1e-8	-1.948e+01	0.0000	7216.0	13.14%	6687.1	12.32%	motif file (matrix)	svg
80	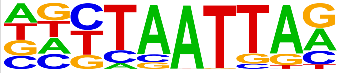	Lhx1(Homeobox)/EmbryoCarcinoma-Lhx1-ChIP-Seq(GSE70957)/Homer	1e-8	-1.934e+01	0.0000	14139.0	25.74%	13396.0	24.67%	motif file (matrix)	svg
81	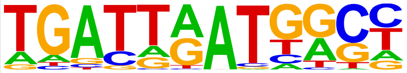	Hoxb4(Homeobox)/ES-Hoxb4-ChIP-Seq(GSE34014)/Homer	1e-8	-1.866e+01	0.0000	2910.0	5.30%	2592.7	4.77%	motif file (matrix)	svg
82	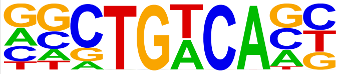	Meis1(Homeobox)/MastCells-Meis1-ChIP-Seq(GSE48085)/Homer	1e-7	-1.808e+01	0.0000	27087.0	49.31%	26130.5	48.12%	motif file (matrix)	svg
83	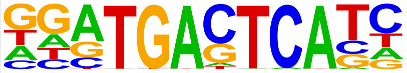	Fra2(bZIP)/Striatum-Fra2-ChIP-Seq(GSE43429)/Homer	1e-7	-1.785e+01	0.0000	6721.0	12.23%	6232.0	11.48%	motif file (matrix)	svg
84	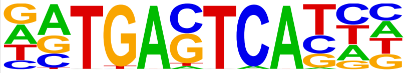	Atf3(bZIP)/GBM-ATF3-ChIP-Seq(GSE33912)/Homer	1e-7	-1.713e+01	0.0000	9035.0	16.45%	8474.6	15.61%	motif file (matrix)	svg
85	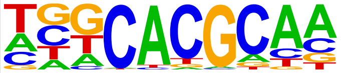	Arnt:Ahr(bHLH)/MCF7-Arnt-ChIP-Seq(Lo_et_al.)/Homer	1e-7	-1.701e+01	0.0000	10246.0	18.65%	9649.4	17.77%	motif file (matrix)	svg
86	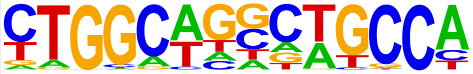	Tlx?(NR)/NPC-H3K4me1-ChIP-Seq(GSE16256)/Homer	1e-7	-1.662e+01	0.0000	8597.0	15.65%	8058.6	14.84%	motif file (matrix)	svg
87	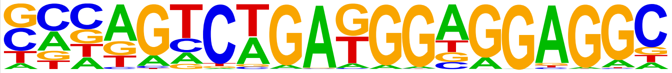	ZSCAN22(Zf)/HEK293-ZSCAN22.GFP-ChIP-Seq(GSE58341)/Homer	1e-6	-1.540e+01	0.0000	1569.0	2.86%	1363.5	2.51%	motif file (matrix)	svg
88	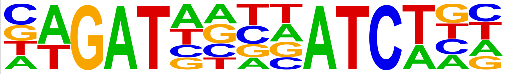	GATA(Zf),IR4/iTreg-Gata3-ChIP-Seq(GSE20898)/Homer	1e-6	-1.529e+01	0.0000	1033.0	1.88%	870.1	1.60%	motif file (matrix)	svg
89	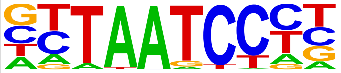	Otx2(Homeobox)/EpiLC-Otx2-ChIP-Seq(GSE56098)/Homer	1e-6	-1.496e+01	0.0000	12968.0	23.61%	12332.9	22.71%	motif file (matrix)	svg
90	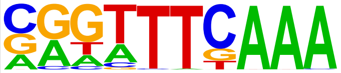	CHR(?)/Hela-CellCycle-Expression/Homer	1e-6	-1.453e+01	0.0000	7742.0	14.09%	7263.4	13.38%	motif file (matrix)	svg
91	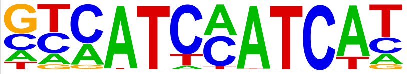	HOXA2(Homeobox)/mES-Hoxa2-ChIP-Seq(Donaldson_et_al.)/Homer	1e-5	-1.368e+01	0.0000	1555.0	2.83%	1362.3	2.51%	motif file (matrix)	svg

92	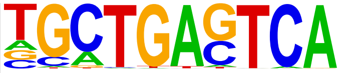	Bach2(bZIP)/OCILy7-Bach2-ChIP-Seq(GSE44420)/Homer	1e-5	-1.261e+01	0.0000	3057.0	5.56%	2788.7	5.14%	motif file (matrix)	svg
93	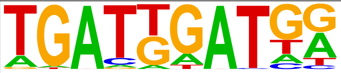	HOXA1(Homeobox)/mES-Hoxa1-ChIP-Seq(SRP084292)/Homer	1e-5	-1.208e+01	0.0000	3640.0	6.63%	3350.2	6.17%	motif file (matrix)	svg
94	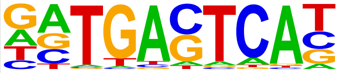	JunB(bZIP)/DendriticCells-Junb-ChIP-Seq(GSE36099)/Homer	1e-5	-1.169e+01	0.0000	7561.0	13.76%	7134.9	13.14%	motif file (matrix)	svg
95	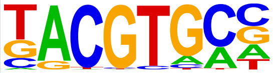	HIF-1a(bHLH)/MCF7-HIF1a-ChIP-Seq(GSE28352)/Homer	1e-4	-1.150e+01	0.0000	3620.0	6.59%	3338.0	6.15%	motif file (matrix)	svg
96	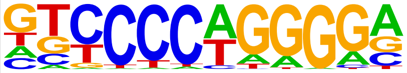	EBF1(EBF)/Near-E2A-ChIP-Seq(GSE21512)/Homer	1e-4	-1.127e+01	0.0001	18571.0	33.81%	17896.9	32.96%	motif file (matrix)	svg
97	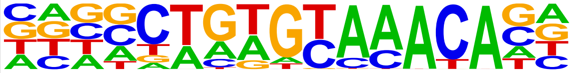	Fox:Ebox(Forkhead,bHLH)/Panc1-Foxa2-ChIP-Seq(GSE47459)/Homer	1e-4	-1.034e+01	0.0001	16263.0	29.60%	15654.8	28.83%	motif file (matrix)	svg
98	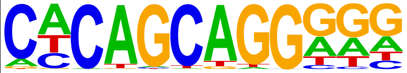	Unknown-ESC-element(?)/mES-Nanog-ChIP-Seq(GSE11724)/Homer	1e-4	-9.997e+00	0.0002	11525.0	20.98%	11025.9	20.31%	motif file (matrix)	svg
99	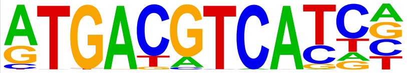	JunD(bZIP)/K562-JunD-ChIP-Seq/Homer	1e-4	-9.361e+00	0.0004	1312.0	2.39%	1168.2	2.15%	motif file (matrix)	svg
100	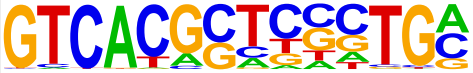	PAX5(Paired,Homeobox),condensed/GM12878-PAX5-ChIP-Seq(GSE32465)/Homer	1e-3	-9.033e+00	0.0005	1847.0	3.36%	1676.2	3.09%	motif file (matrix)	svg
101	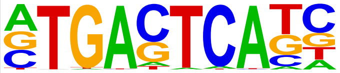	AP-1(bZIP)/ThioMac-PU.1-ChIP-Seq(GSE21512)/Homer	1e-3	-8.531e+00	0.0009	10196.0	18.56%	9761.7	17.98%	motif file (matrix)	svg
102	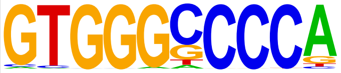	ZNF692(Zf)/HEK293-ZNF692.GFP-ChIP-Seq(GSE58341)/Homer	1e-2	-6.534e+00	0.0062	2414.0	4.39%	2247.7	4.14%	motif file (matrix)	svg
103	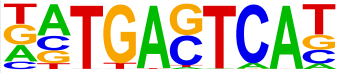	BATF(bZIP)/Th17-BATF-ChIP-Seq(GSE39756)/Homer	1e-2	-6.515e+00	0.0063	8896.0	16.19%	8541.9	15.73%	motif file (matrix)	svg
104	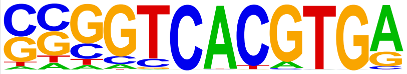	E-box(bHLH)/Promoter/Homer	1e-2	-6.442e+00	0.0067	1118.0	2.04%	1011.8	1.86%	motif file (matrix)	svg
105	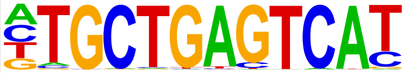	Nrf2(bZIP)/Lymphoblast-Nrf2-ChIP-Seq(GSE37589)/Homer	1e-2	-5.529e+00	0.0166	765.0	1.39%	686.5	1.26%	motif file (matrix)	svg
106	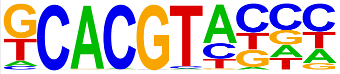	HIF2a(bHLH)/785_O-HIF2a-ChIP-Seq(GSE34871)/Homer	1e-2	-5.404e+00	0.0186	5020.0	9.14%	4789.2	8.82%	motif file (matrix)	svg
107	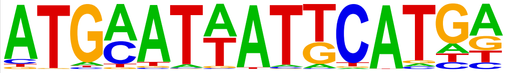	OCT:OCT(POU,Homeobox,IR1)/NPC-Brn2-ChIP-Seq(GSE35496)/Homer	1e-2	-5.396e+00	0.0186	192.0	0.35%	156.8	0.29%	motif file (matrix)	svg
108	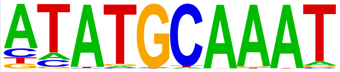	Oct2(POU,Homeobox)/Bcell-Oct2-ChIP-Seq(GSE21512)/Homer	1e-2	-5.061e+00	0.0257	3398.0	6.19%	3221.5	5.93%	motif file (matrix)	svg
109	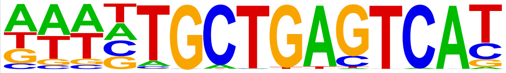	Bach1(bZIP)/K562-Bach1-ChIP-Seq(GSE31477)/Homer	1e-2	-5.027e+00	0.0264	810.0	1.47%	733.0	1.35%	motif file (matrix)	svg
110	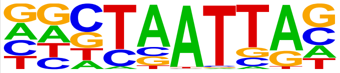	En1(Homeobox)/SUM149-EN1-ChIP-Seq(GSE120957)/Homer	1e-2	-5.001e+00	0.0268	22565.0	41.08%	22022.4	40.56%	motif file (matrix)	svg

111	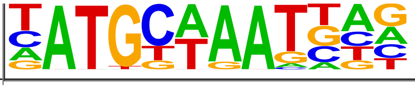	Brn1(POU,Homeobox)/NPC-Brn1-ChIP-Seq(GSE35496)/Homer	1e-2	-4.687e+00	0.0364	4073.0	7.41%	3884.9	7.15%	motif file (matrix)	svg
