# Supplementary material for: AP-2α and AP-2β cooperatively function in the craniofacial surface ectoderm to regulate chromatin and gene expression dynamics during facial development
Source: eLife. 2022 Mar 25;11:e70511. doi: 10.7554/eLife.70511 (PMC9038197; doi:10.7554/eLife.70511)
Supplement: Figure 2—source data 3. [file elife-70511-fig2-data3.pdf]

## Job Description

Job ID: 20201106-public-4.0.4-pOiBTG

Display name: cluster_1_galaxy.bed

Test set: cluster_1_galaxy.bed (10,363 genomic regions)
Show in UCSC genome browser. *How do I look at my regions in the genome?*

Background: Whole genome background

Assembly: Mouse: NCBI build 38 (UCSC mm10, Dec. 2011) *What gene set does GREAT use?*

Associated genomic regions: Basal+extension (constitutive 5.0 kb upstream and 1.0 kb downstream, up to 100.0 kb max extension). Curated regulatory domains are included.
137 of all 10,363 genomic regions (1.3%) are not associated with any genes.
View all genomic region-gene associations. *Which genes are my regions associated with?*
Revise the region-gene association rule. *How are my regions associated with genes?*

## Region-Gene Association Graphs

*What do these graphs illustrate?*

**Number of associated genes per region**

Download as PDF.

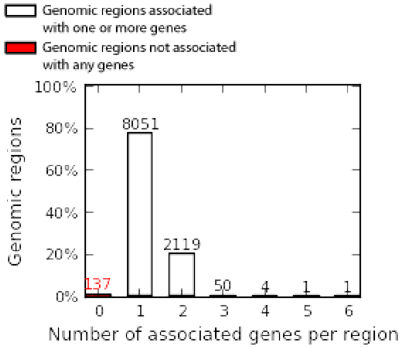


**Binned by orientation and distance to TSS**

Download as PDF.

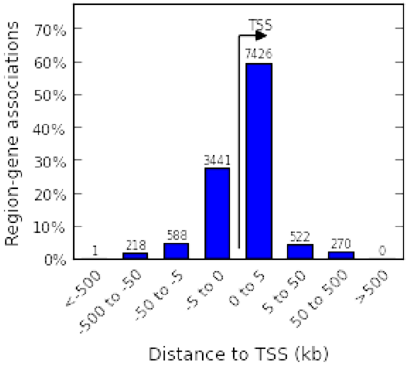


**Binned by absolute distance to TSS**

Download as PDF.

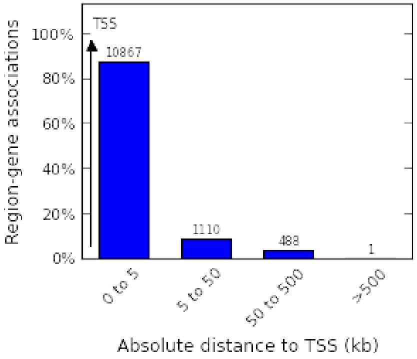


## Global Controls

Global Export *Which data is exported by each option?*

## Ensembl Genes (no terms)

Global controls

## GO Biological Process (20+ terms)

Global controls

*Table controls:* Export | Shown top rows in this table: 20 Set | Term annotation count: Min: 1 Max: Inf Set | Visualize this table: [select one]

| Term Name | Binom Rank | Binom Raw P-Value | *Binom FDR Q-Val* | Binom Fold Enrichment | Binom Observed Region Hits | Binom Region Set Coverage | Hyper Rank | *Hyper FDR Q-Val* | Hyper Fold Enrichment | Hyper Observed Gene Hits | Hyper Total Genes | Hyper Gene Set Coverage |
|---|---|---|---|---|---|---|---|---|---|---|---|---|
| **cellular metabolic process** | 1 | **0.0000** | **0.0000** | 2.1754 | 5,642 | 54.44% | 1 | **0.0000** | 1.3744 | 5,299 | 7,645 | 49.11% |
| **cellular macromolecule metabolic process** | 1 | **0.0000** | **0.0000** | 2.2797 | 4,758 | 45.91% | 1 | **0.0000** | 1.4471 | 4,341 | 5,948 | 40.23% |
| **primary metabolic process** | 1 | **0.0000** | **0.0000** | 2.1537 | 5,656 | 54.58% | 3 | **4.1172e-315** | 1.3374 | 5,288 | 7,840 | 49.01% |
| **organic substance metabolic process** | 1 | **0.0000** | **0.0000** | 2.1356 | 5,863 | 56.58% | 4 | **7.1684e-314** | 1.3241 | 5,495 | 8,229 | 50.93% |

| Term Name | Binom Rank | Binom Raw P-Value | *Binom FDR Q-Val* | Binom Fold Enrichment | Binom Observed Region Hits | Binom Region Set Coverage | Hyper Rank | *Hyper FDR Q-Val* | Hyper Fold Enrichment | Hyper Observed Gene Hits | Hyper Total Genes | Hyper Gene Set Coverage |
|---|---|---|---|---|---|---|---|---|---|---|---|---|
| metabolic process | 1 | **0.0000** | **0.0000** | 2.1144 | 6,062 | 58.50% | 5 | **2.9869e-311** | 1.3088 | 5,732 | 8,684 | 53.12% |
| macromolecule metabolic process | 1 | **0.0000** | **0.0000** | 2.2024 | 5,026 | 48.50% | 6 | **5.5768e-311** | 1.3802 | 4,601 | 6,610 | 42.64% |
| cellular nitrogen compound metabolic process | 1 | **0.0000** | **0.0000** | 2.3494 | 3,605 | 34.79% | 7 | **1.2216e-260** | 1.4552 | 3,224 | 4,393 | 29.88% |
| nitrogen compound metabolic process | 1 | **0.0000** | **0.0000** | 2.2888 | 3,792 | 36.59% | 8 | **1.6901e-252** | 1.4286 | 3,405 | 4,726 | 31.56% |
| nucleobase-containing compound metabolic process | 1 | **0.0000** | **0.0000** | 2.3600 | 3,203 | 30.91% | 9 | **5.2295e-240** | 1.4771 | 2,827 | 3,795 | 26.20% |
| heterocycle metabolic process | 1 | **0.0000** | **0.0000** | 2.3443 | 3,277 | 31.62% | 10 | **1.5912e-235** | 1.4635 | 2,897 | 3,925 | 26.85% |
| nucleic acid metabolic process | 1 | **0.0000** | **0.0000** | 2.4180 | 2,898 | 27.96% | 11 | **6.6600e-231** | 1.5060 | 2,517 | 3,314 | 23.33% |
| cellular aromatic compound metabolic process | 1 | **0.0000** | **0.0000** | 2.3173 | 3,293 | 31.78% | 12 | **1.2004e-228** | 1.4537 | 2,912 | 3,972 | 26.99% |
| organic cyclic compound metabolic process | 1 | **0.0000** | **0.0000** | 2.2870 | 3,389 | 32.70% | 13 | **2.9937e-220** | 1.4337 | 3,005 | 4,156 | 27.85% |
| cellular component organization or biogenesis | 1 | **0.0000** | **0.0000** | 2.0322 | 3,781 | 36.49% | 14 | **2.9067e-195** | 1.3650 | 3,440 | 4,997 | 31.88% |
| gene expression | 1 | **0.0000** | **0.0000** | 2.3642 | 2,742 | 26.46% | 15 | **2.5992e-192** | 1.4760 | 2,353 | 3,161 | 21.81% |
| cellular protein metabolic process | 1 | **0.0000** | **0.0000** | 2.2199 | 2,577 | 24.87% | 16 | **3.7990e-183** | 1.4649 | 2,336 | 3,162 | 21.65% |
| cellular biosynthetic process | 1 | **0.0000** | **0.0000** | 2.1863 | 2,993 | 28.88% | 17 | **1.9868e-173** | 1.4149 | 2,628 | 3,683 | 24.36% |
| RNA metabolic process | 1 | **0.0000** | **0.0000** | 2.3580 | 2,498 | 24.10% | 18 | **1.2928e-171** | 1.4766 | 2,129 | 2,859 | 19.73% |
| organic substance biosynthetic process | 1 | **0.0000** | **0.0000** | 2.1791 | 3,035 | 29.29% | 19 | **1.8193e-170** | 1.4070 | 2,661 | 3,750 | 24.66% |
| biosynthetic process | 1 | **0.0000** | **0.0000** | 2.1688 | 3,078 | 29.70% | 20 | **3.1964e-169** | 1.4004 | 2,707 | 3,833 | 25.09% |

The test set of 10,363 genomic regions picked 10,790 (50%) of all 21,395 genes.
*GO Biological Process* has 13,090 terms covering 17,925 (84%) of all 21,395 genes, and 1,163,819 term - gene associations.
13,090 ontology terms (100%) were tested using an annotation count range of [1, Inf].

## GO Cellular Component (20+ terms)

Global controls

*Table controls:* Export | Shown top rows in this table: 20 Set | Term annotation count: Min: 1 Max: Inf Set | Visualize this table: [select one]

| Term Name | Binom Rank | Binom Raw P-Value | *Binom FDR Q-Val* | Binom Fold Enrichment | Binom Observed Region Hits | Binom Region Set Coverage | Hyper Rank | *Hyper FDR Q-Val* | Hyper Fold Enrichment | Hyper Observed Gene Hits | Hyper Total Genes | Hyper Gene Set Coverage |
|---|---|---|---|---|---|---|---|---|---|---|---|---|
| intracellular | 1 | **0.0000** | **0.0000** | 2.1073 | 8,920 | 86.08% | 1 | **0.0000** | 1.2738 | 8,917 | 13,881 | 82.64% |
| nucleus | 1 | **0.0000** | **0.0000** | 2.2418 | 5,293 | 51.08% | 1 | **0.0000** | 1.4167 | 4,857 | 6,798 | 45.01% |
| cytoplasm | 1 | **0.0000** | **0.0000** | 2.0990 | 7,091 | 68.43% | 1 | **0.0000** | 1.3167 | 6,928 | 10,433 | 64.21% |
| organelle | 1 | **0.0000** | **0.0000** | 2.1049 | 8,418 | 81.23% | 1 | **0.0000** | 1.2831 | 8,323 | 12,862 | 77.14% |
| membrane-bounded organelle | 1 | **0.0000** | **0.0000** | 2.1348 | 7,982 | 77.02% | 1 | **0.0000** | 1.3091 | 7,793 | 11,804 | 72.22% |
| intracellular organelle | 1 | **0.0000** | **0.0000** | 2.1677 | 8,052 | 77.70% | 1 | **0.0000** | 1.3272 | 7,899 | 11,801 | 73.21% |
| intracellular membrane-bounded organelle | 1 | **0.0000** | **0.0000** | 2.2281 | 7,308 | 70.52% | 1 | **0.0000** | 1.3825 | 7,069 | 10,139 | 65.51% |

| Term Name | Binom Rank | Binom Raw P-Value | *Binom FDR Q-Val* | Binom Fold Enrichment | Binom Observed Region Hits | Binom Region Set Coverage | Hyper Rank | *Hyper FDR Q-Val* | Hyper Fold Enrichment | Hyper Observed Gene Hits | Hyper Total Genes | Hyper Gene Set Coverage |
|---|---|---|---|---|---|---|---|---|---|---|---|---|
| organelle lumen | 1 | **0.0000** | **0.0000** | 2.4688 | 3,544 | 34.20% | 1 | **0.0000** | 1.5641 | 3,205 | 4,063 | 29.70% |
| organelle part | 1 | **0.0000** | **0.0000** | 2.2681 | 6,055 | 58.43% | 1 | **0.0000** | 1.4123 | 5,790 | 8,129 | 53.66% |
| intracellular part | 1 | **0.0000** | **0.0000** | 2.1227 | 8,758 | 84.51% | 1 | **0.0000** | 1.2885 | 8,738 | 13,447 | 80.98% |
| nuclear part | 1 | **0.0000** | **0.0000** | 2.4301 | 3,502 | 33.79% | 1 | **0.0000** | 1.5512 | 3,141 | 4,015 | 29.11% |
| cytoplasmic part | 1 | **0.0000** | **0.0000** | 2.1439 | 5,750 | 55.49% | 1 | **0.0000** | 1.3627 | 5,511 | 8,019 | 51.08% |
| intracellular organelle part | 1 | **0.0000** | **0.0000** | 2.3042 | 5,979 | 57.70% | 1 | **0.0000** | 1.4314 | 5,703 | 7,900 | 52.85% |
| intracellular organelle lumen | 1 | **0.0000** | **0.0000** | 2.4720 | 3,543 | 34.19% | 1 | **0.0000** | 1.5648 | 3,204 | 4,060 | 29.69% |
| nuclear lumen | 1 | **0.0000** | **0.0000** | 2.4464 | 3,166 | 30.55% | 15 | **5.6226e-318** | 1.5634 | 2,818 | 3,574 | 26.12% |
| nucleoplasm | 1 | **0.0000** | **0.0000** | 2.5392 | 2,492 | 24.05% | 16 | **5.3197e-280** | 1.6201 | 2,193 | 2,684 | 20.32% |
| macromolecular complex | 1 | **0.0000** | **0.0000** | 2.2565 | 3,964 | 38.25% | 17 | **4.8913e-276** | 1.4257 | 3,667 | 5,100 | 33.99% |
| cytosol | 1 | **0.0000** | **0.0000** | 2.2801 | 2,807 | 27.09% | 18 | **1.6815e-214** | 1.4842 | 2,509 | 3,352 | 23.25% |
| protein complex | 1 | **0.0000** | **0.0000** | 2.3392 | 2,665 | 25.72% | 19 | **1.7751e-193** | 1.4738 | 2,371 | 3,190 | 21.97% |
| intracellular non-membrane-bounded organelle | 1 | **0.0000** | **0.0000** | 2.2206 | 3,222 | 31.09% | 20 | **1.0608e-179** | 1.3930 | 2,905 | 4,135 | 26.92% |

The test set of 10,363 genomic regions picked 10,790 (50%) of all 21,395 genes.
*GO Cellular Component* has 1,694 terms covering 19,074 (89%) of all 21,395 genes, and 371,380 term - gene associations.
1,694 ontology terms (100%) were tested using an annotation count range of [1, Inf].

## GO Molecular Function (20+ terms)

Global controls

*Table controls:* Export Shown top rows in this table: 20 Set Term annotation count: Min: 1 Max: Inf Set Visualize this table: [select one]

| Term Name | Binom Rank | Binom Raw P-Value | *Binom FDR Q-Val* | Binom Fold Enrichment | Binom Observed Region Hits | Binom Region Set Coverage | Hyper Rank | *Hyper FDR Q-Val* | Hyper Fold Enrichment | Hyper Observed Gene Hits | Hyper Total Genes | Hyper Gene Set Coverage |
|---|---|---|---|---|---|---|---|---|---|---|---|---|
| heterocyclic compound binding | 1 | **0.0000** | **0.0000** | 2.1803 | 4,445 | 42.89% | 1 | **1.1660e-203** | 1.3331 | 3,993 | 5,939 | 37.01% |
| organic cyclic compound binding | 1 | **0.0000** | **0.0000** | 2.1609 | 4,474 | 43.17% | 2 | **1.4474e-192** | 1.3205 | 4,017 | 6,032 | 37.23% |
| nucleic acid binding | 1 | **0.0000** | **0.0000** | 2.3833 | 3,267 | 31.53% | 3 | **1.1318e-187** | 1.4118 | 2,836 | 3,983 | 26.28% |
| RNA binding | 8 | **2.8284e-298** | **1.4605e-295** | 2.8352 | 1,584 | 15.29% | 4 | **9.8560e-177** | 1.6399 | 1,382 | 1,671 | 12.81% |
| transferase activity | 11 | **4.2811e-186** | **1.6078e-183** | 2.0565 | 1,812 | 17.49% | 8 | **2.3413e-94** | 1.3992 | 1,647 | 2,334 | 15.26% |
| DNA binding | 12 | **3.8927e-177** | **1.3401e-174** | 2.0867 | 1,680 | 16.21% | 25 | **6.8530e-44** | 1.2896 | 1,371 | 2,108 | 12.71% |
| enzyme binding | 13 | **7.0419e-166** | **2.2377e-163** | 2.1008 | 1,560 | 15.05% | 9 | **2.7784e-84** | 1.4196 | 1,376 | 1,922 | 12.75% |
| hydrolase activity, acting on acid anhydrides | 33 | **1.4043e-63** | **1.7579e-61** | 2.0538 | 656 | 6.33% | 41 | **1.9260e-26** | 1.3631 | 585 | 851 | 5.42% |
| hydrolase activity, acting on acid anhydrides, in phosphorus-containing anhydrides | 34 | **1.6013e-63** | **1.9456e-61** | 2.0543 | 655 | 6.32% | 40 | **1.6960e-26** | 1.3639 | 584 | 849 | 5.41% |
| pyrophosphatase activity | 35 | **2.9674e-63** | **3.5024e-61** | 2.0534 | 653 | 6.30% | 42 | **3.1041e-26** | 1.3625 | 582 | 847 | 5.39% |

| Term Name | Binom Rank | Binom Raw P-Value | *Binom FDR Q-Val* | Binom Fold Enrichment | Binom Observed Region Hits | Binom Region Set Coverage | Hyper Rank | *Hyper FDR Q-Val* | Hyper Fold Enrichment | Hyper Observed Gene Hits | Hyper Total Genes | Hyper Gene Set Coverage |
|---|---|---|---|---|---|---|---|---|---|---|---|---|
| transcription factor binding | 36 | **5.7220e-63** | **6.5660e-61** | 2.2333 | 539 | 5.20% | 30 | **5.0014e-34** | 1.5057 | 426 | 561 | 3.95% |
| kinase binding | 37 | **1.8289e-60** | **2.0419e-58** | 2.1551 | 559 | 5.39% | 35 | **8.3316e-31** | 1.4502 | 471 | 644 | 4.37% |
| chromatin binding | 38 | **2.7096e-59** | **2.9456e-57** | 2.2020 | 523 | 5.05% | 36 | **1.6717e-30** | 1.4801 | 418 | 560 | 3.87% |
| nucleoside-triphosphatase activity | 39 | **8.5414e-59** | **9.0473e-57** | 2.0438 | 614 | 5.92% | 49 | **2.2088e-23** | 1.3515 | 548 | 804 | 5.08% |
| protein kinase binding | 43 | **6.5958e-52** | **6.3365e-50** | 2.1325 | 491 | 4.74% | 39 | **5.7872e-27** | 1.4481 | 417 | 571 | 3.86% |
| ubiquitin-like protein transferase activity | 44 | **5.3931e-51** | **5.0634e-49** | 2.2971 | 411 | 3.97% | 31 | **2.1725e-33** | 1.5518 | 360 | 460 | 3.34% |
| ubiquitin-protein transferase activity | 46 | **1.1365e-49** | **1.0206e-47** | 2.3060 | 397 | 3.83% | 32 | **4.9046e-33** | 1.5602 | 347 | 441 | 3.22% |
| transcription factor activity, protein binding | 47 | **4.7643e-49** | **4.1875e-47** | 2.1159 | 472 | 4.55% | 48 | **1.0574e-23** | 1.4397 | 379 | 522 | 3.51% |
| transcription factor activity, transcription factor binding | 48 | **3.1485e-47** | **2.7097e-45** | 2.1024 | 461 | 4.45% | 53 | **1.9132e-22** | 1.4312 | 371 | 514 | 3.44% |
| transcription cofactor activity | 52 | **3.8286e-43** | **3.0415e-41** | 2.1441 | 402 | 3.88% | 56 | **5.8032e-20** | 1.4380 | 322 | 444 | 2.98% |

The test set of 10,363 genomic regions picked 10,790 (50%) of all 21,395 genes.
*GO Molecular Function* has 4,131 terms covering 17,189 (80%) of all 21,395 genes, and 227,341 term - gene associations.
4,131 ontology terms (100%) were tested using an annotation count range of [1, Inf].

## Human Phenotype (20+ terms)

Global controls

*Table controls:* Export | Shown top rows in this table: 20 Set | Term annotation count: Min: 1 Max: Inf Set | Visualize this table: [select one]

| Term Name | Binom Rank | Binom Raw P-Value | *Binom FDR Q-Val* | Binom Fold Enrichment | Binom Observed Region Hits | Binom Region Set Coverage | Hyper Rank | *Hyper FDR Q-Val* | Hyper Fold Enrichment | Hyper Observed Gene Hits | Hyper Total Genes | Hyper Gene Set Coverage |
|---|---|---|---|---|---|---|---|---|---|---|---|---|
| Abnormality of the nervous system | 1 | **7.7393e-171** | **5.1072e-167** | 2.0426 | 1,702 | 16.42% | 14 | **2.1253e-90** | 1.4187 | 1,471 | 2,056 | 13.63% |
| Abnormality of nervous system physiology | 2 | **1.7719e-163** | **5.8465e-160** | 2.0832 | 1,567 | 15.12% | 10 | **2.9540e-93** | 1.4483 | 1,360 | 1,862 | 12.60% |
| Abnormality of nervous system morphology | 3 | **2.3863e-151** | **5.2491e-148** | 2.1703 | 1,339 | 12.92% | 6 | **1.4349e-98** | 1.5105 | 1,164 | 1,528 | 10.79% |
| Morphological abnormality of the central nervous system | 4 | **5.4701e-149** | **9.0242e-146** | 2.2360 | 1,242 | 11.98% | 2 | **5.0716e-105** | 1.5565 | 1,077 | 1,372 | 9.98% |
| Abnormality of the musculature | 5 | **1.0415e-147** | **1.3746e-144** | 2.2306 | 1,238 | 11.95% | 16 | **2.2242e-89** | 1.5046 | 1,079 | 1,422 | 10.00% |
| Abnormality of skeletal morphology | 6 | **1.2557e-146** | **1.3810e-143** | 2.1283 | 1,354 | 13.07% | 17 | **9.1054e-86** | 1.4710 | 1,161 | 1,565 | 10.76% |
| Neurodevelopmental abnormality | 7 | **2.4964e-146** | **2.3534e-143** | 2.2280 | 1,230 | 11.87% | 3 | **6.5618e-102** | 1.5538 | 1,054 | 1,345 | 9.77% |
| Abnormality of brain morphology | 8 | **4.0281e-143** | **3.3226e-140** | 2.2747 | 1,156 | 11.16% | 1 | **3.4825e-108** | 1.5907 | 1,006 | 1,254 | 9.32% |
| Abnormality of the skeletal system | 9 | **5.6923e-142** | **4.1738e-139** | 2.0823 | 1,377 | 13.29% | 20 | **9.3095e-79** | 1.4430 | 1,184 | 1,627 | 10.97% |
| Abnormality of head or neck | 10 | **1.0100e-140** | **6.6649e-138** | 2.0752 | 1,376 | 13.28% | 23 | **1.0168e-77** | 1.4394 | 1,184 | 1,631 | 10.97% |
| Abnormality of the head | 11 | **3.4607e-140** | **2.0761e-137** | 2.0801 | 1,364 | 13.16% | 21 | **1.9749e-78** | 1.4449 | 1,171 | 1,607 | 10.85% |
| Abnormality of the eye | 12 | **1.2155e-134** | **6.6843e-132** | 2.0871 | 1,304 | 12.58% | 24 | **2.1388e-75** | 1.4457 | 1,125 | 1,543 | 10.43% |

| Term Name | Binom Rank | Binom Raw P-Value | *Binom FDR Q-Val* | Binom Fold Enrichment | Binom Observed Region Hits | Binom Region Set Coverage | Hyper Rank | *Hyper FDR Q-Val* | Hyper Fold Enrichment | Hyper Observed Gene Hits | Hyper Total Genes | Hyper Gene Set Coverage |
|---|---|---|---|---|---|---|---|---|---|---|---|---|
| Abnormal axial skeleton morphology | 13 | **1.5110e-133** | **7.6701e-131** | 2.2080 | 1,149 | 11.09% | 15 | **6.9730e-90** | 1.5336 | 990 | 1,280 | 9.18% |
| Abnormality of muscle physiology | 14 | **4.8357e-131** | **2.2793e-128** | 2.2747 | 1,063 | 10.26% | 19 | **4.0202e-84** | 1.5313 | 936 | 1,212 | 8.67% |
| Neurodevelopmental delay | 15 | **2.3076e-126** | **1.0152e-123** | 2.3535 | 961 | 9.27% | 13 | **9.9819e-91** | 1.6040 | 813 | 1,005 | 7.53% |
| Abnormal muscle tone | 16 | **4.9795e-125** | **2.0537e-122** | 2.3818 | 930 | 8.97% | 12 | **1.4414e-91** | 1.6055 | 817 | 1,009 | 7.57% |
| Abnormality of the skull | 17 | **4.7307e-124** | **1.8364e-121** | 2.2871 | 998 | 9.63% | 11 | **7.0335e-93** | 1.5922 | 860 | 1,071 | 7.97% |
| Global developmental delay | 18 | **1.5712e-123** | **5.7603e-121** | 2.5634 | 805 | 7.77% | 8 | **9.1349e-96** | 1.6904 | 682 | 800 | 6.32% |
| Abnormality of the cerebrum | 20 | **3.2719e-120** | **1.0796e-117** | 2.3923 | 888 | 8.57% | 4 | **4.3467e-101** | 1.6658 | 767 | 913 | 7.11% |
| Abnormality of forebrain morphology | 21 | **6.8040e-120** | **2.1381e-117** | 2.3876 | 889 | 8.58% | 5 | **6.4452e-101** | 1.6643 | 768 | 915 | 7.12% |

The test set of 10,363 genomic regions picked 10,790 (50%) of all 21,395 genes.
*Human Phenotype* has 6,599 terms covering 3,215 (15%) of all 21,395 genes, and 244,972 term - gene associations.
6,599 ontology terms (100%) were tested using an annotation count range of [1, Inf].

## ⊖ Mouse Phenotype Single KO (20+ terms)

Global controls

*Table controls:* Export Shown top rows in this table: 20 Set Term annotation count: Min: 1 Max: Inf Set Visualize this table: [select one]

| Term Name | Binom Rank | Binom Raw P-Value | *Binom FDR Q-Val* | Binom Fold Enrichment | Binom Observed Region Hits | Binom Region Set Coverage | Hyper Rank | *Hyper FDR Q-Val* | Hyper Fold Enrichment | Hyper Observed Gene Hits | Hyper Total Genes | Hyper Gene Set Coverage |
|---|---|---|---|---|---|---|---|---|---|---|---|---|
| preweaning lethality | 1 | **0.0000** | **0.0000** | 2.0809 | 3,096 | 29.88% | 1 | **1.1384e-241** | 1.4963 | 2,697 | 3,574 | 25.00% |
| abnormal survival | 1 | **0.0000** | **0.0000** | 2.0402 | 3,376 | 32.58% | 2 | **7.5098e-241** | 1.4632 | 2,959 | 4,010 | 27.42% |
| prenatal lethality | 4 | **2.0302e-257** | **4.6542e-254** | 2.3251 | 1,908 | 18.41% | 3 | **3.4866e-218** | 1.6448 | 1,659 | 2,000 | 15.38% |
| embryonic lethality | 5 | **8.7049e-219** | **1.5965e-215** | 2.4369 | 1,514 | 14.61% | 5 | **2.2779e-200** | 1.7055 | 1,322 | 1,537 | 12.25% |
| embryo phenotype | 9 | **5.1922e-150** | **5.2903e-147** | 2.1021 | 1,420 | 13.70% | 7 | **5.9988e-119** | 1.5549 | 1,210 | 1,543 | 11.21% |
| embryonic lethality prior to tooth bud stage | 11 | **6.1674e-128** | **5.1414e-125** | 2.6749 | 775 | 7.48% | 6 | **1.2608e-126** | 1.8009 | 683 | 752 | 6.33% |
| embryonic lethality prior to organogenesis | 13 | **6.8683e-121** | **4.8448e-118** | 2.7423 | 704 | 6.79% | 8 | **1.3044e-117** | 1.8108 | 621 | 680 | 5.76% |
| abnormal embryo morphology | 14 | **9.9529e-117** | **6.5192e-114** | 2.1108 | 1,112 | 10.73% | 10 | **5.5429e-98** | 1.5815 | 934 | 1,171 | 8.66% |
| abnormal prenatal growth/weight/body size | 17 | **1.1297e-104** | **6.0938e-102** | 2.1446 | 968 | 9.34% | 9 | **5.1160e-100** | 1.6367 | 818 | 991 | 7.58% |
| preweaning lethality, complete penetrance | 18 | **1.7663e-103** | **8.9986e-101** | 2.4273 | 747 | 7.21% | 11 | **5.1414e-94** | 1.6916 | 668 | 783 | 6.19% |
| abnormal embryo development | 19 | **5.4444e-103** | **2.6276e-100** | 2.2575 | 856 | 8.26% | 13 | **4.9705e-90** | 1.6444 | 724 | 873 | 6.71% |
| abnormal embryonic growth/weight/body size | 22 | **9.1130e-90** | **3.7985e-87** | 2.1755 | 809 | 7.81% | 12 | **5.3138e-90** | 1.6649 | 686 | 817 | 6.36% |
| abnormal prenatal body size | 34 | **2.4610e-75** | **6.6375e-73** | 2.1097 | 729 | 7.03% | 16 | **2.4187e-74** | 1.6356 | 617 | 748 | 5.72% |
| abnormal cell morphology | 35 | **3.2559e-75** | **8.5305e-73** | 2.0077 | 817 | 7.88% | 31 | **1.1448e-37** | 1.3970 | 713 | 1,012 | 6.61% |
| abnormal embryonic tissue morphology | 36 | **5.0065e-75** | **1.2753e-72** | 2.0308 | 793 | 7.65% | 22 | **2.0910e-59** | 1.5407 | 655 | 843 | 6.07% |
| embryonic lethality during organogenesis | 37 | **9.6278e-75** | **2.3861e-72** | 2.1234 | 713 | 6.88% | 19 | **3.8086e-64** | 1.5959 | 598 | 743 | 5.54% |

| Term Name | Binom Rank | Binom Raw P-Value | Binom FDR Q-Val | Binom Fold Enrichment | Binom Observed Region Hits | Binom Region Set Coverage | Hyper Rank | Hyper FDR Q-Val | Hyper Fold Enrichment | Hyper Observed Gene Hits | Hyper Total Genes | Hyper Gene Set Coverage |
|---|---|---|---|---|---|---|---|---|---|---|---|---|
| prenatal growth retardation | 46 | **1.1013e-64** | **2.1954e-62** | 2.2463 | 547 | 5.28% | 20 | **9.2828e-64** | 1.6896 | 461 | 541 | 4.27% |
| abnormal embryo size | 48 | **3.3977e-64** | **6.4910e-62** | 2.1333 | 607 | 5.86% | 17 | **3.0571e-67** | 1.6664 | 516 | 614 | 4.78% |
| embryonic lethality during organogenesis, complete penetrance | 49 | **1.0243e-61** | **1.9169e-59** | 2.1493 | 574 | 5.54% | 23 | **3.6147e-56** | 1.6276 | 481 | 586 | 4.46% |
| embryonic lethality between implantation and placentation | 51 | **6.2623e-61** | **1.1260e-58** | 2.5153 | 413 | 3.99% | 21 | **7.6007e-61** | 1.7741 | 357 | 399 | 3.31% |

The test set of 10,363 genomic regions picked 10,790 (50%) of all 21,395 genes.
*Mouse Phenotype Single KO* has 9,170 terms covering 9,466 (44%) of all 21,395 genes, and 551,620 term - gene associations.
9,170 ontology terms (100%) were tested using an annotation count range of [1, Inf].

## Mouse Phenotype (20+ terms)

Global controls

*Table controls:* Export Shown top rows in this table: 20 Set Term annotation count: Min: 1 Max: Inf Set Visualize this table: [select one]

| Term Name | Binom Rank | Binom Raw P-Value | Binom FDR Q-Val | Binom Fold Enrichment | Binom Observed Region Hits | Binom Region Set Coverage | Hyper Rank | Hyper FDR Q-Val | Hyper Fold Enrichment | Hyper Observed Gene Hits | Hyper Total Genes | Hyper Gene Set Coverage |
|---|---|---|---|---|---|---|---|---|---|---|---|---|
| preweaning lethality | 1 | **0.0000** | **0.0000** | 2.0374 | 3,410 | 32.91% | 1 | **5.2831e-243** | 1.4628 | 2,987 | 4,049 | 27.68% |
| prenatal lethality | 4 | **4.4271e-291** | **1.0597e-287** | 2.2713 | 2,219 | 21.41% | 3 | **6.8570e-228** | 1.6039 | 1,913 | 2,365 | 17.73% |
| embryonic lethality | 5 | **3.3330e-253** | **6.3827e-250** | 2.4105 | 1,764 | 17.02% | 4 | **2.3486e-215** | 1.6759 | 1,518 | 1,796 | 14.07% |
| embryo phenotype | 9 | **9.0263e-174** | **9.6030e-171** | 2.0945 | 1,638 | 15.81% | 6 | **1.6400e-129** | 1.5346 | 1,390 | 1,796 | 12.88% |
| abnormal embryo morphology | 13 | **7.8477e-143** | **5.7802e-140** | 2.1285 | 1,321 | 12.75% | 10 | **1.4175e-110** | 1.5644 | 1,099 | 1,393 | 10.19% |
| embryonic lethality prior to tooth bud stage | 16 | **7.2705e-134** | **4.3510e-131** | 2.6359 | 830 | 8.01% | 7 | **6.8197e-128** | 1.7777 | 728 | 812 | 6.75% |
| abnormal prenatal growth/weight/body size | 19 | **9.8239e-128** | **4.9507e-125** | 2.1546 | 1,159 | 11.18% | 9 | **1.1021e-113** | 1.6187 | 969 | 1,187 | 8.98% |
| embryonic lethality prior to organogenesis | 20 | **1.3045e-125** | **6.2451e-123** | 2.6905 | 754 | 7.28% | 8 | **6.0252e-119** | 1.7884 | 662 | 734 | 6.14% |
| abnormal embryo development | 22 | **1.6141e-118** | **7.0250e-116** | 2.2310 | 1,004 | 9.69% | 12 | **3.0130e-98** | 1.6194 | 842 | 1,031 | 7.80% |
| abnormal embryonic growth/weight/body size | 25 | **2.8873e-114** | **1.1059e-111** | 2.2008 | 997 | 9.62% | 11 | **3.6862e-107** | 1.6557 | 830 | 994 | 7.69% |
| preweaning lethality, complete penetrance | 31 | **6.6396e-105** | **2.0508e-102** | 2.3988 | 774 | 7.47% | 14 | **2.6805e-93** | 1.6763 | 689 | 815 | 6.39% |
| embryonic lethality during organogenesis | 33 | **2.1034e-103** | **6.1030e-101** | 2.1721 | 931 | 8.98% | 17 | **5.6879e-82** | 1.5917 | 765 | 953 | 7.09% |
| abnormal prenatal body size | 40 | **1.0951e-94** | **2.6214e-92** | 2.1500 | 874 | 8.43% | 16 | **2.8525e-87** | 1.6300 | 730 | 888 | 6.77% |
| abnormal embryonic tissue morphology | 42 | **2.8190e-94** | **6.4266e-92** | 2.0501 | 968 | 9.34% | 23 | **2.2771e-66** | 1.5155 | 788 | 1,031 | 7.30% |
| abnormal cell morphology | 44 | **2.0349e-92** | **4.4283e-90** | 2.0243 | 978 | 9.44% | 31 | **2.9209e-48** | 1.4102 | 852 | 1,198 | 7.90% |
| embryonic lethality during organogenesis, complete penetrance | 50 | **5.8681e-85** | **1.1237e-82** | 2.1898 | 756 | 7.30% | 20 | **2.9784e-71** | 1.6181 | 621 | 761 | 5.76% |
| abnormal embryo size | 52 | **1.1754e-82** | **2.1643e-80** | 2.1869 | 738 | 7.12% | 18 | **6.8221e-82** | 1.6709 | 616 | 731 | 5.71% |
| prenatal growth retardation | 60 | **1.3664e-75** | **2.1805e-73** | 2.2067 | 663 | 6.40% | 21 | **2.2047e-70** | 1.6564 | 553 | 662 | 5.13% |

| Term Name | Binom Rank | Binom Raw P-Value | *Binom FDR Q-Val* | Binom Fold Enrichment | Binom Observed Region Hits | Binom Region Set Coverage | Hyper Rank | *Hyper FDR Q-Val* | Hyper Fold Enrichment | Hyper Observed Gene Hits | Hyper Total Genes | Hyper Gene Set Coverage |
|---|---|---|---|---|---|---|---|---|---|---|---|---|
| **decreased embryo size** | 70 | **9.2257e-69** | **1.2619e-66** | 2.1557 | 635 | 6.13% | 22 | **3.8289e-69** | 1.6688 | 526 | 625 | 4.87% |
| **embryonic growth retardation** | 76 | **1.2484e-65** | **1.5729e-63** | 2.2125 | 573 | 5.53% | 24 | **1.2880e-62** | 1.6687 | 478 | 568 | 4.43% |

The test set of 10,363 genomic regions picked 10,790 (50%) of all 21,395 genes.
*Mouse Phenotype* has 9,575 terms covering 9,654 (45%) of all 21,395 genes, and 705,265 term - gene associations.
9,575 ontology terms (100%) were tested using an annotation count range of [1, Inf].
