# Supplementary material for: AP-2α and AP-2β cooperatively function in the craniofacial surface ectoderm to regulate chromatin and gene expression dynamics during facial development
Source: eLife. 2022 Mar 25;11:e70511. doi: 10.7554/eLife.70511 (PMC9038197; doi:10.7554/eLife.70511)
Supplement: Figure 2—source data 4. [file elife-70511-fig2-data4.pdf]

Job Description

Region-Gene Association Graphs

What do these graphs illustrate?

Number of associated genes per region

Download as PDF.

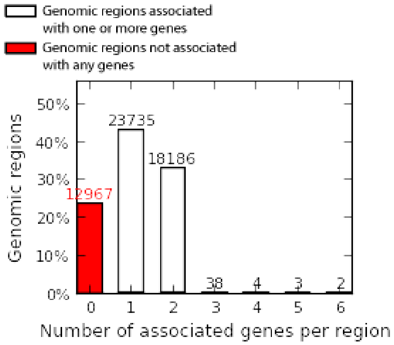

Binned by orientation and distance to TSS

Download as PDF.

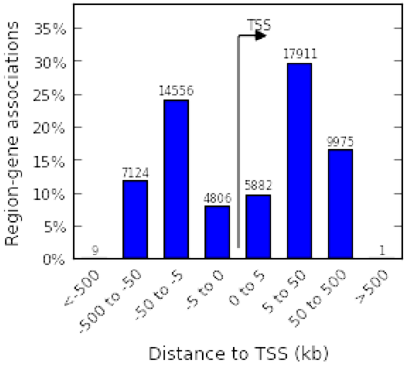

Binned by absolute distance to TSS

Download as PDF.

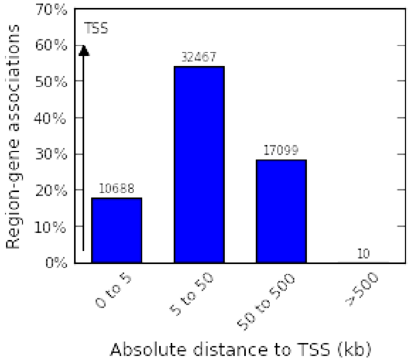

Global Controls

Global Export

Which data is exported by each option?

Ensembl Genes (no terms)

Global controls

GO Biological Process (20+ terms)

Global controls

Table controls: Export Shown top rows in this table: 20 Set Term annotation count: Min: 1 Max: Inf Set Visualize this table: [select one]

Term Name	Binom Rank	Binom Raw P-Value	*Binom FDR Q-Val*	Binom Fold Enrichment	Binom Observed Region Hits	Binom Region Set Coverage	Hyper Rank	*Hyper FDR Q-Val*	Hyper Fold Enrichment	Hyper Observed Gene Hits	Hyper Total Genes	Hyper Gene Set Coverage
**animal organ morphogenesis**	1	**0.0000**	**0.0000**	2.0393	5,190	9.45%	43	**2.1062e-47**	1.2648	874	962	5.69%
**anatomical structure formation involved in morphogenesis**	1	**0.0000**	**0.0000**	2.0189	4,594	8.36%	51	**5.7786e-44**	1.2662	804	884	5.23%
**epithelium development**	1	**0.0000**	**0.0000**	2.0296	5,328	9.70%	72	**4.5403e-39**	1.2344	923	1,041	6.01%
**embryo development**	1	**0.0000**	**0.0000**	2.0091	5,309	9.66%	94	**3.9094e-34**	1.2167	943	1,079	6.14%
**tissue morphogenesis**	1	**0.0000**	**0.0000**	2.1463	3,472	6.32%	96	**8.9745e-34**	1.2800	559	608	3.64%
**embryonic morphogenesis**	1	**0.0000**	**0.0000**	2.1460	3,479	6.33%	98	**2.8486e-33**	1.2745	574	627	3.74%
**tube development**	1	**0.0000**	**0.0000**	2.1484	3,840	6.99%	111	**2.1421e-31**	1.2625	594	655	3.87%
**pattern specification process**	1	**0.0000**	**0.0000**	2.3480	2,622	4.77%	187	**1.4361e-21**	1.2656	400	440	2.60%
**negative regulation of transcription from RNA polymerase II promoter**	1	**0.0000**	**0.0000**	2.0807	4,129	7.52%	242	**1.7075e-17**	1.1790	708	836	4.61%

Term Name	Binom Rank	Binom Raw P-Value	*Binom FDR Q-Val*	Binom Fold Enrichment	Binom Observed Region Hits	Binom Region Set Coverage	Hyper Rank	*Hyper FDR Q-Val*	Hyper Fold Enrichment	Hyper Observed Gene Hits	Hyper Total Genes	Hyper Gene Set Coverage
embryo development ending in birth or egg hatching	166	**6.9169e-323**	**5.4545e-321**	2.0366	3,497	6.37%	274	**8.9678e-16**	1.1826	615	724	4.00%
chordate embryonic development	167	**1.2712e-320**	**9.9643e-319**	2.0376	3,469	6.31%	271	**7.1148e-16**	1.1841	609	716	3.96%
morphogenesis of an epithelium	173	**3.8931e-309**	**2.9457e-307**	2.1689	2,902	5.28%	136	**4.8958e-27**	1.2776	457	498	2.97%
regionalization	177	**5.2691e-293**	**3.8968e-291**	2.4473	2,153	3.92%	206	**3.7245e-20**	1.2870	318	344	2.07%
transcription from RNA polymerase II promoter	178	**2.0853e-292**	**1.5335e-290**	2.1043	2,942	5.36%	171	**1.2477e-23**	1.2450	516	577	3.36%
embryonic organ development	179	**6.4136e-292**	**4.6902e-290**	2.1729	2,733	4.97%	167	**2.8454e-24**	1.2669	445	489	2.90%
tube morphogenesis	184	**2.5089e-288**	**1.7849e-286**	2.2562	2,491	4.53%	176	**4.6166e-23**	1.2864	365	395	2.38%
skeletal system development	191	**5.2037e-266**	**3.5663e-264**	2.1594	2,530	4.61%	165	**1.7510e-24**	1.2747	423	462	2.75%
cell morphogenesis involved in differentiation	196	**3.9151e-260**	**2.6147e-258**	2.0324	2,849	5.19%	95	**4.6168e-34**	1.3073	462	492	3.01%
gland development	204	**2.3350e-251**	**1.4983e-249**	2.0904	2,577	4.69%	211	**7.3255e-20**	1.2506	415	462	2.70%
epithelial cell differentiation	206	**4.3925e-248**	**2.7912e-246**	2.0361	2,708	4.93%	223	**1.6409e-18**	1.2255	478	543	3.11%

The test set of 54,935 genomic regions picked 15,368 (72%) of all 21,395 genes.
GO Biological Process has 13,090 terms covering 17,925 (84%) of all 21,395 genes, and 1,163,819 term - gene associations.
13,090 ontology terms (100%) were tested using an annotation count range of [1, Inf].

GO Cellular Component (19 terms)

Global controls

Table controls: Export | Shown top rows in this table: 20 Set | Term annotation count: Min: 1 Max: Inf Set | Visualize this table: [select one]

Term Name	Binom Rank	Binom Raw P-Value	*Binom FDR Q-Val*	Binom Fold Enrichment	Binom Observed Region Hits	Binom Region Set Coverage	Hyper Rank	*Hyper FDR Q-Val*	Hyper Fold Enrichment	Hyper Observed Gene Hits	Hyper Total Genes	Hyper Gene Set Coverage
cell-cell adherens junction	95	**1.3105e-74**	**2.3367e-73**	2.3108	612	1.11%	121	**2.6806e-6**	1.2927	91	98	0.59%
ruffle membrane	120	**2.5686e-50**	**3.6260e-49**	2.2378	437	0.80%	172	**1.4474e-3**	1.2394	73	82	0.48%
stress fiber	124	**6.0020e-49**	**8.1995e-48**	2.4040	366	0.67%	176	**1.7118e-3**	1.2637	59	65	0.38%
actin filament bundle	126	**7.2958e-49**	**9.8088e-48**	2.3022	399	0.73%	159	**2.6547e-4**	1.2778	67	73	0.44%
actomyosin	138	**1.2346e-45**	**1.5156e-44**	2.2171	403	0.73%	152	**1.3036e-4**	1.2823	70	76	0.46%
transcriptional repressor complex	152	**1.2464e-41**	**1.3891e-40**	2.0476	441	0.80%	218	**2.1950e-2**	1.1842	74	87	0.48%
cortical cytoskeleton	157	**2.0625e-39**	**2.2254e-38**	2.0142	434	0.79%	162	**5.3778e-4**	1.2409	82	92	0.53%
cortical actin cytoskeleton	176	**9.2662e-34**	**8.9187e-33**	2.1411	318	0.58%	199	**6.6614e-3**	1.2399	57	64	0.37%
MHC protein complex	192	**1.8324e-26**	**1.6167e-25**	4.2324	83	0.15%	220	**2.8428e-2**	1.3342	23	24	0.15%
PcG protein complex	210	**2.9334e-23**	**2.3663e-22**	2.1438	213	0.39%	203	**1.0055e-2**	1.2711	42	46	0.27%
dendritic shaft	219	**5.8754e-22**	**4.5447e-21**	2.1174	206	0.37%	166	**7.5573e-4**	1.3546	36	37	0.23%
excitatory synapse	238	**1.0714e-17**	**7.6257e-17**	2.1930	150	0.27%	217	**2.1474e-2**	1.3365	24	25	0.16%

Term Name	Binom Rank	Binom Raw P-Value	*Binom FDR Q-Val*	Binom Fold Enrichment	Binom Observed Region Hits	Binom Region Set Coverage	Hyper Rank	*Hyper FDR Q-Val*	Hyper Fold Enrichment	Hyper Observed Gene Hits	Hyper Total Genes	Hyper Gene Set Coverage
desmosome	239	**1.5025e-17**	**1.0649e-16**	2.2459	141	0.26%	201	**9.5157e-3**	1.3425	27	28	0.18%
compact myelin	266	**1.3256e-14**	**8.4419e-14**	2.4128	99	0.18%	224	**3.7896e-2**	1.3922	16	16	0.10%
costamere	267	**1.4936e-14**	**9.4761e-14**	2.1505	126	0.23%	205	**1.1009e-2**	1.3922	20	20	0.13%
lamellipodium membrane	274	**5.2168e-14**	**3.2253e-13**	2.1481	121	0.22%	222	**3.7757e-2**	1.3316	22	23	0.14%
paranode region of axon	279	**8.4665e-14**	**5.1406e-13**	2.3370	99	0.18%	224	**3.7896e-2**	1.3922	16	16	0.10%
myosin II complex	313	**1.0454e-10**	**5.6577e-10**	2.0349	101	0.18%	236	**4.7527e-2**	1.3289	21	22	0.14%
Golgi medial cisterna	343	**2.2340e-9**	**1.1033e-8**	2.5061	55	0.10%	224	**3.7896e-2**	1.3922	16	16	0.10%

The test set of 54,935 genomic regions picked 15,368 (72%) of all 21,395 genes.
GO Cellular Component has 1,694 terms covering 19,074 (89%) of all 21,395 genes, and 371,380 term - gene associations.
1,694 ontology terms (100%) were tested using an annotation count range of [1, Inf].

GO Molecular Function (20+ terms)

Global controls

Table controls: Export | Shown top rows in this table: 20 Set | Term annotation count: Min: 1 Max: Inf Set | Visualize this table: [select one]

Term Name	Binom Rank	Binom Raw P-Value	*Binom FDR Q-Val*	Binom Fold Enrichment	Binom Observed Region Hits	Binom Region Set Coverage	Hyper Rank	*Hyper FDR Q-Val*	Hyper Fold Enrichment	Hyper Observed Gene Hits	Hyper Total Genes	Hyper Gene Set Coverage
transcription regulatory region sequence-specific DNA binding	1	**0.0000**	**0.0000**	2.1823	3,941	7.17%	4	**9.3706e-35**	1.2625	672	741	4.37%
RNA polymerase II transcription factor activity, sequence-specific DNA binding	1	**0.0000**	**0.0000**	2.1573	4,143	7.54%	5	**1.5525e-34**	1.2577	692	766	4.50%
regulatory region DNA binding	1	**0.0000**	**0.0000**	2.1434	4,611	8.39%	6	**2.1077e-34**	1.2396	796	894	5.18%
transcription regulatory region DNA binding	1	**0.0000**	**0.0000**	2.1427	4,604	8.38%	7	**2.8375e-34**	1.2392	794	892	5.17%
sequence-specific DNA binding	1	**0.0000**	**0.0000**	2.1553	5,398	9.83%	8	**5.0528e-33**	1.2162	933	1,068	6.07%
RNA polymerase II regulatory region sequence-specific DNA binding	1	**0.0000**	**0.0000**	2.2198	3,572	6.50%	9	**9.8904e-33**	1.2691	598	656	3.89%
RNA polymerase II regulatory region DNA binding	1	**0.0000**	**0.0000**	2.2113	3,579	6.51%	10	**1.7536e-32**	1.2675	600	659	3.90%
sequence-specific double-stranded DNA binding	1	**0.0000**	**0.0000**	2.1497	4,025	7.33%	11	**8.2288e-31**	1.2431	692	775	4.50%
double-stranded DNA binding	1	**0.0000**	**0.0000**	2.0995	4,183	7.61%	13	**1.5865e-27**	1.2216	745	849	4.85%
transcription factor activity, RNA polymerase II core promoter proximal region sequence-specific binding	24	**7.0261e-282**	**1.2094e-279**	2.2472	2,457	4.47%	18	**8.9338e-24**	1.2838	391	424	2.54%
transcriptional activator activity, RNA polymerase II transcription regulatory region sequence-specific binding	25	**1.4297e-281**	**2.3625e-279**	2.2513	2,445	4.45%	12	**1.4588e-29**	1.3122	394	418	2.56%

Term Name	Binom Rank	Binom Raw P-Value	*Binom FDR Q-Val*	Binom Fold Enrichment	Binom Observed Region Hits	Binom Region Set Coverage	Hyper Rank	*Hyper FDR Q-Val*	Hyper Fold Enrichment	Hyper Observed Gene Hits	Hyper Total Genes	Hyper Gene Set Coverage
core promoter proximal region DNA binding	27	**6.2579e-255**	**9.5746e-253**	2.2242	2,275	4.14%	32	**1.4474e-18**	1.2591	369	408	2.40%
core promoter proximal region sequence-specific DNA binding	30	**7.9636e-251**	**1.0966e-248**	2.2210	2,246	4.09%	33	**2.8502e-18**	1.2581	366	405	2.38%
RNA polymerase II core promoter proximal region sequence-specific DNA binding	31	**9.8930e-248**	**1.3183e-245**	2.2355	2,188	3.98%	35	**4.0785e-18**	1.2608	355	392	2.31%
transcriptional activator activity, RNA polymerase II core promoter proximal region sequence-specific binding	37	**2.8795e-192**	**3.2149e-190**	2.2491	1,681	3.06%	34	**3.9241e-18**	1.2994	266	285	1.73%
transcriptional repressor activity, RNA polymerase II transcription regulatory region sequence-specific binding	61	**1.9347e-122**	**1.3102e-120**	2.1029	1,245	2.27%	72	**9.3818e-10**	1.2494	210	234	1.37%
transcriptional repressor activity, RNA polymerase II core promoter proximal region sequence-specific binding	71	**7.0170e-96**	**4.0827e-94**	2.2276	854	1.55%	107	**4.8310e-6**	1.2447	135	151	0.88%
enhancer binding	84	**5.3362e-69**	**2.6243e-67**	2.2427	602	1.10%	96	**1.0661e-6**	1.2927	104	112	0.68%
enhancer sequence-specific DNA binding	94	**7.6663e-59**	**3.3691e-57**	2.2042	531	0.97%	104	**3.4657e-6**	1.2957	94	101	0.61%
transcription factor activity, RNA polymerase II distal enhancer sequence-specific binding	102	**1.7629e-55**	**7.1399e-54**	2.0675	580	1.06%	89	**3.2191e-7**	1.3119	98	104	0.64%

The test set of 54,935 genomic regions picked 15,368 (72%) of all 21,395 genes.
GO Molecular Function has 4,131 terms covering 17,189 (80%) of all 21,395 genes, and 227,341 term - gene associations.
4,131 ontology terms (100%) were tested using an annotation count range of [1, Inf].

⊖ Human Phenotype (20+ terms)

Global controls

Table controls: Export Shown top rows in this table: 20 Set Term annotation count: Min: 1 Max: Inf Set Visualize this table: [select one]

Term Name	Binom Rank	Binom Raw P-Value	*Binom FDR Q-Val*	Binom Fold Enrichment	Binom Observed Region Hits	Binom Region Set Coverage	Hyper Rank	*Hyper FDR Q-Val*	Hyper Fold Enrichment	Hyper Observed Gene Hits	Hyper Total Genes	Hyper Gene Set Coverage
Abnormal vascular morphology	55	**5.3009e-123**	**6.3602e-121**	2.0158	1,386	2.52%	46	**5.6904e-6**	1.1852	252	296	1.64%
Oral cleft	60	**2.4385e-118**	**2.6819e-116**	2.0856	1,227	2.23%	160	**2.4463e-3**	1.1532	193	233	1.26%
Abnormality of the nail	64	**1.0185e-112**	**1.0502e-110**	2.2776	959	1.75%	52	**7.9629e-6**	1.2217	172	196	1.12%
Cleft palate	66	**6.1184e-112**	**6.1175e-110**	2.1098	1,129	2.06%	173	**3.3837e-3**	1.1548	180	217	1.17%
Abnormality of the hard palate	68	**4.6439e-110**	**4.5067e-108**	2.0681	1,164	2.12%	152	**2.0881e-3**	1.1591	184	221	1.20%
Abnormality of the periorbital region	69	**1.1720e-109**	**1.1208e-107**	2.0211	1,227	2.23%	93	**2.6769e-4**	1.1715	207	246	1.35%
Abnormal morphology of the great vessels	72	**1.1794e-108**	**1.0810e-106**	2.1375	1,063	1.94%	59	**1.3121e-5**	1.2115	181	208	1.18%
Cleft lip	91	**1.4960e-93**	**1.0848e-91**	2.4528	684	1.25%	350	**3.2943e-2**	1.1812	84	99	0.55%
Abnormality of upper limb joint	92	**1.7149e-93**	**1.2301e-91**	2.0359	1,026	1.87%	138	**1.8211e-3**	1.1623	182	218	1.18%

Term Name	Binom Rank	Binom Raw P-Value	*Binom FDR Q-Val*	Binom Fold Enrichment	Binom Observed Region Hits	Binom Region Set Coverage	Hyper Rank	*Hyper FDR Q-Val*	Hyper Fold Enrichment	Hyper Observed Gene Hits	Hyper Total Genes	Hyper Gene Set Coverage
Cleft upper lip	102	**8.0710e-86**	**5.2216e-84**	2.4203	643	1.17%	301	**2.0478e-2**	1.1976	80	93	0.52%
Aplasia/Hypoplasia of the nails	105	**1.3364e-85**	**8.3990e-84**	2.8127	490	0.89%	115	**8.6456e-4**	1.2808	69	75	0.45%
Abnormality of the stomach	112	**1.5387e-81**	**9.0659e-80**	2.1774	762	1.39%	119	**9.2706e-4**	1.2040	128	148	0.83%
Limb joint contracture	117	**1.1233e-78**	**6.3359e-77**	2.2069	713	1.30%	202	**6.0732e-3**	1.1773	126	149	0.82%
Abnormal systemic arterial morphology	150	**5.2098e-69**	**2.2920e-67**	2.0578	734	1.34%	190	**5.1071e-3**	1.1766	131	155	0.85%
Congenital malformation of the great arteries	154	**3.2310e-68**	**1.3845e-66**	2.0570	726	1.32%	136	**1.7357e-3**	1.1988	124	144	0.81%
Clinodactyly	166	**6.0109e-67**	**2.3895e-65**	2.0147	750	1.37%	262	**1.3949e-2**	1.1649	123	147	0.80%
Aplasia/Hypoplasia involving bones of the thorax	175	**1.1970e-65**	**4.5136e-64**	2.2824	551	1.00%	271	**1.5531e-2**	1.2017	82	95	0.53%
Patent ductus arteriosus	180	**1.1966e-64**	**4.3867e-63**	2.1777	601	1.09%	117	**8.6948e-4**	1.2375	96	108	0.62%
Regional abnormality of skin	186	**7.9654e-62**	**2.8260e-60**	2.0082	697	1.27%	316	**2.3408e-2**	1.1554	122	147	0.79%
Flexion contracture of digit	192	**2.1325e-60**	**7.3294e-59**	2.2161	539	0.98%	254	**1.3234e-2**	1.1992	87	101	0.57%

The test set of 54,935 genomic regions picked 15,368 (72%) of all 21,395 genes.
Human Phenotype has 6,599 terms covering 3,215 (15%) of all 21,395 genes, and 244,972 term - gene associations.
6,599 ontology terms (100%) were tested using an annotation count range of [1, Inf].

⊖ Mouse Phenotype Single KO (20+ terms)

Global controls

Table controls: Export Shown top rows in this table: 20 Set Term annotation count: Min: 1 Max: Inf Set Visualize this table: [select one]

Term Name	Binom Rank	Binom Raw P-Value	*Binom FDR Q-Val*	Binom Fold Enrichment	Binom Observed Region Hits	Binom Region Set Coverage	Hyper Rank	*Hyper FDR Q-Val*	Hyper Fold Enrichment	Hyper Observed Gene Hits	Hyper Total Genes	Hyper Gene Set Coverage
neonatal lethality, complete penetrance	83	**3.9095e-229**	**4.3193e-227**	2	2,618	4.77%	172	**5.3002e-15**	1.2174	418	478	2.72%
abnormal craniofacial development	105	**2.1899e-202**	**1.9125e-200**	2.1813	1,890	3.44%	198	**1.7458e-13**	1.2525	278	309	1.81%
abnormal cartilage morphology	132	**1.1321e-171**	**7.8643e-170**	2.1299	1,694	3.08%	133	**3.7952e-18**	1.2987	264	283	1.72%
abnormal neurocranium morphology	151	**1.2367e-163**	**7.5106e-162**	2.2707	1,403	2.55%	337	**2.4650e-9**	1.2473	198	221	1.29%
perinatal lethality, complete penetrance	157	**1.0899e-158**	**6.3659e-157**	2.2563	1,379	2.51%	377	**1.5142e-8**	1.2361	198	223	1.29%
abnormal eye development	158	**1.2594e-158**	**7.3092e-157**	2.2139	1,436	2.61%	359	**6.3708e-9**	1.2318	215	243	1.40%
abnormal eyelid morphology	175	**1.5027e-144**	**7.8740e-143**	2.3014	1,205	2.19%	341	**2.8320e-9**	1.2656	170	187	1.11%
abnormal autopod morphology	187	**1.6359e-136**	**8.0219e-135**	2.0005	1,568	2.85%	321	**1.4531e-9**	1.2153	268	307	1.74%
abnormal joint morphology	189	**6.6141e-136**	**3.2091e-134**	2.0706	1,434	2.61%	226	**1.8871e-12**	1.2560	249	276	1.62%
abnormal lens morphology	216	**7.1003e-115**	**3.0143e-113**	2.0037	1,314	2.39%	309	**9.6103e-10**	1.2334	233	263	1.52%
abnormal eye size	219	**3.0325e-114**	**1.2698e-112**	2.2321	1,015	1.85%	718	**5.9884e-5**	1.1977	154	179	1.00%
abnormal pectoral girdle bone morphology	225	**9.5613e-112**	**3.8968e-110**	2.1043	1,134	2.06%	303	**8.2954e-10**	1.2625	185	204	1.20%

Term Name	Binom Rank	Binom Raw P-Value	*Binom FDR Q-Val*	Binom Fold Enrichment	Binom Observed Region Hits	Binom Region Set Coverage	Hyper Rank	*Hyper FDR Q-Val*	Hyper Fold Enrichment	Hyper Observed Gene Hits	Hyper Total Genes	Hyper Gene Set Coverage
embryonic lethality during organogenesis, incomplete penetrance	226	**1.5266e-111**	**6.1943e-110**	2.0149	1,258	2.29%	650	**1.9651e-5**	1.1816	202	238	1.31%
microphthalmia	234	**8.3082e-108**	**3.2558e-106**	2.2303	959	1.75%	702	**4.4846e-5**	1.2077	144	166	0.94%
exencephaly	241	**1.7946e-103**	**6.8283e-102**	2.1256	1,025	1.87%	928	**5.4674e-4**	1.1686	162	193	1.05%
abnormal tooth morphology	242	**1.2199e-102**	**4.6225e-101**	2.1111	1,033	1.88%	432	**2.9205e-7**	1.2427	158	177	1.03%
abnormal cornea morphology	247	**7.2808e-101**	**2.7030e-99**	2.2119	913	1.66%	438	**3.7754e-7**	1.2487	148	165	0.96%
abnormal epidermal layer morphology	248	**9.2816e-101**	**3.4320e-99**	2.0386	1,103	2.01%	386	**2.0311e-8**	1.2302	205	232	1.33%
lethality throughout fetal growth and development, incomplete penetrance	253	**7.7269e-100**	**2.8006e-98**	2.1080	1,008	1.83%	784	**1.1026e-4**	1.1911	154	180	1.00%
abnormal renal glomerulus morphology	266	**5.1081e-98**	**1.7609e-96**	2.0123	1,108	2.02%	353	**5.4280e-9**	1.2395	203	228	1.32%

The test set of 54,935 genomic regions picked 15,368 (72%) of all 21,395 genes.
Mouse Phenotype Single KO has 9,170 terms covering 9,466 (44%) of all 21,395 genes, and 551,620 term - gene associations.
9,170 ontology terms (100%) were tested using an annotation count range of [1, Inf].

Mouse Phenotype (20+ terms)

Global controls

Table controls: Export Shown top rows in this table: 20 Set Term annotation count: Min: 1 Max: Inf Set Visualize this table: [select one]

Term Name	Binom Rank	Binom Raw P-Value	*Binom FDR Q-Val*	Binom Fold Enrichment	Binom Observed Region Hits	Binom Region Set Coverage	Hyper Rank	*Hyper FDR Q-Val*	Hyper Fold Enrichment	Hyper Observed Gene Hits	Hyper Total Genes	Hyper Gene Set Coverage
abnormal craniofacial development	104	**3.5940e-262**	**3.3089e-260**	2.1989	2,397	4.36%	198	**2.1192e-18**	1.2604	354	391	2.30%
abnormal eye development	137	**3.1929e-224**	**2.2315e-222**	2.2484	1,959	3.57%	301	**1.6482e-13**	1.2461	290	324	1.89%
abnormal cartilage morphology	151	**1.0504e-214**	**6.6607e-213**	2.0711	2,256	4.11%	120	**8.8151e-26**	1.3003	368	394	2.39%
perinatal lethality, complete penetrance	158	**2.3018e-208**	**1.3949e-206**	2.1959	1,917	3.49%	312	**3.9051e-13**	1.2443	286	320	1.86%
abnormal angiogenesis	167	**1.5490e-196**	**8.8811e-195**	2.0013	2,248	4.09%	137	**1.6146e-23**	1.2838	379	411	2.47%
abnormal autopod morphology	174	**2.4197e-188**	**1.3315e-186**	2.0268	2,089	3.80%	314	**4.1843e-13**	1.2221	345	393	2.24%
abnormal neurocranium morphology	179	**3.2132e-186**	**1.7188e-184**	2.2005	1,707	3.11%	360	**1.2193e-11**	1.2464	248	277	1.61%
abnormal tail morphology	186	**5.3958e-182**	**2.7777e-180**	2.0082	2,066	3.76%	239	**8.5275e-16**	1.2371	367	413	2.39%
abnormal joint morphology	189	**8.5176e-181**	**4.3151e-179**	2.1142	1,814	3.30%	214	**4.5683e-17**	1.2686	308	338	2.00%
abnormal palate morphology	196	**3.2595e-177**	**1.5923e-175**	2.0465	1,921	3.50%	405	**1.0878e-10**	1.2187	288	329	1.87%
abnormal neural tube closure	198	**6.5162e-177**	**3.1511e-175**	2.0368	1,940	3.53%	396	**8.7964e-11**	1.2041	333	385	2.17%
abnormal eyelid morphology	204	**1.5765e-171**	**7.3997e-170**	2.1930	1,585	2.89%	289	**9.6899e-14**	1.2739	237	259	1.54%
abnormal spinal cord morphology	215	**5.9663e-163**	**2.6571e-161**	2.0183	1,829	3.33%	223	**2.6228e-16**	1.2619	310	342	2.02%
abnormal forebrain development	218	**1.3470e-161**	**5.9163e-160**	2.1687	1,531	2.79%	409	**1.3220e-10**	1.2448	228	255	1.48%

Term Name	Binom Rank	Binom Raw P-Value	*Binom FDR Q-Val*	Binom Fold Enrichment	Binom Observed Region Hits	Binom Region Set Coverage	Hyper Rank	*Hyper FDR Q-Val*	Hyper Fold Enrichment	Hyper Observed Gene Hits	Hyper Total Genes	Hyper Gene Set Coverage
abnormal epidermal layer morphology	235	**1.2759e-150**	**5.1988e-149**	2.0175	1,693	3.08%	320	**6.3471e-13**	1.2298	318	360	2.07%
abnormal pectoral girdle bone morphology	244	**7.9106e-148**	**3.1042e-146**	2.0856	1,533	2.79%	313	**4.1338e-13**	1.2633	245	270	1.59%
exencephaly	281	**1.1637e-127**	**3.9652e-126**	2.0543	1,373	2.50%	788	**4.0465e-6**	1.1820	225	265	1.46%
abnormal spinal cord grey matter morphology	283	**5.3482e-127**	**1.8095e-125**	2.0836	1,320	2.40%	316	**4.2156e-13**	1.2796	216	235	1.41%
abnormal brainstem morphology	285	**1.2911e-125**	**4.3378e-124**	2.0493	1,359	2.47%	311	**3.2870e-13**	1.2771	222	242	1.44%
abnormal eye size	286	**1.3919e-125**	**4.6600e-124**	2.1194	1,254	2.28%	643	**2.9082e-7**	1.2166	194	222	1.26%

The test set of 54,935 genomic regions picked 15,368 (72%) of all 21,395 genes.
Mouse Phenotype has 9,575 terms covering 9,654 (45%) of all 21,395 genes, and 705,265 term - gene associations.
9,575 ontology terms (100%) were tested using an annotation count range of [1, Inf].
