# Supplementary material for: AP-2α and AP-2β cooperatively function in the craniofacial surface ectoderm to regulate chromatin and gene expression dynamics during facial development
Source: eLife. 2022 Mar 25;11:e70511. doi: 10.7554/eLife.70511 (PMC9038197; doi:10.7554/eLife.70511)
Supplement: Figure 2—source data 5. [file elife-70511-fig2-data5.pdf]

Homer Known Motif Enrichment Results (/Users/vanottee/output/)

Homer *de novo* Motif Results
Gene Ontology Enrichment Results
Known Motif Enrichment Results (txt file)
Total Target Sequences = 9244, Total Background Sequences = 39886

Rank	Motif	Name	P-value	log P-pvalue	q-value (Benjamini)	# Target Sequences with Motif	% of Targets Sequences with Motif	# Background Sequences with Motif	% of Background Sequences with Motif	Motif File	SVG
1	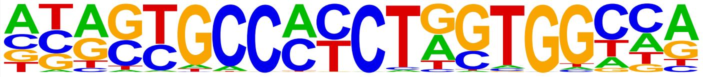	CTCF(Zf)/CD4+-CTCF-ChIP-Seq(Barski_et_al.)/Homer	1e-2372	-5.463e+03	0.0000	3914.0	42.34%	2133.9	5.35%	motif file (matrix)	svg
2	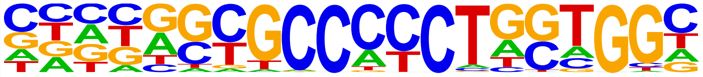	BORIS(Zf)/K562-CTCFL-ChIP-Seq(GSE32465)/Homer	1e-1935	-4.456e+03	0.0000	4311.0	46.64%	3599.7	9.02%	motif file (matrix)	svg
3	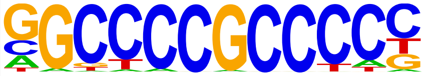	Sp1(Zf)/Promoter/Homer	1e-93	-2.156e+02	0.0000	2017.0	21.82%	5549.3	13.91%	motif file (matrix)	svg
4	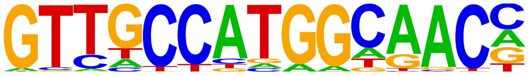	Rfx2(HTH)/LoVo-RFX2-ChIP-Seq(GSE49402)/Homer	1e-85	-1.979e+02	0.0000	674.0	7.29%	1247.8	3.13%	motif file (matrix)	svg
5	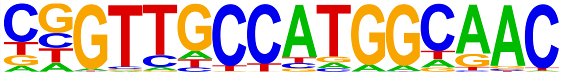	RFX(HTH)/K562-RFX3-ChIP-Seq(SRA012198)/Homer	1e-85	-1.974e+02	0.0000	649.0	7.02%	1179.5	2.96%	motif file (matrix)	svg
6	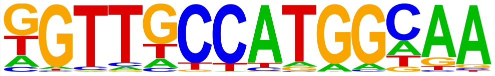	X-box(HTH)/NPC-H3K4me1-ChIP-Seq(GSE16256)/Homer	1e-60	-1.384e+02	0.0000	603.0	6.52%	1248.3	3.13%	motif file (matrix)	svg
7	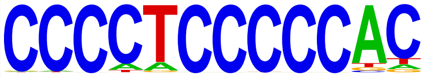	Zfp281(Zf)/ES-Zfp281-ChIP-Seq(GSE81042)/Homer	1e-56	-1.305e+02	0.0000	1602.0	17.33%	4660.4	11.68%	motif file (matrix)	svg
8	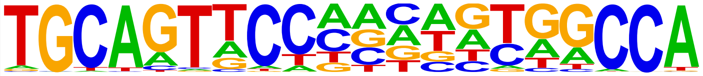	CTCF-SatelliteElement(Zf?)/CD4+-CTCF-ChIP-Seq(Barski_et_al.)/Homer	1e-50	-1.165e+02	0.0000	188.0	2.03%	214.1	0.54%	motif file (matrix)	svg
9	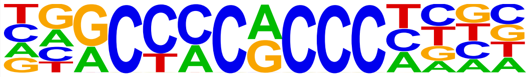	KLF3(Zf)/MEF-Klf3-ChIP-Seq(GSE44748)/Homer	1e-44	-1.035e+02	0.0000	2737.0	29.61%	9265.8	23.22%	motif file (matrix)	svg
10	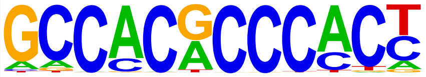	Klf9(Zf)/GBM-Klf9-ChIP-Seq(GSE62211)/Homer	1e-44	-1.019e+02	0.0000	2313.0	25.02%	7614.8	19.08%	motif file (matrix)	svg
11	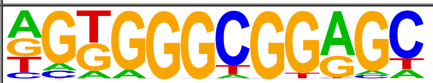	Sp5(Zf)/mES-Sp5.Flag-ChIP-Seq(GSE72989)/Homer	1e-41	-9.551e+01	0.0000	4390.0	47.49%	16165.6	40.51%	motif file (matrix)	svg
12	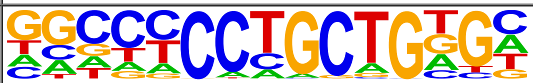	Zic3(Zf)/mES-Zic3-ChIP-Seq(GSE37889)/Homer	1e-34	-7.877e+01	0.0000	2676.0	28.95%	9339.8	23.40%	motif file (matrix)	svg
13	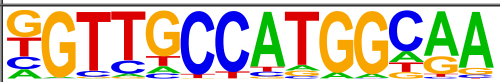	Rfx1(HTH)/NPC-H3K4me1-ChIP-Seq(GSE16256)/Homer	1e-29	-6.745e+01	0.0000	921.0	9.96%	2716.0	6.81%	motif file (matrix)	svg
14	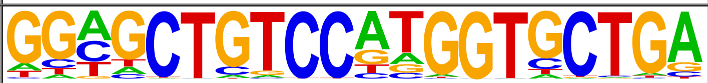	REST-NRSF(Zf)/Jurkat-NRSF-ChIP-Seq/Homer	1e-25	-5.969e+01	0.0000	107.0	1.16%	134.2	0.34%	motif file (matrix)	svg
15		Tcf12(bHLH)/GM12878-Tcf12-ChIP-	1e-	-5.774e+01	0.0000	3588.0	38.81%	13417.2	33.62%	motif	svg

	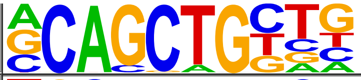	Seq(GSE32465)/Homer	25							file (matrix)	
16	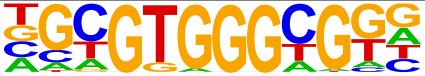	Egr2(Zf)/Thymocytes-Egr2-ChIP-Seq(GSE34254)/Homer	1e-22	-5.253e+01	0.0000	968.0	10.47%	3028.9	7.59%	motif file (matrix)	svg
17	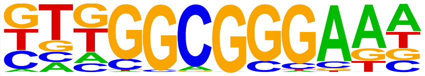	E2F3(E2F)/MEF-E2F3-ChIP-Seq(GSE71376)/Homer	1e-21	-5.063e+01	0.0000	2440.0	26.40%	8818.1	22.10%	motif file (matrix)	svg
18	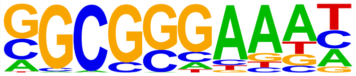	E2F4(E2F)/K562-E2F4-ChIP-Seq(GSE31477)/Homer	1e-21	-4.977e+01	0.0000	1760.0	19.04%	6109.6	15.31%	motif file (matrix)	svg
19	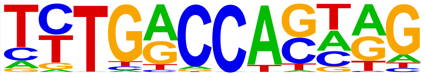	Bcl11a(Zf)/HSPC-BCL11A-ChIP-Seq(GSE104676)/Homer	1e-20	-4.806e+01	0.0000	2378.0	25.72%	8612.0	21.58%	motif file (matrix)	svg
20	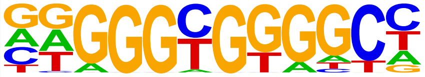	KLF1(Zf)/HUDEP2-KLF1-CutnRun(GSE136251)/Homer	1e-20	-4.636e+01	0.0000	3890.0	42.08%	14910.9	37.37%	motif file (matrix)	svg
21	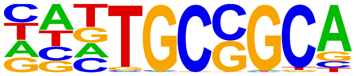	Zfp57(Zf)/H1-ZFP57.HA-ChIP-Seq(GSE115387)/Homer	1e-19	-4.553e+01	0.0000	1551.0	16.78%	5349.3	13.40%	motif file (matrix)	svg
22	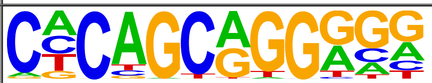	Zic2(Zf)/ESC-Zic2-ChIP-Seq(SRP197560)/Homer	1e-17	-3.981e+01	0.0000	2048.0	22.15%	7423.1	18.60%	motif file (matrix)	svg
23	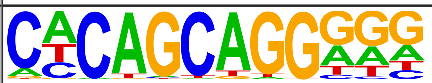	Unknown-ESC-element(?)/mES-Nanog-ChIP-Seq(GSE11724)/Homer	1e-17	-3.923e+01	0.0000	2327.0	25.17%	8566.6	21.47%	motif file (matrix)	svg
24	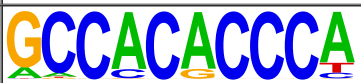	Klf4(Zf)/mES-Klf4-ChIP-Seq(GSE11431)/Homer	1e-15	-3.513e+01	0.0000	1856.0	20.08%	6735.7	16.88%	motif file (matrix)	svg
25	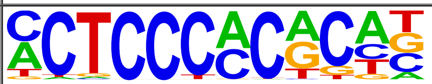	WT1(Zf)/Kidney-WT1-ChIP-Seq(GSE90016)/Homer	1e-15	-3.462e+01	0.0000	2514.0	27.20%	9426.5	23.62%	motif file (matrix)	svg
26	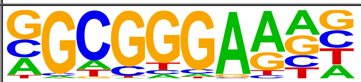	E2F6(E2F)/Hela-E2F6-ChIP-Seq(GSE31477)/Homer	1e-13	-3.126e+01	0.0000	2016.0	21.81%	7459.3	18.69%	motif file (matrix)	svg
27	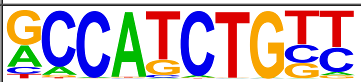	NeuroD1(bHLH)/Islet-NeuroD1-ChIP-Seq(GSE30298)/Homer	1e-13	-3.114e+01	0.0000	2792.0	30.20%	10654.7	26.70%	motif file (matrix)	svg
28	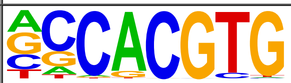	c-Myc(bHLH)/LNCAP-cMyc-ChIP-Seq(Unpublished)/Homer	1e-12	-2.844e+01	0.0000	1591.0	17.21%	5796.3	14.52%	motif file (matrix)	svg
29	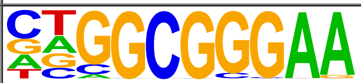	E2F1(E2F)/Hela-E2F1-ChIP-Seq(GSE22478)/Homer	1e-12	-2.802e+01	0.0000	1005.0	10.87%	3478.6	8.72%	motif file (matrix)	svg
30	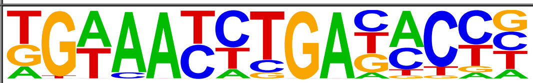	Six4(Homeobox)/MCF7-SIX4-ChIP-Seq(Encode)/Homer	1e-11	-2.706e+01	0.0000	178.0	1.93%	435.8	1.09%	motif file (matrix)	svg
31	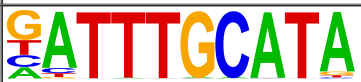	Oct11(POU,Homeobox)/NCIH1048-POU2F3-ChIP-seq(GSE115123)/Homer	1e-11	-2.544e+01	0.0000	625.0	6.76%	2052.1	5.14%	motif file (matrix)	svg
32	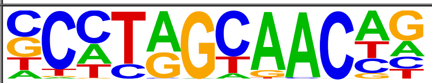	Rfx5(HTH)/GM12878-Rfx5-ChIP-Seq(GSE31477)/Homer	1e-10	-2.492e+01	0.0000	1208.0	13.07%	4331.7	10.85%	motif file (matrix)	svg
33		Myf5(bHLH)/GM-Myf5-ChIP-	1e-9	-2.160e+01	0.0000	2478.0	26.81%	9593.1	24.04%	motif	svg

	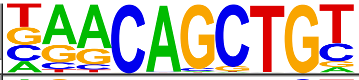	Seq(GSE24852)/Homer								file (matrix)	
34	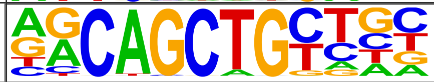	MyoD(bHLH)/Myotube-MyoD-ChIP-Seq(GSE21614)/Homer	1e-9	-2.135e+01	0.0000	2866.0	31.00%	11222.0	28.12%	motif file (matrix)	svg
35	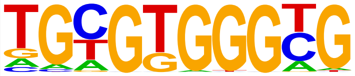	Egr1(Zf)/K562-Egr1-ChIP-Seq(GSE32465)/Homer	1e-8	-1.930e+01	0.0000	2769.0	29.95%	10876.9	27.26%	motif file (matrix)	svg
36	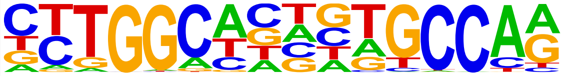	NF1(CTF)/LNCAP-NF1-ChIP-Seq(Unpublished)/Homer	1e-8	-1.883e+01	0.0000	1395.0	15.09%	5209.5	13.05%	motif file (matrix)	svg
37	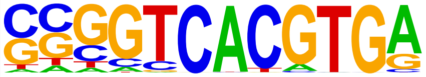	E-box(bHLH)/Promoter/Homer	1e-8	-1.868e+01	0.0000	323.0	3.49%	1004.6	2.52%	motif file (matrix)	svg
38	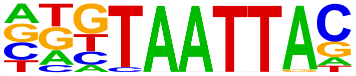	Dlx3(Homeobox)/Kerainocytes-Dlx3-ChIP-Seq(GSE89884)/Homer	1e-7	-1.752e+01	0.0000	1146.0	12.40%	4232.1	10.61%	motif file (matrix)	svg
39	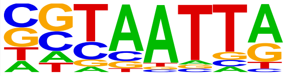	DLX5(Homeobox)/BasalGanglia-Dlx5-ChIP-seq(GSE124936)/Homer	1e-7	-1.745e+01	0.0000	1393.0	15.07%	5234.0	13.12%	motif file (matrix)	svg
40	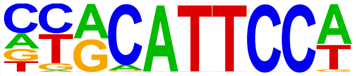	TEAD1(TEAD)/HepG2-TEAD1-ChIP-Seq(Encode)/Homer	1e-7	-1.626e+01	0.0000	2291.0	24.78%	8973.0	22.49%	motif file (matrix)	svg
41	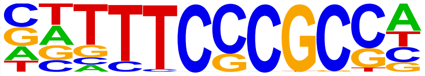	E2F7(E2F)/Hela-E2F7-ChIP-Seq(GSE32673)/Homer	1e-6	-1.596e+01	0.0000	505.0	5.46%	1725.7	4.32%	motif file (matrix)	svg
42	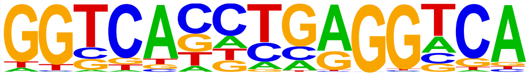	THRb(NR)/HepG2-THRb.Flag-ChIP-Seq(Encode)/Homer	1e-6	-1.557e+01	0.0000	2086.0	22.57%	8140.6	20.40%	motif file (matrix)	svg
43	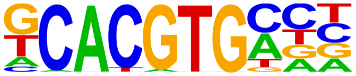	bHLHE40(bHLH)/HepG2-BHLHE40-ChIP-Seq(GSE31477)/Homer	1e-6	-1.556e+01	0.0000	1005.0	10.87%	3708.7	9.29%	motif file (matrix)	svg
44	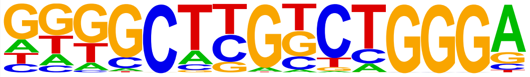	Zfp809(Zf)/ES-Zfp809-ChIP-Seq(GSE70799)/Homer	1e-6	-1.528e+01	0.0000	920.0	9.95%	3373.4	8.45%	motif file (matrix)	svg
45	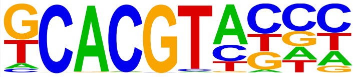	HIF2a(bHLH)/785_O-HIF2a-ChIP-Seq(GSE34871)/Homer	1e-6	-1.416e+01	0.0000	1051.0	11.37%	3926.5	9.84%	motif file (matrix)	svg
46	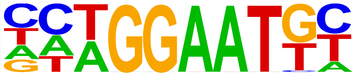	TEAD(TEA)/Fibroblast-PU.1-ChIP-Seq(Unpublished)/Homer	1e-6	-1.387e+01	0.0000	1482.0	16.03%	5693.7	14.27%	motif file (matrix)	svg
47	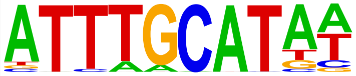	Oct4(POU,Homeobox)/mES-Oct4-ChIP-Seq(GSE11431)/Homer	1e-5	-1.364e+01	0.0000	807.0	8.73%	2956.8	7.41%	motif file (matrix)	svg
48	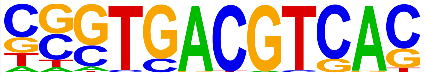	CRE(bZIP)/Promoter/Homer	1e-5	-1.363e+01	0.0000	729.0	7.89%	2645.3	6.63%	motif file (matrix)	svg
49	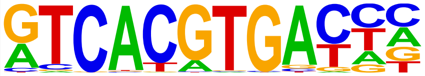	TFE3(bHLH)/MEF-TFE3-ChIP-Seq(GSE75757)/Homer	1e-5	-1.359e+01	0.0000	288.0	3.12%	932.7	2.34%	motif file (matrix)	svg
50	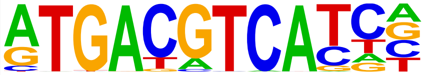	JunD(bZIP)/K562-JunD-ChIP-Seq/Homer	1e-5	-1.298e+01	0.0000	282.0	3.05%	917.8	2.30%	motif file (matrix)	svg
51		NFkB-p50,p52(RHD)/Monocyte-p50-	1e-5	-1.254e+01	0.0000	443.0	4.79%	1539.2	3.86%	motif	svg

	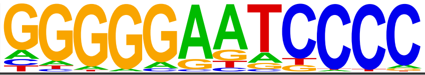	ChIP-Chip(Schreiber_et_al.)/Homer								file (matrix)	
52	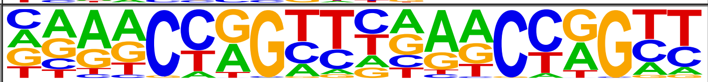	Tcfcp2l1(CP2)/mES-Tcfcp2l1-ChIP-Seq(GSE11431)/Homer	1e-5	-1.197e+01	0.0001	602.0	6.51%	2174.1	5.45%	motif file (matrix)	svg
53	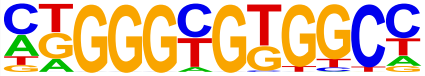	KLF6(Zf)/PDAC-KLF6-ChIP-Seq(GSE64557)/Homer	1e-5	-1.193e+01	0.0001	4009.0	43.37%	16412.3	41.13%	motif file (matrix)	svg
54	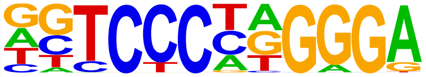	EBF(EBF)/proBcell-EBF-ChIP-Seq(GSE21978)/Homer	1e-5	-1.189e+01	0.0001	829.0	8.97%	3084.6	7.73%	motif file (matrix)	svg
55	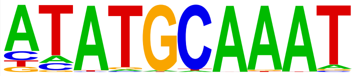	Oct2(POU,Homeobox)/Bcell-Oct2-ChIP-Seq(GSE21512)/Homer	1e-5	-1.163e+01	0.0001	483.0	5.23%	1711.8	4.29%	motif file (matrix)	svg
56	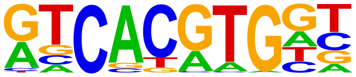	Usf2(bHLH)/C2C12-Usf2-ChIP-Seq(GSE36030)/Homer	1e-4	-1.148e+01	0.0001	900.0	9.74%	3381.4	8.47%	motif file (matrix)	svg
57	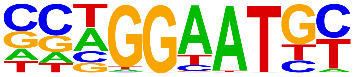	TEAD4(TEA)/Tropoblast-Tead4-ChIP-Seq(GSE37350)/Homer	1e-4	-1.130e+01	0.0001	2145.0	23.20%	8533.2	21.38%	motif file (matrix)	svg
58	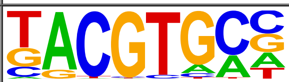	HIF-1a(bHLH)/MCF7-HIF1a-ChIP-Seq(GSE28352)/Homer	1e-4	-1.119e+01	0.0001	776.0	8.39%	2887.5	7.24%	motif file (matrix)	svg
59	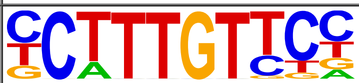	Sox4(HMG)/proB-Sox4-ChIP-Seq(GSE50066)/Homer	1e-4	-1.085e+01	0.0001	2059.0	22.27%	8190.3	20.52%	motif file (matrix)	svg
60	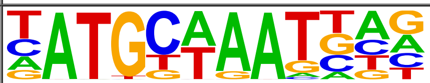	Brn1(POU,Homeobox)/NPC-Brn1-ChIP-Seq(GSE35496)/Homer	1e-4	-1.044e+01	0.0002	583.0	6.31%	2131.0	5.34%	motif file (matrix)	svg
61	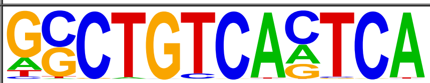	PBX1(Homeobox)/MCF7-PBX1-ChIP-Seq(GSE28007)/Homer	1e-4	-1.040e+01	0.0002	328.0	3.55%	1128.3	2.83%	motif file (matrix)	svg
62	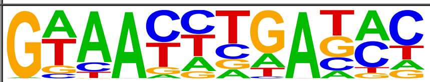	Six2(Homeobox)/NephronProgenitor-Six2-ChIP-Seq(GSE39837)/Homer	1e-4	-1.039e+01	0.0002	2188.0	23.67%	8749.4	21.93%	motif file (matrix)	svg
63	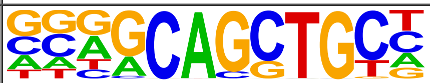	Ascl2(bHLH)/ESC-Ascl2-ChIP-Seq(GSE97712)/Homer	1e-4	-1.035e+01	0.0002	3868.0	41.84%	15881.4	39.80%	motif file (matrix)	svg
64	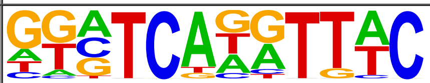	Six1(Homeobox)/Myoblast-Six1-ChIP-Chip(GSE20150)/Homer	1e-4	-1.034e+01	0.0002	611.0	6.61%	2244.8	5.63%	motif file (matrix)	svg
65	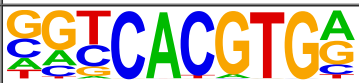	USF1(bHLH)/GM12878-Usf1-ChIP-Seq(GSE32465)/Homer	1e-4	-9.828e+00	0.0004	1357.0	14.68%	5303.1	13.29%	motif file (matrix)	svg
66	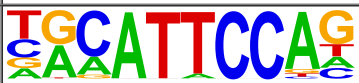	TEAD3(TEA)/HepG2-TEAD3-ChIP-Seq(Encode)/Homer	1e-3	-9.173e+00	0.0007	2558.0	27.67%	10361.7	25.97%	motif file (matrix)	svg
67	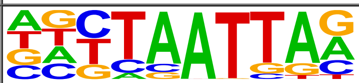	Lhx1(Homeobox)/EmbryoCarcinoma-Lhx1-ChIP-Seq(GSE70957)/Homer	1e-3	-9.000e+00	0.0008	1889.0	20.43%	7551.7	18.92%	motif file (matrix)	svg
68	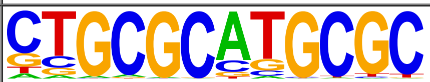	NRF1(NRF)/MCF7-NRF1-ChIP-Seq(Unpublished)/Homer	1e-3	-8.675e+00	0.0011	686.0	7.42%	2586.5	6.48%	motif file (matrix)	svg
69		p73(p53)/Trachea-p73-ChIP-	1e-3	-8.398e+00	0.0014	177.0	1.91%	580.4	1.45%	motif	svg

	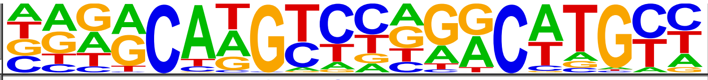	Seq(PRJNA310161)/Homer								file (matrix)	
70	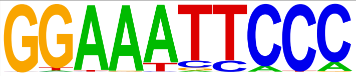	NFkB-p65-Rel(RHD)/ThioMac-LPS-Expression(GSE23622)/Homer	1e-3	-7.790e+00	0.0026	163.0	1.76%	535.7	1.34%	motif file (matrix)	svg
71	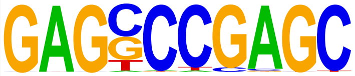	ZNF519(Zf)/HEK293-ZNF519.GFP-ChIP-Seq(GSE58341)/Homer	1e-3	-7.349e+00	0.0040	984.0	10.64%	3846.8	9.64%	motif file (matrix)	svg
72	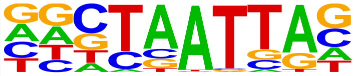	En1(Homeobox)/SUM149-EN1-ChIP-Seq(GSE120957)/Homer	1e-3	-7.085e+00	0.0051	3274.0	35.42%	13512.4	33.86%	motif file (matrix)	svg
73	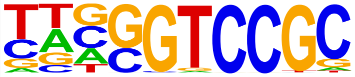	HINFP(Zf)/K562-HINFP.eGFP-ChIP-Seq(Encode)/Homer	1e-2	-6.395e+00	0.0100	1179.0	12.75%	4691.9	11.76%	motif file (matrix)	svg
74	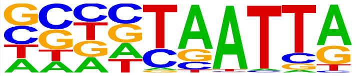	DLX1(Homeobox)/BasalGanglia-Dlx1-ChIP-seq(GSE124936)/Homer	1e-2	-6.198e+00	0.0120	2285.0	24.72%	9354.9	23.44%	motif file (matrix)	svg
75	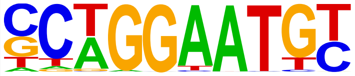	TEAD2(TEA)/Py2T-Tead2-ChIP-Seq(GSE55709)/Homer	1e-2	-5.788e+00	0.0179	1291.0	13.97%	5185.8	13.00%	motif file (matrix)	svg
76	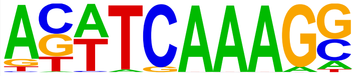	Tcf3(HMG)/mES-Tcf3-ChIP-Seq(GSE11724)/Homer	1e-2	-5.630e+00	0.0207	602.0	6.51%	2331.0	5.84%	motif file (matrix)	svg
77	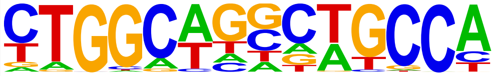	Tlx?(NR)/NPC-H3K4me1-ChIP-Seq(GSE16256)/Homer	1e-2	-5.394e+00	0.0259	1509.0	16.32%	6119.7	15.34%	motif file (matrix)	svg
78	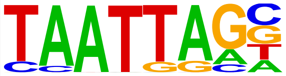	Lhx2(Homeobox)/HFSC-Lhx2-ChIP-Seq(GSE48068)/Homer	1e-2	-5.329e+00	0.0272	1842.0	19.93%	7527.8	18.86%	motif file (matrix)	svg
79	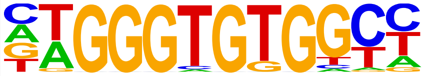	EKLF(Zf)/Erythrocyte-Klf1-ChIP-Seq(GSE20478)/Homer	1e-2	-5.306e+00	0.0275	834.0	9.02%	3300.2	8.27%	motif file (matrix)	svg
80	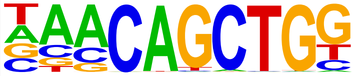	Tcf21(bHLH)/ArterySmoothMuscle-Tcf21-ChIP-Seq(GSE61369)/Homer	1e-2	-5.194e+00	0.0304	3064.0	33.15%	12732.0	31.91%	motif file (matrix)	svg
81	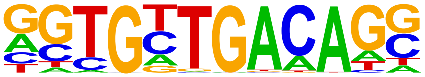	Tbx20(T-box)/Heart-Tbx20-ChIP-Seq(GSE29636)/Homer	1e-2	-5.058e+00	0.0344	644.0	6.97%	2523.8	6.32%	motif file (matrix)	svg
82	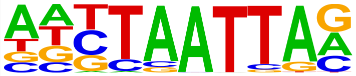	Lhx3(Homeobox)/Neuron-Lhx3-ChIP-Seq(GSE31456)/Homer	1e-2	-5.025e+00	0.0351	2755.0	29.80%	11424.8	28.63%	motif file (matrix)	svg
83	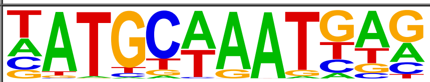	Oct6(POU,Homeobox)/NPC-Pou3f1-ChIP-Seq(GSE35496)/Homer	1e-2	-4.960e+00	0.0370	729.0	7.89%	2878.9	7.21%	motif file (matrix)	svg
84	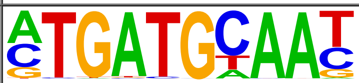	Atf4(bZIP)/MEF-Atf4-ChIP-Seq(GSE35681)/Homer	1e-2	-4.916e+00	0.0382	463.0	5.01%	1784.8	4.47%	motif file (matrix)	svg
85	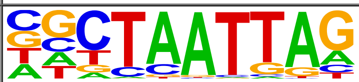	LXH9(Homeobox)/Hct116-LXH9.V5-ChIP-Seq(GSE116822)/Homer	1e-2	-4.832e+00	0.0411	2581.0	27.92%	10695.6	26.80%	motif file (matrix)	svg
86	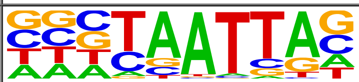	DLX2(Homeobox)/BasalGanglia-Dlx2-ChIP-seq(GSE124936)/Homer	1e-2	-4.795e+00	0.0421	2541.0	27.49%	10527.5	26.38%	motif file (matrix)	svg
87		E2F(E2F)/Hela-CellCycle-	1e-2	-4.720e+00	0.0449	227.0	2.46%	834.2	2.09%	motif	svg

	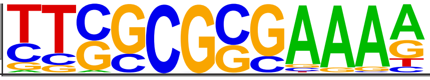	Expression/Homer								file (matrix)	
