# Supplementary material for: AP-2α and AP-2β cooperatively function in the craniofacial surface ectoderm to regulate chromatin and gene expression dynamics during facial development
Source: eLife. 2022 Mar 25;11:e70511. doi: 10.7554/eLife.70511 (PMC9038197; doi:10.7554/eLife.70511)
Supplement: Figure 2—source data 6. [file elife-70511-fig2-data6.pdf]

Job Description

Region-Gene Association Graphs

What do these graphs illustrate?

Number of associated genes per region

Download as PDF.

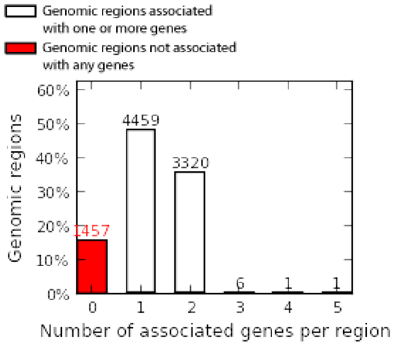

Binned by orientation and distance to TSS

Download as PDF.

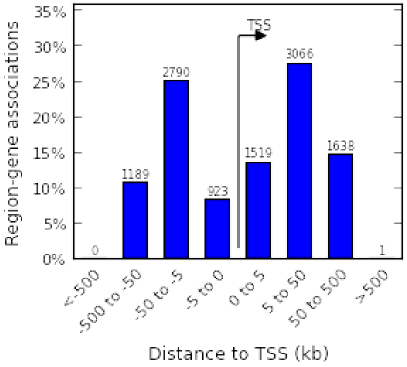

Binned by absolute distance to TSS

Download as PDF.

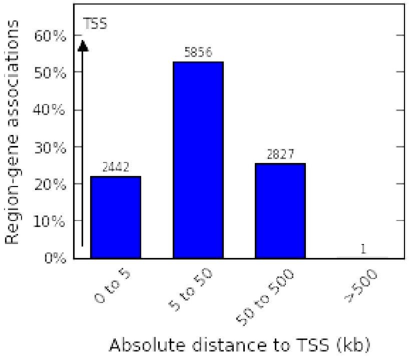

Global Controls

Global Export

Which data is exported by each option?

Ensembl Genes (no terms)

Global controls

GO Biological Process (20+ terms)

Global controls

Table controls: Export Shown top rows in this table: 20 Set Term annotation count: Min: 1 Max: Inf Set Visualize this table: [select one]

Term Name	Binom Rank	Binom Raw P-Value	*Binom FDR Q-Val*	Binom Fold Enrichment	Binom Observed Region Hits	Binom Region Set Coverage	Hyper Rank	*Hyper FDR Q-Val*	Hyper Fold Enrichment	Hyper Observed Gene Hits	Hyper Total Genes	Hyper Gene Set Coverage
anion transport	229	**1.2952e-32**	**7.4037e-31**	2.0061	352	3.81%	160	**4.0300e-8**	1.3834	233	470	3.04%
small molecule biosynthetic process	313	**1.7909e-23**	**7.4898e-22**	2.0334	241	2.61%	394	**2.1154e-4**	1.3193	165	349	2.15%
regulation of myeloid cell differentiation	354	**5.7643e-21**	**2.1315e-19**	2.2096	176	1.90%	262	**1.2219e-5**	1.4829	110	207	1.43%
carboxylic acid transport	365	**3.6501e-20**	**1.3090e-18**	2.1414	181	1.96%	320	**5.9435e-5**	1.4330	114	222	1.49%
organic acid transport	373	**8.7233e-20**	**3.0613e-18**	2.0884	188	2.03%	343	**8.9741e-5**	1.4132	118	233	1.54%
neuromuscular process	398	**2.9900e-18**	**9.8339e-17**	2.3672	131	1.42%	237	**3.1939e-6**	1.6743	72	120	0.94%
plasma membrane organization	416	**1.1338e-17**	**3.5676e-16**	2.0258	179	1.94%	332	**8.3201e-5**	1.4268	113	221	1.47%
developmental maturation	418	**1.4534e-17**	**4.5513e-16**	2.0294	177	1.91%	253	**8.7497e-6**	1.4700	118	224	1.54%
anion transmembrane transport	442	**1.6416e-16**	**4.8615e-15**	2.0515	161	1.74%	295	**3.7937e-5**	1.4548	110	211	1.43%
regulation of innate immune response	443	**2.2220e-16**	**6.5657e-15**	2.0290	164	1.77%	560	**2.1729e-3**	1.3316	115	241	1.50%
monocarboxylic acid transport	466	**2.8150e-15**	**7.9073e-14**	2.4322	102	1.10%	397	**2.1997e-4**	1.5946	60	105	0.78%

Term Name	Binom Rank	Binom Raw P-Value	*Binom FDR Q-Val*	Binom Fold Enrichment	Binom Observed Region Hits	Binom Region Set Coverage	Hyper Rank	*Hyper FDR Q-Val*	Hyper Fold Enrichment	Hyper Observed Gene Hits	Hyper Total Genes	Hyper Gene Set Coverage
regulation of cysteine-type endopeptidase activity involved in apoptotic process	468	**2.8891e-15**	**8.0810e-14**	2.0218	153	1.66%	622	**4.1771e-3**	1.3411	99	206	1.29%
carboxylic acid biosynthetic process	474	**3.3060e-15**	**9.1297e-14**	2.0029	156	1.69%	674	**5.8735e-3**	1.3192	104	220	1.36%
regulation of mitochondrion organization	491	**9.9366e-15**	**2.6491e-13**	2.0809	137	1.48%	988	**3.0452e-2**	1.2733	94	206	1.23%
neurotransmitter transport	492	**1.5164e-14**	**4.0344e-13**	2.2243	116	1.25%	317	**5.5781e-5**	1.5850	71	125	0.93%
monocarboxylic acid biosynthetic process	512	**4.0888e-14**	**1.0454e-12**	2.2166	113	1.22%	701	**6.7699e-3**	1.3953	71	142	0.93%
regulation of purine nucleotide biosynthetic process	527	**6.8593e-14**	**1.7038e-12**	2.1645	117	1.27%	191	**4.0532e-7**	1.6958	79	130	1.03%
regulation of cyclic nucleotide biosynthetic process	528	**6.9966e-14**	**1.7346e-12**	2.1977	113	1.22%	211	**7.1584e-7**	1.7015	75	123	0.98%
regulation of cyclic nucleotide metabolic process	532	**7.8414e-14**	**1.9294e-12**	2.0922	126	1.36%	227	**1.7020e-6**	1.6278	84	144	1.10%
neuromuscular process controlling balance	546	**2.0705e-13**	**4.9639e-12**	2.5961	78	0.84%	361	**1.4241e-4**	1.7758	42	66	0.55%

The test set of 9,244 genomic regions picked 7,667 (36%) of all 21,395 genes.
GO Biological Process has 13,090 terms covering 17,925 (84%) of all 21,395 genes, and 1,163,819 term - gene associations.
13,090 ontology terms (100%) were tested using an annotation count range of [1, Inf].

GO Cellular Component (20+ terms)

Global controls

Table controls: Export | Shown top rows in this table: 20 Set | Term annotation count: Min: 1 Max: Inf Set | Visualize this table: [select one]

Term Name	Binom Rank	Binom Raw P-Value	*Binom FDR Q-Val*	Binom Fold Enrichment	Binom Observed Region Hits	Binom Region Set Coverage	Hyper Rank	*Hyper FDR Q-Val*	Hyper Fold Enrichment	Hyper Observed Gene Hits	Hyper Total Genes	Hyper Gene Set Coverage
microtubule	78	**9.2038e-27**	**1.9989e-25**	2.0243	280	3.03%	78	**1.9137e-4**	1.2956	182	392	2.37%
myelin sheath	87	**3.9348e-21**	**7.6615e-20**	2.3275	159	1.72%	111	**5.1290e-3**	1.3275	98	206	1.28%
late endosome	106	**7.9624e-18**	**1.2725e-16**	2.1610	155	1.68%	69	**9.7930e-5**	1.4213	109	214	1.42%
anchored component of membrane	130	**1.4745e-13**	**1.9215e-12**	2.1155	120	1.30%	89	**7.7627e-4**	1.4297	83	162	1.08%
synaptic vesicle	142	**2.4720e-12**	**2.9490e-11**	2.0585	115	1.24%	125	**1.7660e-2**	1.3402	73	152	0.95%
transport vesicle membrane	149	**1.8462e-11**	**2.0989e-10**	2.0253	110	1.19%	146	**3.6723e-2**	1.3178	68	144	0.89%
melanosome	153	**3.5290e-11**	**3.9072e-10**	2.2346	85	0.92%	109	**3.8660e-3**	1.4742	56	106	0.73%
extrinsic component of cytoplasmic side of plasma membrane	158	**1.2353e-10**	**1.3245e-9**	2.1029	92	1.00%	110	**4.8049e-3**	1.4593	57	109	0.74%
caveola	171	**1.3267e-9**	**1.3143e-8**	2.1201	80	0.87%	62	**2.8465e-5**	1.7356	51	82	0.67%
multivesicular body	173	**1.6339e-9**	**1.5999e-8**	3.1688	38	0.41%	101	**1.3123e-3**	1.9449	23	33	0.30%
ruffle membrane	177	**2.3035e-9**	**2.2046e-8**	2.1911	72	0.78%	118	**1.0271e-2**	1.4974	44	82	0.57%

Term Name	Binom Rank	Binom Raw P-Value	*Binom FDR Q-Val*	Binom Fold Enrichment	Binom Observed Region Hits	Binom Region Set Coverage	Hyper Rank	*Hyper FDR Q-Val*	Hyper Fold Enrichment	Hyper Observed Gene Hits	Hyper Total Genes	Hyper Gene Set Coverage
**presynaptic membrane**	185	**7.5145e-9**	**6.8809e-8**	2.1266	72	0.78%	119	**1.0985e-2**	1.5328	39	71	0.51%
**transcriptional repressor complex**	186	**1.1172e-8**	**1.0175e-7**	2.0694	75	0.81%	114	**5.8112e-3**	1.5075	47	87	0.61%
**cytoplasmic microtubule**	187	**1.2982e-8**	**1.1760e-7**	2.5199	49	0.53%	150	**4.6441e-2**	1.4883	32	60	0.42%
**intrinsic component of synaptic vesicle membrane**	196	**5.4874e-8**	**4.7427e-7**	3.5866	26	0.28%	124	**1.3260e-2**	1.9932	15	21	0.20%
**sperm flagellum**	207	**1.8675e-7**	**1.5283e-6**	2.0078	67	0.72%	148	**4.0983e-2**	1.3812	49	99	0.64%
**cortical actin cytoskeleton**	213	**6.9926e-7**	**5.5613e-6**	2.1207	53	0.57%	147	**3.9482e-2**	1.4825	34	64	0.44%
**integral component of synaptic vesicle membrane**	230	**4.4891e-6**	**3.3063e-5**	3.6485	18	0.19%	128	**1.8289e-2**	2.1926	11	14	0.14%
**nuclear transcriptional repressor complex**	232	**5.3078e-6**	**3.8756e-5**	2.5213	31	0.34%	133	**2.4346e-2**	1.7441	20	32	0.26%
**dendritic shaft**	239	**7.9700e-6**	**5.6491e-5**	2.2601	37	0.40%	143	**3.3915e-2**	1.6592	22	37	0.29%

The test set of 9,244 genomic regions picked 7,667 (36%) of all 21,395 genes.
GO Cellular Component has 1,694 terms covering 19,074 (89%) of all 21,395 genes, and 371,380 term - gene associations.
1,694 ontology terms (100%) were tested using an annotation count range of [1, Inf].

GO Molecular Function (20+ terms)

Global controls

Table controls: Export Shown top rows in this table: 20 Set Term annotation count: Min: 1 Max: Inf Set Visualize this table: [select one]

Term Name	Binom Rank	Binom Raw P-Value	*Binom FDR Q-Val*	Binom Fold Enrichment	Binom Observed Region Hits	Binom Region Set Coverage	Hyper Rank	*Hyper FDR Q-Val*	Hyper Fold Enrichment	Hyper Observed Gene Hits	Hyper Total Genes	Hyper Gene Set Coverage
**core promoter proximal region sequence-specific DNA binding**	52	**4.9084e-37**	**3.8993e-35**	2.1038	358	3.87%	31	**1.4803e-9**	1.4538	211	405	2.75%
**core promoter proximal region DNA binding**	53	**4.3128e-36**	**3.3615e-34**	2.0800	358	3.87%	35	**3.1962e-9**	1.4431	211	408	2.75%
**RNA polymerase II core promoter proximal region sequence-specific DNA binding**	54	**2.1988e-35**	**1.6820e-33**	2.0948	345	3.73%	32	**1.7918e-9**	1.4593	205	392	2.67%
**transcription factor activity, RNA polymerase II core promoter proximal region sequence-specific binding**	55	**9.6292e-35**	**7.2324e-33**	2.0165	371	4.01%	26	**2.7054e-10**	1.4611	222	424	2.90%
**transcriptional activator activity, RNA polymerase II transcription regulatory region sequence-specific binding**	56	**2.3556e-34**	**1.7377e-32**	2.0136	368	3.98%	6	**3.2009e-14**	1.5555	233	418	3.04%
**transcriptional activator activity, RNA polymerase II core promoter proximal region sequence-specific binding**	66	**3.6541e-28**	**2.2871e-26**	2.1150	266	2.88%	28	**5.8326e-10**	1.5568	159	285	2.07%
**symporter activity**	104	**1.2071e-15**	**4.7946e-14**	2.2150	127	1.37%	75	**3.0600e-4**	1.4969	81	151	1.06%
**enhancer binding**	105	**1.3214e-15**	**5.1987e-14**	2.3911	108	1.17%	65	**8.5003e-5**	1.6195	65	112	0.85%

Term Name	Binom Rank	Binom Raw P-Value	Binom FDR Q-Val	Binom Fold Enrichment	Binom Observed Region Hits	Binom Region Set Coverage	Hyper Rank	Hyper FDR Q-Val	Hyper Fold Enrichment	Hyper Observed Gene Hits	Hyper Total Genes	Hyper Gene Set Coverage
enhancer sequence-specific DNA binding	111	**1.3775e-14**	**5.1265e-13**	2.4176	98	1.06%	80	**3.9578e-4**	1.6025	58	101	0.76%
RNA polymerase II distal enhancer sequence-specific DNA binding	113	**3.2125e-14**	**1.1744e-12**	2.6962	78	0.84%	72	**1.9033e-4**	1.7172	48	78	0.63%
lyase activity	115	**2.1315e-13**	**7.6568e-12**	2.0544	127	1.37%	135	**2.5300e-2**	1.3311	83	174	1.08%
channel regulator activity	116	**2.8420e-13**	**1.0121e-11**	2.2204	105	1.14%	53	**2.7978e-5**	1.6203	72	124	0.94%
carboxylic acid transmembrane transporter activity	118	**3.8298e-13**	**1.3408e-11**	2.1904	107	1.16%	104	**2.9760e-3**	1.4734	66	125	0.86%
organic acid transmembrane transporter activity	119	**1.2745e-12**	**4.4242e-11**	2.0887	114	1.23%	112	**6.4481e-3**	1.4258	70	137	0.91%
solute:cation symporter activity	127	**4.0499e-11**	**1.3173e-9**	2.2529	83	0.90%	88	**8.1264e-4**	1.5714	58	103	0.76%
phosphatidylserine binding	129	**6.9842e-11**	**2.2366e-9**	2.7958	54	0.58%	77	**3.0693e-4**	1.9738	29	41	0.38%
monocarboxylic acid transmembrane transporter activity	130	**8.2294e-11**	**2.6151e-9**	2.9970	48	0.52%	157	**4.1274e-2**	1.6743	24	40	0.31%
SNARE binding	131	**8.4224e-11**	**2.6560e-9**	2.1499	89	0.96%	131	**2.3909e-2**	1.4202	57	112	0.74%
ion channel regulator activity	132	**1.1073e-10**	**3.4653e-9**	2.2691	78	0.84%	46	**1.6575e-5**	1.7728	54	85	0.70%
dioxygenase activity	138	**7.1353e-10**	**2.1359e-8**	2.2410	73	0.79%	137	**2.5188e-2**	1.4755	46	87	0.60%

The test set of 9,244 genomic regions picked 7,667 (36%) of all 21,395 genes.
GO Molecular Function has 4,131 terms covering 17,189 (80%) of all 21,395 genes, and 227,341 term - gene associations.
4,131 ontology terms (100%) were tested using an annotation count range of [1, Inf].

Human Phenotype (5 terms)

Global controls

Table controls: Export Shown top rows in this table: 20 Set Term annotation count: Min: 1 Max: Inf Set Visualize this table: [select one]

Term Name	Binom Rank	Binom Raw P-Value	Binom FDR Q-Val	Binom Fold Enrichment	Binom Observed Region Hits	Binom Region Set Coverage	Hyper Rank	Hyper FDR Q-Val	Hyper Fold Enrichment	Hyper Observed Gene Hits	Hyper Total Genes	Hyper Gene Set Coverage
Abnormal larynx morphology	232	**1.8204e-8**	**5.1779e-7**	2.3278	56	0.61%	24	**3.5371e-2**	1.6943	34	56	0.44%
Hypotelorism	276	**1.7403e-7**	**4.1610e-6**	2.3543	47	0.51%	8	**8.7007e-3**	1.9827	27	38	0.35%
Abnormality of the phalanges of the toes	301	**4.3211e-7**	**9.4733e-6**	2.4072	42	0.45%	25	**4.2184e-2**	1.8877	23	34	0.30%
Adrenal overactivity	397	**7.5549e-6**	**1.2558e-4**	2.8703	24	0.26%	17	**3.4348e-2**	2.1563	17	22	0.22%
Abnormality of the gingiva	404	**8.5219e-6**	**1.3920e-4**	2.0307	47	0.51%	31	**4.7144e-2**	1.6743	33	55	0.43%

The test set of 9,244 genomic regions picked 7,667 (36%) of all 21,395 genes.
Human Phenotype has 6,599 terms covering 3,215 (15%) of all 21,395 genes, and 244,972 term - gene associations.
6,599 ontology terms (100%) were tested using an annotation count range of [1, Inf].

Mouse Phenotype Single KO (20+ terms)

Global controls

Table controls: Export Shown top rows in this table: 20 Set Term annotation count: Min: 1 Max: Inf Set Visualize this table: [select one]

Term Name	Binom Rank	Binom Raw P-Value	*Binom FDR Q-Val*	Binom Fold Enrichment	Binom Observed Region Hits	Binom Region Set Coverage	Hyper Rank	*Hyper FDR Q-Val*	Hyper Fold Enrichment	Hyper Observed Gene Hits	Hyper Total Genes	Hyper Gene Set Coverage
abnormal digestive system physiology	107	**2.2104e-34**	**1.8943e-32**	2.0210	365	3.95%	93	**8.9588e-11**	1.4391	246	477	3.21%
abnormal immunoglobulin level	116	**7.9157e-32**	**6.2575e-30**	2.0333	332	3.59%	165	**2.0060e-7**	1.3733	219	445	2.86%
decreased inflammatory response	127	**1.0075e-30**	**7.2743e-29**	2.4935	207	2.24%	150	**3.8586e-8**	1.5412	132	239	1.72%
abnormal acute inflammation	132	**3.8851e-29**	**2.6989e-27**	2.3170	226	2.44%	243	**1.2453e-5**	1.4051	143	284	1.87%
abnormal blood vessel physiology	147	**1.8589e-27**	**1.1596e-25**	2.0497	279	3.02%	68	**1.4713e-12**	1.5776	186	329	2.43%
abnormal circulating triglyceride level	180	**9.2423e-25**	**4.7085e-23**	2.0468	251	2.72%	407	**3.5120e-4**	1.3117	157	334	2.05%
abnormal macrophage physiology	181	**1.0048e-24**	**5.0905e-23**	2.0134	261	2.82%	269	**2.0663e-5**	1.3520	172	355	2.24%
increased or absent threshold for auditory brainstem response	187	**2.5743e-24**	**1.2624e-22**	2.0341	250	2.70%	196	**1.0159e-6**	1.4322	155	302	2.02%
decreased immunoglobulin level	220	**1.0762e-20**	**4.4858e-19**	2.0598	204	2.21%	343	**1.0641e-4**	1.3699	135	275	1.76%
decreased triglyceride level	233	**6.3001e-20**	**2.4795e-18**	2.0723	193	2.09%	528	**1.3380e-3**	1.3247	122	257	1.59%
abnormal posture	241	**1.6437e-19**	**6.2541e-18**	2.0752	188	2.03%	149	**3.7568e-8**	1.5849	117	206	1.53%
abnormal mean corpuscular volume	251	**7.4184e-19**	**2.7102e-17**	2.1600	165	1.78%	1,155	**3.9467e-2**	1.2446	95	213	1.24%
decreased acute inflammation	253	**8.0677e-19**	**2.9241e-17**	2.3984	132	1.43%	306	**5.1056e-5**	1.5079	87	161	1.13%
decreased circulating triglyceride level	282	**1.3822e-17**	**4.4947e-16**	2.1530	154	1.67%	757	**9.8754e-3**	1.3059	95	203	1.24%
abnormal granulocyte physiology	306	**2.5652e-16**	**7.6872e-15**	2.0618	157	1.70%	897	**1.7165e-2**	1.2707	102	224	1.33%
abnormal fat cell morphology	328	**1.4205e-15**	**3.9715e-14**	2.0738	147	1.59%	504	**1.0829e-3**	1.4032	88	175	1.15%
improved glucose tolerance	332	**2.4675e-15**	**6.8153e-14**	2.0002	158	1.71%	446	**5.4012e-4**	1.3953	100	200	1.30%
abnormal hypersensitivity reaction	335	**3.7505e-15**	**1.0266e-13**	2.2631	117	1.27%	636	**3.9223e-3**	1.3863	77	155	1.00%
abnormal intestine physiology	349	**1.6798e-14**	**4.4137e-13**	2.0188	145	1.57%	632	**3.9266e-3**	1.3340	98	205	1.28%
abnormal IgG1 level	354	**2.8096e-14**	**7.2780e-13**	2.0160	143	1.55%	405	**3.4490e-4**	1.4256	94	184	1.23%

The test set of 9,244 genomic regions picked 7,667 (36%) of all 21,395 genes.
Mouse Phenotype Single KO has 9,170 terms covering 9,466 (44%) of all 21,395 genes, and 551,620 term - gene associations.
9,170 ontology terms (100%) were tested using an annotation count range of [1, Inf].

Mouse Phenotype (20+ terms)

Global controls

Table controls: Export Shown top rows in this table: 20 Set Term annotation count: Min: 1 Max: Inf Set Visualize this table: [select one]

Term Name	Binom Rank	Binom Raw P-Value	*Binom FDR Q-Val*	Binom Fold Enrichment	Binom Observed Region Hits	Binom Region Set Coverage	Hyper Rank	*Hyper FDR Q-Val*	Hyper Fold Enrichment	Hyper Observed Gene Hits	Hyper Total Genes	Hyper Gene Set Coverage
abnormal acute inflammation	144	**1.1868e-33**	**7.8912e-32**	2.3075	265	2.87%	317	**5.9274e-6**	1.3787	166	336	2.17%

Term Name	Binom Rank	Binom Raw P-Value	*Binom FDR Q-Val*	Binom Fold Enrichment	Binom Observed Region Hits	Binom Region Set Coverage	Hyper Rank	*Hyper FDR Q-Val*	Hyper Fold Enrichment	Hyper Observed Gene Hits	Hyper Total Genes	Hyper Gene Set Coverage
abnormal blood vessel physiology	153	**6.6757e-33**	**4.1778e-31**	2.0089	354	3.83%	67	**9.7026e-15**	1.5518	233	419	3.04%
decreased inflammatory response	168	**1.5602e-30**	**8.8920e-29**	2.3285	235	2.54%	275	**2.0710e-6**	1.4237	150	294	1.96%
increased or absent threshold for auditory brainstem response	219	**1.3326e-25**	**5.8262e-24**	2.0342	264	2.86%	227	**3.1030e-7**	1.4346	164	319	2.14%
decreased immunoglobulin level	240	**3.0786e-24**	**1.2282e-22**	2.0247	252	2.73%	356	**2.2054e-5**	1.3545	166	342	2.17%
abnormal posture	257	**1.4785e-22**	**5.5082e-21**	2.0023	240	2.60%	196	**9.3041e-8**	1.4856	148	278	1.93%
abnormal granulocyte physiology	288	**2.1786e-20**	**7.2432e-19**	2.1127	189	2.04%	875	**5.9085e-3**	1.2751	122	267	1.59%
decreased acute inflammation	298	**3.1341e-20**	**1.0070e-18**	2.3031	155	1.68%	534	**3.9624e-4**	1.4023	99	197	1.29%
abnormal mean corpuscular volume	307	**2.5567e-19**	**7.9740e-18**	2.0798	185	2.00%	1,162	**1.6868e-2**	1.2529	110	245	1.43%
increased acute inflammation	349	**2.0853e-17**	**5.7211e-16**	2.3706	124	1.34%	1,001	**9.9203e-3**	1.3491	73	151	0.95%
decreased circulating triglyceride level	351	**2.3441e-17**	**6.3944e-16**	2.0375	173	1.87%	996	**9.8828e-3**	1.2737	110	241	1.43%
decreased susceptibility to weight gain	365	**7.5067e-17**	**1.9692e-15**	2.2292	136	1.47%	293	**3.0090e-6**	1.5893	86	151	1.12%
decreased circulating HDL cholesterol level	384	**4.1991e-16**	**1.0470e-14**	2.2161	131	1.42%	647	**1.2563e-3**	1.4226	78	153	1.02%
abnormal hypersensitivity reaction	391	**4.9521e-16**	**1.2127e-14**	2.2189	130	1.41%	844	**5.0132e-3**	1.3477	85	176	1.11%
increased susceptibility to injury	418	**4.7656e-15**	**1.0916e-13**	2.1357	132	1.43%	355	**2.1759e-5**	1.5441	83	150	1.08%
increased energy expenditure	429	**1.5809e-14**	**3.5284e-13**	2.0676	137	1.48%	308	**4.7149e-6**	1.5599	90	161	1.17%
paralysis	431	**1.7537e-14**	**3.8960e-13**	2.1105	130	1.41%	927	**7.7386e-3**	1.3585	74	152	0.97%
decreased susceptibility to diet-induced obesity	456	**5.8854e-14**	**1.2358e-12**	2.2038	113	1.22%	447	**1.0074e-4**	1.5503	70	126	0.91%
abnormal interleukin-6 secretion	464	**1.1591e-13**	**2.3919e-12**	2.0470	131	1.42%	799	**3.6968e-3**	1.3498	89	184	1.16%
cardiomyopathy	479	**1.8721e-13**	**3.7422e-12**	2.0203	133	1.44%	298	**3.3072e-6**	1.5697	90	160	1.17%

The test set of 9,244 genomic regions picked 7,667 (36%) of all 21,395 genes.
Mouse Phenotype has 9,575 terms covering 9,654 (45%) of all 21,395 genes, and 705,265 term - gene associations.
9,575 ontology terms (100%) were tested using an annotation count range of [1, Inf].
