# Supplementary material for: AP-2α and AP-2β cooperatively function in the craniofacial surface ectoderm to regulate chromatin and gene expression dynamics during facial development
Source: eLife. 2022 Mar 25;11:e70511. doi: 10.7554/eLife.70511 (PMC9038197; doi:10.7554/eLife.70511)
Supplement: Figure 2—source data 7. [file elife-70511-fig2-data7.pdf]

Homer Known Motif Enrichment Results (/Users/vanottee/output3/)

[Homer *de novo* Motif Results](#)
[Gene Ontology Enrichment Results](#)
[Known Motif Enrichment Results (txt file)](#)
Total Target Sequences = 20888, Total Background Sequences = 28802

Rank	Motif	Name	P-value	log P-pvalue	q-value (Benjamini)	# Target Sequences with Motif	% of Targets Sequences with Motif	# Background Sequences with Motif	% of Background Sequences with Motif	Motif File	SVG
1	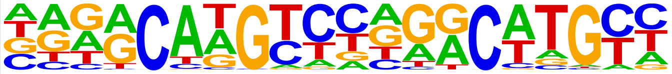	p73(p53)/Trachea-p73-ChIP-Seq(PRJNA310161)/Homer	1e-1476	-3.400e+03	0.0000	2446.0	11.71%	362.9	1.26%	motif file (matrix)	svg
2	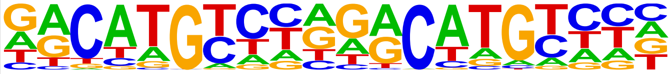	p53(p53)/Saos-p53-ChIP-Seq(GSE15780)/Homer	1e-1391	-3.205e+03	0.0000	3368.0	16.13%	839.3	2.91%	motif file (matrix)	svg
3	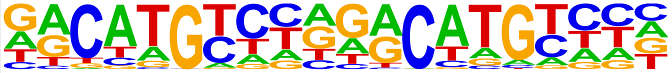	p53(p53)/Saos-p53-ChIP-Seq/Homer	1e-1391	-3.205e+03	0.0000	3368.0	16.13%	839.3	2.91%	motif file (matrix)	svg
4	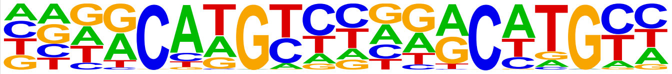	p63(p53)/Keratinocyte-p63-ChIP-Seq(GSE17611)/Homer	1e-1360	-3.132e+03	0.0000	6340.0	30.36%	2994.7	10.40%	motif file (matrix)	svg
5	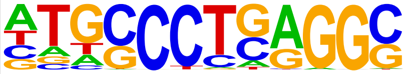	AP-2alpha(AP2)/Hela-AP2alpha-ChIP-Seq(GSE31477)/Homer	1e-627	-1.446e+03	0.0000	7058.0	33.79%	5243.9	18.21%	motif file (matrix)	svg
6	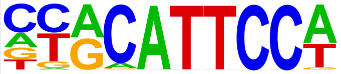	TEAD1(TEAD)/HepG2-TEAD1-ChIP-Seq(Encode)/Homer	1e-554	-1.277e+03	0.0000	8274.0	39.62%	6869.0	23.85%	motif file (matrix)	svg
7	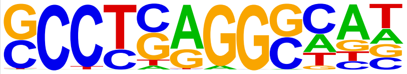	AP-2gamma(AP2)/MCF7-TFAP2C-ChIP-Seq(GSE21234)/Homer	1e-533	-1.229e+03	0.0000	8226.0	39.39%	6889.9	23.93%	motif file (matrix)	svg
8	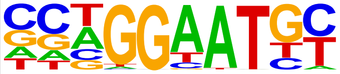	TEAD4(TEA)/Tropoblast-Tead4-ChIP-Seq(GSE37350)/Homer	1e-509	-1.173e+03	0.0000	7561.0	36.20%	6204.9	21.55%	motif file (matrix)	svg
9	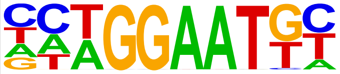	TEAD(TEA)/Fibroblast-PU.1-ChIP-Seq(Unpublished)/Homer	1e-502	-1.156e+03	0.0000	6291.0	30.12%	4801.1	16.67%	motif file (matrix)	svg
10	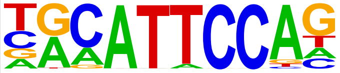	TEAD3(TEA)/HepG2-TEAD3-ChIP-Seq(Encode)/Homer	1e-482	-1.111e+03	0.0000	9003.0	43.11%	8026.1	27.87%	motif file (matrix)	svg
11	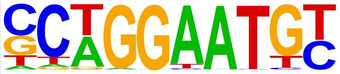	TEAD2(TEA)/Py2T-Tead2-ChIP-Seq(GSE55709)/Homer	1e-412	-9.498e+02	0.0000	5240.0	25.09%	3960.6	13.75%	motif file (matrix)	svg
12	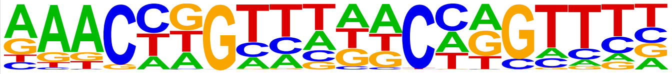	GRHL2(CP2)/HBE-GRHL2-ChIP-Seq(GSE46194)/Homer	1e-355	-8.175e+02	0.0000	4114.0	19.70%	2963.8	10.29%	motif file (matrix)	svg
13	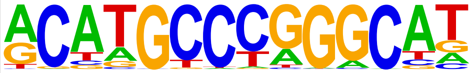	p53(p53)/mES-cMyc-ChIP-Seq(GSE11431)/Homer	1e-229	-5.286e+02	0.0000	559.0	2.68%	133.7	0.46%	motif file (matrix)	svg
14	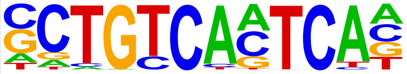	Pbx3(Homeobox)/GM12878-PBX3-ChIP-Seq(GSE32465)/Homer	1e-109	-2.519e+02	0.0000	2306.0	11.04%	1967.3	6.83%	motif file (matrix)	svg
15	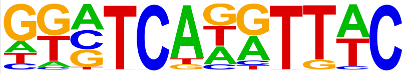	Six1(Homeobox)/Myoblast-Six1-ChIP-Chip(GSE20150)/Homer	1e-108	-2.489e+02	0.0000	2129.0	10.19%	1780.1	6.18%	motif file (matrix)	svg

16	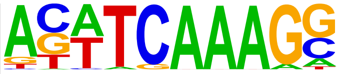	Tcf3(HMG)/mES-Tcf3-ChIP-Seq(GSE11724)/Homer	1e-90	-2.075e+02	0.0000	2327.0	11.14%	2089.2	7.26%	motif file (matrix)	svg
17	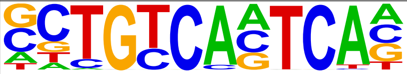	Pknox1(Homeobox)/ES-Prep1-ChIP-Seq(GSE63282)/Homer	1e-88	-2.036e+02	0.0000	2117.0	10.14%	1863.6	6.47%	motif file (matrix)	svg
18	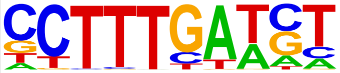	LEF1(HMG)/H1-LEF1-ChIP-Seq(GSE64758)/Homer	1e-74	-1.718e+02	0.0000	4900.0	23.46%	5294.1	18.39%	motif file (matrix)	svg
19	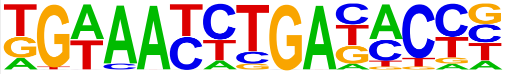	Six4(Homeobox)/MCF7-SIX4-ChIP-Seq(Encode)/Homer	1e-73	-1.684e+02	0.0000	568.0	2.72%	330.9	1.15%	motif file (matrix)	svg
20	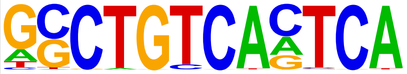	PBX1(Homeobox)/MCF7-PBX1-ChIP-Seq(GSE28007)/Homer	1e-72	-1.665e+02	0.0000	963.0	4.61%	705.0	2.45%	motif file (matrix)	svg
21	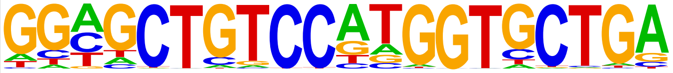	REST-NRSF(Zf)/Jurkat-NRSF-ChIP-Seq/Homer	1e-70	-1.627e+02	0.0000	185.0	0.89%	48.1	0.17%	motif file (matrix)	svg
22	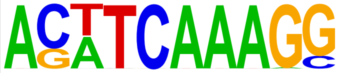	TCFL2(HMG)/K562-TCF7L2-ChIP-Seq(GSE29196)/Homer	1e-62	-1.439e+02	0.0000	890.0	4.26%	667.1	2.32%	motif file (matrix)	svg
23	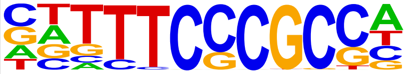	E2F7(E2F)/Hela-E2F7-ChIP-Seq(GSE32673)/Homer	1e-57	-1.322e+02	0.0000	593.0	2.84%	393.0	1.36%	motif file (matrix)	svg
24	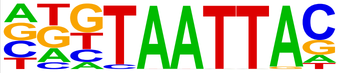	Dlx3(Homeobox)/Kerainocytes-Dlx3-ChIP-Seq(GSE89884)/Homer	1e-53	-1.236e+02	0.0000	4028.0	19.29%	4405.4	15.30%	motif file (matrix)	svg
25	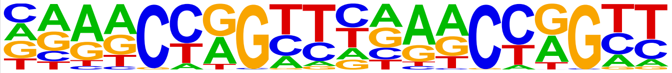	Tcfcp2l1(CP2)/mES-Tcfcp2l1-ChIP-Seq(GSE11431)/Homer	1e-53	-1.231e+02	0.0000	1351.0	6.47%	1199.3	4.16%	motif file (matrix)	svg
26	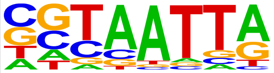	DLX5(Homeobox)/BasalGanglia-Dlx5-ChIP-seq(GSE124936)/Homer	1e-50	-1.167e+02	0.0000	4669.0	22.35%	5248.2	18.23%	motif file (matrix)	svg
27	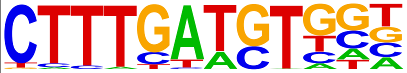	Tcf7(HMG)/GM12878-TCF7-ChIP-Seq(Encode)/Homer	1e-38	-8.753e+01	0.0000	2540.0	12.16%	2716.7	9.43%	motif file (matrix)	svg
28	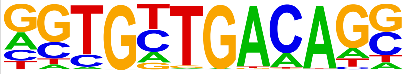	Tbx20(T-box)/Heart-Tbx20-ChIP-Seq(GSE29636)/Homer	1e-35	-8.281e+01	0.0000	1712.0	8.20%	1732.3	6.02%	motif file (matrix)	svg
29	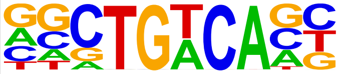	Meis1(Homeobox)/MastCells-Meis1-ChIP-Seq(GSE48085)/Homer	1e-35	-8.084e+01	0.0000	10259.0	49.12%	12907.5	44.83%	motif file (matrix)	svg
30	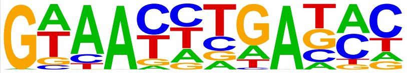	Six2(Homeobox)/NephronProgenitor-Six2-ChIP-Seq(GSE39837)/Homer	1e-26	-6.084e+01	0.0000	5890.0	28.20%	7182.0	24.94%	motif file (matrix)	svg
31	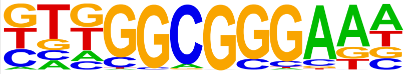	E2F3(E2F)/MEF-E2F3-ChIP-Seq(GSE71376)/Homer	1e-22	-5.279e+01	0.0000	2492.0	11.93%	2827.9	9.82%	motif file (matrix)	svg
32	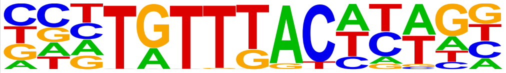	Foxa3(Forkhead)/Liver-Foxa3-ChIP-Seq(GSE77670)/Homer	1e-19	-4.376e+01	0.0000	2148.0	10.28%	2445.0	8.49%	motif file (matrix)	svg
33	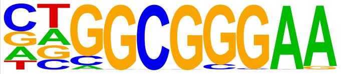	E2F1(E2F)/Hela-E2F1-ChIP-Seq(GSE22478)/Homer	1e-18	-4.324e+01	0.0000	809.0	3.87%	804.2	2.79%	motif file (matrix)	svg
34	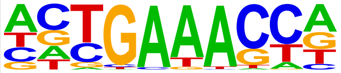	IRF4(IRF)/GM12878-IRF4-ChIP-Seq(GSE32465)/Homer	1e-16	-3.755e+01	0.0000	2654.0	12.71%	3131.1	10.87%	motif file (matrix)	svg

35	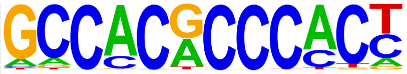	Klf9(Zf)/GBM-Klf9-ChIP-Seq(GSE62211)/Homer	1e-16	-3.730e+01	0.0000	2401.0	11.50%	2808.9	9.75%	motif file (matrix)	svg
36	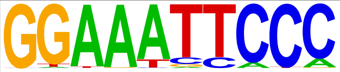	NFkB-p65-Rel(RHD)/ThioMac-LPS-Expression(GSE23622)/Homer	1e-15	-3.519e+01	0.0000	394.0	1.89%	353.1	1.23%	motif file (matrix)	svg
37	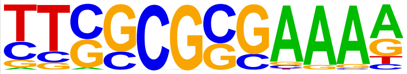	E2F(E2F)/Hela-CellCycle-Expression/Homer	1e-14	-3.425e+01	0.0000	216.0	1.03%	165.9	0.58%	motif file (matrix)	svg
38	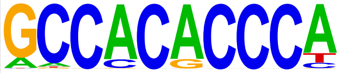	Klf4(Zf)/mES-Klf4-ChIP-Seq(GSE11431)/Homer	1e-14	-3.402e+01	0.0000	2383.0	11.41%	2808.6	9.75%	motif file (matrix)	svg
39	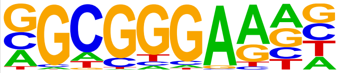	E2F6(E2F)/Hela-E2F6-ChIP-Seq(GSE31477)/Homer	1e-14	-3.349e+01	0.0000	2027.0	9.71%	2356.5	8.18%	motif file (matrix)	svg
40	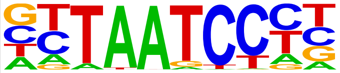	Otx2(Homeobox)/EpiLC-Otx2-ChIP-Seq(GSE56098)/Homer	1e-13	-3.158e+01	0.0000	5337.0	25.55%	6713.4	23.31%	motif file (matrix)	svg
41	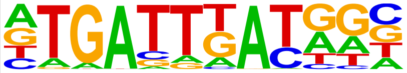	PBX2(Homeobox)/K562-PBX2-ChIP-Seq(Encode)/Homer	1e-13	-3.053e+01	0.0000	4669.0	22.35%	5834.9	20.26%	motif file (matrix)	svg
42	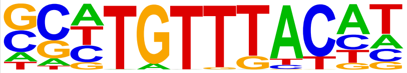	FOXK2(Forkhead)/U2OS-FOXK2-ChIP-Seq(E-MTAB-2204)/Homer	1e-11	-2.537e+01	0.0000	3677.0	17.61%	4573.7	15.88%	motif file (matrix)	svg
43	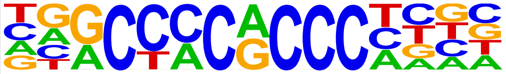	KLF3(Zf)/MEF-Klf3-ChIP-Seq(GSE44748)/Homer	1e-10	-2.445e+01	0.0000	2752.0	13.18%	3365.2	11.69%	motif file (matrix)	svg
44	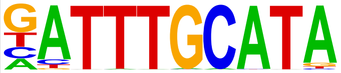	Oct11(POU,Homeobox)/NCIH1048-POU2F3-ChIP-seq(GSE115123)/Homer	1e-10	-2.340e+01	0.0000	1797.0	8.60%	2134.8	7.41%	motif file (matrix)	svg
45	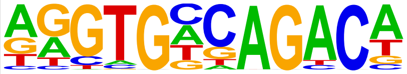	Tbox:Smad(T-box,MAD)/ESCd5-Smad2_3-ChIP-Seq(GSE29422)/Homer	1e-10	-2.312e+01	0.0000	1333.0	6.38%	1543.7	5.36%	motif file (matrix)	svg
46	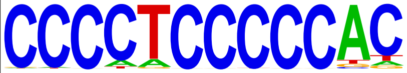	Zfp281(Zf)/ES-Zfp281-ChIP-Seq(GSE81042)/Homer	1e-8	-2.062e+01	0.0000	1578.0	7.56%	1874.3	6.51%	motif file (matrix)	svg
47	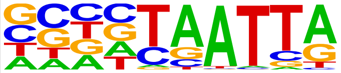	DLX1(Homeobox)/BasalGanglia-Dlx1-ChIP-seq(GSE124936)/Homer	1e-8	-1.861e+01	0.0000	7045.0	33.73%	9185.7	31.90%	motif file (matrix)	svg
48	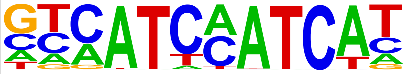	HOXA2(Homeobox)/mES-Hoxa2-ChIP-Seq(Donaldson_et_al.)/Homer	1e-8	-1.847e+01	0.0000	647.0	3.10%	711.9	2.47%	motif file (matrix)	svg
49	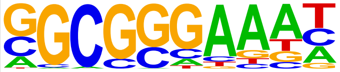	E2F4(E2F)/K562-E2F4-ChIP-Seq(GSE31477)/Homer	1e-7	-1.778e+01	0.0000	1363.0	6.53%	1620.7	5.63%	motif file (matrix)	svg
50	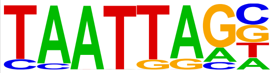	Lhx2(Homeobox)/HFSC-Lhx2-ChIP-Seq(GSE48068)/Homer	1e-7	-1.762e+01	0.0000	5887.0	28.19%	7631.9	26.50%	motif file (matrix)	svg
51	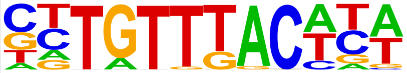	Foxa2(Forkhead)/Liver-Foxa2-ChIP-Seq(GSE25694)/Homer	1e-7	-1.666e+01	0.0000	4705.0	22.53%	6052.7	21.02%	motif file (matrix)	svg
52	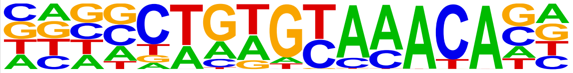	Fox:Ebox(Forkhead,bHLH)/Panc1-Foxa2-ChIP-Seq(GSE47459)/Homer	1e-6	-1.445e+01	0.0000	6314.0	30.23%	8262.4	28.69%	motif file (matrix)	svg
53	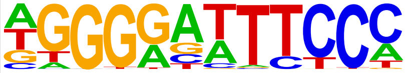	NFkB-p65(RHD)/GM12787-p65-ChIP-Seq(GSE19485)/Homer	1e-6	-1.407e+01	0.0000	2683.0	12.85%	3386.8	11.76%	motif file (matrix)	svg

54	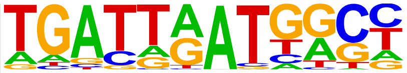	Hoxb4(Homeobox)/ES-Hoxb4-ChIP-Seq(GSE34014)/Homer	1e-6	-1.401e+01	0.0000	1162.0	5.56%	1392.1	4.83%	motif file (matrix)	svg
55	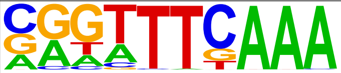	CHR(?)/Hela-CellCycle-Expression/Homer	1e-5	-1.347e+01	0.0000	3319.0	15.89%	4241.6	14.73%	motif file (matrix)	svg
56	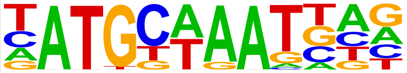	Brn1(POU,Homeobox)/NPC-Brn1-ChIP-Seq(GSE35496)/Homer	1e-5	-1.321e+01	0.0000	1738.0	8.32%	2149.8	7.47%	motif file (matrix)	svg
57	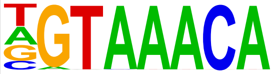	Foxo3(Forkhead)/U2OS-Foxo3-ChIP-Seq(E-MTAB-2701)/Homer	1e-5	-1.319e+01	0.0000	4150.0	19.87%	5359.3	18.61%	motif file (matrix)	svg
58	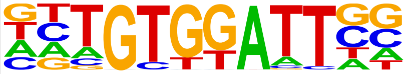	Foxh1(Forkhead)/hESC-FOXH1-ChIP-Seq(GSE29422)/Homer	1e-4	-1.085e+01	0.0001	3375.0	16.16%	4356.3	15.13%	motif file (matrix)	svg
59	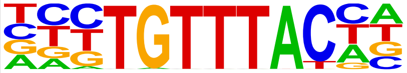	FOXP1(Forkhead)/H9-FOXP1-ChIP-Seq(GSE31006)/Homer	1e-4	-1.061e+01	0.0002	2511.0	12.02%	3204.4	11.13%	motif file (matrix)	svg
60	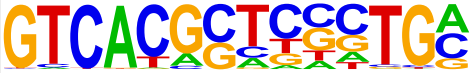	PAX5(Paired,Homeobox),condensed/GM12878-PAX5-ChIP-Seq(GSE32465)/Homer	1e-4	-1.013e+01	0.0003	665.0	3.18%	785.4	2.73%	motif file (matrix)	svg
61	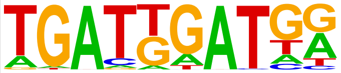	HOXA1(Homeobox)/mES-Hoxa1-ChIP-Seq(SRP084292)/Homer	1e-3	-9.067e+00	0.0008	1455.0	6.97%	1824.6	6.34%	motif file (matrix)	svg
62	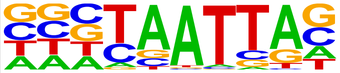	DLX2(Homeobox)/BasalGanglia-Dlx2-ChIP-seq(GSE124936)/Homer	1e-3	-8.361e+00	0.0017	7746.0	37.09%	10343.7	35.92%	motif file (matrix)	svg
63	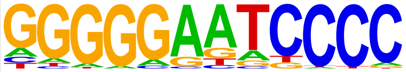	NFkB-p50,p52(RHD)/Monocyte-p50-ChIP-Chip(Schreiber_et_al.)/Homer	1e-3	-7.475e+00	0.0039	569.0	2.72%	683.0	2.37%	motif file (matrix)	svg
64	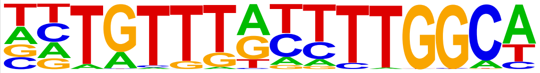	NF1:FOXA1(CTF,Forkhead)/LNCAP-FOXA1-ChIP-Seq(GSE27824)/Homer	1e-3	-7.472e+00	0.0039	317.0	1.52%	362.8	1.26%	motif file (matrix)	svg
65	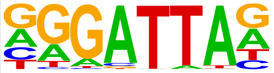	GSC(Homeobox)/FrogEmbryos-GSC-ChIP-Seq(DRA000576)/Homer	1e-3	-6.994e+00	0.0062	6983.0	33.43%	9335.6	32.42%	motif file (matrix)	svg
66	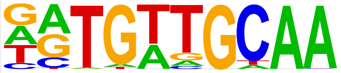	CEBP:AP1(bZIP)/ThioMac-CEBPb-ChIP-Seq(GSE21512)/Homer	1e-3	-6.959e+00	0.0063	3813.0	18.26%	5020.1	17.43%	motif file (matrix)	svg
67	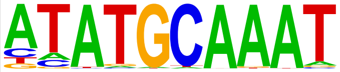	Oct2(POU,Homeobox)/Bcell-Oct2-ChIP-Seq(GSE21512)/Homer	1e-2	-6.588e+00	0.0090	1491.0	7.14%	1906.0	6.62%	motif file (matrix)	svg
68	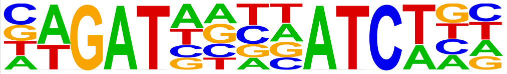	GATA(Zf),IR4/iTreg-Gata3-ChIP-Seq(GSE20898)/Homer	1e-2	-6.530e+00	0.0094	451.0	2.16%	539.5	1.87%	motif file (matrix)	svg
69	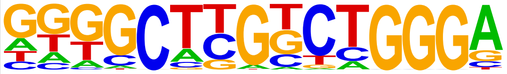	Zfp809(Zf)/ES-Zfp809-ChIP-Seq(GSE70799)/Homer	1e-2	-6.291e+00	0.0118	1015.0	4.86%	1278.4	4.44%	motif file (matrix)	svg
70	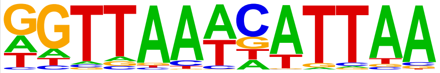	Hnf1(Homeobox)/Liver-Foxa2-Chip-Seq(GSE25694)/Homer	1e-2	-6.226e+00	0.0124	645.0	3.09%	793.9	2.76%	motif file (matrix)	svg
71	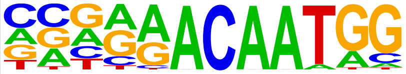	Sox7(HMG)/ESC-Sox7-ChIP-Seq(GSE133899)/Homer	1e-2	-5.485e+00	0.0256	1691.0	8.10%	2190.4	7.61%	motif file (matrix)	svg
72	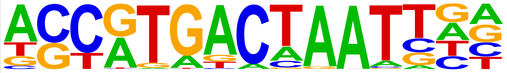	PAX3:FKHR-fusion(Paired,Homeobox)/Rh4-PAX3:FKHR-ChIP-Seq(GSE19063)/Homer	1e-2	-5.440e+00	0.0264	1200.0	5.75%	1535.0	5.33%	motif file (matrix)	svg

73	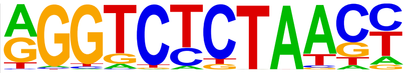	PRDM14(Zf)/H1-PRDM14-ChIP-Seq(GSE22767)/Homer	1e-2	-5.420e+00	0.0266	1983.0	9.49%	2583.0	8.97%	motif file (matrix)	svg
74	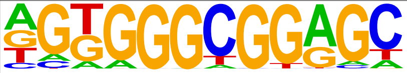	Sp5(Zf)/mES-Sp5.Flag-ChIP-Seq(GSE72989)/Homer	1e-2	-5.304e+00	0.0294	4658.0	22.30%	6209.4	21.56%	motif file (matrix)	svg
75	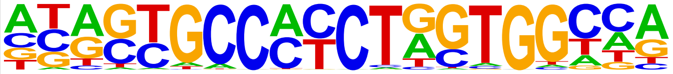	CTCF(Zf)/CD4+-CTCF-ChIP-Seq(Barski_et_al.)/Homer	1e-2	-5.249e+00	0.0307	886.0	4.24%	1121.9	3.90%	motif file (matrix)	svg
76	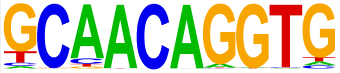	SCRT1(Zf)/HEK293-SCRT1.eGFP-ChIP-Seq(Encode)/Homer	1e-2	-4.862e+00	0.0446	1838.0	8.80%	2400.0	8.33%	motif file (matrix)	svg
