# Supplementary material for: AP-2α and AP-2β cooperatively function in the craniofacial surface ectoderm to regulate chromatin and gene expression dynamics during facial development
Source: eLife. 2022 Mar 25;11:e70511. doi: 10.7554/eLife.70511 (PMC9038197; doi:10.7554/eLife.70511)
Supplement: Figure 2—source data 8. [file elife-70511-fig2-data8.pdf]

Job Description

Region-Gene Association Graphs

What do these graphs illustrate?

Number of associated genes per region

Download as PDF.

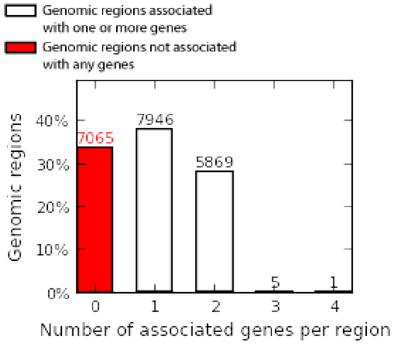

Binned by orientation and distance to TSS

Download as PDF.

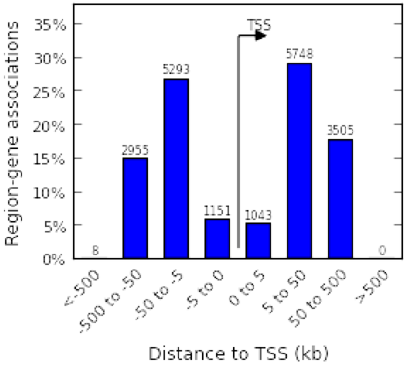

Binned by absolute distance to TSS

Download as PDF.

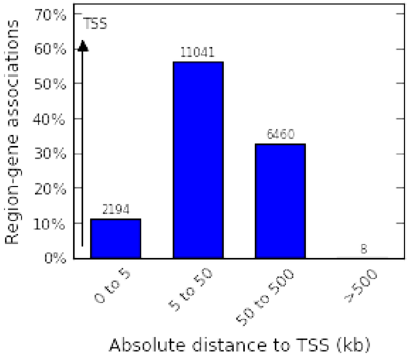

Global Controls

Global Export

Which data is exported by each option?

Ensembl Genes (no terms)

Global controls

GO Biological Process (20+ terms)

Global controls

Table controls: Export Shown top rows in this table: 20 Set Term annotation count: Min: 1 Max: Inf Set Visualize this table: [select one]

Term Name	Binom Rank	Binom Raw P-Value	*Binom FDR Q-Val*	Binom Fold Enrichment	Binom Observed Region Hits	Binom Region Set Coverage	Hyper Rank	*Hyper FDR Q-Val*	Hyper Fold Enrichment	Hyper Observed Gene Hits	Hyper Total Genes	Hyper Gene Set Coverage
skin development	117	**3.5355e-51**	**3.9555e-49**	2.2042	456	2.18%	245	**2.6325e-10**	1.4902	162	246	1.71%
tube formation	210	**2.5797e-32**	**1.6080e-30**	2.0208	347	1.66%	307	**9.6779e-9**	1.5497	113	165	1.20%
embryonic limb morphogenesis	234	**3.5163e-30**	**1.9670e-28**	2.0205	323	1.55%	338	**2.8346e-8**	1.5714	100	144	1.06%
gland morphogenesis	244	**2.2338e-29**	**1.1984e-27**	2.0307	310	1.48%	350	**5.9832e-8**	1.5983	89	126	0.94%
hair cycle	265	**3.7487e-27**	**1.8517e-25**	2.2357	228	1.09%	432	**1.2715e-6**	1.6163	70	98	0.74%
odontogenesis	266	**3.7995e-27**	**1.8698e-25**	2.1305	254	1.22%	494	**6.3188e-6**	1.5584	73	106	0.77%
odontogenesis of dentin-containing tooth	267	**7.6039e-27**	**3.7279e-25**	2.3054	211	1.01%	551	**3.3132e-5**	1.6040	56	79	0.59%
cranial skeletal system development	281	**1.8479e-25**	**8.6084e-24**	2.4833	172	0.82%	620	**1.6814e-4**	1.6114	47	66	0.50%
somatic stem cell population maintenance	288	**6.9481e-25**	**3.1580e-23**	2.6611	148	0.71%	511	**1.0952e-5**	1.8010	39	49	0.41%
hair cycle process	297	**1.4597e-24**	**6.4336e-23**	2.2009	212	1.02%	438	**1.5526e-6**	1.6457	64	88	0.68%
hair follicle development	307	**3.8710e-24**	**1.6505e-22**	2.2109	206	0.99%	492	**6.2440e-6**	1.6239	61	85	0.65%

Term Name	Binom Rank	Binom Raw P-Value	*Binom FDR Q-Val*	Binom Fold Enrichment	Binom Observed Region Hits	Binom Region Set Coverage	Hyper Rank	*Hyper FDR Q-Val*	Hyper Fold Enrichment	Hyper Observed Gene Hits	Hyper Total Genes	Hyper Gene Set Coverage
canonical Wnt signaling pathway	310	**4.5065e-24**	**1.9029e-22**	2.1574	217	1.04%	520	**1.3583e-5**	1.5721	66	95	0.70%
mesoderm development	318	**1.3298e-23**	**5.4741e-22**	2.0670	235	1.13%	356	**8.5380e-8**	1.6309	80	111	0.85%
skin epidermis development	321	**1.7286e-23**	**7.0492e-22**	2.1819	206	0.99%	532	**2.1533e-5**	1.5866	61	87	0.65%
non-canonical Wnt signaling pathway	359	**8.1133e-21**	**2.9583e-19**	2.9309	104	0.50%	938	**4.1173e-3**	1.6810	26	35	0.27%
embryonic skeletal system morphogenesis	364	**2.0555e-20**	**7.3919e-19**	2.1348	186	0.89%	1,138	**1.1970e-2**	1.3577	60	100	0.63%
keratinocyte differentiation	401	**6.8715e-19**	**2.2431e-17**	2.2360	154	0.74%	1,459	**4.6046e-2**	1.2843	63	111	0.67%
positive regulation of mesenchymal cell proliferation	413	**1.3606e-18**	**4.3123e-17**	2.3970	131	0.63%	465	**3.2924e-6**	1.9570	32	37	0.34%
embryonic hindlimb morphogenesis	419	**3.8616e-18**	**1.2064e-16**	2.4837	119	0.57%	1,016	**6.4749e-3**	1.6638	25	34	0.26%
regulation of mesenchymal cell proliferation	422	**5.2678e-18**	**1.6340e-16**	2.2509	144	0.69%	421	**8.9362e-7**	1.9108	38	45	0.40%

The test set of 20,886 genomic regions picked 9,455 (44%) of all 21,395 genes.
GO Biological Process has 13,090 terms covering 17,925 (84%) of all 21,395 genes, and 1,163,819 term - gene associations.
13,090 ontology terms (100%) were tested using an annotation count range of [1, Inf].

GO Cellular Component (5 terms)

Global controls

Table controls: Export Shown top rows in this table: 20 Set Term annotation count: Min: 1 Max: Inf Set Visualize this table: [select one]

Term Name	Binom Rank	Binom Raw P-Value	*Binom FDR Q-Val*	Binom Fold Enrichment	Binom Observed Region Hits	Binom Region Set Coverage	Hyper Rank	*Hyper FDR Q-Val*	Hyper Fold Enrichment	Hyper Observed Gene Hits	Hyper Total Genes	Hyper Gene Set Coverage
actin filament bundle	72	**4.6695e-13**	**1.0986e-11**	2.0033	132	0.63%	143	**2.3533e-2**	1.3949	45	73	0.48%
desmosome	116	**8.0699e-8**	**1.1785e-6**	2.2624	54	0.26%	87	**8.4850e-4**	1.8587	23	28	0.24%
laminin complex	184	**9.1010e-5**	**8.3788e-4**	2.7912	19	0.09%	137	**1.7958e-2**	2.2628	8	8	0.08%
Golgi cis cisterna	236	**6.1781e-4**	**4.4346e-3**	2.8357	14	0.07%	137	**1.7958e-2**	2.2628	8	8	0.08%
growth cone membrane	289	**3.1616e-3**	**1.8532e-2**	2.1601	17	0.08%	156	**3.5702e-2**	2.2628	7	7	0.07%

The test set of 20,886 genomic regions picked 9,455 (44%) of all 21,395 genes.
GO Cellular Component has 1,694 terms covering 19,074 (89%) of all 21,395 genes, and 371,380 term - gene associations.
1,694 ontology terms (100%) were tested using an annotation count range of [1, Inf].

GO Molecular Function (3 terms)

Global controls

Table controls: Export Shown top rows in this table: 20 Set Term annotation count: Min: 1 Max: Inf Set Visualize this table: [select one]

Term Name	Binom Rank	Binom Raw P-Value	*Binom FDR Q-Val*	Binom Fold Enrichment	Binom Observed Region Hits	Binom Region Set Coverage	Hyper Rank	*Hyper FDR Q-Val*	Hyper Fold Enrichment	Hyper Observed Gene Hits	Hyper Total Genes	Hyper Gene Set Coverage
frizzled binding	44	**3.5313e-17**	**3.3154e-15**	2.6284	101	0.48%	110	**2.7639e-2**	1.6078	27	38	0.29%

Term Name	Binom Rank	Binom Raw P-Value	*Binom FDR Q-Val*	Binom Fold Enrichment	Binom Observed Region Hits	Binom Region Set Coverage	Hyper Rank	*Hyper FDR Q-Val*	Hyper Fold Enrichment	Hyper Observed Gene Hits	Hyper Total Genes	Hyper Gene Set Coverage
I-SMAD binding	143	**1.0660e-5**	**3.0796e-4**	2.3563	33	0.16%	80	**6.4624e-3**	2.2628	11	11	0.12%
neuropilin binding	186	**8.5861e-5**	**1.9069e-3**	2.2009	30	0.14%	101	**1.7408e-2**	2.0888	12	13	0.13%

The test set of 20,886 genomic regions picked 9,455 (44%) of all 21,395 genes.
GO Molecular Function has 4,131 terms covering 17,189 (80%) of all 21,395 genes, and 227,341 term - gene associations.
4,131 ontology terms (100%) were tested using an annotation count range of [1, Inf].

Human Phenotype (20+ terms)

Global controls

Table controls: Export | Shown top rows in this table: 20 Set | Term annotation count: Min: 1 Max: Inf Set | Visualize this table: [select one]

Term Name	Binom Rank	Binom Raw P-Value	*Binom FDR Q-Val*	Binom Fold Enrichment	Binom Observed Region Hits	Binom Region Set Coverage	Hyper Rank	*Hyper FDR Q-Val*	Hyper Fold Enrichment	Hyper Observed Gene Hits	Hyper Total Genes	Hyper Gene Set Coverage
Abnormality of the nail	12	**3.1718e-34**	**1.7442e-31**	2.0989	336	1.61%	105	**1.8855e-3**	1.3277	115	196	1.22%
Cleft lip	18	**4.8097e-31**	**1.7633e-28**	2.3108	245	1.17%	134	**3.6532e-3**	1.4400	63	99	0.67%
Aplasia/Hypoplasia of the eyebrow	22	**8.3668e-30**	**2.5097e-27**	2.7557	169	0.81%	219	**1.7735e-2**	1.4457	46	72	0.49%
Abnormal number of teeth	25	**4.5672e-29**	**1.2056e-26**	2.1920	256	1.23%	141	**4.3276e-3**	1.4169	67	107	0.71%
Cleft upper lip	35	**1.2807e-26**	**2.4146e-24**	2.2276	225	1.08%	155	**5.9888e-3**	1.4356	59	93	0.62%
Abnormality of the eyelashes	36	**1.3291e-26**	**2.4364e-24**	2.3380	203	0.97%	109	**2.0755e-3**	1.4563	65	101	0.69%
Aplasia/Hypoplasia of the nails	39	**2.9555e-26**	**5.0009e-24**	2.5516	169	0.81%	165	**7.1203e-3**	1.4784	49	75	0.52%
Reduced number of teeth	49	**7.1761e-24**	**9.6643e-22**	2.1942	207	0.99%	226	**1.9926e-2**	1.3815	58	95	0.61%
Sparse or absent eyelashes	51	**2.4536e-23**	**3.1747e-21**	2.8953	120	0.57%	211	**1.5916e-2**	1.5557	33	48	0.35%
Anonychia	58	**1.5548e-21**	**1.7690e-19**	3.7726	76	0.36%	171	**8.5958e-3**	1.9966	15	17	0.16%
Finger syndactyly	65	**1.3284e-20**	**1.3486e-18**	2.6445	122	0.58%	9	**1.1746e-6**	2.0365	36	40	0.38%
Nail dysplasia	81	**3.7252e-18**	**3.0349e-16**	2.1215	166	0.79%	302	**4.0534e-2**	1.3528	55	92	0.58%
Abnormality of the plantar skin of foot	95	**6.0902e-17**	**4.2305e-15**	2.5247	107	0.51%	93	**1.6068e-3**	1.6536	38	52	0.40%
Plantar hyperkeratosis	97	**7.2613e-17**	**4.9399e-15**	3.1214	75	0.36%	246	**2.2752e-2**	1.6457	24	33	0.25%
Hypodontia	100	**1.0258e-16**	**6.7690e-15**	2.2326	135	0.65%	312	**4.5947e-2**	1.4331	38	60	0.40%
Abnormality of hair texture	133	**3.8755e-14**	**1.9229e-12**	2.0227	141	0.68%	139	**4.2749e-3**	1.4512	59	92	0.62%
Abnormality of epidermal morphology	139	**9.3992e-14**	**4.4622e-12**	2.5868	81	0.39%	314	**4.6472e-2**	1.5482	26	38	0.27%
Abnormal blistering of the skin	156	**9.2997e-13**	**3.9339e-11**	4.0533	39	0.19%	297	**3.9322e-2**	1.9396	12	14	0.13%
Cutaneous syndactyly	197	**2.8941e-11**	**9.6944e-10**	2.3409	78	0.37%	17	**2.8645e-5**	2.1012	26	28	0.27%
Fragile nails	198	**3.1876e-11**	**1.0624e-9**	3.3935	42	0.20%	142	**4.4170e-3**	2.1120	14	15	0.15%

The test set of 20,886 genomic regions picked 9,455 (44%) of all 21,395 genes.
Human Phenotype has 6,599 terms covering 3,215 (15%) of all 21,395 genes, and 244,972 term - gene associations.

6,599 ontology terms (100%) were tested using an annotation count range of [1, Inf].

Mouse Phenotype Single KO (20+ terms)

Global controls

Table controls: Export | Shown top rows in this table: 20 Set | Term annotation count: Min: 1 Max: Inf Set | Visualize this table: [select one]

Term Name	Binom Rank	Binom Raw P-Value	*Binom FDR Q-Val*	Binom Fold Enrichment	Binom Observed Region Hits	Binom Region Set Coverage	Hyper Rank	*Hyper FDR Q-Val*	Hyper Fold Enrichment	Hyper Observed Gene Hits	Hyper Total Genes	Hyper Gene Set Coverage
abnormal vibrissa morphology	112	**9.2927e-24**	**7.6084e-22**	2.2036	204	0.98%	256	**6.1663e-7**	1.6435	69	95	0.73%
abnormal cardiac outflow tract development	127	**3.6388e-22**	**2.6274e-20**	2.0047	237	1.13%	213	**8.9432e-8**	1.6191	83	116	0.88%
abnormal sphenoid bone morphology	139	**2.2335e-21**	**1.4735e-19**	2.1928	184	0.88%	168	**6.5612e-9**	1.8162	61	76	0.65%
abnormal tongue morphology	142	**5.2565e-21**	**3.3945e-19**	2.1295	193	0.92%	419	**3.5544e-5**	1.5685	61	88	0.65%
abnormal epidermis stratum spinosum morphology	165	**1.4210e-19**	**7.8972e-18**	2.6110	118	0.56%	1,182	**1.5900e-2**	1.4435	37	58	0.39%
abnormal hair shaft morphology	178	**5.7720e-19**	**2.9735e-17**	2.3610	138	0.66%	606	**5.9133e-4**	1.5666	45	65	0.48%
abnormal epidermis stratum basale morphology	198	**2.5862e-17**	**1.1977e-15**	2.8083	91	0.44%	1,292	**2.2070e-2**	1.5517	24	35	0.25%
abnormal merocrine gland morphology	200	**3.0943e-17**	**1.4187e-15**	2.0319	174	0.83%	281	**1.1339e-6**	1.7124	56	74	0.59%
abnormal basicranium morphology	206	**5.1547e-17**	**2.2946e-15**	2.1129	156	0.75%	259	**6.7392e-7**	1.7456	54	70	0.57%
abnormal frontal bone morphology	221	**4.9979e-16**	**2.0738e-14**	2.0344	160	0.77%	391	**2.1212e-5**	1.6881	47	63	0.50%
abnormal epidermis stratum granulosum morphology	222	**5.2441e-16**	**2.1662e-14**	2.2625	125	0.60%	1,220	**1.7223e-2**	1.4234	39	62	0.41%
abnormal tooth development	223	**5.3293e-16**	**2.1915e-14**	2.1110	146	0.70%	892	**4.9023e-3**	1.4743	43	66	0.45%
eyelids open at birth	224	**6.5007e-16**	**2.6612e-14**	2.1117	145	0.69%	388	**2.0636e-5**	1.7505	41	53	0.43%
abnormal midface morphology	232	**2.8103e-15**	**1.1108e-13**	3.9015	50	0.24%	1,360	**2.6061e-2**	2.0365	9	10	0.10%
abnormal hair follicle orientation	233	**2.8687e-15**	**1.1290e-13**	2.9275	74	0.35%	1,260	**2.0580e-2**	1.8385	13	16	0.14%
abnormal keratinocyte morphology	234	**2.9348e-15**	**1.1501e-13**	2.3992	105	0.50%	752	**2.0697e-3**	1.6028	34	48	0.36%
abnormal basisphenoid bone morphology	246	**1.7587e-14**	**6.5557e-13**	2.3091	107	0.51%	254	**5.5474e-7**	1.9471	37	43	0.39%
abnormal nasal septum morphology	265	**7.4468e-14**	**2.5769e-12**	2.4013	94	0.45%	297	**1.7198e-6**	2.0042	31	35	0.33%
epidermis stratum spinosum hyperplasia	266	**8.9135e-14**	**3.0728e-12**	2.5720	82	0.39%	1,400	**2.7643e-2**	1.4902	27	41	0.29%
abnormal keratinocyte physiology	270	**1.0351e-13**	**3.5156e-12**	2.1236	121	0.58%	1,102	**1.3031e-2**	1.4367	40	63	0.42%

The test set of 20,886 genomic regions picked 9,455 (44%) of all 21,395 genes.
Mouse Phenotype Single KO has 9,170 terms covering 9,466 (44%) of all 21,395 genes, and 551,620 term - gene associations.
9,170 ontology terms (100%) were tested using an annotation count range of [1, Inf].

Mouse Phenotype (20+ terms)

Global controls

Table controls: Export | Shown top rows in this table: 20 Set | Term annotation count: Min: 1 Max: Inf Set | Visualize this table: [select one]

Term Name	Binom Rank	Binom Raw P-Value	*Binom FDR Q-Val*	Binom Fold Enrichment	Binom Observed Region Hits	Binom Region Set Coverage	Hyper Rank	*Hyper FDR Q-Val*	Hyper Fold Enrichment	Hyper Observed Gene Hits	Hyper Total Genes	Hyper Gene Set Coverage
**abnormal epidermis stratum basale morphology**	106	**7.3426e-35**	**6.6326e-33**	3.0588	169	0.81%	977	**1.3292e-3**	1.5463	41	60	0.43%
**curly tail**	160	**5.2672e-28**	**3.1521e-26**	2.6337	171	0.82%	906	**8.3646e-4**	1.5307	46	68	0.49%
**abnormal epidermis stratum corneum morphology**	171	**6.2889e-27**	**3.5214e-25**	2.0355	281	1.35%	690	**1.2073e-4**	1.3734	105	173	1.11%
**abnormal ectoderm morphology**	179	**2.7606e-26**	**1.4767e-24**	2.7650	147	0.70%	542	**1.6859e-5**	1.8103	36	45	0.38%
**blistering**	197	**4.3473e-25**	**2.1130e-23**	3.3876	103	0.49%	866	**5.8924e-4**	1.7828	26	33	0.27%
**abnormal vibrissa morphology**	199	**4.8834e-25**	**2.3497e-23**	2.0483	256	1.23%	314	**4.6668e-8**	1.6163	85	119	0.90%
**abnormal apical ectodermal ridge morphology**	211	**3.1159e-24**	**1.4139e-22**	2.8122	131	0.63%	565	**2.3039e-5**	1.8567	32	39	0.34%
**abnormal tongue morphology**	214	**6.5298e-24**	**2.9216e-22**	2.0208	252	1.21%	439	**1.6496e-6**	1.5463	82	120	0.87%
**thick epidermis**	230	**1.2159e-22**	**5.0617e-21**	2.0547	228	1.09%	677	**1.0459e-4**	1.4574	76	118	0.80%
**abnormal epidermis stratum spinosum morphology**	231	**1.2962e-22**	**5.3728e-21**	2.4288	158	0.76%	968	**1.2697e-3**	1.4795	51	78	0.54%
**abnormal sphenoid bone morphology**	246	**1.8676e-21**	**7.2692e-20**	2.0361	220	1.05%	260	**5.6636e-9**	1.7143	75	99	0.79%
**decreased intestinal adenoma incidence**	253	**5.8296e-21**	**2.2063e-19**	5.6889	48	0.23%	1,697	**1.8551e-2**	2.2628	7	7	0.07%
**abnormal basicranium morphology**	255	**6.6410e-21**	**2.4936e-19**	2.0885	201	0.96%	366	**2.1703e-7**	1.6610	69	94	0.73%
**impaired skin barrier function**	264	**1.6921e-20**	**6.1370e-19**	2.2203	171	0.82%	920	**9.3715e-4**	1.4512	59	92	0.62%
**abnormal keratinocyte morphology**	267	**1.8422e-20**	**6.6065e-19**	2.2009	174	0.83%	632	**6.9824e-5**	1.5428	60	88	0.63%
**abnormal dendritic cell number**	271	**2.1323e-20**	**7.5338e-19**	2.0572	203	0.97%	657	**8.3913e-5**	1.4534	79	123	0.84%
**abnormal hair shaft morphology**	277	**3.2789e-20**	**1.1334e-18**	2.1877	174	0.83%	755	**2.4211e-4**	1.5085	58	87	0.61%
**decreased organ/body region tumor incidence**	280	**3.7680e-20**	**1.2885e-18**	2.5194	130	0.62%	1,203	**4.0506e-3**	1.5557	33	48	0.35%
**abnormal substantia nigra morphology**	283	**6.1182e-20**	**2.0700e-18**	3.2254	86	0.41%	1,492	**1.0271e-2**	1.6762	20	27	0.21%
**oligodactyly**	285	**8.3295e-20**	**2.7984e-18**	2.1503	177	0.85%	382	**2.7015e-7**	1.7529	55	71	0.58%

The test set of 20,886 genomic regions picked 9,455 (44%) of all 21,395 genes.
Mouse Phenotype has 9,575 terms covering 9,654 (45%) of all 21,395 genes, and 705,265 term - gene associations.
9,575 ontology terms (100%) were tested using an annotation count range of [1, Inf].
