# Supplementary material for: AP-2α and AP-2β cooperatively function in the craniofacial surface ectoderm to regulate chromatin and gene expression dynamics during facial development
Source: eLife. 2022 Mar 25;11:e70511. doi: 10.7554/eLife.70511 (PMC9038197; doi:10.7554/eLife.70511)
Supplement: Figure 2—source data 9. [file elife-70511-fig2-data9.pdf]

Homer Known Motif Enrichment Results (/Users/vanottee/output2/)

Homer *de novo* Motif Results
Gene Ontology Enrichment Results
Known Motif Enrichment Results (txt file)
Total Target Sequences = 24808, Total Background Sequences = 24866

Rank	Motif	Name	P-value	log P-pvalue	q-value (Benjamini)	# Target Sequences with Motif	% of Targets Sequences with Motif	# Background Sequences with Motif	% of Background Sequences with Motif	Motif File	SVG
1	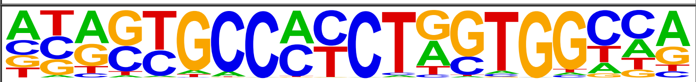	CTCF(Zf)/CD4+-CTCF-ChIP-Seq(Barski_et_al.)/Homer	1e-1276	-2.938e+03	0.0000	4788.0	19.30%	1337.8	5.38%	motif file (matrix)	svg
2	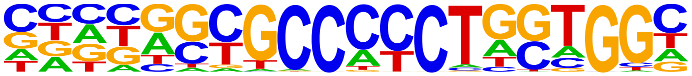	BORIS(Zf)/K562-CTCFL-ChIP-Seq(GSE32465)/Homer	1e-1067	-2.458e+03	0.0000	5600.0	22.58%	2012.5	8.09%	motif file (matrix)	svg
3	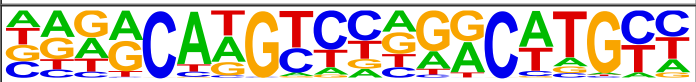	p73(p53)/Trachea-p73-ChIP-Seq(PRJNA310161)/Homer	1e-436	-1.006e+03	0.0000	1542.0	6.22%	389.6	1.57%	motif file (matrix)	svg
4	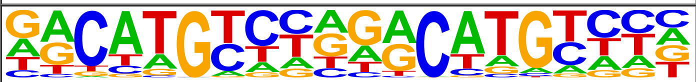	p53(p53)/Saos-p53-ChIP-Seq(GSE15780)/Homer	1e-417	-9.620e+02	0.0000	2231.0	8.99%	772.5	3.11%	motif file (matrix)	svg
5	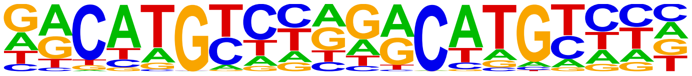	p53(p53)/Saos-p53-ChIP-Seq/Homer	1e-417	-9.620e+02	0.0000	2231.0	8.99%	772.5	3.11%	motif file (matrix)	svg
6	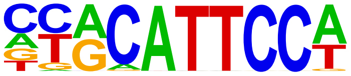	TEAD1(TEAD)/HepG2-TEAD1-ChIP-Seq(Encode)/Homer	1e-399	-9.202e+02	0.0000	9404.0	37.91%	6352.4	25.54%	motif file (matrix)	svg
7	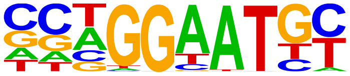	TEAD4(TEA)/Tropoblast-Tead4-ChIP-Seq(GSE37350)/Homer	1e-365	-8.415e+02	0.0000	8833.0	35.61%	5969.0	24.00%	motif file (matrix)	svg
8	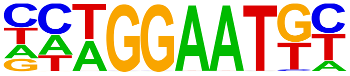	TEAD(TEA)/Fibroblast-PU.1-ChIP-Seq(Unpublished)/Homer	1e-359	-8.274e+02	0.0000	6773.0	27.30%	4222.2	16.98%	motif file (matrix)	svg
9	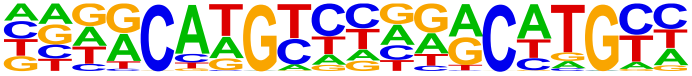	p63(p53)/Keratinocyte-p63-ChIP-Seq(GSE17611)/Homer	1e-357	-8.243e+02	0.0000	5510.0	22.21%	3199.3	12.86%	motif file (matrix)	svg
10	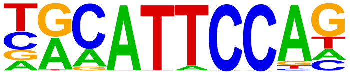	TEAD3(TEA)/HepG2-TEAD3-ChIP-Seq(Encode)/Homer	1e-350	-8.074e+02	0.0000	10258.0	41.35%	7308.5	29.39%	motif file (matrix)	svg
11	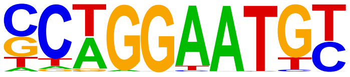	TEAD2(TEA)/Py2T-Tead2-ChIP-Seq(GSE55709)/Homer	1e-308	-7.104e+02	0.0000	6009.0	24.22%	3751.8	15.09%	motif file (matrix)	svg
12	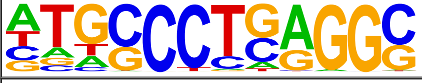	AP-2alpha(AP2)/Hela-AP2alpha-ChIP-Seq(GSE31477)/Homer	1e-215	-4.971e+02	0.0000	9649.0	38.90%	7352.6	29.57%	motif file (matrix)	svg
13	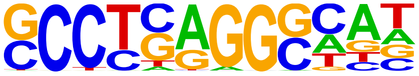	AP-2gamma(AP2)/MCF7-TFAP2C-ChIP-Seq(GSE21234)/Homer	1e-195	-4.507e+02	0.0000	11429.0	46.08%	9150.5	36.79%	motif file (matrix)	svg
14	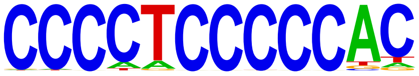	Zfp281(Zf)/ES-Zfp281-ChIP-Seq(GSE81042)/Homer	1e-137	-3.172e+02	0.0000	4186.0	16.88%	2860.1	11.50%	motif file (matrix)	svg
15		GRHL2(CP2)/HBE-GRHL2-ChIP-	1e-	-2.733e+02	0.0000	4054.0	16.34%	2834.7	11.40%	motif	svg

	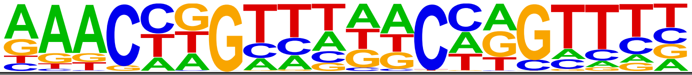	Seq(GSE46194)/Homer	118							file (matrix)	
16	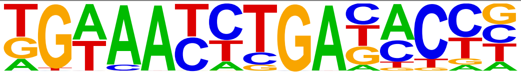	Six4(Homeobox)/MCF7-SIX4-ChIP-Seq(Encode)/Homer	1e-108	-2.495e+02	0.0000	789.0	3.18%	322.1	1.30%	motif file (matrix)	svg
17	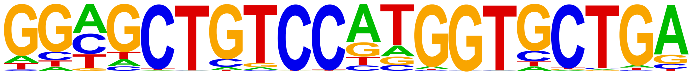	REST-NRSF(Zf)/Jurkat-NRSF-ChIP-Seq/Homer	1e-105	-2.429e+02	0.0000	316.0	1.27%	68.5	0.28%	motif file (matrix)	svg
18	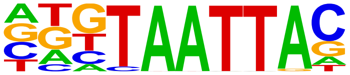	Dlx3(Homeobox)/Kerainocytes-Dlx3-ChIP-Seq(GSE89884)/Homer	1e-95	-2.195e+02	0.0000	4413.0	17.79%	3266.7	13.14%	motif file (matrix)	svg
19	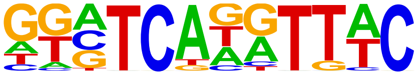	Six1(Homeobox)/Myoblast-Six1-ChIP-Chip(GSE20150)/Homer	1e-90	-2.086e+02	0.0000	2443.0	9.85%	1607.5	6.46%	motif file (matrix)	svg
20	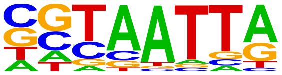	DLX5(Homeobox)/BasalGanglia-Dlx5-ChIP-seq(GSE124936)/Homer	1e-83	-1.929e+02	0.0000	5179.0	20.88%	4022.0	16.17%	motif file (matrix)	svg
21	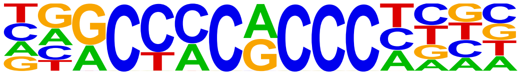	KLF3(Zf)/MEF-Klf3-ChIP-Seq(GSE44748)/Homer	1e-82	-1.906e+02	0.0000	6365.0	25.66%	5113.3	20.56%	motif file (matrix)	svg
22	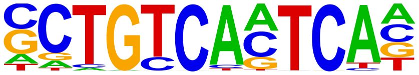	Pbx3(Homeobox)/GM12878-PBX3-ChIP-Seq(GSE32465)/Homer	1e-79	-1.836e+02	0.0000	2947.0	11.88%	2078.0	8.36%	motif file (matrix)	svg
23	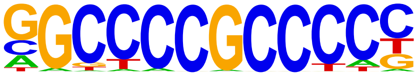	Sp1(Zf)/Promoter/Homer	1e-74	-1.718e+02	0.0000	3314.0	13.36%	2421.3	9.74%	motif file (matrix)	svg
24	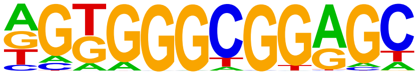	Sp5(Zf)/mES-Sp5.Flag-ChIP-Seq(GSE72989)/Homer	1e-71	-1.650e+02	0.0000	10357.0	41.75%	9007.5	36.22%	motif file (matrix)	svg
25	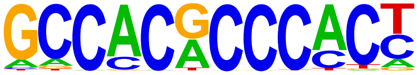	Klf9(Zf)/GBM-Klf9-ChIP-Seq(GSE62211)/Homer	1e-68	-1.571e+02	0.0000	5337.0	21.52%	4275.5	17.19%	motif file (matrix)	svg
26	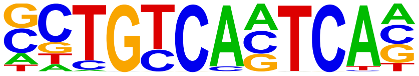	Pknox1(Homeobox)/ES-Prep1-ChIP-Seq(GSE63282)/Homer	1e-65	-1.508e+02	0.0000	2760.0	11.13%	1992.3	8.01%	motif file (matrix)	svg
27	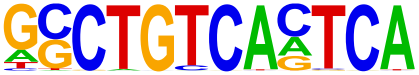	PBX1(Homeobox)/MCF7-PBX1-ChIP-Seq(GSE28007)/Homer	1e-64	-1.489e+02	0.0000	1231.0	4.96%	735.5	2.96%	motif file (matrix)	svg
28	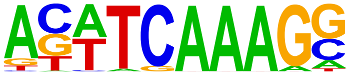	Tcf3(HMG)/mES-Tcf3-ChIP-Seq(GSE11724)/Homer	1e-61	-1.417e+02	0.0000	2446.0	9.86%	1744.7	7.02%	motif file (matrix)	svg
29	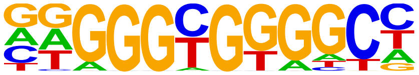	KLF1(Zf)/HUDEP2-KLF1-CutnRun(GSE136251)/Homer	1e-53	-1.235e+02	0.0000	9563.0	38.55%	8418.6	33.85%	motif file (matrix)	svg
30	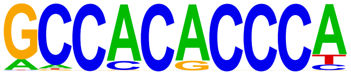	Klf4(Zf)/mES-Klf4-ChIP-Seq(GSE11431)/Homer	1e-53	-1.232e+02	0.0000	4750.0	19.15%	3853.0	15.49%	motif file (matrix)	svg
31	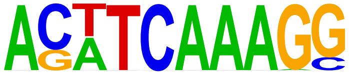	TCFL2(HMG)/K562-TCF7L2-ChIP-Seq(GSE29196)/Homer	1e-52	-1.217e+02	0.0000	968.0	3.90%	571.6	2.30%	motif file (matrix)	svg
32	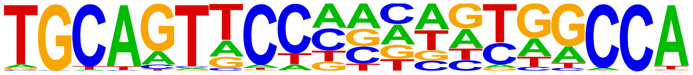	CTCF-SatelliteElement(Zf?)/CD4+-CTCF-ChIP-Seq(Barski_et_al.)/Homer	1e-50	-1.166e+02	0.0000	396.0	1.60%	168.3	0.68%	motif file (matrix)	svg
33		p53(p53)/mES-cMyc-ChIP-Seq(GSE11431)/Homer	1e-43	-1.006e+02	0.0000	354.0	1.43%	153.1	0.62%	motif file	svg

	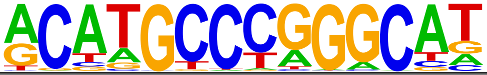									(matrix)	
34	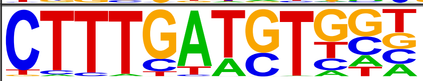	Tcf7(HMG)/GM12878-TCF7-ChIP-Seq(Encode)/Homer	1e-42	-9.741e+01	0.0000	2922.0	11.78%	2280.7	9.17%	motif file (matrix)	svg
35	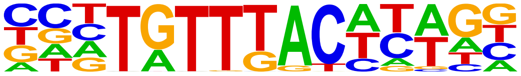	Foxa3(Forkhead)/Liver-Foxa3-ChIP-Seq(GSE77670)/Homer	1e-41	-9.593e+01	0.0000	2460.0	9.92%	1874.1	7.54%	motif file (matrix)	svg
36	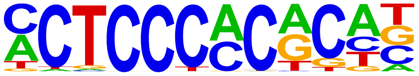	WT1(Zf)/Kidney-WT1-ChIP-Seq(GSE90016)/Homer	1e-41	-9.531e+01	0.0000	6521.0	26.29%	5626.1	22.62%	motif file (matrix)	svg
37	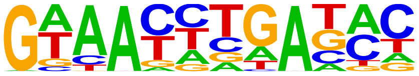	Six2(Homeobox)/NephronProgenitor-Six2-ChIP-Seq(GSE39837)/Homer	1e-41	-9.492e+01	0.0000	7276.0	29.33%	6349.2	25.53%	motif file (matrix)	svg
38	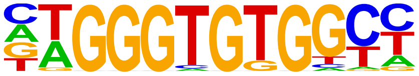	EKLF(Zf)/Erythrocyte-Klf1-ChIP-Seq(GSE20478)/Homer	1e-40	-9.352e+01	0.0000	2728.0	11.00%	2119.7	8.52%	motif file (matrix)	svg
39	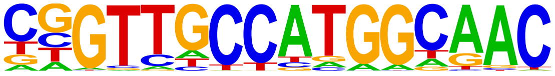	RFX(HTH)/K562-RFX3-ChIP-Seq(SRA012198)/Homer	1e-39	-8.998e+01	0.0000	1082.0	4.36%	712.8	2.87%	motif file (matrix)	svg
40	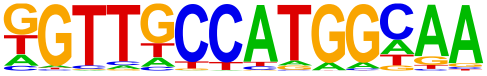	X-box(HTH)/NPC-H3K4me1-ChIP-Seq(GSE16256)/Homer	1e-38	-8.833e+01	0.0000	1193.0	4.81%	806.6	3.24%	motif file (matrix)	svg
41	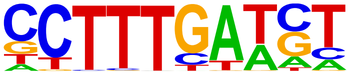	LEF1(HMG)/H1-LEF1-ChIP-Seq(GSE64758)/Homer	1e-38	-8.828e+01	0.0000	5263.0	21.22%	4469.3	17.97%	motif file (matrix)	svg
42	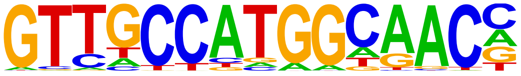	Rfx2(HTH)/LoVo-RFX2-ChIP-Seq(GSE49402)/Homer	1e-37	-8.742e+01	0.0000	1175.0	4.74%	793.8	3.19%	motif file (matrix)	svg
43	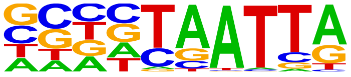	DLX1(Homeobox)/BasalGanglia-Dlx1-ChIP-seq(GSE124936)/Homer	1e-32	-7.503e+01	0.0000	7957.0	32.08%	7113.5	28.60%	motif file (matrix)	svg
44	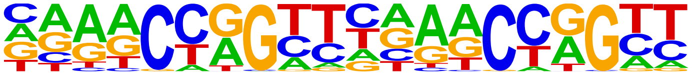	Tcfcp2l1(CP2)/mES-Tcfcp2l1-ChIP-Seq(GSE11431)/Homer	1e-29	-6.710e+01	0.0000	1885.0	7.60%	1453.2	5.84%	motif file (matrix)	svg
45	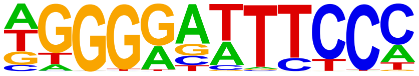	NFkB-p65(RHD)/GM12787-p65-ChIP-Seq(GSE19485)/Homer	1e-24	-5.601e+01	0.0000	4372.0	17.63%	3788.8	15.23%	motif file (matrix)	svg
46	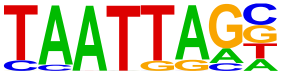	Lhx2(Homeobox)/HFSC-Lhx2-ChIP-Seq(GSE48068)/Homer	1e-23	-5.469e+01	0.0000	6523.0	26.30%	5851.8	23.53%	motif file (matrix)	svg
47	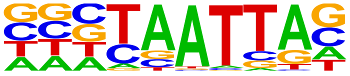	DLX2(Homeobox)/BasalGanglia-Dlx2-ChIP-seq(GSE124936)/Homer	1e-23	-5.389e+01	0.0000	8754.0	35.29%	8027.1	32.28%	motif file (matrix)	svg
48	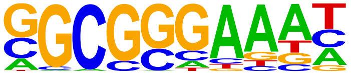	E2F4(E2F)/K562-E2F4-ChIP-Seq(GSE31477)/Homer	1e-22	-5.268e+01	0.0000	3245.0	13.08%	2748.1	11.05%	motif file (matrix)	svg
49	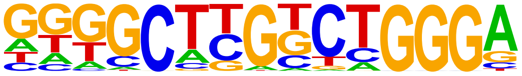	Zfp809(Zf)/ES-Zfp809-ChIP-Seq(GSE70799)/Homer	1e-22	-5.107e+01	0.0000	2364.0	9.53%	1942.6	7.81%	motif file (matrix)	svg
50	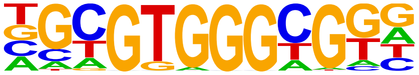	Egr2(Zf)/Thymocytes-Egr2-ChIP-Seq(GSE34254)/Homer	1e-22	-5.095e+01	0.0000	1942.0	7.83%	1559.8	6.27%	motif file (matrix)	svg
51		NFkB-p50,p52(RHD)/Monocyte-p50-ChIP-Chip(Schreiber_et_al.)/Homer	1e-21	-4.931e+01	0.0000	1199.0	4.83%	904.7	3.64%	motif file	svg

	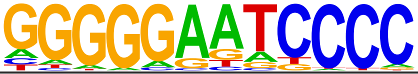									(matrix)	
52	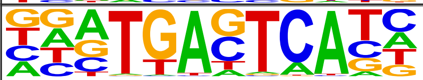	Fra1(bZIP)/BT549-Fra1-ChIP-Seq(GSE46166)/Homer	1e-19	-4.488e+01	0.0000	3787.0	15.27%	3297.7	13.26%	motif file (matrix)	svg
53	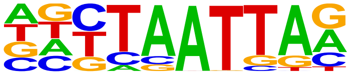	Lhx1(Homeobox)/EmbryoCarcinoma-Lhx1-ChIP-Seq(GSE70957)/Homer	1e-18	-4.315e+01	0.0000	6533.0	26.34%	5940.6	23.89%	motif file (matrix)	svg
54	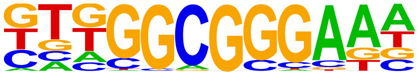	E2F3(E2F)/MEF-E2F3-ChIP-Seq(GSE71376)/Homer	1e-18	-4.241e+01	0.0000	4783.0	19.28%	4259.9	17.13%	motif file (matrix)	svg
55	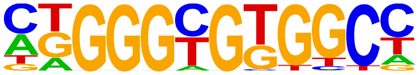	KLF6(Zf)/PDAC-KLF6-ChIP-Seq(GSE64557)/Homer	1e-18	-4.237e+01	0.0000	10122.0	40.81%	9465.7	38.06%	motif file (matrix)	svg
56	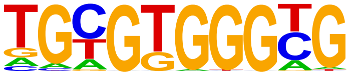	Egr1(Zf)/K562-Egr1-ChIP-Seq(GSE32465)/Homer	1e-17	-3.977e+01	0.0000	6837.0	27.56%	6261.8	25.18%	motif file (matrix)	svg
57	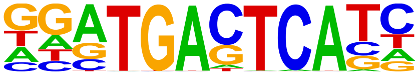	Fra2(bZIP)/Striatum-Fra2-ChIP-Seq(GSE43429)/Homer	1e-17	-3.936e+01	0.0000	3425.0	13.81%	2987.6	12.01%	motif file (matrix)	svg
58	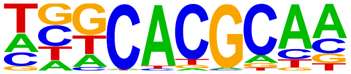	Arnt:Ahr(bHLH)/MCF7-Arnt-ChIP-Seq(Lo_et_al.)/Homer	1e-16	-3.815e+01	0.0000	5261.0	21.21%	4747.4	19.09%	motif file (matrix)	svg
59	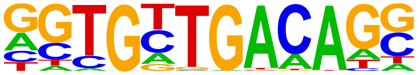	Tbx20(T-box)/Heart-Tbx20-ChIP-Seq(GSE29636)/Homer	1e-16	-3.778e+01	0.0000	2177.0	8.78%	1829.3	7.36%	motif file (matrix)	svg
60	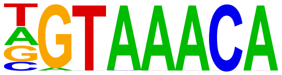	Foxo3(Forkhead)/U2OS-Foxo3-ChIP-Seq(E-MTAB-2701)/Homer	1e-16	-3.773e+01	0.0000	4722.0	19.04%	4232.7	17.02%	motif file (matrix)	svg
61	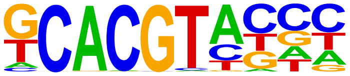	HIF2a(bHLH)/785_O-HIF2a-ChIP-Seq(GSE34871)/Homer	1e-16	-3.734e+01	0.0000	2628.0	10.59%	2250.6	9.05%	motif file (matrix)	svg
62	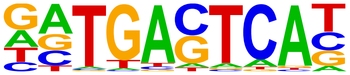	JunB(bZIP)/DendriticCells-Junb-ChIP-Seq(GSE36099)/Homer	1e-15	-3.483e+01	0.0000	3811.0	15.36%	3381.1	13.60%	motif file (matrix)	svg
63	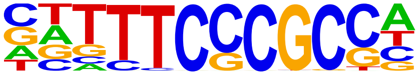	E2F7(E2F)/Hela-E2F7-ChIP-Seq(GSE32673)/Homer	1e-15	-3.464e+01	0.0000	928.0	3.74%	711.2	2.86%	motif file (matrix)	svg
64	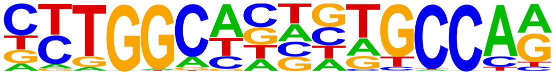	NF1(CTF)/LNCAP-NF1-ChIP-Seq(Unpublished)/Homer	1e-14	-3.390e+01	0.0000	3987.0	16.07%	3555.7	14.30%	motif file (matrix)	svg
65	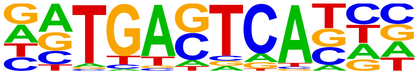	Fosl2(bZIP)/3T3L1-Fosl2-ChIP-Seq(GSE56872)/Homer	1e-14	-3.349e+01	0.0000	2579.0	10.40%	2225.5	8.95%	motif file (matrix)	svg
66	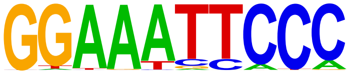	NFkB-p65-Rel(RHD)/ThioMac-LPS-Expression(GSE23622)/Homer	1e-14	-3.281e+01	0.0000	551.0	2.22%	391.1	1.57%	motif file (matrix)	svg
67	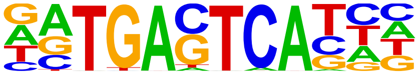	Atf3(bZIP)/GBM-ATF3-ChIP-Seq(GSE33912)/Homer	1e-13	-3.185e+01	0.0000	4450.0	17.94%	4013.2	16.14%	motif file (matrix)	svg
68	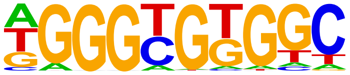	KLF5(Zf)/LoVo-KLF5-ChIP-Seq(GSE49402)/Homer	1e-13	-3.160e+01	0.0000	11741.0	47.33%	11175.0	44.94%	motif file (matrix)	svg
69		FOXP1(Forkhead)/H9-FOXP1-ChIP-Seq(GSE31006)/Homer	1e-13	-3.030e+01	0.0000	2988.0	12.05%	2629.5	10.57%	motif file	svg

	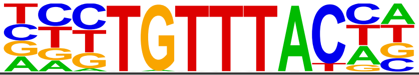									(matrix)	
70	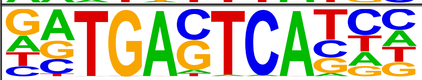	Jun-AP1(bZIP)/K562-cJun-ChIP-Seq(GSE31477)/Homer	1e-13	-2.998e+01	0.0000	1899.0	7.66%	1610.9	6.48%	motif file (matrix)	svg
71	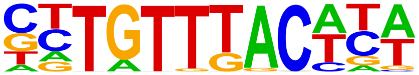	Foxa2(Forkhead)/Liver-Foxa2-ChIP-Seq(GSE25694)/Homer	1e-12	-2.973e+01	0.0000	5419.0	21.85%	4965.4	19.97%	motif file (matrix)	svg
72	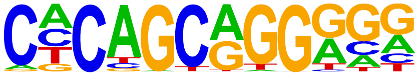	Zic2(Zf)/ESC-Zic2-ChIP-Seq(SRP197560)/Homer	1e-12	-2.920e+01	0.0000	4931.0	19.88%	4497.4	18.08%	motif file (matrix)	svg
73	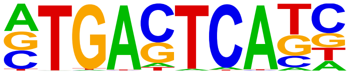	AP-1(bZIP)/ThioMac-PU.1-ChIP-Seq(GSE21512)/Homer	1e-11	-2.744e+01	0.0000	5012.0	20.21%	4590.1	18.46%	motif file (matrix)	svg
74	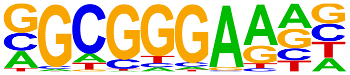	E2F6(E2F)/Hela-E2F6-ChIP-Seq(GSE31477)/Homer	1e-11	-2.684e+01	0.0000	4044.0	16.30%	3661.9	14.73%	motif file (matrix)	svg
75	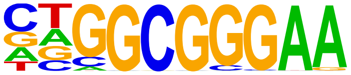	E2F1(E2F)/Hela-E2F1-ChIP-Seq(GSE22478)/Homer	1e-11	-2.667e+01	0.0000	1747.0	7.04%	1486.0	5.98%	motif file (matrix)	svg
76	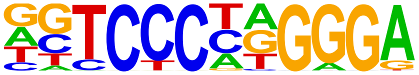	EBF(EBF)/proBcell-EBF-ChIP-Seq(GSE21978)/Homer	1e-11	-2.666e+01	0.0000	2360.0	9.51%	2059.2	8.28%	motif file (matrix)	svg
77	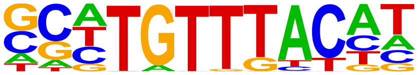	FOXK2(Forkhead)/U2OS-FOXK2-ChIP-Seq(E-MTAB-2204)/Homer	1e-11	-2.610e+01	0.0000	4282.0	17.26%	3896.5	15.67%	motif file (matrix)	svg
78	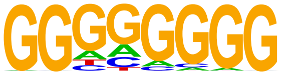	Maz(Zf)/HepG2-Maz-ChIP-Seq(GSE31477)/Homer	1e-10	-2.460e+01	0.0000	11946.0	48.16%	11456.7	46.07%	motif file (matrix)	svg
79	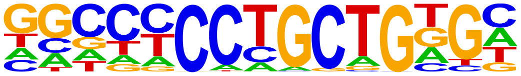	Zic3(Zf)/mES-Zic3-ChIP-Seq(GSE37889)/Homer	1e-10	-2.390e+01	0.0000	6315.0	25.46%	5891.2	23.69%	motif file (matrix)	svg
80	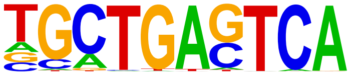	Bach2(bZIP)/OCILy7-Bach2-ChIP-Seq(GSE44420)/Homer	1e-10	-2.386e+01	0.0000	1604.0	6.47%	1368.9	5.50%	motif file (matrix)	svg
81	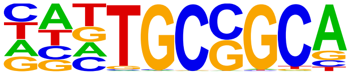	Zfp57(Zf)/H1-ZFP57.HA-ChIP-Seq(GSE115387)/Homer	1e-9	-2.283e+01	0.0000	2839.0	11.45%	2538.2	10.21%	motif file (matrix)	svg
82	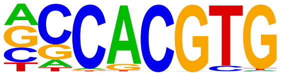	c-Myc(bHLH)/LNCAP-cMyc-ChIP-Seq(Unpublished)/Homer	1e-9	-2.236e+01	0.0000	3629.0	14.63%	3298.7	13.26%	motif file (matrix)	svg
83	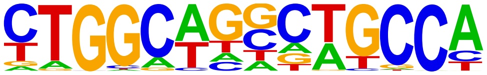	Tlx?(NR)/NPC-H3K4me1-ChIP-Seq(GSE16256)/Homer	1e-9	-2.205e+01	0.0000	4648.0	18.74%	4285.4	17.23%	motif file (matrix)	svg
84	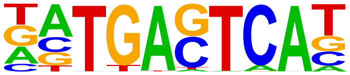	BATF(bZIP)/Th17-BATF-ChIP-Seq(GSE39756)/Homer	1e-9	-2.163e+01	0.0000	4416.0	17.80%	4064.9	16.35%	motif file (matrix)	svg
85	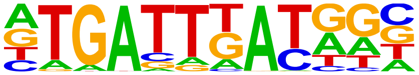	PBX2(Homeobox)/K562-PBX2-ChIP-Seq(Encode)/Homer	1e-9	-2.092e+01	0.0000	5103.0	20.57%	4738.3	19.05%	motif file (matrix)	svg
86	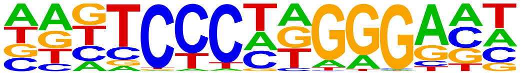	EBF2(EBF)/BrownAdipose-EBF2-ChIP-Seq(GSE97114)/Homer	1e-8	-2.065e+01	0.0000	8363.0	33.71%	7941.8	31.93%	motif file (matrix)	svg
87		JunD(bZIP)/K562-JunD-ChIP-Seq/Homer	1e-8	-2.019e+01	0.0000	698.0	2.81%	557.0	2.24%	motif file	svg

	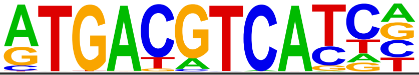									(matrix)	
88	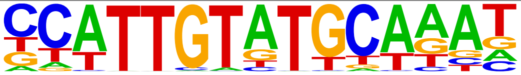	Oct4:Sox17(POU,Homeobox,HMG)/F9-Sox17-ChIP-Seq(GSE44553)/Homer	1e-8	-1.873e+01	0.0000	796.0	3.21%	650.9	2.62%	motif file (matrix)	svg
89	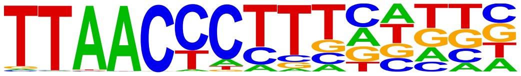	ZNF652/HepG2-ZNF652.Flag-ChIP-Seq(Encode)/Homer	1e-8	-1.864e+01	0.0000	1696.0	6.84%	1484.1	5.97%	motif file (matrix)	svg
90	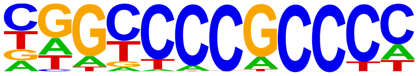	Sp2(Zf)/HEK293-Sp2.eGFP-ChIP-Seq(Encode)/Homer	1e-7	-1.815e+01	0.0000	12910.0	52.05%	12503.1	50.28%	motif file (matrix)	svg
91	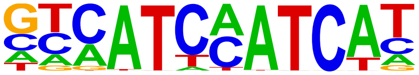	HOXA2(Homeobox)/mES-Hoxa2-ChIP-Seq(Donaldson_et_al.)/Homer	1e-7	-1.814e+01	0.0000	727.0	2.93%	591.0	2.38%	motif file (matrix)	svg
92	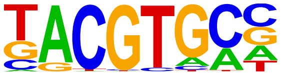	HIF-1a(bHLH)/MCF7-HIF1a-ChIP-Seq(GSE28352)/Homer	1e-7	-1.685e+01	0.0000	1910.0	7.70%	1698.9	6.83%	motif file (matrix)	svg
93	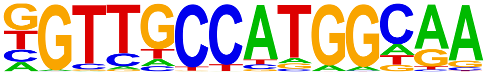	Rfx1(HTH)/NPC-H3K4me1-ChIP-Seq(GSE16256)/Homer	1e-7	-1.623e+01	0.0000	1899.0	7.66%	1692.5	6.81%	motif file (matrix)	svg
94	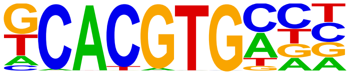	bHLHE40(bHLH)/HepG2-BHLHE40-ChIP-Seq(GSE31477)/Homer	1e-7	-1.622e+01	0.0000	2482.0	10.01%	2248.5	9.04%	motif file (matrix)	svg
95	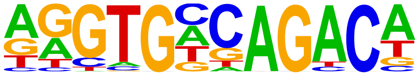	Tbox:Smad(T-box,MAD)/ESCd5-Smad2_3-ChIP-Seq(GSE29422)/Homer	1e-6	-1.569e+01	0.0000	1899.0	7.66%	1696.2	6.82%	motif file (matrix)	svg
96	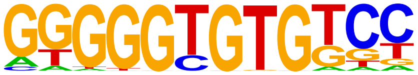	KLF10(Zf)/HEK293-KLF10.GFP-ChIP-Seq(GSE58341)/Homer	1e-6	-1.399e+01	0.0000	7113.0	28.68%	6792.5	27.31%	motif file (matrix)	svg
97	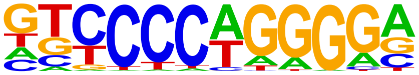	EBF1(EBF)/Near-E2A-ChIP-Seq(GSE21512)/Homer	1e-5	-1.366e+01	0.0000	9723.0	39.20%	9386.0	37.74%	motif file (matrix)	svg
98	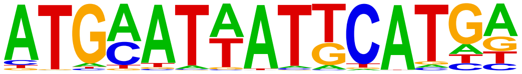	OCT:OCT(POU,Homeobox,IR1)/NPC-Brn2-ChIP-Seq(GSE35496)/Homer	1e-5	-1.276e+01	0.0000	92.0	0.37%	55.2	0.22%	motif file (matrix)	svg
99	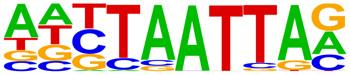	Lhx3(Homeobox)/Neuron-Lhx3-ChIP-Seq(GSE31456)/Homer	1e-5	-1.266e+01	0.0000	9098.0	36.68%	8779.0	35.30%	motif file (matrix)	svg
100	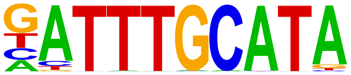	Oct11(POU,Homeobox)/NCIH1048-POU2F3-ChIP-seq(GSE115123)/Homer	1e-5	-1.201e+01	0.0000	1766.0	7.12%	1598.4	6.43%	motif file (matrix)	svg
101	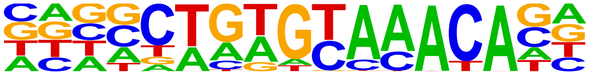	Fox:Ebox(Forkhead,bHLH)/Panc1-Foxa2-ChIP-Seq(GSE47459)/Homer	1e-5	-1.195e+01	0.0000	7807.0	31.47%	7509.3	30.20%	motif file (matrix)	svg
102	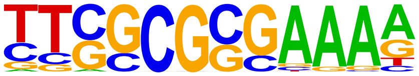	E2F(E2F)/Hela-CellCycle-Expression/Homer	1e-5	-1.176e+01	0.0000	417.0	1.68%	336.5	1.35%	motif file (matrix)	svg
103	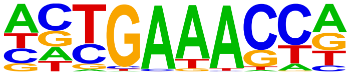	IRF4(IRF)/GM12878-IRF4-ChIP-Seq(GSE32465)/Homer	1e-5	-1.171e+01	0.0000	2938.0	11.84%	2731.0	10.98%	motif file (matrix)	svg
104	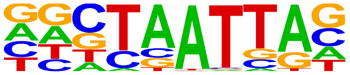	En1(Homeobox)/SUM149-EN1-ChIP-Seq(GSE120957)/Homer	1e-5	-1.162e+01	0.0000	10331.0	41.65%	10024.1	40.31%	motif file (matrix)	svg
105		LXH9(Homeobox)/Hct116-LXH9.V5-ChIP-Seq(GSE116822)/Homer	1e-4	-1.081e+01	0.0001	8393.0	33.84%	8109.9	32.61%	motif file	svg

	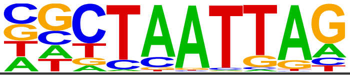									(matrix)	
106	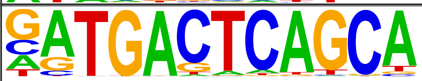	NF-E2(bZIP)/K562-NFE2-ChIP-Seq(GSE31477)/Homer	1e-4	-1.070e+01	0.0001	474.0	1.91%	392.8	1.58%	motif file (matrix)	svg
107	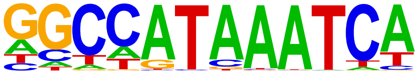	Hoxc9(Homeobox)/Ainv15-Hoxc9-ChIP-Seq(GSE21812)/Homer	1e-4	-1.067e+01	0.0001	2818.0	11.36%	2625.1	10.56%	motif file (matrix)	svg
108	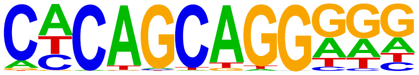	Unknown-ESC-element(?)/mES-Nanog-ChIP-Seq(GSE11724)/Homer	1e-4	-1.055e+01	0.0001	6092.0	24.56%	5835.9	23.47%	motif file (matrix)	svg
109	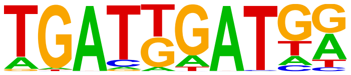	HOXA1(Homeobox)/mES-Hoxa1-ChIP-Seq(SRP084292)/Homer	1e-4	-1.012e+01	0.0002	1703.0	6.87%	1554.6	6.25%	motif file (matrix)	svg
110	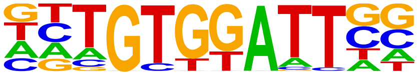	Foxh1(Forkhead)/hESC-FOXH1-ChIP-Seq(GSE29422)/Homer	1e-4	-9.475e+00	0.0003	3962.0	15.97%	3756.1	15.10%	motif file (matrix)	svg
111	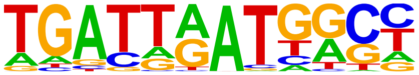	Hoxb4(Homeobox)/ES-Hoxb4-ChIP-Seq(GSE34014)/Homer	1e-4	-9.381e+00	0.0003	1340.0	5.40%	1213.1	4.88%	motif file (matrix)	svg
112	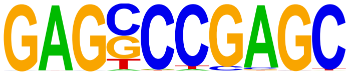	ZNF519(Zf)/HEK293-ZNF519.GFP-ChIP-Seq(GSE58341)/Homer	1e-3	-9.025e+00	0.0005	2172.0	8.76%	2017.7	8.11%	motif file (matrix)	svg
113	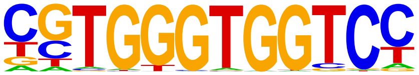	GLI3(Zf)/Limb-GLI3-ChIP-Chip(GSE11077)/Homer	1e-3	-8.910e+00	0.0005	1340.0	5.40%	1217.8	4.90%	motif file (matrix)	svg
114	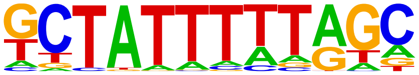	Mef2d(MADS)/Retina-Mef2d-ChIP-Seq(GSE61391)/Homer	1e-3	-8.772e+00	0.0006	984.0	3.97%	879.4	3.54%	motif file (matrix)	svg
115	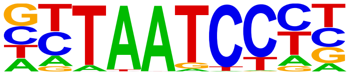	Otx2(Homeobox)/EpiLC-Otx2-ChIP-Seq(GSE56098)/Homer	1e-3	-8.675e+00	0.0007	5992.0	24.16%	5767.6	23.19%	motif file (matrix)	svg
116	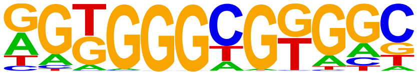	KLF14(Zf)/HEK293-KLF14.GFP-ChIP-Seq(GSE58341)/Homer	1e-3	-8.261e+00	0.0010	14238.0	57.40%	14002.1	56.30%	motif file (matrix)	svg
117	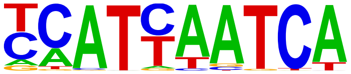	Pdx1(Homeobox)/Islet-Pdx1-ChIP-Seq(SRA008281)/Homer	1e-3	-8.058e+00	0.0012	5391.0	21.73%	5184.4	20.85%	motif file (matrix)	svg
118	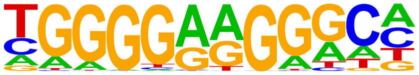	ZNF467(Zf)/HEK293-ZNF467.GFP-ChIP-Seq(GSE58341)/Homer	1e-3	-7.860e+00	0.0014	8578.0	34.58%	8348.3	33.57%	motif file (matrix)	svg
119	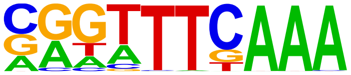	CHR(?)/Hela-CellCycle-Expression/Homer	1e-3	-7.455e+00	0.0021	3420.0	13.79%	3254.2	13.09%	motif file (matrix)	svg
120	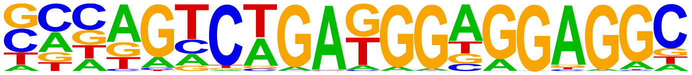	ZSCAN22(Zf)/HEK293-ZSCAN22.GFP-ChIP-Seq(GSE58341)/Homer	1e-3	-7.432e+00	0.0022	889.0	3.58%	799.2	3.21%	motif file (matrix)	svg
121	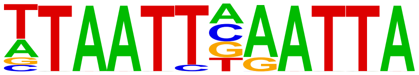	Phox2b(Homeobox)/CLBGA-PHOX2B-ChIP-Seq(GSE90683)/Homer	1e-3	-6.991e+00	0.0033	837.0	3.37%	753.1	3.03%	motif file (matrix)	svg
122	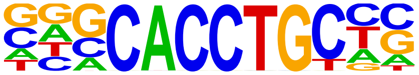	Slug(Zf)/Mesoderm-Snai2-ChIP-Seq(GSE61475)/Homer	1e-3	-6.954e+00	0.0034	5762.0	23.23%	5571.3	22.40%	motif file (matrix)	svg
123		ZNF692(Zf)/HEK293-ZNF692.GFP-ChIP-Seq(GSE58341)/Homer	1e-2	-6.386e+00	0.0060	1398.0	5.64%	1297.0	5.22%	motif file	svg

	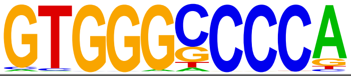									(matrix)	
124	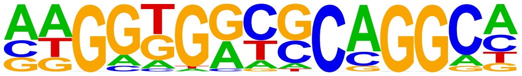	ZNF165(Zf)/WHIM12-ZNF165-ChIP-Seq(GSE65937)/Homer	1e-2	-6.349e+00	0.0062	1380.0	5.56%	1280.2	5.15%	motif file (matrix)	svg
125	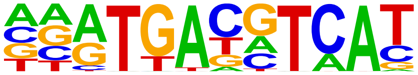	CREB5(bZIP)/LNCaP-CREB5.V5-ChIP-Seq(GSE137775)/Homer	1e-2	-5.927e+00	0.0093	2300.0	9.27%	2180.6	8.77%	motif file (matrix)	svg
126	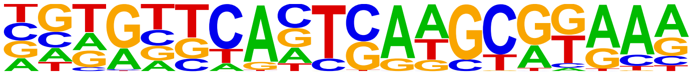	PAX6(Paired,Homeobox)/Forebrain-Pax6-ChIP-Seq(GSE66961)/Homer	1e-2	-5.693e+00	0.0117	698.0	2.81%	631.5	2.54%	motif file (matrix)	svg
127	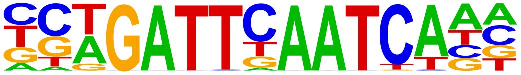	DUX(Homeobox)/C2C12-Dux-ChIP-Seq(GSE87279)/Homer	1e-2	-5.621e+00	0.0125	17.0	0.07%	8.4	0.03%	motif file (matrix)	svg
128	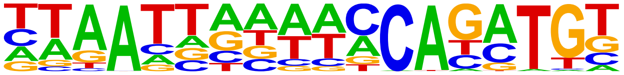	Pitx1:Ebox(Homeobox,bHLH)/Hindlimb-Pitx1-ChIP-Seq(GSE41591)/Homer	1e-2	-5.503e+00	0.0139	1060.0	4.27%	980.1	3.94%	motif file (matrix)	svg
129	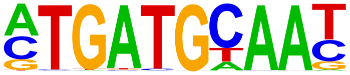	Atf4(bZIP)/MEF-Atf4-ChIP-Seq(GSE35681)/Homer	1e-2	-5.443e+00	0.0147	1420.0	5.72%	1330.0	5.35%	motif file (matrix)	svg
130	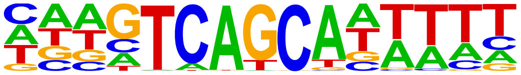	MafF(bZIP)/HepG2-MafF-ChIP-Seq(GSE31477)/Homer	1e-2	-5.107e+00	0.0204	1496.0	6.03%	1407.7	5.66%	motif file (matrix)	svg
131	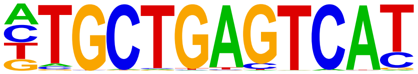	Nrf2(bZIP)/Lymphoblast-Nrf2-ChIP-Seq(GSE37589)/Homer	1e-2	-5.062e+00	0.0212	385.0	1.55%	339.9	1.37%	motif file (matrix)	svg
132	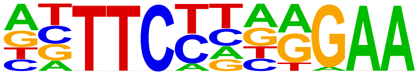	STAT6(Stat)/CD4-Stat6-ChIP-Seq(GSE22104)/Homer	1e-2	-4.812e+00	0.0270	3270.0	13.18%	3151.4	12.67%	motif file (matrix)	svg
133	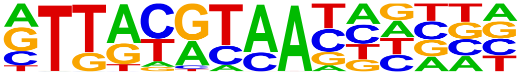	NFIL3(bZIP)/HepG2-NFIL3-ChIP-Seq(Encode)/Homer	1e-2	-4.773e+00	0.0278	3114.0	12.55%	2998.8	12.06%	motif file (matrix)	svg
