# Supplementary material for: AP-2α and AP-2β cooperatively function in the craniofacial surface ectoderm to regulate chromatin and gene expression dynamics during facial development
Source: eLife. 2022 Mar 25;11:e70511. doi: 10.7554/eLife.70511 (PMC9038197; doi:10.7554/eLife.70511)
Supplement: Figure 2—source data 10. [file elife-70511-fig2-data10.pdf]

## ⊕ Job Description

## ⊕ Region-Gene Association Graphs

*What do these graphs illustrate?*

**Number of associated genes per region**

Download as PDF.

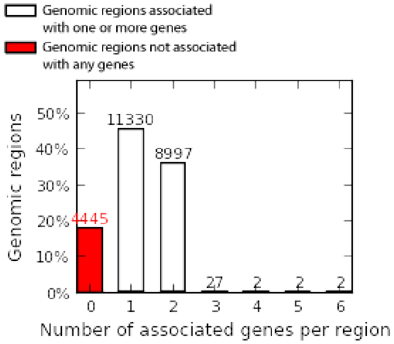


**Binned by orientation and distance to TSS**

Download as PDF.

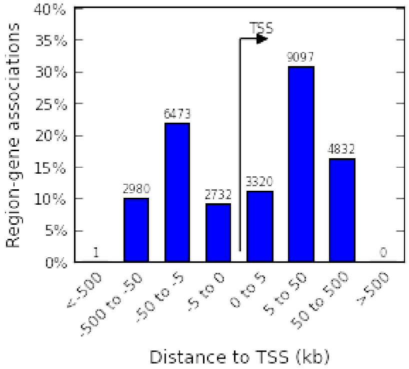


**Binned by absolute distance to TSS**

Download as PDF.

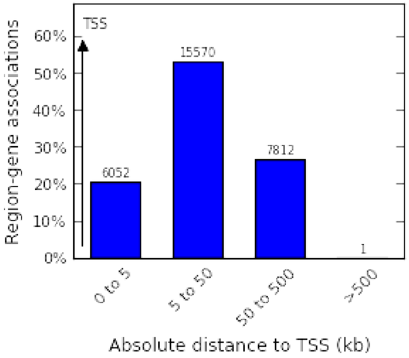


## ⊕ Global Controls

Global Export

*Which data is exported by each option?*

## ⊕ Ensembl Genes (no terms)

Global controls

## ⊖ GO Biological Process (20+ terms)

Global controls

*Table controls:* Export | Shown top rows in this table: 20 Set | Term annotation count: Min: 1 Max: Inf Set | Visualize this table: [select one]

| Term Name | Binom Rank | Binom Raw P-Value | *Binom FDR Q-Val* | Binom Fold Enrichment | Binom Observed Region Hits | Binom Region Set Coverage | Hyper Rank | *Hyper FDR Q-Val* | Hyper Fold Enrichment | Hyper Observed Gene Hits | Hyper Total Genes | Hyper Gene Set Coverage |
|---|---|---|---|---|---|---|---|---|---|---|---|---|
| single-multicellular organism process | 1 | **0.0000** | **0.0000** | 2.0001 | 10,420 | 42.01% | 1 | **2.4473e-139** | 1.3017 | 3,475 | 5,396 | 32.83% |
| multicellular organism development | 1 | **0.0000** | **0.0000** | 2.0657 | 9,621 | 38.79% | 2 | **9.0450e-127** | 1.3137 | 3,069 | 4,722 | 28.99% |
| anatomical structure development | 1 | **0.0000** | **0.0000** | 2.0346 | 9,980 | 40.23% | 3 | **2.8168e-124** | 1.2978 | 3,236 | 5,040 | 30.57% |
| single-organism developmental process | 1 | **0.0000** | **0.0000** | 2.0032 | 10,305 | 41.54% | 4 | **1.8629e-122** | 1.2843 | 3,398 | 5,348 | 32.10% |
| system development | 1 | **0.0000** | **0.0000** | 2.0974 | 8,766 | 35.34% | 7 | **3.6754e-113** | 1.3200 | 2,722 | 4,168 | 25.72% |
| anatomical structure morphogenesis | 1 | **0.0000** | **0.0000** | 2.3402 | 5,498 | 22.16% | 8 | **2.6350e-105** | 1.4590 | 1,500 | 2,078 | 14.17% |
| cell differentiation | 1 | **0.0000** | **0.0000** | 2.1221 | 7,523 | 30.33% | 10 | **1.1261e-95** | 1.3273 | 2,297 | 3,498 | 21.70% |
| regulation of multicellular organismal process | 1 | **0.0000** | **0.0000** | 2.0663 | 5,943 | 23.96% | 11 | **1.5412e-95** | 1.3783 | 1,850 | 2,713 | 17.48% |
| cellular developmental process | 1 | **0.0000** | **0.0000** | 2.1090 | 7,635 | 30.78% | 12 | **7.9571e-94** | 1.3195 | 2,339 | 3,583 | 22.10% |
| animal organ development | 1 | **0.0000** | **0.0000** | 2.1695 | 6,916 | 27.88% | 14 | **2.5174e-90** | 1.3410 | 2,058 | 3,102 | 19.44% |

| Term Name | Binom Rank | Binom Raw P-Value | *Binom FDR Q-Val* | Binom Fold Enrichment | Binom Observed Region Hits | Binom Region Set Coverage | Hyper Rank | *Hyper FDR Q-Val* | Hyper Fold Enrichment | Hyper Observed Gene Hits | Hyper Total Genes | Hyper Gene Set Coverage |
|---|---|---|---|---|---|---|---|---|---|---|---|---|
| tissue development | 1 | **0.0000** | **0.0000** | 2.4020 | 4,330 | 17.46% | 15 | **1.3586e-79** | 1.4565 | 1,171 | 1,625 | 11.06% |
| nervous system development | 1 | **0.0000** | **0.0000** | 2.2183 | 5,305 | 21.39% | 16 | **1.6188e-77** | 1.3856 | 1,493 | 2,178 | 14.10% |
| regulation of developmental process | 1 | **0.0000** | **0.0000** | 2.1940 | 5,630 | 22.70% | 17 | **1.1891e-73** | 1.3580 | 1,601 | 2,383 | 15.13% |
| neurogenesis | 1 | **0.0000** | **0.0000** | 2.2714 | 4,027 | 16.23% | 18 | **1.5015e-73** | 1.4522 | 1,105 | 1,538 | 10.44% |
| regulation of signaling | 1 | **0.0000** | **0.0000** | 2.0223 | 6,271 | 25.28% | 19 | **1.3925e-72** | 1.3059 | 2,008 | 3,108 | 18.97% |
| regulation of cell communication | 1 | **0.0000** | **0.0000** | 2.0176 | 6,220 | 25.08% | 20 | **3.0463e-72** | 1.3064 | 1,994 | 3,085 | 18.84% |
| regulation of multicellular organismal development | 1 | **0.0000** | **0.0000** | 2.2143 | 4,417 | 17.81% | 21 | **4.3188e-72** | 1.4129 | 1,254 | 1,794 | 11.85% |
| positive regulation of metabolic process | 1 | **0.0000** | **0.0000** | 2.0810 | 6,355 | 25.62% | 23 | **1.9676e-67** | 1.2950 | 1,990 | 3,106 | 18.80% |
| generation of neurons | 1 | **0.0000** | **0.0000** | 2.2925 | 3,805 | 15.34% | 24 | **6.1038e-67** | 1.4507 | 1,017 | 1,417 | 9.61% |
| animal organ morphogenesis | 1 | **0.0000** | **0.0000** | 2.6123 | 3,002 | 12.10% | 26 | **6.7155e-66** | 1.5443 | 735 | 962 | 6.94% |

The test set of 24,805 genomic regions picked 10,585 (49%) of all 21,395 genes.
*GO Biological Process* has 13,090 terms covering 17,925 (84%) of all 21,395 genes, and 1,163,819 term - gene associations.
13,090 ontology terms (100%) were tested using an annotation count range of [1, Inf].

## ⊖ GO Cellular Component (20+ terms)

Global controls

*Table controls:* Export  Shown top rows in this table: 20 Set  Term annotation count: Min: 1 Max: Inf Set  Visualize this table: [select one]

| Term Name | Binom Rank | Binom Raw P-Value | *Binom FDR Q-Val* | Binom Fold Enrichment | Binom Observed Region Hits | Binom Region Set Coverage | Hyper Rank | *Hyper FDR Q-Val* | Hyper Fold Enrichment | Hyper Observed Gene Hits | Hyper Total Genes | Hyper Gene Set Coverage |
|---|---|---|---|---|---|---|---|---|---|---|---|---|
| anchoring junction | 44 | **1.1968e-173** | **4.6077e-172** | 2.3923 | 1,316 | 5.31% | 30 | **4.3661e-22** | 1.4327 | 370 | 522 | 3.50% |
| adherens junction | 46 | **3.8450e-162** | **1.4160e-160** | 2.3630 | 1,260 | 5.08% | 32 | **7.0055e-20** | 1.4185 | 353 | 503 | 3.33% |
| transcription factor complex | 48 | **7.2550e-142** | **2.5604e-140** | 2.4867 | 1,002 | 4.04% | 72 | **7.8103e-11** | 1.3566 | 249 | 371 | 2.35% |
| cell-cell junction | 51 | **1.2686e-134** | **4.2137e-133** | 2.2404 | 1,169 | 4.71% | 31 | **2.6072e-21** | 1.4532 | 330 | 459 | 3.12% |
| actin cytoskeleton | 59 | **1.0631e-124** | **3.0523e-123** | 2.2776 | 1,047 | 4.22% | 33 | **1.3543e-19** | 1.4386 | 321 | 451 | 3.03% |
| focal adhesion | 60 | **2.4568e-122** | **6.9363e-121** | 2.3881 | 934 | 3.77% | 62 | **3.4409e-12** | 1.3719 | 262 | 386 | 2.48% |
| cell-substrate adherens junction | 63 | **7.6843e-121** | **2.0662e-119** | 2.3706 | 936 | 3.77% | 66 | **6.6771e-12** | 1.3647 | 264 | 391 | 2.49% |
| cell-substrate junction | 64 | **1.6481e-119** | **4.3623e-118** | 2.3486 | 943 | 3.80% | 65 | **6.3816e-12** | 1.3628 | 267 | 396 | 2.52% |
| cell body | 70 | **3.1652e-100** | **7.6597e-99** | 2.0183 | 1,107 | 4.46% | 49 | **8.4962e-16** | 1.3671 | 349 | 516 | 3.30% |
| extracellular matrix | 71 | **9.1001e-99** | **2.1712e-97** | 2.0050 | 1,109 | 4.47% | 50 | **1.2318e-15** | 1.3589 | 359 | 534 | 3.39% |
| cell leading edge | 72 | **2.8389e-95** | **6.6792e-94** | 2.1694 | 889 | 3.58% | 67 | **7.5375e-12** | 1.3771 | 248 | 364 | 2.34% |
| neuronal cell body | 76 | **1.8748e-88** | **4.1788e-87** | 2.0213 | 974 | 3.93% | 53 | **1.0770e-13** | 1.3688 | 300 | 443 | 2.83% |
| axon | 77 | **8.3800e-88** | **1.8436e-86** | 2.0045 | 987 | 3.98% | 34 | **1.4595e-19** | 1.4471 | 310 | 433 | 2.93% |

| Term Name | Binom Rank | Binom Raw P-Value | *Binom FDR Q-Val* | Binom Fold Enrichment | Binom Observed Region Hits | Binom Region Set Coverage | Hyper Rank | *Hyper FDR Q-Val* | Hyper Fold Enrichment | Hyper Observed Gene Hits | Hyper Total Genes | Hyper Gene Set Coverage |
|---|---|---|---|---|---|---|---|---|---|---|---|---|
| cell projection membrane | 83 | **1.9502e-74** | **3.9803e-73** | 2.2394 | 647 | 2.61% | 106 | **1.7782e-7** | 1.3378 | 184 | 278 | 1.74% |
| membrane raft | 88 | **2.2844e-68** | **4.3974e-67** | 2.0767 | 704 | 2.84% | 46 | **2.7629e-17** | 1.4963 | 228 | 308 | 2.15% |
| cytoplasmic region | 89 | **3.2257e-68** | **6.1397e-67** | 2.0674 | 710 | 2.86% | 102 | **1.2621e-7** | 1.3129 | 215 | 331 | 2.03% |
| cell cortex | 91 | **5.2806e-67** | **9.8300e-66** | 2.1655 | 626 | 2.52% | 121 | **2.3952e-6** | 1.3127 | 176 | 271 | 1.66% |
| extrinsic component of membrane | 98 | **1.2844e-60** | **2.2201e-59** | 2.1254 | 590 | 2.38% | 73 | **1.3188e-10** | 1.4119 | 190 | 272 | 1.79% |
| ruffle | 99 | **1.9145e-59** | **3.2760e-58** | 2.4261 | 437 | 1.76% | 148 | **4.7796e-4** | 1.3144 | 106 | 163 | 1.00% |
| apical plasma membrane | 100 | **2.0046e-59** | **3.3957e-58** | 2.0117 | 660 | 2.66% | 57 | **6.0984e-13** | 1.4351 | 213 | 300 | 2.01% |

The test set of 24,805 genomic regions picked 10,585 (49%) of all 21,395 genes.
*GO Cellular Component* has 1,694 terms covering 19,074 (89%) of all 21,395 genes, and 371,380 term - gene associations.
1,694 ontology terms (100%) were tested using an annotation count range of [1, Inf].

## ⊖ GO Molecular Function (20+ terms)

Global controls

*Table controls:* Export Shown top rows in this table: 20 Set Term annotation count: Min: 1 Max: Inf Set Visualize this table: [select one]

| Term Name | Binom Rank | Binom Raw P-Value | *Binom FDR Q-Val* | Binom Fold Enrichment | Binom Observed Region Hits | Binom Region Set Coverage | Hyper Rank | *Hyper FDR Q-Val* | Hyper Fold Enrichment | Hyper Observed Gene Hits | Hyper Total Genes | Hyper Gene Set Coverage |
|---|---|---|---|---|---|---|---|---|---|---|---|---|
| sequence-specific DNA binding | 1 | **0.0000** | **0.0000** | 2.7571 | 3,118 | 12.57% | 3 | **3.3153e-49** | 1.4516 | 767 | 1,068 | 7.25% |
| RNA polymerase II transcription factor activity, sequence-specific DNA binding | 1 | **0.0000** | **0.0000** | 2.7562 | 2,390 | 9.64% | 4 | **3.2749e-48** | 1.5278 | 579 | 766 | 5.47% |
| transcription regulatory region DNA binding | 1 | **0.0000** | **0.0000** | 2.7293 | 2,648 | 10.68% | 5 | **2.5564e-46** | 1.4797 | 653 | 892 | 6.17% |
| regulatory region DNA binding | 1 | **0.0000** | **0.0000** | 2.7281 | 2,650 | 10.68% | 6 | **2.7101e-46** | 1.4786 | 654 | 894 | 6.18% |
| RNA polymerase II regulatory region DNA binding | 1 | **0.0000** | **0.0000** | 2.8352 | 2,072 | 8.35% | 7 | **4.6393e-46** | 1.5550 | 507 | 659 | 4.79% |
| RNA polymerase II regulatory region sequence-specific DNA binding | 1 | **0.0000** | **0.0000** | 2.8489 | 2,070 | 8.35% | 8 | **4.5715e-46** | 1.5560 | 505 | 656 | 4.77% |
| transcription regulatory region sequence-specific DNA binding | 1 | **0.0000** | **0.0000** | 2.7753 | 2,263 | 9.12% | 9 | **2.6241e-45** | 1.5194 | 557 | 741 | 5.26% |
| sequence-specific double-stranded DNA binding | 1 | **0.0000** | **0.0000** | 2.7241 | 2,303 | 9.28% | 10 | **2.0639e-41** | 1.4866 | 570 | 775 | 5.38% |
| double-stranded DNA binding | 1 | **0.0000** | **0.0000** | 2.6378 | 2,373 | 9.57% | 12 | **1.3160e-36** | 1.4380 | 604 | 849 | 5.71% |
| transcription factor activity, sequence-specific DNA binding | 1 | **0.0000** | **0.0000** | 2.4487 | 2,905 | 11.71% | 18 | **1.7001e-26** | 1.3190 | 755 | 1,157 | 7.13% |
| enzyme binding | 1 | **0.0000** | **0.0000** | 2.0282 | 3,605 | 14.53% | 19 | **1.9478e-26** | 1.2430 | 1,182 | 1,922 | 11.17% |
| DNA binding | 1 | **0.0000** | **0.0000** | 2.2651 | 4,365 | 17.60% | 22 | **1.0928e-23** | 1.2187 | 1,271 | 2,108 | 12.01% |
| macromolecular complex binding | 19 | **2.7995e-315** | **6.0866e-313** | 2.1242 | 2,971 | 11.98% | 27 | **5.5151e-19** | 1.2373 | 906 | 1,480 | 8.56% |

| Term Name | Binom Rank | Binom Raw P-Value | *Binom FDR Q-Val* | Binom Fold Enrichment | Binom Observed Region Hits | Binom Region Set Coverage | Hyper Rank | *Hyper FDR Q-Val* | Hyper Fold Enrichment | Hyper Observed Gene Hits | Hyper Total Genes | Hyper Gene Set Coverage |
|---|---|---|---|---|---|---|---|---|---|---|---|---|
| transcription factor activity, RNA polymerase II core promoter proximal region sequence-specific binding | 23 | **2.6800e-264** | **4.8136e-262** | 2.8965 | 1,430 | 5.76% | 13 | **8.0953e-35** | 1.6018 | 336 | 424 | 3.17% |
| transcriptional activator activity, RNA polymerase II transcription regulatory region sequence-specific binding | 24 | **2.9772e-245** | **5.1245e-243** | 2.8222 | 1,384 | 5.58% | 11 | **3.6409e-37** | 1.6247 | 336 | 418 | 3.17% |
| core promoter proximal region DNA binding | 26 | **3.5868e-222** | **5.6989e-220** | 2.7823 | 1,285 | 5.18% | 15 | **3.5019e-28** | 1.5556 | 314 | 408 | 2.97% |
| core promoter proximal region sequence-specific DNA binding | 27 | **1.2043e-216** | **1.8426e-214** | 2.7681 | 1,264 | 5.10% | 16 | **1.2785e-27** | 1.5521 | 311 | 405 | 2.94% |
| RNA polymerase II core promoter proximal region sequence-specific DNA binding | 28 | **2.7077e-216** | **3.9948e-214** | 2.8013 | 1,238 | 4.99% | 14 | **7.8696e-29** | 1.5727 | 305 | 392 | 2.88% |
| protein domain specific binding | 33 | **3.7764e-196** | **4.7274e-194** | 2.2821 | 1,627 | 6.56% | 25 | **7.3318e-20** | 1.3551 | 478 | 713 | 4.52% |
| transcription factor binding | 34 | **1.7449e-190** | **2.1201e-188** | 2.4270 | 1,402 | 5.65% | 34 | **1.5462e-14** | 1.3439 | 373 | 561 | 3.52% |

The test set of 24,805 genomic regions picked 10,585 (49%) of all 21,395 genes.
*GO Molecular Function* has 4,131 terms covering 17,189 (80%) of all 21,395 genes, and 227,341 term - gene associations.
4,131 ontology terms (100%) were tested using an annotation count range of [1, Inf].

## Human Phenotype (20+ terms)

Global controls

*Table controls:* Export Shown top rows in this table: 20 Set Term annotation count: Min: 1 Max: Inf Set Visualize this table: [select one]

| Term Name | Binom Rank | Binom Raw P-Value | *Binom FDR Q-Val* | Binom Fold Enrichment | Binom Observed Region Hits | Binom Region Set Coverage | Hyper Rank | *Hyper FDR Q-Val* | Hyper Fold Enrichment | Hyper Observed Gene Hits | Hyper Total Genes | Hyper Gene Set Coverage |
|---|---|---|---|---|---|---|---|---|---|---|---|---|
| Autosomal dominant inheritance | 1 | **2.8691e-313** | **1.8933e-309** | 2.1284 | 2,941 | 11.86% | 1 | **5.8139e-42** | 1.3812 | 889 | 1,301 | 8.40% |
| Abnormality of the integument | 9 | **6.5918e-202** | **4.8333e-199** | 2.0164 | 2,195 | 8.85% | 10 | **5.1793e-15** | 1.2523 | 684 | 1,104 | 6.46% |
| Abnormality of the mouth | 11 | **4.4709e-190** | **2.6822e-187** | 2.0715 | 1,945 | 7.84% | 9 | **1.8223e-15** | 1.2852 | 571 | 898 | 5.39% |
| Abnormality of the oral cavity | 16 | **3.6164e-175** | **1.4915e-172** | 2.1466 | 1,659 | 6.69% | 11 | **1.3645e-14** | 1.3079 | 473 | 731 | 4.47% |
| Abnormality of the orbital region | 21 | **2.9844e-158** | **9.3783e-156** | 2.0143 | 1,741 | 7.02% | 15 | **4.9969e-12** | 1.2639 | 514 | 822 | 4.86% |
| Abnormal appendicular skeleton morphology | 26 | **1.2439e-140** | **3.1571e-138** | 2.0014 | 1,577 | 6.36% | 22 | **3.3669e-10** | 1.2518 | 475 | 767 | 4.49% |
| Abnormality of cardiovascular system morphology | 27 | **1.2376e-131** | **3.0249e-129** | 2.0330 | 1,423 | 5.74% | 46 | **3.5994e-8** | 1.2307 | 439 | 721 | 4.15% |
| Abnormality of the nose | 31 | **2.9773e-130** | **6.3379e-128** | 2.0151 | 1,439 | 5.80% | 51 | **7.4452e-8** | 1.2302 | 423 | 695 | 4.00% |
| Abnormality of digit | 34 | **1.1253e-123** | **2.1840e-121** | 2.0481 | 1,315 | 5.30% | 27 | **2.4949e-9** | 1.2725 | 379 | 602 | 3.58% |
| Abnormality of skin adnexa morphology | 35 | **3.5042e-121** | **6.6070e-119** | 2.0636 | 1,266 | 5.10% | 56 | **2.0062e-7** | 1.2410 | 369 | 601 | 3.49% |
| Abnormality of the palate | 36 | **1.3281e-119** | **2.4345e-117** | 2.2716 | 1,010 | 4.07% | 25 | **1.5270e-9** | 1.3344 | 272 | 412 | 2.57% |

| Term Name | Binom Rank | Binom Raw P-Value | *Binom FDR Q-Val* | Binom Fold Enrichment | Binom Observed Region Hits | Binom Region Set Coverage | Hyper Rank | *Hyper FDR Q-Val* | Hyper Fold Enrichment | Hyper Observed Gene Hits | Hyper Total Genes | Hyper Gene Set Coverage |
|---|---|---|---|---|---|---|---|---|---|---|---|---|
| Aplasia/hypoplasia affecting bones of the axial skeleton | 40 | **2.2350e-117** | **3.6871e-115** | 2.3967 | 890 | 3.59% | 47 | **4.1593e-8** | 1.3270 | 237 | 361 | 2.24% |
| Abnormal morphology of the ocular adnexa | 41 | **3.2782e-116** | **5.2763e-114** | 2.0391 | 1,250 | 5.04% | 30 | **3.3823e-9** | 1.2732 | 371 | 589 | 3.50% |
| Abnormal heart morphology | 43 | **7.2568e-116** | **1.1137e-113** | 2.0588 | 1,218 | 4.91% | 74 | **9.4583e-7** | 1.2259 | 373 | 615 | 3.52% |
| Abnormality of the dentition | 45 | **2.5972e-115** | **3.8086e-113** | 2.2898 | 958 | 3.86% | 62 | **4.4710e-7** | 1.3017 | 237 | 368 | 2.24% |
| Abnormality of the gastrointestinal tract | 46 | **3.8034e-115** | **5.4562e-113** | 2.0715 | 1,193 | 4.81% | 34 | **6.5531e-9** | 1.2770 | 350 | 554 | 3.31% |
| Abnormality of connective tissue | 47 | **7.4816e-115** | **1.0504e-112** | 2.0804 | 1,178 | 4.75% | 23 | **4.6271e-10** | 1.2935 | 359 | 561 | 3.39% |
| Abnormality of the vasculature | 50 | **8.5108e-110** | **1.1233e-107** | 2.0841 | 1,122 | 4.52% | 40 | **1.9764e-8** | 1.2702 | 345 | 549 | 3.26% |
| Aplasia/hypoplasia involving the skeleton | 52 | **8.8742e-109** | **1.1262e-106** | 2.0871 | 1,108 | 4.47% | 48 | **6.0122e-8** | 1.2712 | 322 | 512 | 3.04% |
| Abnormal reproductive system morphology | 54 | **3.4735e-107** | **4.2448e-105** | 2.0163 | 1,186 | 4.78% | 149 | **5.9925e-5** | 1.2044 | 317 | 532 | 2.99% |

The test set of 24,805 genomic regions picked 10,585 (49%) of all 21,395 genes.
*Human Phenotype* has 6,599 terms covering 3,215 (15%) of all 21,395 genes, and 244,972 term - gene associations.
6,599 ontology terms (100%) were tested using an annotation count range of [1, Inf].

## ⊖ Mouse Phenotype Single KO (20+ terms)

Global controls

*Table controls:* Export Shown top rows in this table: 20 Set Term annotation count: Min: 1 Max: Inf Set Visualize this table: [select one]

| Term Name | Binom Rank | Binom Raw P-Value | *Binom FDR Q-Val* | Binom Fold Enrichment | Binom Observed Region Hits | Binom Region Set Coverage | Hyper Rank | *Hyper FDR Q-Val* | Hyper Fold Enrichment | Hyper Observed Gene Hits | Hyper Total Genes | Hyper Gene Set Coverage |
|---|---|---|---|---|---|---|---|---|---|---|---|---|
| nervous system phenotype | 1 | **0.0000** | **0.0000** | 2.0198 | 6,250 | 25.20% | 1 | **2.4017e-99** | 1.3777 | 1,933 | 2,836 | 18.26% |
| lethality during fetal growth through weaning | 1 | **0.0000** | **0.0000** | 2.2636 | 4,591 | 18.51% | 6 | **4.0579e-81** | 1.4424 | 1,256 | 1,760 | 11.87% |
| preweaning lethality | 1 | **0.0000** | **0.0000** | 2.0297 | 7,228 | 29.14% | 8 | **8.4757e-80** | 1.2957 | 2,291 | 3,574 | 21.64% |
| abnormal nervous system morphology | 1 | **0.0000** | **0.0000** | 2.1284 | 5,237 | 21.11% | 9 | **7.1345e-79** | 1.3841 | 1,527 | 2,230 | 14.43% |
| cardiovascular system phenotype | 1 | **0.0000** | **0.0000** | 2.0038 | 4,855 | 19.57% | 12 | **5.1338e-65** | 1.3469 | 1,506 | 2,260 | 14.23% |
| skeleton phenotype | 1 | **0.0000** | **0.0000** | 2.0889 | 4,309 | 17.37% | 14 | **5.4533e-58** | 1.3576 | 1,295 | 1,928 | 12.23% |
| abnormal skeleton morphology | 1 | **0.0000** | **0.0000** | 2.1029 | 4,150 | 16.73% | 18 | **4.5641e-55** | 1.3582 | 1,231 | 1,832 | 11.63% |
| perinatal lethality | 1 | **0.0000** | **0.0000** | 2.3825 | 2,795 | 11.27% | 21 | **3.3852e-54** | 1.4908 | 725 | 983 | 6.85% |
| abnormal brain morphology | 1 | **0.0000** | **0.0000** | 2.1793 | 3,392 | 13.67% | 27 | **5.4929e-50** | 1.3996 | 941 | 1,359 | 8.89% |
| abnormal cardiovascular system morphology | 1 | **0.0000** | **0.0000** | 2.0816 | 3,851 | 15.53% | 30 | **1.1038e-47** | 1.3459 | 1,138 | 1,709 | 10.75% |
| abnormal axial skeleton morphology | 1 | **0.0000** | **0.0000** | 2.2849 | 2,936 | 11.84% | 39 | **2.1698e-41** | 1.3958 | 799 | 1,157 | 7.55% |
| vision/eye phenotype | 1 | **0.0000** | **0.0000** | 2.2103 | 3,254 | 13.12% | 41 | **5.1436e-41** | 1.3624 | 916 | 1,359 | 8.65% |
| abnormal eye morphology | 1 | **0.0000** | **0.0000** | 2.2631 | 3,093 | 12.47% | 42 | **5.3812e-39** | 1.3690 | 848 | 1,252 | 8.01% |
| embryo phenotype | 1 | **0.0000** | **0.0000** | 2.1980 | 3,554 | 14.33% | 66 | **1.1045e-28** | 1.2838 | 980 | 1,543 | 9.26% |

| Term Name | Binom Rank | Binom Raw P-Value | *Binom FDR Q-Val* | Binom Fold Enrichment | Binom Observed Region Hits | Binom Region Set Coverage | Hyper Rank | *Hyper FDR Q-Val* | Hyper Fold Enrichment | Hyper Observed Gene Hits | Hyper Total Genes | Hyper Gene Set Coverage |
|---|---|---|---|---|---|---|---|---|---|---|---|---|
| abnormal embryo morphology | 1 | **0.0000** | **0.0000** | 2.2514 | 2,839 | 11.45% | 98 | **5.3992e-24** | 1.2997 | 753 | 1,171 | 7.11% |
| craniofacial phenotype | 37 | **1.0672e-321** | **2.6449e-319** | 2.2965 | 2,582 | 10.41% | 50 | **2.7947e-37** | 1.4134 | 672 | 961 | 6.35% |
| abnormal craniofacial morphology | 38 | **6.4130e-321** | **1.5476e-318** | 2.2966 | 2,576 | 10.39% | 52 | **4.7118e-37** | 1.4130 | 669 | 957 | 6.32% |
| abnormal nervous system development | 41 | **4.5886e-309** | **1.0263e-306** | 2.2738 | 2,535 | 10.22% | 46 | **1.2492e-37** | 1.4166 | 670 | 956 | 6.33% |
| digestive/alimentary phenotype | 42 | **8.2277e-308** | **1.7964e-305** | 2.2508 | 2,577 | 10.39% | 53 | **5.4517e-37** | 1.3921 | 728 | 1,057 | 6.88% |
| integument phenotype | 43 | **5.2598e-304** | **1.1217e-301** | 2.0038 | 3,270 | 13.18% | 40 | **4.1423e-41** | 1.3401 | 1,019 | 1,537 | 9.63% |

The test set of 24,805 genomic regions picked 10,585 (49%) of all 21,395 genes.
*Mouse Phenotype Single KO* has 9,170 terms covering 9,466 (44%) of all 21,395 genes, and 551,620 term - gene associations.
9,170 ontology terms (100%) were tested using an annotation count range of [1, Inf].

## Mouse Phenotype (20+ terms)

Global controls

*Table controls:* Export | Shown top rows in this table: 20 Set | Term annotation count: Min: 1 Max: Inf Set | Visualize this table: [select one]

| Term Name | Binom Rank | Binom Raw P-Value | *Binom FDR Q-Val* | Binom Fold Enrichment | Binom Observed Region Hits | Binom Region Set Coverage | Hyper Rank | *Hyper FDR Q-Val* | Hyper Fold Enrichment | Hyper Observed Gene Hits | Hyper Total Genes | Hyper Gene Set Coverage |
|---|---|---|---|---|---|---|---|---|---|---|---|---|
| nervous system phenotype | 1 | **0.0000** | **0.0000** | 2.0178 | 7,247 | 29.22% | 1 | **5.4564e-118** | 1.3728 | 2,278 | 3,354 | 21.52% |
| preweaning lethality | 1 | **0.0000** | **0.0000** | 2.0169 | 8,080 | 32.57% | 6 | **6.3495e-98** | 1.3034 | 2,611 | 4,049 | 24.67% |
| abnormal nervous system morphology | 1 | **0.0000** | **0.0000** | 2.1077 | 6,259 | 25.23% | 7 | **2.5688e-97** | 1.3800 | 1,868 | 2,736 | 17.65% |
| lethality during fetal growth through weaning | 1 | **0.0000** | **0.0000** | 2.1916 | 5,683 | 22.91% | 9 | **3.9913e-96** | 1.4142 | 1,619 | 2,314 | 15.30% |
| cardiovascular system phenotype | 1 | **0.0000** | **0.0000** | 2.0191 | 5,626 | 22.68% | 12 | **2.1219e-82** | 1.3588 | 1,760 | 2,618 | 16.63% |
| skeleton phenotype | 1 | **0.0000** | **0.0000** | 2.0935 | 4,916 | 19.82% | 14 | **5.7776e-73** | 1.3716 | 1,497 | 2,206 | 14.14% |
| perinatal lethality | 1 | **0.0000** | **0.0000** | 2.3141 | 3,636 | 14.66% | 15 | **1.0290e-70** | 1.4760 | 980 | 1,342 | 9.26% |
| abnormal skeleton morphology | 1 | **0.0000** | **0.0000** | 2.1000 | 4,721 | 19.03% | 19 | **1.3892e-68** | 1.3707 | 1,420 | 2,094 | 13.42% |
| abnormal cardiovascular system morphology | 1 | **0.0000** | **0.0000** | 2.1081 | 4,671 | 18.83% | 22 | **4.3602e-65** | 1.3645 | 1,390 | 2,059 | 13.13% |
| abnormal brain morphology | 1 | **0.0000** | **0.0000** | 2.1813 | 4,221 | 17.02% | 24 | **9.8089e-64** | 1.3973 | 1,189 | 1,720 | 11.23% |
| muscle phenotype | 1 | **0.0000** | **0.0000** | 2.1167 | 3,461 | 13.95% | 25 | **1.5579e-61** | 1.4261 | 1,028 | 1,457 | 9.71% |
| abnormal neuron morphology | 1 | **0.0000** | **0.0000** | 2.1135 | 3,696 | 14.90% | 30 | **5.5625e-58** | 1.3999 | 1,077 | 1,555 | 10.17% |
| neonatal lethality | 1 | **0.0000** | **0.0000** | 2.3326 | 2,770 | 11.17% | 32 | **5.3319e-57** | 1.4996 | 736 | 992 | 6.95% |
| abnormal cardiovascular system physiology | 1 | **0.0000** | **0.0000** | 2.0101 | 3,632 | 14.64% | 34 | **1.4099e-55** | 1.3813 | 1,118 | 1,636 | 10.56% |
| vision/eye phenotype | 1 | **0.0000** | **0.0000** | 2.2196 | 3,859 | 15.56% | 35 | **1.6325e-54** | 1.3830 | 1,090 | 1,593 | 10.30% |
| abnormal eye morphology | 1 | **0.0000** | **0.0000** | 2.2657 | 3,675 | 14.82% | 37 | **1.1838e-52** | 1.3911 | 1,020 | 1,482 | 9.64% |
| abnormal axial skeleton morphology | 1 | **0.0000** | **0.0000** | 2.2751 | 3,411 | 13.75% | 39 | **3.9310e-51** | 1.4036 | 943 | 1,358 | 8.91% |
| digestive/alimentary phenotype | 1 | **0.0000** | **0.0000** | 2.2263 | 3,300 | 13.30% | 40 | **2.4669e-50** | 1.3961 | 958 | 1,387 | 9.05% |

| Term Name | Binom Rank | Binom Raw P-Value | *Binom FDR Q-Val* | Binom Fold Enrichment | Binom Observed Region Hits | Binom Region Set Coverage | Hyper Rank | *Hyper FDR Q-Val* | Hyper Fold Enrichment | Hyper Observed Gene Hits | Hyper Total Genes | Hyper Gene Set Coverage |
|---|---|---|---|---|---|---|---|---|---|---|---|---|
| **abnormal heart morphology** | 1 | **0.0000** | **0.0000** | 2.1154 | 3,401 | 13.71% | 45 | **8.8750e-49** | 1.3771 | 1,007 | 1,478 | 9.51% |
| **postnatal lethality** | 1 | **0.0000** | **0.0000** | 2.1099 | 3,290 | 13.26% | 47 | **1.5822e-48** | 1.3906 | 946 | 1,375 | 8.94% |

The test set of 24,805 genomic regions picked 10,585 (49%) of all 21,395 genes.
*Mouse Phenotype* has 9,575 terms covering 9,654 (45%) of all 21,395 genes, and 705,265 term - gene associations.
9,575 ontology terms (100%) were tested using an annotation count range of [1, Inf].
