# Supplementary figures and images for: AP-2α and AP-2β cooperatively function in the craniofacial surface ectoderm to regulate chromatin and gene expression dynamics during facial development
Source: eLife. 2022 Mar 25;11:e70511. doi: 10.7554/eLife.70511 (PMC9038197; doi:10.7554/eLife.70511)

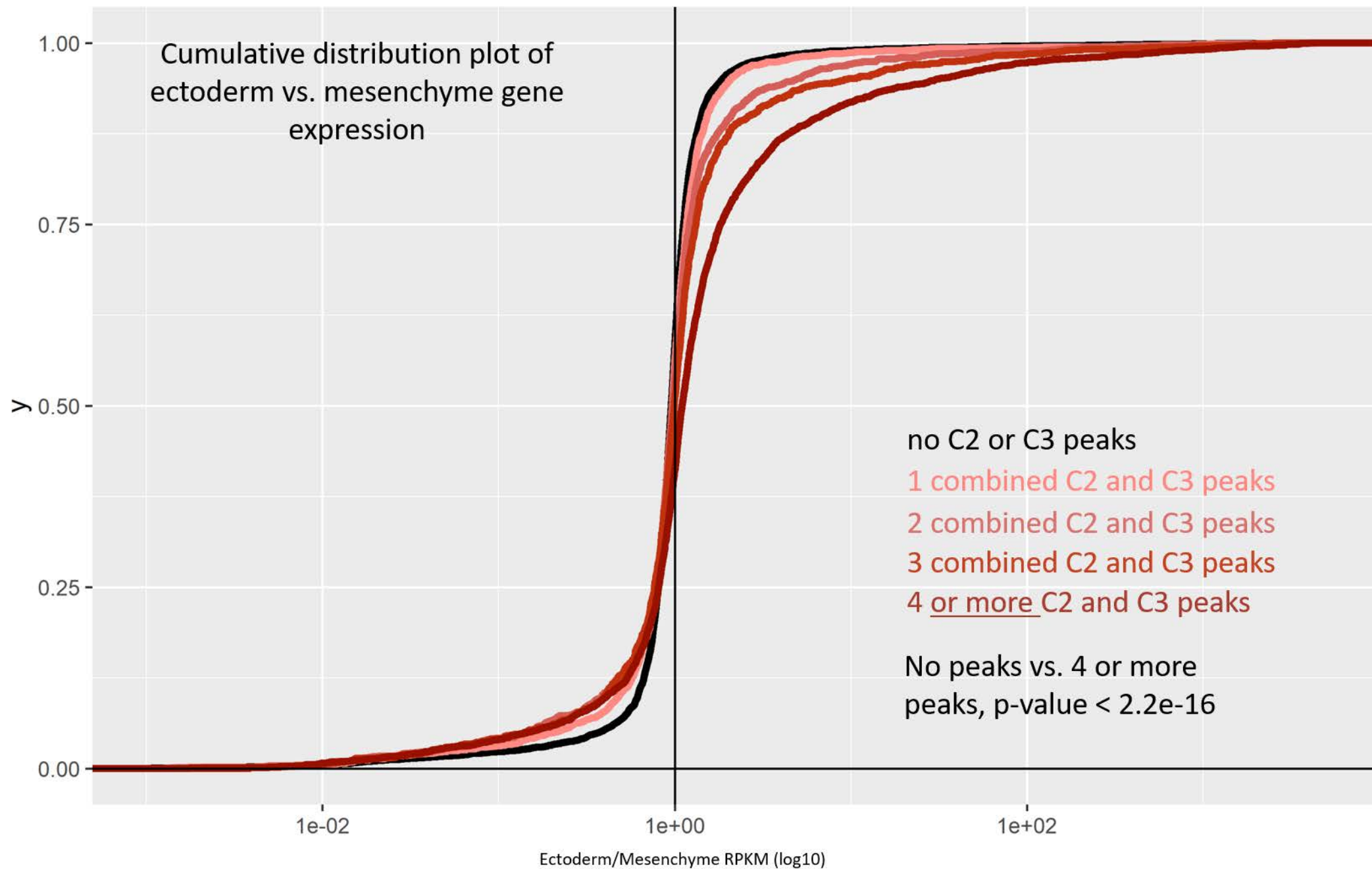

Supplement: Figure 2—source data 11. [file elife-70511-fig2-data11.pdf]
