# Supplementary material for: AP-2α and AP-2β cooperatively function in the craniofacial surface ectoderm to regulate chromatin and gene expression dynamics during facial development
Source: eLife. 2022 Mar 25;11:e70511. doi: 10.7554/eLife.70511 (PMC9038197; doi:10.7554/eLife.70511)
Supplement: Figure 3—source data 1. [file elife-70511-fig3-data1.pdf]

Homer Known Motif Enrichment Results (/Users/vanottee/output/)

Homer *de novo* Motif Results
Gene Ontology Enrichment Results
Known Motif Enrichment Results (txt file)
Total Target Sequences = 63641, Total Background Sequences = 62370

Rank	Motif	Name	P-value	log P-pvalue	q-value (Benjamini)	# Target Sequences with Motif	% of Targets Sequences with Motif	# Background Sequences with Motif	% of Background Sequences with Motif	Motif File	SVG
1	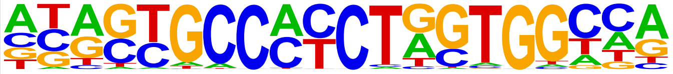	CTCF(Zf)/CD4+-CTCF-ChIP-Seq(Barski_et_al.)/Homer	1e-1933	-4.451e+03	0.0000	10558.0	16.59%	3711.0	5.95%	motif file (matrix)	svg
2	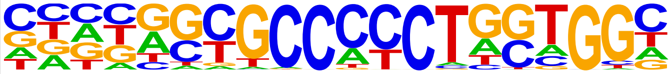	BORIS(Zf)/K562-CTCFL-ChIP-Seq(GSE32465)/Homer	1e-1518	-3.497e+03	0.0000	13347.0	20.97%	6116.4	9.81%	motif file (matrix)	svg
3	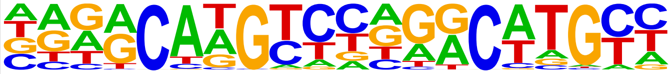	p73(p53)/Trachea-p73-ChIP-Seq(PRJNA310161)/Homer	1e-961	-2.215e+03	0.0000	3900.0	6.13%	1073.3	1.72%	motif file (matrix)	svg
4	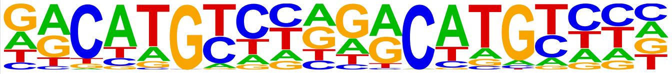	p53(p53)/Saos-p53-ChIP-Seq(GSE15780)/Homer	1e-828	-1.908e+03	0.0000	5646.0	8.87%	2197.1	3.53%	motif file (matrix)	svg
5	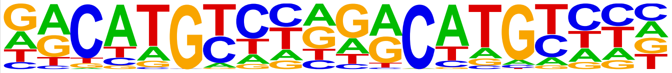	p53(p53)/Saos-p53-ChIP-Seq/Homer	1e-828	-1.908e+03	0.0000	5646.0	8.87%	2197.1	3.53%	motif file (matrix)	svg
6	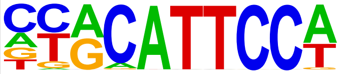	TEAD1(TEAD)/HepG2-TEAD1-ChIP-Seq(Encode)/Homer	1e-765	-1.763e+03	0.0000	25316.0	39.77%	17944.7	28.79%	motif file (matrix)	svg
7	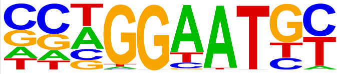	TEAD4(TEA)/Tropoblast-Tead4-ChIP-Seq(GSE37350)/Homer	1e-732	-1.686e+03	0.0000	23485.0	36.89%	16453.3	26.40%	motif file (matrix)	svg
8	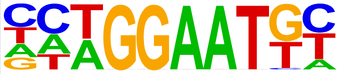	TEAD(TEA)/Fibroblast-PU.1-ChIP-Seq(Unpublished)/Homer	1e-682	-1.571e+03	0.0000	18212.0	28.61%	12081.8	19.38%	motif file (matrix)	svg
9	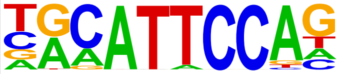	TEAD3(TEA)/HepG2-TEAD3-ChIP-Seq(Encode)/Homer	1e-615	-1.418e+03	0.0000	27633.0	43.41%	20736.9	33.27%	motif file (matrix)	svg
10	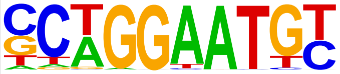	TEAD2(TEA)/Py2T-Tead2-ChIP-Seq(GSE55709)/Homer	1e-612	-1.411e+03	0.0000	15780.0	24.79%	10302.2	16.53%	motif file (matrix)	svg
11	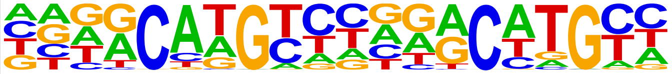	p63(p53)/Keratinocyte-p63-ChIP-Seq(GSE17611)/Homer	1e-574	-1.322e+03	0.0000	13498.0	21.20%	8563.2	13.74%	motif file (matrix)	svg
12	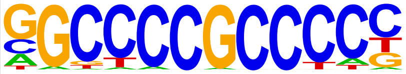	Sp1(Zf)/Promoter/Homer	1e-417	-9.620e+02	0.0000	12415.0	19.50%	8273.4	13.27%	motif file (matrix)	svg
13	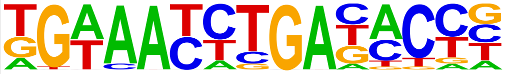	Six4(Homeobox)/MCF7-SIX4-ChIP-Seq(Encode)/Homer	1e-336	-7.744e+02	0.0000	2371.0	3.72%	926.1	1.49%	motif file (matrix)	svg
14	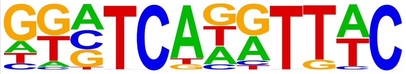	Six1(Homeobox)/Myoblast-Six1-ChIP-Chip(GSE20150)/Homer	1e-287	-6.615e+02	0.0000	7379.0	11.59%	4690.5	7.53%	motif file (matrix)	svg
15	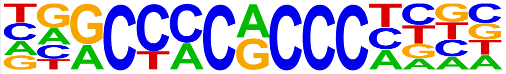	KLF3(Zf)/MEF-Klf3-ChIP-Seq(GSE44748)/Homer	1e-262	-6.053e+02	0.0000	18778.0	29.50%	14657.0	23.52%	motif file (matrix)	svg

16	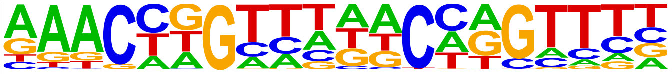	GRHL2(CP2)/HBE-GRHL2-ChIP-Seq(GSE46194)/Homer	1e-227	-5.229e+02	0.0000	10765.0	16.91%	7788.2	12.50%	motif file (matrix)	svg
17	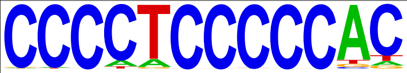	Zfp281(Zf)/ES-Zfp281-ChIP-Seq(GSE81042)/Homer	1e-199	-4.594e+02	0.0000	10803.0	16.97%	7981.4	12.81%	motif file (matrix)	svg
18	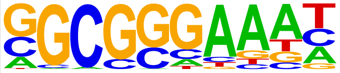	E2F4(E2F)/K562-E2F4-ChIP-Seq(GSE31477)/Homer	1e-178	-4.101e+02	0.0000	12491.0	19.62%	9601.6	15.41%	motif file (matrix)	svg
19	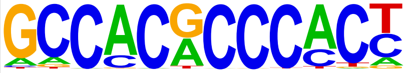	Klf9(Zf)/GBM-Klf9-ChIP-Seq(GSE62211)/Homer	1e-176	-4.062e+02	0.0000	15756.0	24.75%	12543.2	20.13%	motif file (matrix)	svg
20	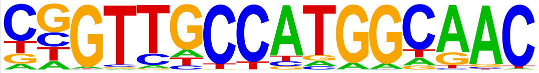	RFX(HTH)/K562-RFX3-ChIP-Seq(SRA012198)/Homer	1e-170	-3.928e+02	0.0000	3470.0	5.45%	2055.7	3.30%	motif file (matrix)	svg
21	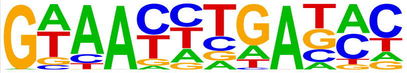	Six2(Homeobox)/NephronProgenitor-Six2-ChIP-Seq(GSE39837)/Homer	1e-160	-3.688e+02	0.0000	21978.0	34.53%	18428.8	29.57%	motif file (matrix)	svg
22	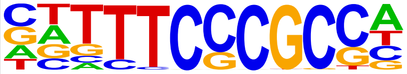	E2F7(E2F)/Hela-E2F7-ChIP-Seq(GSE32673)/Homer	1e-155	-3.584e+02	0.0000	4204.0	6.60%	2681.6	4.30%	motif file (matrix)	svg
23	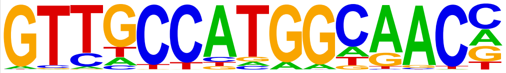	Rfx2(HTH)/LoVo-RFX2-ChIP-Seq(GSE49402)/Homer	1e-149	-3.440e+02	0.0000	3687.0	5.79%	2298.9	3.69%	motif file (matrix)	svg
24	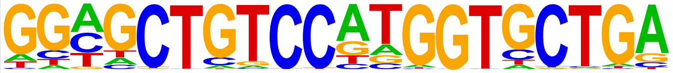	REST-NRSF(Zf)/Jurkat-NRSF-ChIP-Seq/Homer	1e-145	-3.349e+02	0.0000	683.0	1.07%	207.7	0.33%	motif file (matrix)	svg
25	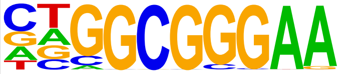	E2F1(E2F)/Hela-E2F1-ChIP-Seq(GSE22478)/Homer	1e-140	-3.234e+02	0.0000	7408.0	11.64%	5412.1	8.68%	motif file (matrix)	svg
26	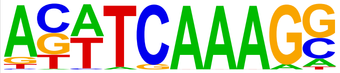	Tcf3(HMG)/mES-Tcf3-ChIP-Seq(GSE11724)/Homer	1e-132	-3.061e+02	0.0000	7032.0	11.05%	5136.4	8.24%	motif file (matrix)	svg
27	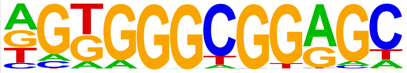	Sp5(Zf)/mES-Sp5.Flag-ChIP-Seq(GSE72989)/Homer	1e-124	-2.859e+02	0.0000	28869.0	45.35%	25368.5	40.70%	motif file (matrix)	svg
28	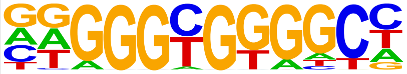	KLF1(Zf)/HUDEP2-KLF1-CutnRun(GSE136251)/Homer	1e-118	-2.720e+02	0.0000	26940.0	42.32%	23583.7	37.84%	motif file (matrix)	svg
29	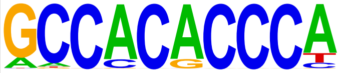	Klf4(Zf)/mES-Klf4-ChIP-Seq(GSE11431)/Homer	1e-117	-2.712e+02	0.0000	13522.0	21.24%	11007.4	17.66%	motif file (matrix)	svg
30	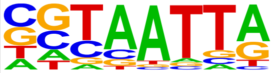	DLX5(Homeobox)/BasalGanglia-Dlx5-ChIP-seq(GSE124936)/Homer	1e-111	-2.564e+02	0.0000	14716.0	23.12%	12160.4	19.51%	motif file (matrix)	svg
31	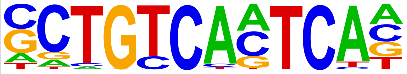	Pbx3(Homeobox)/GM12878-PBX3-ChIP-Seq(GSE32465)/Homer	1e-109	-2.522e+02	0.0000	7674.0	12.06%	5845.1	9.38%	motif file (matrix)	svg
32	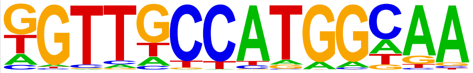	X-box(HTH)/NPC-H3K4me1-ChIP-Seq(GSE16256)/Homer	1e-109	-2.515e+02	0.0000	3585.0	5.63%	2384.1	3.83%	motif file (matrix)	svg
33	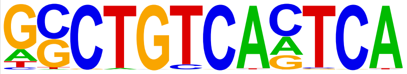	PBX1(Homeobox)/MCF7-PBX1-ChIP-Seq(GSE28007)/Homer	1e-108	-2.502e+02	0.0000	3175.0	4.99%	2057.2	3.30%	motif file (matrix)	svg
34	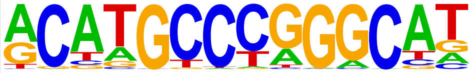	p53(p53)/mES-cMyc-ChIP-Seq(GSE11431)/Homer	1e-99	-2.294e+02	0.0000	904.0	1.42%	399.1	0.64%	motif file (matrix)	svg

35	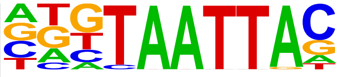	Dlx3(Homeobox)/Kerainocytes-Dlx3-ChIP-Seq(GSE89884)/Homer	1e-97	-2.244e+02	0.0000	12468.0	19.59%	10238.9	16.43%	motif file (matrix)	svg
36	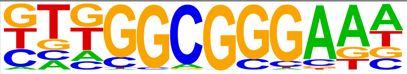	E2F3(E2F)/MEF-E2F3-ChIP-Seq(GSE71376)/Homer	1e-96	-2.215e+02	0.0000	16301.0	25.61%	13783.0	22.11%	motif file (matrix)	svg
37	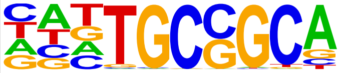	Zfp57(Zf)/H1-ZFP57.HA-ChIP-Seq(GSE115387)/Homer	1e-93	-2.160e+02	0.0000	10372.0	16.29%	8371.5	13.43%	motif file (matrix)	svg
38	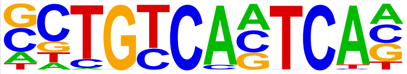	Pknox1(Homeobox)/ES-Prep1-ChIP-Seq(GSE63282)/Homer	1e-85	-1.976e+02	0.0000	7296.0	11.46%	5692.8	9.13%	motif file (matrix)	svg
39	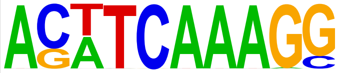	TCFL2(HMG)/K562-TCF7L2-ChIP-Seq(GSE29196)/Homer	1e-82	-1.894e+02	0.0000	2664.0	4.18%	1764.6	2.83%	motif file (matrix)	svg
40	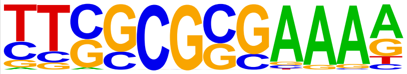	E2F(E2F)/Hela-CellCycle-Expression/Homer	1e-81	-1.883e+02	0.0000	1929.0	3.03%	1185.8	1.90%	motif file (matrix)	svg
41	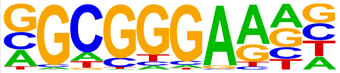	E2F6(E2F)/Hela-E2F6-ChIP-Seq(GSE31477)/Homer	1e-79	-1.840e+02	0.0000	14086.0	22.13%	11912.0	19.11%	motif file (matrix)	svg
42	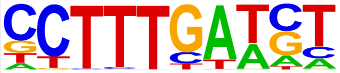	LEF1(HMG)/H1-LEF1-ChIP-Seq(GSE64758)/Homer	1e-72	-1.672e+02	0.0000	15236.0	23.93%	13069.2	20.97%	motif file (matrix)	svg
43	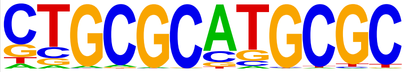	NRF1(NRF)/MCF7-NRF1-ChIP-Seq(Unpublished)/Homer	1e-70	-1.625e+02	0.0000	5247.0	8.24%	4013.4	6.44%	motif file (matrix)	svg
44	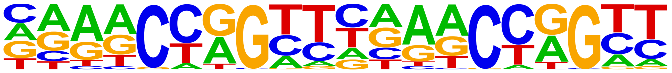	Tcfcp2l1(CP2)/mES-Tcfcp2l1-ChIP-Seq(GSE11431)/Homer	1e-67	-1.543e+02	0.0000	5066.0	7.96%	3882.1	6.23%	motif file (matrix)	svg
45	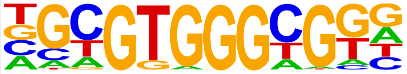	Egr2(Zf)/Thymocytes-Egr2-ChIP-Seq(GSE34254)/Homer	1e-64	-1.494e+02	0.0000	6455.0	10.14%	5123.3	8.22%	motif file (matrix)	svg
46	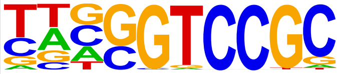	HINFP(Zf)/K562-HINFP.eGFP-ChIP-Seq(Encode)/Homer	1e-61	-1.407e+02	0.0000	8817.0	13.85%	7286.0	11.69%	motif file (matrix)	svg
47	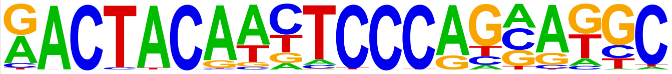	Ronin(THAP)/ES-Thap11-ChIP-Seq(GSE51522)/Homer	1e-55	-1.276e+02	0.0000	1208.0	1.90%	727.0	1.17%	motif file (matrix)	svg
48	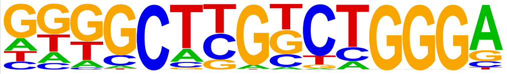	Zfp809(Zf)/ES-Zfp809-ChIP-Seq(GSE70799)/Homer	1e-53	-1.226e+02	0.0000	6726.0	10.57%	5475.8	8.79%	motif file (matrix)	svg
49	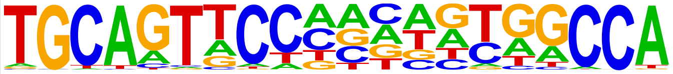	CTCF-SatelliteElement(Zf?)/CD4+-CTCF-ChIP-Seq(Barski_et_al.)/Homer	1e-45	-1.058e+02	0.0000	726.0	1.14%	398.3	0.64%	motif file (matrix)	svg
50	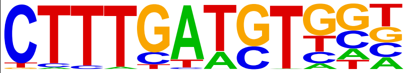	Tcf7(HMG)/GM12878-TCF7-ChIP-Seq(Encode)/Homer	1e-45	-1.055e+02	0.0000	7992.0	12.55%	6706.8	10.76%	motif file (matrix)	svg
51	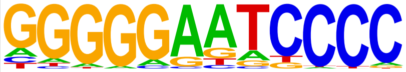	NFkB-p50,p52(RHD)/Monocyte-p50-ChIP-Chip(Schreiber_et_al.)/Homer	1e-44	-1.021e+02	0.0000	3428.0	5.39%	2630.4	4.22%	motif file (matrix)	svg
52	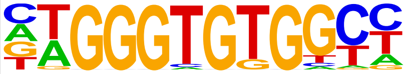	EKLF(Zf)/Erythrocyte-Klf1-ChIP-Seq(GSE20478)/Homer	1e-43	-1.006e+02	0.0000	6792.0	10.67%	5637.5	9.05%	motif file (matrix)	svg
53	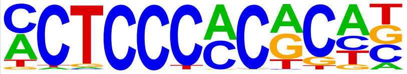	WT1(Zf)/Kidney-WT1-ChIP-Seq(GSE90016)/Homer	1e-42	-9.748e+01	0.0000	17764.0	27.91%	15901.5	25.51%	motif file (matrix)	svg

54	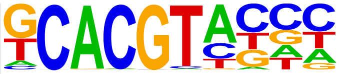	HIF2a(bHLH)/785_O-HIF2a-ChIP-Seq(GSE34871)/Homer	1e-36	-8.498e+01	0.0000	8450.0	13.27%	7242.2	11.62%	motif file (matrix)	svg
55	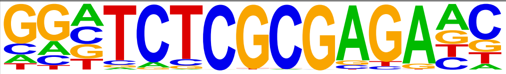	ZBTB33(Zf)/GM12878-ZBTB33-ChIP-Seq(GSE32465)/Homer	1e-36	-8.343e+01	0.0000	1411.0	2.22%	970.5	1.56%	motif file (matrix)	svg
56	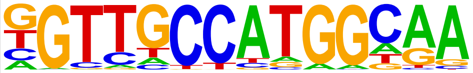	Rfx1(HTH)/NPC-H3K4me1-ChIP-Seq(GSE16256)/Homer	1e-29	-6.830e+01	0.0000	5809.0	9.13%	4911.3	7.88%	motif file (matrix)	svg
57	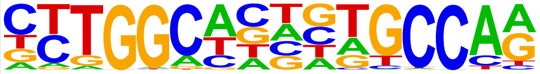	NF1(CTF)/LNCAP-NF1-ChIP-Seq(Unpublished)/Homer	1e-28	-6.639e+01	0.0000	10438.0	16.40%	9220.0	14.79%	motif file (matrix)	svg
58	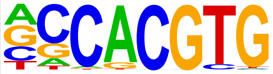	c-Myc(bHLH)/LNCAP-cMyc-ChIP-Seq(Unpublished)/Homer	1e-28	-6.638e+01	0.0000	11540.0	18.13%	10256.5	16.46%	motif file (matrix)	svg
59	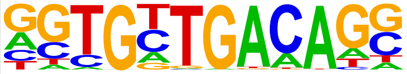	Tbx20(T-box)/Heart-Tbx20-ChIP-Seq(GSE29636)/Homer	1e-25	-5.904e+01	0.0000	5917.0	9.29%	5065.1	8.13%	motif file (matrix)	svg
60	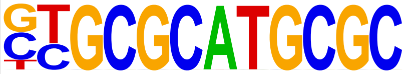	NRF(NRF)/Promoter/Homer	1e-25	-5.812e+01	0.0000	6054.0	9.51%	5198.0	8.34%	motif file (matrix)	svg
61	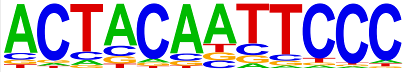	GFY(?)/Promoter/Homer	1e-24	-5.750e+01	0.0000	2029.0	3.19%	1567.8	2.52%	motif file (matrix)	svg
62	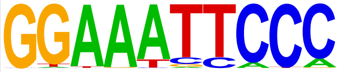	NFkB-p65-Rel(RHD)/ThioMac-LPS-Expression(GSE23622)/Homer	1e-24	-5.637e+01	0.0000	1450.0	2.28%	1073.0	1.72%	motif file (matrix)	svg
63	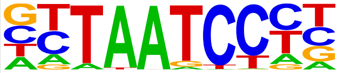	Otx2(Homeobox)/EpiLC-Otx2-ChIP-Seq(GSE56098)/Homer	1e-24	-5.581e+01	0.0000	17940.0	28.18%	16440.1	26.38%	motif file (matrix)	svg
64	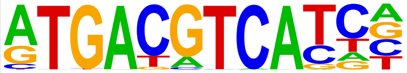	JunD(bZIP)/K562-JunD-ChIP-Seq/Homer	1e-22	-5.211e+01	0.0000	2249.0	3.53%	1780.5	2.86%	motif file (matrix)	svg
65	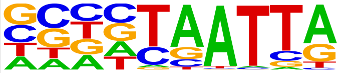	DLX1(Homeobox)/BasalGanglia-Dlx1-ChIP-seq(GSE124936)/Homer	1e-21	-5.047e+01	0.0000	22522.0	35.38%	20912.0	33.55%	motif file (matrix)	svg
66	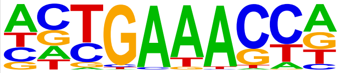	IRF4(IRF)/GM12878-IRF4-ChIP-Seq(GSE32465)/Homer	1e-21	-4.868e+01	0.0000	8994.0	14.13%	8006.5	12.85%	motif file (matrix)	svg
67	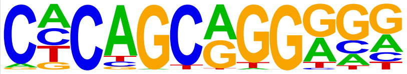	Zic2(Zf)/ESC-Zic2-ChIP-Seq(SRP197560)/Homer	1e-20	-4.748e+01	0.0000	13381.0	21.02%	12170.3	19.53%	motif file (matrix)	svg
68	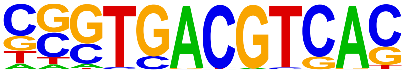	CRE(bZIP)/Promoter/Homer	1e-20	-4.738e+01	0.0000	5670.0	8.91%	4913.4	7.88%	motif file (matrix)	svg
69	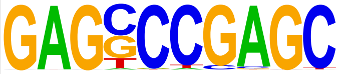	ZNF519(Zf)/HEK293-ZNF519.GFP-ChIP-Seq(GSE58341)/Homer	1e-19	-4.549e+01	0.0000	6918.0	10.87%	6087.6	9.77%	motif file (matrix)	svg
70	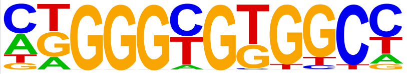	KLF6(Zf)/PDAC-KLF6-ChIP-Seq(GSE64557)/Homer	1e-19	-4.472e+01	0.0000	27988.0	43.97%	26287.0	42.18%	motif file (matrix)	svg
71	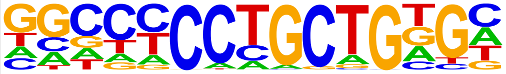	Zic3(Zf)/mES-Zic3-ChIP-Seq(GSE37889)/Homer	1e-19	-4.435e+01	0.0000	16489.0	25.90%	15174.2	24.35%	motif file (matrix)	svg
72	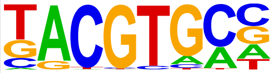	HIF-1a(bHLH)/MCF7-HIF1a-ChIP-Seq(GSE28352)/Homer	1e-18	-4.371e+01	0.0000	6004.0	9.43%	5249.3	8.42%	motif file (matrix)	svg

73	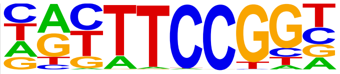	Elk4(ETS)/Hela-Elk4-ChIP-Seq(GSE31477)/Homer	1e-18	-4.227e+01	0.0000	14897.0	23.40%	13673.5	21.94%	motif file (matrix)	svg
74	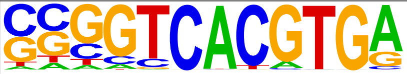	E-box(bHLH)/Promoter/Homer	1e-16	-3.779e+01	0.0000	2166.0	3.40%	1769.0	2.84%	motif file (matrix)	svg
75	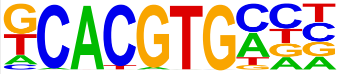	bHLHE40(bHLH)/HepG2-BHLHE40-ChIP-Seq(GSE31477)/Homer	1e-14	-3.227e+01	0.0000	7493.0	11.77%	6741.9	10.82%	motif file (matrix)	svg
76	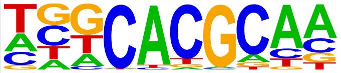	Arnt:Ahr(bHLH)/MCF7-Arnt-ChIP-Seq(Lo_et_al.)/Homer	1e-13	-3.056e+01	0.0000	15799.0	24.82%	14684.1	23.56%	motif file (matrix)	svg
77	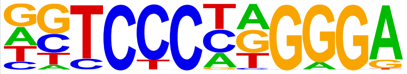	EBF(EBF)/proBcell-EBF-ChIP-Seq(GSE21978)/Homer	1e-12	-2.796e+01	0.0000	5954.0	9.35%	5333.2	8.56%	motif file (matrix)	svg
78	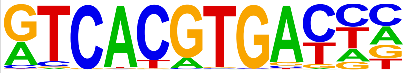	TFE3(bHLH)/MEF-TFE3-ChIP-Seq(GSE75757)/Homer	1e-10	-2.486e+01	0.0000	2091.0	3.28%	1768.2	2.84%	motif file (matrix)	svg
79	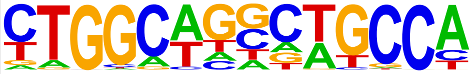	Tlx?(NR)/NPC-H3K4me1-ChIP-Seq(GSE16256)/Homer	1e-10	-2.456e+01	0.0000	11581.0	18.19%	10719.5	17.20%	motif file (matrix)	svg
80	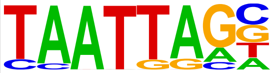	Lhx2(Homeobox)/HFSC-Lhx2-ChIP-Seq(GSE48068)/Homer	1e-10	-2.404e+01	0.0000	18178.0	28.56%	17076.0	27.40%	motif file (matrix)	svg
81	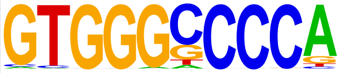	ZNF692(Zf)/HEK293-ZNF692.GFP-ChIP-Seq(GSE58341)/Homer	1e-10	-2.322e+01	0.0000	3856.0	6.06%	3410.5	5.47%	motif file (matrix)	svg
82	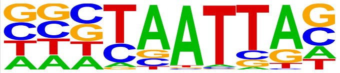	DLX2(Homeobox)/BasalGanglia-Dlx2-ChIP-seq(GSE124936)/Homer	1e-9	-2.224e+01	0.0000	24562.0	38.58%	23300.7	37.39%	motif file (matrix)	svg
83	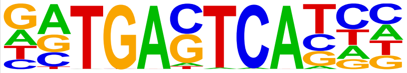	Jun-AP1(bZIP)/K562-cJun-ChIP-Seq(GSE31477)/Homer	1e-9	-2.172e+01	0.0000	4885.0	7.67%	4388.9	7.04%	motif file (matrix)	svg
84	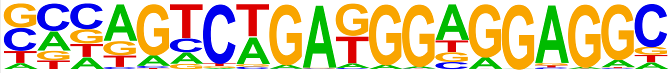	ZSCAN22(Zf)/HEK293-ZSCAN22.GFP-ChIP-Seq(GSE58341)/Homer	1e-8	-2.062e+01	0.0000	2371.0	3.72%	2052.6	3.29%	motif file (matrix)	svg
85	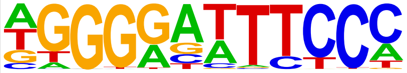	NFkB-p65(RHD)/GM12787-p65-ChIP-Seq(GSE19485)/Homer	1e-8	-1.900e+01	0.0000	11319.0	17.78%	10549.1	16.93%	motif file (matrix)	svg
86	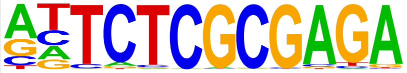	GFX(?)/Promoter/Homer	1e-7	-1.721e+01	0.0000	421.0	0.66%	313.3	0.50%	motif file (matrix)	svg
87	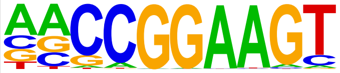	ETS(ETS)/Promoter/Homer	1e-7	-1.690e+01	0.0000	8956.0	14.07%	8316.9	13.34%	motif file (matrix)	svg
88	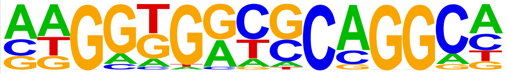	ZNF165(Zf)/WHIM12-ZNF165-ChIP-Seq(GSE65937)/Homer	1e-6	-1.456e+01	0.0000	3944.0	6.20%	3576.7	5.74%	motif file (matrix)	svg
89	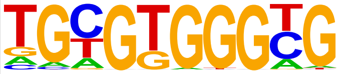	Egr1(Zf)/K562-Egr1-ChIP-Seq(GSE32465)/Homer	1e-5	-1.291e+01	0.0000	19011.0	29.86%	18099.9	29.04%	motif file (matrix)	svg
90	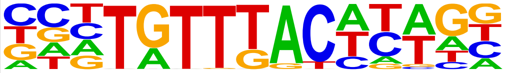	Foxa3(Forkhead)/Liver-Foxa3-ChIP-Seq(GSE77670)/Homer	1e-5	-1.224e+01	0.0000	6335.0	9.95%	5880.2	9.43%	motif file (matrix)	svg
91	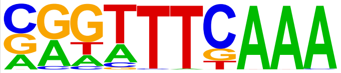	CHR(?)/Hela-CellCycle-Expression/Homer	1e-5	-1.201e+01	0.0000	10691.0	16.79%	10067.7	16.15%	motif file (matrix)	svg

92	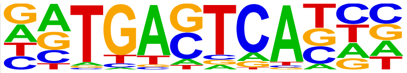	Fosl2(bZIP)/3T3L1-Fosl2-ChIP-Seq(GSE56872)/Homer	1e-4	-1.054e+01	0.0001	6557.0	10.30%	6120.1	9.82%	motif file (matrix)	svg
93	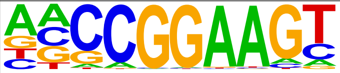	ELF1(ETS)/Jurkat-ELF1-ChIP-Seq(SRA014231)/Homer	1e-4	-1.017e+01	0.0002	13674.0	21.48%	12989.5	20.84%	motif file (matrix)	svg
94	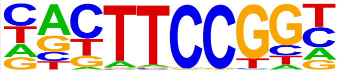	Elk1(ETS)/Hela-Elk1-ChIP-Seq(GSE31477)/Homer	1e-4	-1.014e+01	0.0002	14529.0	22.82%	13818.9	22.17%	motif file (matrix)	svg
95	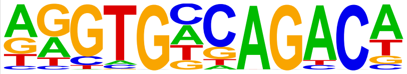	Tbox:Smad(T-box,MAD)/ESCd5-Smad2_3-ChIP-Seq(GSE29422)/Homer	1e-4	-1.009e+01	0.0002	4905.0	7.71%	4547.6	7.30%	motif file (matrix)	svg
96	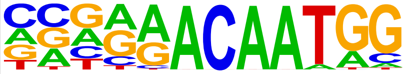	Sox7(HMG)/ESC-Sox7-ChIP-Seq(GSE133899)/Homer	1e-4	-9.775e+00	0.0003	6226.0	9.78%	5816.3	9.33%	motif file (matrix)	svg
97	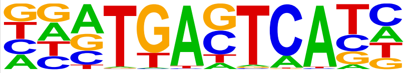	Fra1(bZIP)/BT549-Fra1-ChIP-Seq(GSE46166)/Homer	1e-3	-8.672e+00	0.0008	9978.0	15.67%	9450.2	15.16%	motif file (matrix)	svg
98	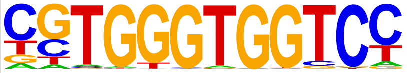	GLI3(Zf)/Limb-GLI3-ChIP-Chip(GSE11077)/Homer	1e-2	-6.523e+00	0.0066	3374.0	5.30%	3141.0	5.04%	motif file (matrix)	svg
99	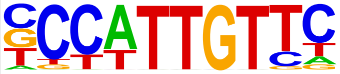	Sox2(HMG)/mES-Sox2-ChIP-Seq(GSE11431)/Homer	1e-2	-6.351e+00	0.0077	17472.0	27.45%	16785.1	26.93%	motif file (matrix)	svg
100	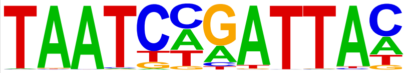	Pax7(Paired,Homeobox),long/Myoblast-Pax7-ChIP-Seq(GSE25064)/Homer	1e-2	-5.901e+00	0.0120	351.0	0.55%	295.5	0.47%	motif file (matrix)	svg
101	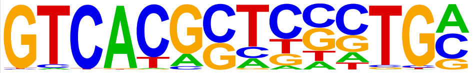	PAX5(Paired,Homeobox),condensed/GM12878-PAX5-ChIP-Seq(GSE32465)/Homer	1e-2	-5.644e+00	0.0154	2705.0	4.25%	2516.1	4.04%	motif file (matrix)	svg
102	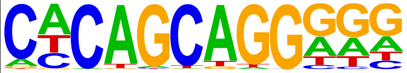	Unknown-ESC-element(?)/mES-Nanog-ChIP-Seq(GSE11724)/Homer	1e-2	-5.327e+00	0.0209	15169.0	23.83%	14580.1	23.39%	motif file (matrix)	svg
103	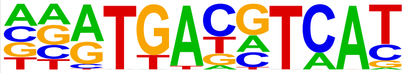	CREB5(bZIP)/LNCaP-CREB5.V5-ChIP-Seq(GSE137775)/Homer	1e-2	-4.783e+00	0.0356	6836.0	10.74%	6511.8	10.45%	motif file (matrix)	svg
