# Supplementary material for: AP-2α and AP-2β cooperatively function in the craniofacial surface ectoderm to regulate chromatin and gene expression dynamics during facial development
Source: eLife. 2022 Mar 25;11:e70511. doi: 10.7554/eLife.70511 (PMC9038197; doi:10.7554/eLife.70511)
Supplement: Figure 3—source data 2. [file elife-70511-fig3-data2.pdf]

Homer Known Motif Enrichment Results (/Users/vanottee/output/)

Homer *de novo* Motif Results
Gene Ontology Enrichment Results
Known Motif Enrichment Results (txt file)
Total Target Sequences = 3103, Total Background Sequences = 46084

Rank	Motif	Name	P-value	log P-pvalue	q-value (Benjamini)	# Target Sequences with Motif	% of Targets Sequences with Motif	# Background Sequences with Motif	% of Background Sequences with Motif	Motif File	SVG
1	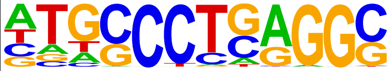	AP-2alpha(AP2)/Hela-AP2alpha-ChIP-Seq(GSE31477)/Homer	1e-736	-1.695e+03	0.0000	1916.0	61.75%	7212.1	15.65%	motif file (matrix)	svg
2	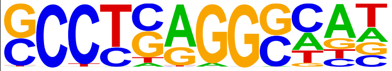	AP-2gamma(AP2)/MCF7-TFAP2C-ChIP-Seq(GSE21234)/Homer	1e-724	-1.667e+03	0.0000	2128.0	68.58%	9452.7	20.52%	motif file (matrix)	svg
3	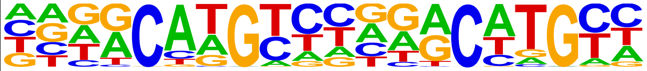	p63(p53)/Keratinocyte-p63-ChIP-Seq(GSE17611)/Homer	1e-152	-3.509e+02	0.0000	724.0	23.33%	3644.6	7.91%	motif file (matrix)	svg
4	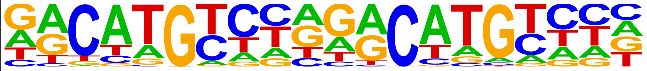	p53(p53)/Saos-p53-ChIP-Seq(GSE15780)/Homer	1e-95	-2.192e+02	0.0000	291.0	9.38%	978.2	2.12%	motif file (matrix)	svg
5	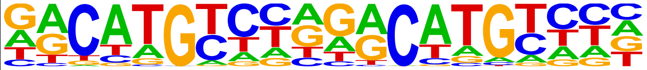	p53(p53)/Saos-p53-ChIP-Seq/Homer	1e-95	-2.192e+02	0.0000	291.0	9.38%	978.2	2.12%	motif file (matrix)	svg
6	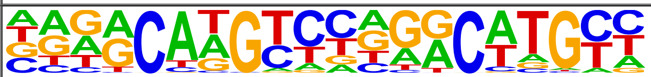	p73(p53)/Trachea-p73-ChIP-Seq(PRJNA310161)/Homer	1e-79	-1.830e+02	0.0000	179.0	5.77%	430.3	0.93%	motif file (matrix)	svg
7	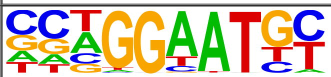	TEAD4(TEA)/Tropoblast-Tead4-ChIP-Seq(GSE37350)/Homer	1e-76	-1.760e+02	0.0000	929.0	29.94%	7596.7	16.49%	motif file (matrix)	svg
8	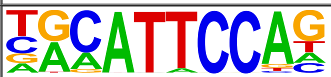	TEAD3(TEA)/HepG2-TEAD3-ChIP-Seq(Encode)/Homer	1e-76	-1.756e+02	0.0000	1135.0	36.58%	10099.9	21.92%	motif file (matrix)	svg
9	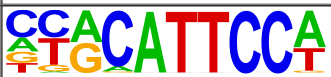	TEAD1(TEAD)/HepG2-TEAD1-ChIP-Seq(Encode)/Homer	1e-68	-1.576e+02	0.0000	992.0	31.97%	8645.9	18.76%	motif file (matrix)	svg
10	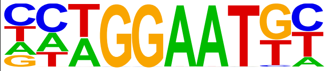	TEAD(TEA)/Fibroblast-PU.1-ChIP-Seq(Unpublished)/Homer	1e-48	-1.127e+02	0.0000	706.0	22.75%	6004.4	13.03%	motif file (matrix)	svg
11	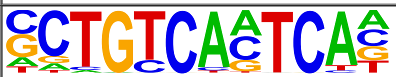	Pbx3(Homeobox)/GM12878-PBX3-ChIP-Seq(GSE32465)/Homer	1e-44	-1.019e+02	0.0000	351.0	11.31%	2290.1	4.97%	motif file (matrix)	svg
12	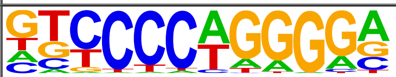	EBF1(EBF)/Near-E2A-ChIP-Seq(GSE21512)/Homer	1e-42	-9.815e+01	0.0000	981.0	31.61%	9683.0	21.02%	motif file (matrix)	svg
13	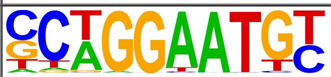	TEAD2(TEA)/Py2T-Tead2-ChIP-Seq(GSE55709)/Homer	1e-42	-9.774e+01	0.0000	583.0	18.79%	4836.1	10.50%	motif file (matrix)	svg
14	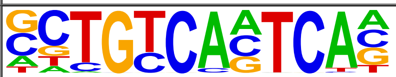	Pknox1(Homeobox)/ES-Prep1-ChIP-Seq(GSE63282)/Homer	1e-35	-8.195e+01	0.0000	312.0	10.05%	2130.7	4.62%	motif file (matrix)	svg
15	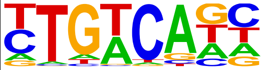	Tgif1(Homeobox)/mES-Tgif1-ChIP-Seq(GSE55404)/Homer	1e-30	-7.138e+01	0.0000	2061.0	66.42%	25868.3	56.14%	motif file (matrix)	svg
16	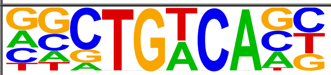	Meis1(Homeobox)/MastCells-Meis1-ChIP-Seq(GSE48085)/Homer	1e-26	-6.089e+01	0.0000	1375.0	44.31%	16105.3	34.95%	motif file (matrix)	svg

17	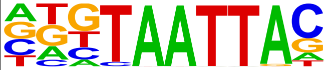	Dlx3(Homeobox)/Kerainocytes-Dlx3-ChIP-Seq(GSE89884)/Homer	1e-26	-6.070e+01	0.0000	585.0	18.85%	5588.2	12.13%	motif file (matrix)	svg
18	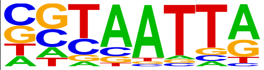	DLX5(Homeobox)/BasalGanglia-Dlx5-ChIP-seq(GSE124936)/Homer	1e-25	-5.937e+01	0.0000	662.0	21.33%	6576.3	14.27%	motif file (matrix)	svg
19	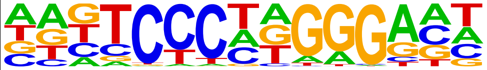	EBF2(EBF)/BrownAdipose-EBF2-ChIP-Seq(GSE97114)/Homer	1e-25	-5.920e+01	0.0000	816.0	26.30%	8550.6	18.56%	motif file (matrix)	svg
20	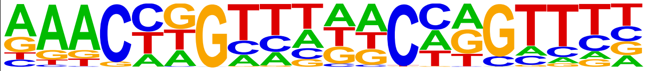	GRHL2(CP2)/HBE-GRHL2-ChIP-Seq(GSE46194)/Homer	1e-25	-5.803e+01	0.0000	414.0	13.34%	3605.7	7.83%	motif file (matrix)	svg
21	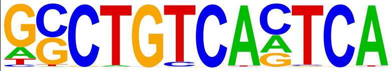	PBX1(Homeobox)/MCF7-PBX1-ChIP-Seq(GSE28007)/Homer	1e-20	-4.726e+01	0.0000	135.0	4.35%	796.5	1.73%	motif file (matrix)	svg
22	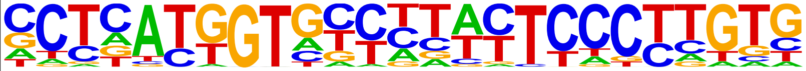	ZNF41(Zf)/HEK293-ZNF41.GFP-ChIP-Seq(GSE58341)/Homer	1e-16	-3.861e+01	0.0000	54.0	1.74%	194.5	0.42%	motif file (matrix)	svg
23	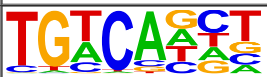	Tgif2(Homeobox)/mES-Tgif2-ChIP-Seq(GSE55404)/Homer	1e-15	-3.496e+01	0.0000	2047.0	65.97%	27169.6	58.97%	motif file (matrix)	svg
24	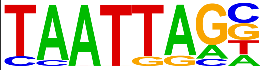	Lhx2(Homeobox)/HFSC-Lhx2-ChIP-Seq(GSE48068)/Homer	1e-13	-3.200e+01	0.0000	825.0	26.59%	9605.4	20.85%	motif file (matrix)	svg
25	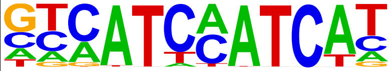	HOXA2(Homeobox)/mES-Hoxa2-ChIP-Seq(Donaldson_et_al.)/Homer	1e-11	-2.690e+01	0.0000	123.0	3.96%	916.0	1.99%	motif file (matrix)	svg
26	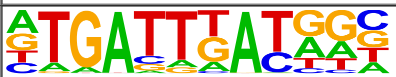	PBX2(Homeobox)/K562-PBX2-ChIP-Seq(Encode)/Homer	1e-11	-2.600e+01	0.0000	636.0	20.50%	7308.0	15.86%	motif file (matrix)	svg
27	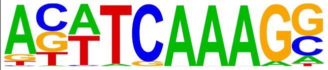	Tcf3(HMG)/mES-Tcf3-ChIP-Seq(GSE11724)/Homer	1e-11	-2.595e+01	0.0000	259.0	8.35%	2476.8	5.38%	motif file (matrix)	svg
28	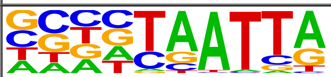	DLX1(Homeobox)/BasalGanglia-Dlx1-ChIP-seq(GSE124936)/Homer	1e-10	-2.488e+01	0.0000	962.0	31.00%	11829.6	25.67%	motif file (matrix)	svg
29	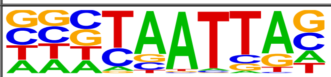	DLX2(Homeobox)/BasalGanglia-Dlx2-ChIP-seq(GSE124936)/Homer	1e-10	-2.337e+01	0.0000	1062.0	34.22%	13318.2	28.91%	motif file (matrix)	svg
30	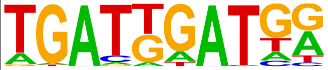	HOXA1(Homeobox)/mES-Hoxa1-ChIP-Seq(SRP084292)/Homer	1e-9	-2.265e+01	0.0000	231.0	7.44%	2223.1	4.82%	motif file (matrix)	svg
31	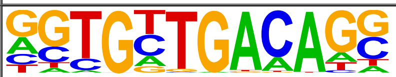	Tbx20(T-box)/Heart-Tbx20-ChIP-Seq(GSE29636)/Homer	1e-8	-2.028e+01	0.0000	217.0	6.99%	2116.8	4.59%	motif file (matrix)	svg
32	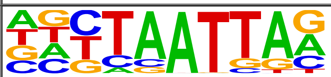	Lhx1(Homeobox)/EmbryoCarcinoma-Lhx1-ChIP-Seq(GSE70957)/Homer	1e-8	-1.982e+01	0.0000	813.0	26.20%	10024.7	21.76%	motif file (matrix)	svg
33	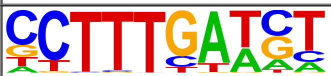	LEF1(HMG)/H1-LEF1-ChIP-Seq(GSE64758)/Homer	1e-7	-1.781e+01	0.0000	538.0	17.34%	6360.1	13.80%	motif file (matrix)	svg
34	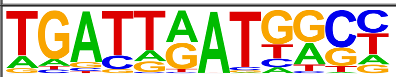	Hoxb4(Homeobox)/ES-Hoxb4-ChIP-Seq(GSE34014)/Homer	1e-7	-1.630e+01	0.0000	170.0	5.48%	1654.6	3.59%	motif file (matrix)	svg
35	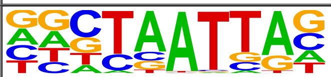	En1(Homeobox)/SUM149-EN1-ChIP-Seq(GSE120957)/Homer	1e-6	-1.483e+01	0.0000	1219.0	39.28%	16122.3	34.99%	motif file (matrix)	svg
36	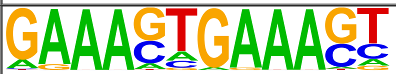	IRF1(IRF)/PBMC-IRF1-ChIP-Seq(GSE43036)/Homer	1e-6	-1.465e+01	0.0000	127.0	4.09%	1183.2	2.57%	motif file (matrix)	svg

37	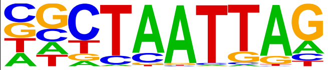	LXH9(Homeobox)/Hct116-LXH9.V5-ChIP-Seq(GSE116822)/Homer	1e-5	-1.362e+01	0.0000	977.0	31.49%	12734.7	27.64%	motif file (matrix)	svg
38	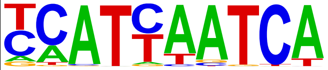	Pdx1(Homeobox)/Islet-Pdx1-ChIP-Seq(SRA008281)/Homer	1e-5	-1.329e+01	0.0000	645.0	20.79%	8077.0	17.53%	motif file (matrix)	svg
39	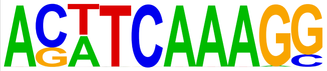	TCFL2(HMG)/K562-TCF7L2-ChIP-Seq(GSE29196)/Homer	1e-5	-1.257e+01	0.0000	90.0	2.90%	800.2	1.74%	motif file (matrix)	svg
40	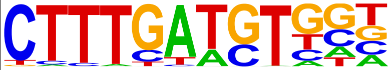	Tcf7(HMG)/GM12878-TCF7-ChIP-Seq(Encode)/Homer	1e-5	-1.234e+01	0.0000	272.0	8.77%	3075.6	6.68%	motif file (matrix)	svg
41	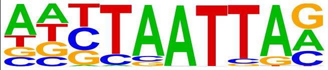	Lhx3(Homeobox)/Neuron-Lhx3-ChIP-Seq(GSE31456)/Homer	1e-5	-1.152e+01	0.0001	1092.0	35.19%	14552.9	31.58%	motif file (matrix)	svg
42	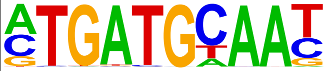	Atf4(bZIP)/MEF-Atf4-ChIP-Seq(GSE35681)/Homer	1e-4	-1.049e+01	0.0003	182.0	5.87%	1986.0	4.31%	motif file (matrix)	svg
43	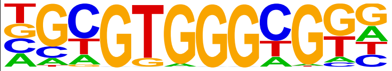	Egr2(Zf)/Thymocytes-Egr2-ChIP-Seq(GSE34254)/Homer	1e-4	-1.045e+01	0.0003	138.0	4.45%	1431.4	3.11%	motif file (matrix)	svg
44	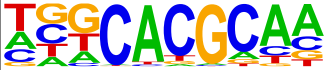	Arnt:Ahr(bHLH)/MCF7-Arnt-ChIP-Seq(Lo_et_al.)/Homer	1e-4	-1.018e+01	0.0004	438.0	14.12%	5415.2	11.75%	motif file (matrix)	svg
45	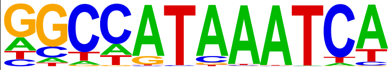	Hoxc9(Homeobox)/Ainv15-Hoxc9-ChIP-Seq(GSE21812)/Homer	1e-4	-1.017e+01	0.0004	357.0	11.50%	4312.2	9.36%	motif file (matrix)	svg
46	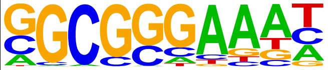	E2F4(E2F)/K562-E2F4-ChIP-Seq(GSE31477)/Homer	1e-4	-9.785e+00	0.0005	227.0	7.32%	2597.1	5.64%	motif file (matrix)	svg
47	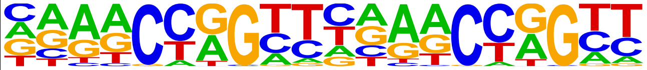	Tcfcp2l1(CP2)/mES-Tcfcp2l1-ChIP-Seq(GSE11431)/Homer	1e-4	-9.619e+00	0.0006	134.0	4.32%	1408.8	3.06%	motif file (matrix)	svg
48	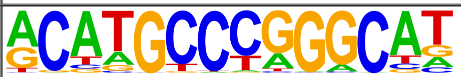	p53(p53)/mES-cMyc-ChIP-Seq(GSE11431)/Homer	1e-3	-9.191e+00	0.0009	26.0	0.84%	166.7	0.36%	motif file (matrix)	svg
49	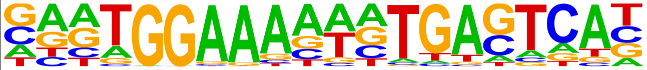	NFAT:AP1(RHD,bZIP)/Jurkat-NFATC1-ChIP-Seq(Jolma_et_al.)/Homer	1e-3	-8.378e+00	0.0021	120.0	3.87%	1274.7	2.77%	motif file (matrix)	svg
50	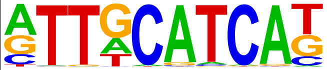	Chop(bZIP)/MEF-Chop-ChIP-Seq(GSE35681)/Homer	1e-3	-8.112e+00	0.0026	138.0	4.45%	1512.7	3.28%	motif file (matrix)	svg
51	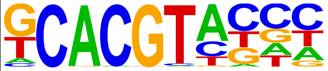	HIF2a(bHLH)/785_O-HIF2a-ChIP-Seq(GSE34871)/Homer	1e-3	-7.952e+00	0.0030	219.0	7.06%	2578.3	5.60%	motif file (matrix)	svg
52	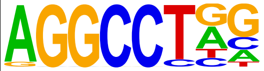	ZFX(Zf)/mES-Zfx-ChIP-Seq(GSE11431)/Homer	1e-3	-7.890e+00	0.0032	910.0	29.33%	12261.9	26.61%	motif file (matrix)	svg
53	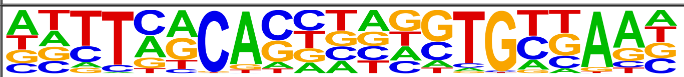	Brachyury(T-box)/Mesoendoderm-Brachyury-ChIP-exo(GSE54963)/Homer	1e-3	-7.334e+00	0.0054	211.0	6.80%	2503.0	5.43%	motif file (matrix)	svg
54	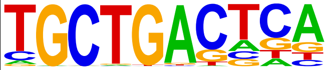	MafA(bZIP)/Islet-MafA-ChIP-Seq(GSE30298)/Homer	1e-3	-7.177e+00	0.0062	632.0	20.37%	8353.3	18.13%	motif file (matrix)	svg
55	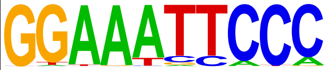	NFkB-p65-Rel(RHD)/ThioMac-LPS-Expression(GSE23622)/Homer	1e-3	-6.926e+00	0.0078	49.0	1.58%	449.7	0.98%	motif file (matrix)	svg
56	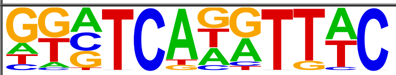	Six1(Homeobox)/Myoblast-Six1-ChIP-Chip(GSE20150)/Homer	1e-2	-6.269e+00	0.0148	178.0	5.74%	2118.7	4.60%	motif file (matrix)	svg

57	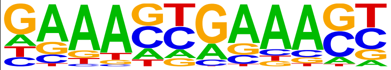	IRF2(IRF)/Erythroblas-IRF2-ChIP-Seq(GSE36985)/Homer	1e-2	-6.097e+00	0.0173	81.0	2.61%	861.9	1.87%	motif file (matrix)	svg
58	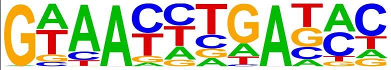	Six2(Homeobox)/NephronProgenitor-Six2-ChIP-Seq(GSE39837)/Homer	1e-2	-5.732e+00	0.0245	649.0	20.92%	8737.6	18.96%	motif file (matrix)	svg
59	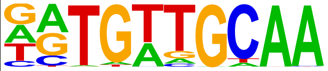	CEBP:AP1(bZIP)/ThioMac-CEBPb-ChIP-Seq(GSE21512)/Homer	1e-2	-5.606e+00	0.0273	465.0	14.99%	6133.1	13.31%	motif file (matrix)	svg
60	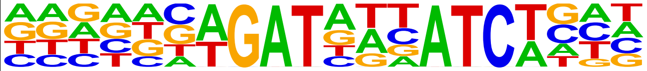	GATA(Zf),IR3/iTreg-Gata3-ChIP-Seq(GSE20898)/Homer	1e-2	-5.571e+00	0.0278	106.0	3.42%	1201.9	2.61%	motif file (matrix)	svg
61	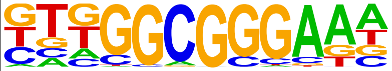	E2F3(E2F)/MEF-E2F3-ChIP-Seq(GSE71376)/Homer	1e-2	-5.107e+00	0.0435	324.0	10.44%	4196.3	9.11%	motif file (matrix)	svg
62	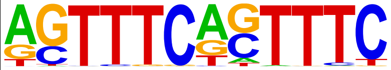	ISRE(IRF)/ThioMac-LPS-Expression(GSE23622)/Homer	1e-2	-5.098e+00	0.0435	51.0	1.64%	519.4	1.13%	motif file (matrix)	svg
