# Supplementary material for: AP-2α and AP-2β cooperatively function in the craniofacial surface ectoderm to regulate chromatin and gene expression dynamics during facial development
Source: eLife. 2022 Mar 25;11:e70511. doi: 10.7554/eLife.70511 (PMC9038197; doi:10.7554/eLife.70511)
Supplement: Figure 3—source data 3. [file elife-70511-fig3-data3.pdf]

Job Description

Region-Gene Association Graphs

What do these graphs illustrate?

Number of associated genes per region

Download as PDF.

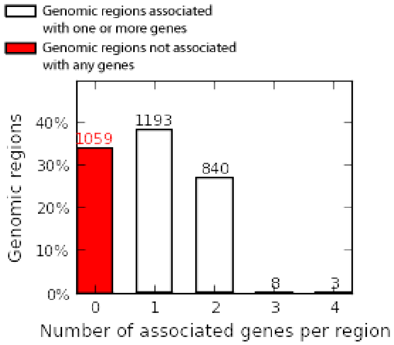

Binned by orientation and distance to TSS

Download as PDF.

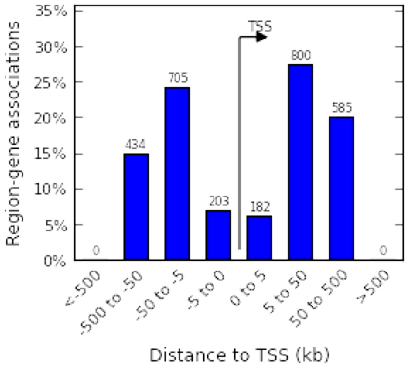

Binned by absolute distance to TSS

Download as PDF.

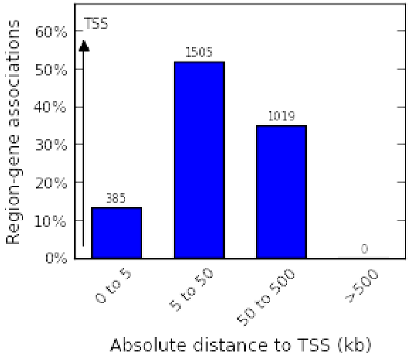

Global Controls

Global Export

Which data is exported by each option?

Ensembl Genes (no terms)

Global controls

GO Biological Process (20+ terms)

Global controls

Table controls: Export Shown top rows in this table: 20 Set Term annotation count: Min: 1 Max: Inf Set Visualize this table: [select one]

Term Name	Binom Rank	Binom Raw P-Value	*Binom FDR Q-Val*	Binom Fold Enrichment	Binom Observed Region Hits	Binom Region Set Coverage	Hyper Rank	*Hyper FDR Q-Val*	Hyper Fold Enrichment	Hyper Observed Gene Hits	Hyper Total Genes	Hyper Gene Set Coverage
pattern specification process	23	**2.2839e-32**	**1.2998e-29**	2.7903	176	5.67%	48	**9.5937e-11**	2.1198	98	440	4.36%
regulation of protein binding	31	**3.9155e-30**	**1.6534e-27**	3.9113	103	3.32%	371	**1.3204e-3**	1.9227	40	198	1.78%
regulation of binding	45	**7.0139e-27**	**2.0403e-24**	2.9083	136	4.38%	261	**7.2776e-5**	1.8050	66	348	2.94%
central nervous system development	47	**2.9169e-26**	**8.1238e-24**	2.0008	272	8.77%	128	**8.0532e-8**	1.6138	156	920	6.94%
brain development	53	**2.8999e-25**	**7.1622e-23**	2.1025	233	7.51%	192	**4.4645e-6**	1.6035	124	736	5.52%
head development	64	**1.0367e-23**	**2.1204e-21**	2.0254	238	7.67%	205	**7.2470e-6**	1.5720	129	781	5.74%
Ras protein signal transduction	66	**2.0442e-23**	**4.0543e-21**	4.1349	74	2.38%	750	**3.9994e-2**	1.8163	25	131	1.11%
small GTPase mediated signal transduction	113	**3.4680e-18**	**4.0174e-16**	2.8415	93	3.00%	681	**2.8701e-2**	1.5989	42	250	1.87%
positive regulation of intracellular transport	134	**8.6727e-17**	**8.4720e-15**	2.5838	100	3.22%	585	**1.6448e-2**	1.5810	50	301	2.22%
regulation of cell morphogenesis	140	**1.7814e-16**	**1.6656e-14**	2.1938	135	4.35%	417	**2.6842e-3**	1.5678	71	431	3.16%
gland development	141	**1.8120e-16**	**1.6822e-14**	2.1111	147	4.74%	113	**2.4797e-8**	1.9364	94	462	4.18%

Term Name	Binom Rank	Binom Raw P-Value	*Binom FDR Q-Val*	Binom Fold Enrichment	Binom Observed Region Hits	Binom Region Set Coverage	Hyper Rank	*Hyper FDR Q-Val*	Hyper Fold Enrichment	Hyper Observed Gene Hits	Hyper Total Genes	Hyper Gene Set Coverage
defense response to virus	147	**9.4252e-16**	**8.3929e-14**	3.6380	56	1.80%	504	**8.7262e-3**	1.8203	35	183	1.56%
metencephalon development	151	**3.3094e-15**	**2.8689e-13**	3.3490	60	1.93%	782	**4.7604e-2**	1.8128	24	126	1.07%
regulation of cellular protein localization	156	**4.2193e-15**	**3.5405e-13**	2.0339	146	4.71%	495	**7.2730e-3**	1.4450	87	573	3.87%
actin cytoskeleton organization	163	**6.6548e-15**	**5.3443e-13**	2.1692	124	4.00%	589	**1.7238e-2**	1.4835	65	417	2.89%
protein heterotetramerization	166	**7.3753e-15**	**5.8158e-13**	7.9822	25	0.81%	155	**4.9257e-7**	4.2299	20	45	0.89%
hindbrain development	178	**1.8311e-14**	**1.3466e-12**	2.8239	73	2.35%	443	**4.4376e-3**	1.9147	34	169	1.51%
muscle structure development	188	**5.3888e-14**	**3.7521e-12**	2.0113	138	4.45%	127	**8.0596e-8**	1.8914	94	473	4.18%
response to virus	189	**5.5440e-14**	**3.8397e-12**	2.8832	68	2.19%	451	**4.8427e-3**	1.7236	46	254	2.05%
adult walking behavior	190	**5.6152e-14**	**3.8686e-12**	5.2353	33	1.06%	588	**1.6819e-2**	2.7856	12	41	0.53%

The test set of 3,103 genomic regions picked 2,248 (11%) of all 21,395 genes.
GO Biological Process has 13,090 terms covering 17,925 (84%) of all 21,395 genes, and 1,163,819 term - gene associations.
13,090 ontology terms (100%) were tested using an annotation count range of [1, Inf].

GO Cellular Component (8 terms)

Global controls

Table controls: Export Shown top rows in this table: 20 Set Term annotation count: Min: 1 Max: Inf Set Visualize this table: [select one]

Term Name	Binom Rank	Binom Raw P-Value	*Binom FDR Q-Val*	Binom Fold Enrichment	Binom Observed Region Hits	Binom Region Set Coverage	Hyper Rank	*Hyper FDR Q-Val*	Hyper Fold Enrichment	Hyper Observed Gene Hits	Hyper Total Genes	Hyper Gene Set Coverage
anchoring junction	23	**1.2121e-18**	**8.9277e-17**	2.2088	152	4.90%	27	**5.7414e-4**	1.5862	87	522	3.87%
adherens junction	24	**1.2430e-17**	**8.7734e-16**	2.1888	146	4.71%	31	**3.4431e-3**	1.5326	81	503	3.60%
endoplasmic reticulum lumen	50	**5.4844e-11**	**1.8581e-9**	3.3789	41	1.32%	33	**5.7393e-3**	2.2031	25	108	1.11%
nuclear nucleosome	56	**3.6967e-10**	**1.1182e-8**	7.4791	17	0.55%	48	**2.6663e-2**	2.7856	12	41	0.53%
nucleosome	69	**1.0227e-6**	**2.5109e-5**	3.4468	22	0.71%	9	**5.3360e-6**	2.7423	34	118	1.51%
cell-cell adherens junction	70	**1.0369e-6**	**2.5093e-5**	2.4733	37	1.19%	15	**6.3112e-5**	2.7192	28	98	1.25%
DNA packaging complex	72	**7.2118e-6**	**1.6968e-4**	3.0419	22	0.71%	11	**2.5357e-5**	2.5682	34	126	1.51%
basement membrane	86	**1.3524e-4**	**2.6640e-3**	2.1080	31	1.00%	26	**5.8999e-4**	2.5312	25	94	1.11%

The test set of 3,103 genomic regions picked 2,248 (11%) of all 21,395 genes.
GO Cellular Component has 1,694 terms covering 19,074 (89%) of all 21,395 genes, and 371,380 term - gene associations.
1,694 ontology terms (100%) were tested using an annotation count range of [1, Inf].

GO Molecular Function (4 terms)

Global controls

Table controls: Export Shown top rows in this table: 20 Set Term annotation count: Min: 1 Max: Inf Set Visualize this table: [select one]

Term Name	Binom Rank	Binom Raw P-Value	Binom FDR Q-Val	Binom Fold Enrichment	Binom Observed Region Hits	Binom Region Set Coverage	Hyper Rank	Hyper FDR Q-Val	Hyper Fold Enrichment	Hyper Observed Gene Hits	Hyper Total Genes	Hyper Gene Set Coverage
type I interferon receptor binding	13	**3.3247e-15**	**1.0565e-12**	20.5527	15	0.48%	11	**3.3779e-4**	4.2982	14	31	0.62%
frizzled binding	48	**1.6529e-7**	**1.4225e-5**	3.8536	22	0.71%	14	**4.7547e-3**	3.5064	14	38	0.62%
S100 protein binding	86	**6.5131e-5**	**3.1286e-3**	5.3566	9	0.29%	22	**2.5432e-2**	5.1247	7	13	0.31%
beta-catenin binding	88	**7.6381e-5**	**3.5856e-3**	2.2841	28	0.90%	18	**1.3020e-2**	2.4983	21	80	0.93%

The test set of 3,103 genomic regions picked 2,248 (11%) of all 21,395 genes.
GO Molecular Function has 4,131 terms covering 17,189 (80%) of all 21,395 genes, and 227,341 term - gene associations.
4,131 ontology terms (100%) were tested using an annotation count range of [1, Inf].

Human Phenotype (20+ terms)

Global controls

Table controls: Export Shown top rows in this table: 20 Set Term annotation count: Min: 1 Max: Inf Set Visualize this table: [select one]

Term Name	Binom Rank	Binom Raw P-Value	Binom FDR Q-Val	Binom Fold Enrichment	Binom Observed Region Hits	Binom Region Set Coverage	Hyper Rank	Hyper FDR Q-Val	Hyper Fold Enrichment	Hyper Observed Gene Hits	Hyper Total Genes	Hyper Gene Set Coverage
Functional abnormality of the middle ear	17	**1.1778e-33**	**4.5718e-31**	6.1359	74	2.38%	97	**4.0674e-2**	2.2325	19	81	0.85%
Limited elbow movement	46	**4.9100e-11**	**7.0437e-9**	4.0397	33	1.06%	69	**2.8787e-2**	2.4128	18	71	0.80%
Sparse hair	52	**1.4130e-10**	**1.7932e-8**	2.8990	49	1.58%	13	**3.9849e-3**	2.2560	32	135	1.42%
Abnormality of hair density	54	**3.3501e-10**	**4.0939e-8**	2.8231	49	1.58%	19	**5.1740e-3**	2.1910	32	139	1.42%
Cleft lip	59	**1.2060e-9**	**1.3489e-7**	2.8569	45	1.45%	1	**1.2962e-3**	2.7879	29	99	1.29%
Limitation of joint mobility	63	**5.3272e-9**	**5.5800e-7**	2.8759	41	1.32%	74	**3.2566e-2**	2.0512	25	116	1.11%
Aplasia/Hypoplasia of the eyebrow	65	**9.6810e-9**	**9.8285e-7**	3.4023	31	1.00%	45	**1.7114e-2**	2.5115	19	72	0.85%
Syndactyly	66	**1.1190e-8**	**1.1189e-6**	2.5244	49	1.58%	17	**4.2162e-3**	2.2230	32	137	1.42%
Regional abnormality of skin	69	**1.5129e-8**	**1.4469e-6**	2.4994	49	1.58%	47	**1.6571e-2**	2.0071	31	147	1.38%
Cleft upper lip	73	**2.1284e-8**	**1.9240e-6**	2.7322	41	1.32%	7	**2.0747e-3**	2.6608	26	93	1.16%
Cleft palate	75	**2.2795e-8**	**2.0056e-6**	2.1505	65	2.09%	10	**4.1233e-3**	1.9736	45	217	2.00%
Oral cleft	76	**3.2068e-8**	**2.7844e-6**	2.0763	69	2.22%	6	**1.7865e-3**	2.0015	49	233	2.18%
Abnormality of the hard palate	81	**6.3399e-8**	**5.1651e-6**	2.0760	66	2.13%	9	**3.2742e-3**	1.9810	46	221	2.05%
Aplasia/Hypoplasia affecting the eye	86	**9.1991e-8**	**7.0587e-6**	2.2063	56	1.80%	23	**5.6627e-3**	2.0355	37	173	1.65%
Abnormality of thumb phalanx	88	**1.2509e-7**	**9.3801e-6**	3.0258	31	1.00%	33	**1.2469e-2**	2.6207	19	69	0.85%
Clinodactyly	94	**2.8547e-7**	**2.0041e-5**	2.2828	48	1.55%	65	**2.8012e-2**	1.9423	30	147	1.33%
Microphthalmia	97	**3.1997e-7**	**2.1768e-5**	2.3725	44	1.42%	12	**3.7257e-3**	2.3050	31	128	1.38%
Finger syndactyly	109	**9.1930e-7**	**5.5655e-5**	3.3557	23	0.74%	3	**2.4568e-3**	3.8069	16	40	0.71%
Triangular shaped phalanges of the hand	111	**1.0230e-6**	**6.0820e-5**	2.7346	31	1.00%	58	**2.3789e-2**	2.4111	19	75	0.85%

Term Name	Binom Rank	Binom Raw P-Value	*Binom FDR Q-Val*	Binom Fold Enrichment	Binom Observed Region Hits	Binom Region Set Coverage	Hyper Rank	*Hyper FDR Q-Val*	Hyper Fold Enrichment	Hyper Observed Gene Hits	Hyper Total Genes	Hyper Gene Set Coverage
Abnormal morphology of bones of the upper limbs	112	**1.1005e-6**	**6.4839e-5**	2.1937	47	1.51%	35	**1.2072e-2**	2.0440	32	149	1.42%

The test set of 3,103 genomic regions picked 2,248 (11%) of all 21,395 genes.
Human Phenotype has 6,599 terms covering 3,215 (15%) of all 21,395 genes, and 244,972 term - gene associations.
6,599 ontology terms (100%) were tested using an annotation count range of [1, Inf].

Mouse Phenotype Single KO (20+ terms)

Global controls

Table controls: Export Shown top rows in this table: 20 Set Term annotation count: Min: 1 Max: Inf Set Visualize this table: [select one]

Term Name	Binom Rank	Binom Raw P-Value	*Binom FDR Q-Val*	Binom Fold Enrichment	Binom Observed Region Hits	Binom Region Set Coverage	Hyper Rank	*Hyper FDR Q-Val*	Hyper Fold Enrichment	Hyper Observed Gene Hits	Hyper Total Genes	Hyper Gene Set Coverage
abnormal trachea morphology	17	**1.3432e-31**	**7.2452e-29**	6.0599	70	2.26%	526	**3.1097e-2**	2.2069	16	69	0.71%
abnormal brain ventricular system morphology	28	**1.3143e-19**	**4.3044e-17**	2.7250	108	3.48%	645	**4.7009e-2**	1.5173	44	276	1.96%
postnatal lethality, incomplete penetrance	31	**5.9181e-19**	**1.7506e-16**	2.0694	179	5.77%	86	**2.1858e-5**	1.6602	101	579	4.49%
abnormal erythrocyte morphology	35	**1.2254e-18**	**3.2106e-16**	2.0537	179	5.77%	329	**8.9542e-3**	1.3902	104	712	4.63%
abnormal brain development	37	**4.4079e-18**	**1.0925e-15**	2.0357	177	5.70%	104	**5.2967e-5**	1.6237	101	592	4.49%
abnormal esophageal smooth muscle morphology	80	**2.7347e-9**	**3.1347e-7**	14.9062	10	0.32%	258	**4.3216e-3**	9.5173	4	4	0.18%
abnormal renal glomerulus basement membrane morphology	97	**5.9975e-8**	**5.6698e-6**	4.0934	22	0.71%	125	**1.4487e-4**	3.8584	15	37	0.67%
abnormal hair growth	109	**2.4744e-7**	**2.0817e-5**	2.1053	58	1.87%	90	**3.0880e-5**	2.1506	47	208	2.09%
abnormal esophagus development	122	**7.5993e-7**	**5.7120e-5**	9.4344	9	0.29%	164	**7.1321e-4**	9.5173	5	5	0.22%
abnormal cornea morphology	126	**9.6236e-7**	**7.0038e-5**	2.1445	50	1.61%	80	**1.3037e-5**	2.3649	41	165	1.82%
abnormal incisor morphology	129	**1.0342e-6**	**7.3513e-5**	2.5492	35	1.13%	178	**9.5195e-4**	2.5453	23	86	1.02%
abnormal keratinocyte morphology	134	**1.3741e-6**	**9.4035e-5**	3.3836	22	0.71%	222	**3.1107e-3**	2.9742	15	48	0.67%
abnormal renal glomerulus basement membrane thickness	139	**1.8537e-6**	**1.2229e-4**	4.3169	16	0.52%	224	**3.2176e-3**	3.9656	10	24	0.44%
alopecia	141	**1.8843e-6**	**1.2254e-4**	2.5189	34	1.10%	102	**5.0241e-5**	2.7192	28	98	1.25%
abnormal eye size	147	**2.9844e-6**	**1.8617e-4**	2.0245	52	1.68%	115	**9.2589e-5**	2.1800	41	179	1.82%
abnormal cranial suture morphology	152	**4.0612e-6**	**2.4501e-4**	2.8994	25	0.81%	103	**5.2780e-5**	3.4775	19	52	0.85%
microphthalmia	161	**6.1808e-6**	**3.5204e-4**	2.0174	49	1.58%	112	**8.7548e-5**	2.2360	39	166	1.73%
thick epidermis	171	**1.1351e-5**	**6.0872e-4**	2.6634	26	0.84%	157	**5.7890e-4**	2.7759	21	72	0.93%
abnormal palatine bone horizontal plate morphology	173	**1.2354e-5**	**6.5482e-4**	3.5259	17	0.55%	198	**2.0482e-3**	3.8774	11	27	0.49%

Term Name	Binom Rank	Binom Raw P-Value	*Binom FDR Q-Val*	Binom Fold Enrichment	Binom Observed Region Hits	Binom Region Set Coverage	Hyper Rank	*Hyper FDR Q-Val*	Hyper Fold Enrichment	Hyper Observed Gene Hits	Hyper Total Genes	Hyper Gene Set Coverage
abnormal outer ear morphology	175	**1.4051e-5**	**7.3629e-4**	2.2236	36	1.16%	188	**1.3991e-3**	2.3344	26	106	1.16%

The test set of 3,103 genomic regions picked 2,248 (11%) of all 21,395 genes.
Mouse Phenotype Single KO has 9,170 terms covering 9,466 (44%) of all 21,395 genes, and 551,620 term - gene associations.
9,170 ontology terms (100%) were tested using an annotation count range of [1, Inf].

Mouse Phenotype (20+ terms)

Global controls

Table controls: Export Shown top rows in this table: 20 Set Term annotation count: Min: 1 Max: Inf Set Visualize this table: [select one]

Term Name	Binom Rank	Binom Raw P-Value	*Binom FDR Q-Val*	Binom Fold Enrichment	Binom Observed Region Hits	Binom Region Set Coverage	Hyper Rank	*Hyper FDR Q-Val*	Hyper Fold Enrichment	Hyper Observed Gene Hits	Hyper Total Genes	Hyper Gene Set Coverage
abnormal digestive system morphology	18	**6.6267e-30**	**3.5251e-27**	2.0062	308	9.93%	59	**1.4257e-9**	1.6207	188	1,104	8.36%
abnormal trachea morphology	19	**4.0448e-29**	**2.0384e-26**	4.9653	77	2.48%	329	**9.7812e-4**	2.4595	23	89	1.02%
abnormal mean corpuscular volume	40	**3.3527e-21**	**8.0256e-19**	3.1482	94	3.03%	893	**3.4596e-2**	1.5539	40	245	1.78%
abnormal blood urea nitrogen level	43	**3.2717e-20**	**7.2852e-18**	2.9968	96	3.09%	654	**1.3867e-2**	1.6465	41	237	1.82%
abnormal brain ventricular system morphology	54	**4.0917e-18**	**7.2552e-16**	2.3295	132	4.25%	599	**9.7321e-3**	1.5130	62	390	2.76%
abnormal respiratory epithelium morphology	59	**1.7901e-17**	**2.9051e-15**	2.6339	101	3.25%	517	**6.0016e-3**	1.6861	45	254	2.00%
abnormal tail morphology	85	**2.0432e-14**	**2.3016e-12**	2.1339	124	4.00%	138	**1.3824e-5**	1.7975	78	413	3.47%
curly tail	88	**4.8934e-14**	**5.3243e-12**	4.2504	41	1.32%	574	**8.7165e-3**	2.3793	17	68	0.76%
increased regulatory T cell number	92	**7.4708e-14**	**7.7753e-12**	4.4985	38	1.22%	672	**1.5378e-2**	2.3072	16	66	0.71%
abnormal brainstem morphology	101	**4.0140e-13**	**3.8053e-11**	2.3760	89	2.87%	135	**1.1412e-5**	2.0844	53	242	2.36%
abnormal midbrain morphology	112	**2.0878e-12**	**1.7849e-10**	2.6795	67	2.16%	244	**1.9432e-4**	2.1823	36	157	1.60%
abnormal regulatory T cell number	113	**2.2854e-12**	**1.9365e-10**	2.9883	56	1.80%	427	**2.6466e-3**	2.0071	31	147	1.38%
abnormal regulatory T cell morphology	121	**6.1611e-12**	**4.8754e-10**	2.9108	56	1.80%	461	**3.6095e-3**	1.9669	31	150	1.38%
hair follicle degeneration	122	**6.1648e-12**	**4.8384e-10**	6.1226	24	0.77%	148	**2.0863e-5**	4.5946	14	29	0.62%
decreased bone marrow cell number	134	**2.5847e-11**	**1.8469e-9**	2.7704	57	1.84%	622	**1.0812e-2**	1.8421	30	155	1.33%
abnormal circulating amino acid level	136	**4.3498e-11**	**3.0624e-9**	2.3833	73	2.35%	937	**3.5475e-2**	1.5475	40	246	1.78%
abnormal amino acid level	144	**1.3331e-10**	**8.8645e-9**	2.1738	84	2.71%	952	**3.7798e-2**	1.4784	48	309	2.14%
increased cell proliferation	151	**1.8315e-10**	**1.1614e-8**	2.2201	79	2.55%	334	**1.0599e-3**	1.8057	48	253	2.14%
abnormal hair growth	153	**2.0547e-10**	**1.2859e-8**	2.1103	88	2.84%	105	**8.8795e-7**	2.0528	66	306	2.94%
small hair follicles	168	**7.3622e-10**	**4.1960e-8**	7.7448	16	0.52%	774	**2.3534e-2**	4.0789	6	14	0.27%

The test set of 3,103 genomic regions picked 2,248 (11%) of all 21,395 genes.
Mouse Phenotype has 9,575 terms covering 9,654 (45%) of all 21,395 genes, and 705,265 term - gene associations.
9,575 ontology terms (100%) were tested using an annotation count range of [1, Inf].
