# Supplementary material for: AP-2α and AP-2β cooperatively function in the craniofacial surface ectoderm to regulate chromatin and gene expression dynamics during facial development
Source: eLife. 2022 Mar 25;11:e70511. doi: 10.7554/eLife.70511 (PMC9038197; doi:10.7554/eLife.70511)
Supplement: Figure 3—source data 4. [file elife-70511-fig3-data4.pdf]

Job Description

Region-Gene Association Graphs

What do these graphs illustrate?

Number of associated genes per region

Download as PDF.

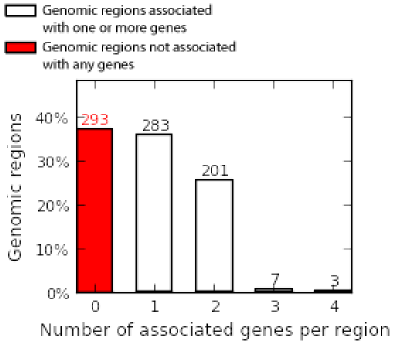

Binned by orientation and distance to TSS

Download as PDF.

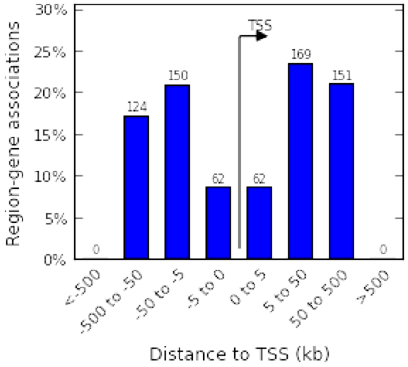

Binned by absolute distance to TSS

Download as PDF.

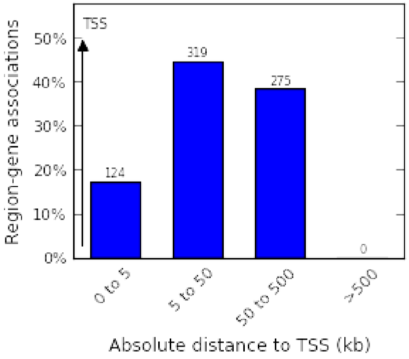

Global Controls

Global Export

Which data is exported by each option?

Ensembl Genes (no terms)

Global controls

GO Biological Process (20+ terms)

Global controls

Table controls: Export   Shown top rows in this table: 20 Set   Term annotation count: Min: 1 Max: Inf Set   Visualize this table: [select one]

Term Name	Binom Rank	Binom Raw P-Value	*Binom FDR Q-Val*	Binom Fold Enrichment	Binom Observed Region Hits	Binom Region Set Coverage	Hyper Rank	*Hyper FDR Q-Val*	Hyper Fold Enrichment	Hyper Observed Gene Hits	Hyper Total Genes	Hyper Gene Set Coverage
regulation of protein binding	11	**3.3774e-49**	**4.0191e-46**	10.7800	72	9.15%	164	**9.1956e-4**	3.4144	17	198	3.16%
pattern specification process	12	**4.6653e-48**	**5.0891e-45**	6.3134	101	12.83%	19	**1.7294e-9**	3.6152	40	440	7.43%
regulation of binding	23	**4.6367e-40**	**2.6389e-37**	6.7453	80	10.17%	187	**1.9099e-3**	2.6283	23	348	4.28%
brain development	33	**2.1480e-27**	**8.5206e-25**	3.5578	100	12.71%	150	**4.0043e-4**	2.1613	40	736	7.43%
central nervous system development	35	**7.4253e-27**	**2.7771e-24**	3.2194	111	14.10%	118	**7.4670e-5**	2.1181	49	920	9.11%
head development	36	**1.1628e-26**	**4.2282e-24**	3.4224	102	12.96%	142	**3.1632e-4**	2.1386	42	781	7.81%
locomotion	39	**2.6540e-24**	**8.9080e-22**	2.9700	112	14.23%	296	**2.3415e-2**	1.6740	45	1,069	8.36%
nervous system development	48	**4.6297e-21**	**1.2626e-18**	2.1614	164	20.84%	76	**2.6415e-6**	1.7711	97	2,178	18.03%
regulation of anatomical structure morphogenesis	49	**7.7588e-21**	**2.0727e-18**	2.9249	98	12.45%	141	**2.8664e-4**	2.0427	47	915	8.74%
cell projection organization	52	**2.5787e-20**	**6.4914e-18**	2.8343	100	12.71%	279	**1.5997e-2**	1.7343	43	986	7.99%

Term Name	Binom Rank	Binom Raw P-Value	*Binom FDR Q-Val*	Binom Fold Enrichment	Binom Observed Region Hits	Binom Region Set Coverage	Hyper Rank	*Hyper FDR Q-Val*	Hyper Fold Enrichment	Hyper Observed Gene Hits	Hyper Total Genes	Hyper Gene Set Coverage
DNA replication-dependent nucleosome assembly	53	**4.0209e-20**	**9.9308e-18**	54.8546	14	1.78%	72	**9.3054e-7**	12.0508	10	33	1.86%
regulation of developmental process	56	**1.3150e-19**	**3.0738e-17**	2.0635	168	21.35%	22	**1.8492e-9**	1.9191	115	2,383	21.38%
protein heterotetramerization	58	**9.7633e-19**	**2.2035e-16**	22.6602	18	2.29%	21	**1.7387e-9**	12.3722	14	45	2.60%
chromatin silencing at rDNA	76	**5.1014e-14**	**8.7864e-12**	19.5040	14	1.78%	107	**4.7332e-5**	7.9535	10	50	1.86%
innate immune response in mucosa	82	**1.5673e-13**	**2.5020e-11**	40.9740	10	1.27%	115	**5.9591e-5**	13.2559	7	21	1.30%
protein heterooligomerization	94	**1.3399e-11**	**1.8660e-9**	6.8756	21	2.67%	74	**1.5359e-6**	5.6338	17	120	3.16%
negative regulation of transcription, DNA-templated	98	**2.4885e-11**	**3.3239e-9**	2.1766	86	10.93%	42	**5.4079e-8**	2.2468	70	1,239	13.01%
nucleosome assembly	100	**4.4017e-11**	**5.7619e-9**	8.5597	17	2.16%	23	**2.6306e-9**	7.1653	20	111	3.72%
protein tetramerization	101	**5.7845e-11**	**7.4969e-9**	5.9953	22	2.80%	97	**1.6537e-5**	4.7276	17	143	3.16%
negative regulation of nucleic acid-templated transcription	102	**6.0915e-11**	**7.8174e-9**	2.1375	86	10.93%	47	**1.5556e-7**	2.1868	70	1,273	13.01%

The test set of 787 genomic regions picked 538 (3%) of all 21,395 genes.
GO Biological Process has 13,090 terms covering 17,925 (84%) of all 21,395 genes, and 1,163,819 term - gene associations.
13,090 ontology terms (100%) were tested using an annotation count range of [1, Inf].

⊖ GO Cellular Component (9 terms)

Global controls

Table controls: Export Shown top rows in this table: 20 Set Term annotation count: Min: 1 Max: Inf Set Visualize this table: [select one]

Term Name	Binom Rank	Binom Raw P-Value	*Binom FDR Q-Val*	Binom Fold Enrichment	Binom Observed Region Hits	Binom Region Set Coverage	Hyper Rank	*Hyper FDR Q-Val*	Hyper Fold Enrichment	Hyper Observed Gene Hits	Hyper Total Genes	Hyper Gene Set Coverage
nuclear nucleosome	23	**2.6968e-15**	**1.9863e-13**	24.2849	14	1.78%	12	**7.3036e-6**	9.6994	10	41	1.86%
nucleosome	25	**1.9105e-12**	**1.2946e-10**	10.5013	17	2.16%	1	**9.6018e-19**	10.1104	30	118	5.58%
DNA packaging complex	28	**1.3103e-11**	**7.9273e-10**	9.2680	17	2.16%	2	**3.7732e-18**	9.4685	30	126	5.58%
nuclear chromatin	48	**2.4684e-7**	**8.7114e-6**	3.1089	28	3.56%	27	**3.6489e-3**	2.5657	22	341	4.09%
chromatin	49	**4.0390e-7**	**1.3963e-5**	2.6327	35	4.45%	4	**6.0265e-11**	3.5179	46	520	8.55%
protein-DNA complex	51	**7.4228e-7**	**2.4655e-5**	4.1302	18	2.29%	3	**1.2662e-12**	5.8426	31	211	5.76%
extracellular matrix	56	**1.1946e-5**	**3.6136e-4**	2.1654	38	4.83%	25	**2.6720e-3**	2.2341	30	534	5.58%
basement membrane	72	**1.6216e-3**	**3.8153e-2**	2.9492	11	1.40%	33	**3.0857e-2**	3.8075	9	94	1.67%
cell-cell adherens junction	73	**1.8493e-3**	**4.2914e-2**	2.8992	11	1.40%	31	**9.8123e-3**	4.0579	10	98	1.86%

The test set of 787 genomic regions picked 538 (3%) of all 21,395 genes.
GO Cellular Component has 1,694 terms covering 19,074 (89%) of all 21,395 genes, and 371,380 term - gene associations.
1,694 ontology terms (100%) were tested using an annotation count range of [1, Inf].

GO Molecular Function (7 terms)

Global controls

Table controls: Export | Shown top rows in this table: 20 Set | Term annotation count: Min: 1 Max: Inf Set | Visualize this table: [select one]

Term Name	Binom Rank	Binom Raw P-Value	*Binom FDR Q-Val*	Binom Fold Enrichment	Binom Observed Region Hits	Binom Region Set Coverage	Hyper Rank	*Hyper FDR Q-Val*	Hyper Fold Enrichment	Hyper Observed Gene Hits	Hyper Total Genes	Hyper Gene Set Coverage
frizzled binding	22	**4.7801e-9**	**8.9757e-7**	8.9784	13	1.65%	22	**7.3648e-3**	7.3256	7	38	1.30%
G-protein coupled receptor binding	24	**1.0489e-8**	**1.8055e-6**	3.6336	28	3.56%	26	**3.5468e-2**	2.6934	17	251	3.16%
chromatin DNA binding	25	**1.2979e-8**	**2.1447e-6**	5.8248	17	2.16%	23	**1.5759e-2**	4.0504	11	108	2.04%
chromatin binding	26	**1.5735e-8**	**2.5001e-6**	2.5503	46	5.84%	10	**1.4270e-5**	2.6985	38	560	7.06%
sequence-specific DNA binding	27	**3.0690e-8**	**4.6956e-6**	2.0067	72	9.15%	9	**3.4330e-7**	2.3086	62	1,068	11.52%
transcriptional activator activity, RNA polymerase II transcription regulatory region sequence-specific binding	30	**2.9998e-7**	**4.1307e-5**	2.5066	39	4.96%	13	**9.5919e-5**	2.8541	30	418	5.58%
protein heterodimerization activity	54	**3.0945e-5**	**2.3673e-3**	2.1202	36	4.57%	4	**1.5900e-9**	3.2901	46	556	8.55%

The test set of 787 genomic regions picked 538 (3%) of all 21,395 genes.
GO Molecular Function has 4,131 terms covering 17,189 (80%) of all 21,395 genes, and 227,341 term - gene associations.
4,131 ontology terms (100%) were tested using an annotation count range of [1, Inf].

Human Phenotype (20+ terms)

Global controls

Table controls: Export | Shown top rows in this table: 20 Set | Term annotation count: Min: 1 Max: Inf Set | Visualize this table: [select one]

Term Name	Binom Rank	Binom Raw P-Value	*Binom FDR Q-Val*	Binom Fold Enrichment	Binom Observed Region Hits	Binom Region Set Coverage	Hyper Rank	*Hyper FDR Q-Val*	Hyper Fold Enrichment	Hyper Observed Gene Hits	Hyper Total Genes	Hyper Gene Set Coverage
Recurrent upper respiratory tract infections	11	**5.9186e-65**	**3.5506e-62**	30.9501	58	7.37%	255	**4.8293e-2**	3.9768	7	70	1.30%
Abnormality of the nasopharynx	16	**1.8390e-59**	**7.5847e-57**	22.7873	60	7.62%	120	**9.8230e-3**	4.4739	9	80	1.67%
Abnormality of the pharynx	18	**2.8977e-57**	**1.0623e-54**	20.8675	60	7.62%	162	**1.7760e-2**	3.9768	9	90	1.67%
Conductive hearing impairment	19	**1.2727e-56**	**4.4204e-54**	20.3359	60	7.62%	195	**2.5720e-2**	4.0787	8	78	1.49%
Functional abnormality of the middle ear	20	**1.0032e-55**	**3.3099e-53**	19.6158	60	7.62%	205	**3.1434e-2**	3.9277	8	81	1.49%
Abnormality of the middle ear	21	**4.2467e-48**	**1.3345e-45**	13.1214	63	8.01%	226	**4.5449e-2**	2.8970	11	151	2.04%
Abnormality of the upper respiratory tract	23	**4.8568e-47**	**1.3935e-44**	11.5820	66	8.39%	116	**8.6702e-3**	3.1633	14	176	2.60%
Abnormality of the nose	27	**1.1965e-34**	**2.9244e-32**	4.4136	100	12.71%	19	**3.9342e-4**	2.2888	40	695	7.43%
Hearing impairment	30	**4.0454e-33**	**8.8986e-31**	4.9860	85	10.80%	140	**1.3498e-2**	2.0544	28	542	5.20%
Hearing abnormality	31	**6.1047e-33**	**1.2995e-30**	4.9566	85	10.80%	149	**1.4271e-2**	2.0394	28	546	5.20%
Abnormality of the ear	33	**5.3954e-31**	**1.0789e-28**	3.6724	109	13.85%	48	**1.4405e-3**	1.9670	46	930	8.55%
Abnormality of the face	35	**3.2887e-29**	**6.2006e-27**	3.0068	132	16.77%	2	**3.3248e-4**	1.9469	68	1,389	12.64%

Term Name	Binom Rank	Binom Raw P-Value	*Binom FDR Q-Val*	Binom Fold Enrichment	Binom Observed Region Hits	Binom Region Set Coverage	Hyper Rank	*Hyper FDR Q-Val*	Hyper Fold Enrichment	Hyper Observed Gene Hits	Hyper Total Genes	Hyper Gene Set Coverage
Abnormality of the head	39	**1.1600e-26**	**1.9627e-24**	2.7511	137	17.41%	8	**2.4106e-4**	1.8312	74	1,607	13.75%
Abnormality of head or neck	40	**3.2823e-26**	**5.4151e-24**	2.7206	137	17.41%	11	**3.0947e-4**	1.8043	74	1,631	13.75%
Abnormality of the periorbital region	42	**2.6820e-9**	**4.2140e-7**	3.5644	31	3.94%	1	**4.8351e-4**	3.7181	23	246	4.28%
Abnormality of skin adnexa morphology	43	**7.9423e-9**	**1.2189e-6**	2.5174	49	6.23%	6	**2.3365e-4**	2.5144	38	601	7.06%
Abnormality of the urethra	44	**9.9646e-9**	**1.4945e-6**	4.5086	22	2.80%	30	**7.1423e-4**	4.4540	14	125	2.60%
Abnormality of the eyebrow	45	**2.3899e-8**	**3.5046e-6**	3.7052	26	3.30%	15	**3.1887e-4**	3.7969	19	199	3.53%
Abnormality of the hand	46	**3.2637e-8**	**4.6820e-6**	2.4032	49	6.23%	3	**2.6289e-4**	2.5342	39	612	7.25%
Cleft lip	47	**4.4126e-8**	**6.1954e-6**	4.7559	19	2.41%	42	**1.1262e-3**	4.8203	12	99	2.23%

The test set of 787 genomic regions picked 538 (3%) of all 21,395 genes.
Human Phenotype has 6,599 terms covering 3,215 (15%) of all 21,395 genes, and 244,972 term - gene associations.
6,599 ontology terms (100%) were tested using an annotation count range of [1, Inf].

⊖ Mouse Phenotype Single KO (20+ terms)

Global controls

Table controls: Export   Shown top rows in this table: 20 Set   Term annotation count: Min: 1 Max: Inf Set   Visualize this table: [select one]

Term Name	Binom Rank	Binom Raw P-Value	*Binom FDR Q-Val*	Binom Fold Enrichment	Binom Observed Region Hits	Binom Region Set Coverage	Hyper Rank	*Hyper FDR Q-Val*	Hyper Fold Enrichment	Hyper Observed Gene Hits	Hyper Total Genes	Hyper Gene Set Coverage
abnormal trachea morphology	13	**3.7820e-58**	**2.6678e-55**	20.8210	61	7.75%	111	**4.6035e-3**	5.1871	9	69	1.67%
abnormal digestive system morphology	19	**1.9968e-32**	**9.6373e-30**	3.7591	111	14.10%	21	**2.4122e-5**	2.3279	48	820	8.92%
digestive/alimentary phenotype	22	**3.4243e-31**	**1.4273e-28**	3.3585	122	15.50%	20	**2.3333e-5**	2.1445	57	1,057	10.59%
postnatal lethality, incomplete penetrance	24	**6.1193e-29**	**2.3381e-26**	4.1024	90	11.44%	69	**6.3329e-4**	2.3352	34	579	6.32%
abnormal brain development	26	**1.7542e-27**	**6.1868e-25**	3.9904	88	11.18%	158	**1.4155e-2**	2.0153	30	592	5.58%
lethality during fetal growth through weaning	28	**4.3656e-27**	**1.4297e-24**	2.5019	161	20.46%	7	**2.4442e-9**	2.1240	94	1,760	17.47%
abnormal respiratory system morphology	29	**2.4420e-26**	**7.7217e-24**	3.6251	94	11.94%	80	**1.0637e-3**	2.1217	39	731	7.25%
abnormal nervous system development	30	**6.9579e-25**	**2.1268e-22**	3.0816	109	13.85%	67	**6.1322e-4**	1.9967	48	956	8.92%
postnatal lethality	31	**1.7867e-23**	**5.2851e-21**	2.9933	107	13.60%	55	**3.5407e-4**	2.0340	49	958	9.11%
respiratory system phenotype	34	**1.9534e-22**	**5.2686e-20**	2.8097	112	14.23%	61	**4.3644e-4**	1.9186	55	1,140	10.22%
abnormal craniofacial morphology	45	**4.8215e-12**	**9.8251e-10**	2.3042	82	10.42%	1	**8.5832e-11**	2.8257	68	957	12.64%
craniofacial phenotype	46	**5.4121e-12**	**1.0789e-9**	2.2988	82	10.42%	2	**5.2573e-11**	2.8139	68	961	12.64%
abnormal palate morphology	47	**5.8652e-12**	**1.1443e-9**	3.8249	38	4.83%	8	**1.3741e-8**	4.6983	28	237	5.20%
abnormal head morphology	48	**1.0399e-11**	**1.9866e-9**	2.4677	70	8.89%	3	**9.4308e-11**	3.0713	58	751	10.78%
abnormal facial morphology	52	**3.3636e-10**	**5.9315e-8**	2.5148	58	7.37%	9	**2.0495e-8**	3.0539	46	599	8.55%
abnormal cranium morphology	54	**8.8416e-10**	**1.5014e-7**	2.4722	57	7.24%	12	**3.0486e-8**	3.0383	45	589	8.36%

Term Name	Binom Rank	Binom Raw P-Value	*Binom FDR Q-Val*	Binom Fold Enrichment	Binom Observed Region Hits	Binom Region Set Coverage	Hyper Rank	*Hyper FDR Q-Val*	Hyper Fold Enrichment	Hyper Observed Gene Hits	Hyper Total Genes	Hyper Gene Set Coverage
abnormal craniofacial bone morphology	56	**1.8483e-9**	**3.0266e-7**	2.4212	57	7.24%	11	**1.9860e-8**	3.0387	46	602	8.55%
abnormal axial skeleton morphology	57	**2.8223e-9**	**4.5405e-7**	2.0114	82	10.42%	10	**2.0437e-8**	2.3716	69	1,157	12.83%
abnormal mouth morphology	58	**3.8257e-9**	**6.0485e-7**	2.6751	46	5.84%	13	**3.1437e-7**	3.2911	36	435	6.69%
abnormal esophageal smooth muscle morphology	59	**2.8613e-8**	**4.4471e-6**	35.2635	6	0.76%	41	**8.8457e-5**	39.7677	4	4	0.74%

The test set of 787 genomic regions picked 538 (3%) of all 21,395 genes.
Mouse Phenotype Single KO has 9,170 terms covering 9,466 (44%) of all 21,395 genes, and 551,620 term - gene associations.
9,170 ontology terms (100%) were tested using an annotation count range of [1, Inf].

Mouse Phenotype (20+ terms)

Global controls

Table controls: Export Shown top rows in this table: 20 Set Term annotation count: Min: 1 Max: Inf Set Visualize this table: [select one]

Term Name	Binom Rank	Binom Raw P-Value	*Binom FDR Q-Val*	Binom Fold Enrichment	Binom Observed Region Hits	Binom Region Set Coverage	Hyper Rank	*Hyper FDR Q-Val*	Hyper Fold Enrichment	Hyper Observed Gene Hits	Hyper Total Genes	Hyper Gene Set Coverage
abnormal trachea morphology	14	**9.6276e-51**	**6.5846e-48**	15.5092	61	7.75%	242	**1.5873e-2**	4.0214	9	89	1.67%
abnormal digestive system morphology	18	**4.8410e-33**	**2.5751e-30**	3.3387	130	16.52%	17	**2.3039e-7**	2.3054	64	1,104	11.90%
digestive/alimentary phenotype	20	**3.8211e-31**	**1.8294e-28**	2.9982	141	17.92%	18	**8.2531e-7**	2.0930	73	1,387	13.57%
lethality during fetal growth through weaning	22	**2.1712e-29**	**9.4496e-27**	2.3459	193	24.52%	5	**2.6591e-13**	2.1310	124	2,314	23.05%
abnormal brain development	25	**1.6297e-28**	**6.2418e-26**	3.5322	105	13.34%	77	**1.6180e-4**	2.1736	44	805	8.18%
abnormal respiratory system morphology	29	**4.7671e-25**	**1.5740e-22**	3.1848	105	13.34%	65	**8.6502e-5**	2.1086	50	943	9.29%
postnatal lethality, incomplete penetrance	30	**1.2501e-24**	**3.9898e-22**	3.3154	98	12.45%	123	**1.2382e-3**	2.0280	41	804	7.62%
abnormal nervous system development	31	**1.1887e-23**	**3.6716e-21**	2.7189	124	15.76%	53	**4.3969e-5**	1.9741	62	1,249	11.52%
respiratory system phenotype	32	**1.3662e-23**	**4.0880e-21**	2.6326	130	16.52%	43	**1.4269e-5**	1.9467	70	1,430	13.01%
postnatal lethality	35	**4.0381e-22**	**1.1047e-19**	2.5671	127	16.14%	58	**5.3420e-5**	1.9088	66	1,375	12.27%
abnormal respiratory system physiology	36	**7.6612e-20**	**2.0377e-17**	2.8871	95	12.07%	319	**3.0685e-2**	1.6913	39	917	7.25%
abnormal brain morphology	40	**2.6930e-16**	**6.4464e-14**	2.1337	131	16.65%	199	**6.3061e-3**	1.5722	68	1,720	12.64%
abnormal head morphology	43	**8.1410e-15**	**1.8128e-12**	2.5195	87	11.05%	2	**2.7389e-13**	3.0328	71	931	13.20%
abnormal facial morphology	45	**3.8650e-13**	**8.2238e-11**	2.5691	74	9.40%	9	**5.7930e-11**	3.0309	58	761	10.78%
abnormal craniofacial morphology	46	**1.1567e-12**	**2.4076e-10**	2.2098	94	11.94%	6	**7.0677e-12**	2.6466	77	1,157	14.31%
craniofacial phenotype	47	**1.2888e-12**	**2.6256e-10**	2.2054	94	11.94%	7	**7.2856e-12**	2.6375	77	1,161	14.31%
abnormal palate morphology	48	**1.5346e-12**	**3.0612e-10**	3.4207	46	5.84%	11	**1.3958e-8**	3.9889	33	329	6.13%
abnormal mouth morphology	53	**6.6388e-10**	**1.1994e-7**	2.5430	55	6.99%	16	**1.6467e-7**	2.9986	42	557	7.81%
abnormal autopod morphology	56	**3.3224e-9**	**5.6807e-7**	2.8444	42	5.34%	32	**6.8015e-6**	3.1369	31	393	5.76%

Term Name	Binom Rank	Binom Raw P-Value	*Binom FDR Q-Val*	Binom Fold Enrichment	Binom Observed Region Hits	Binom Region Set Coverage	Hyper Rank	*Hyper FDR Q-Val*	Hyper Fold Enrichment	Hyper Observed Gene Hits	Hyper Total Genes	Hyper Gene Set Coverage
abnormal cranium morphology	58	**5.0886e-9**	**8.4006e-7**	2.2537	62	7.88%	12	**1.8607e-8**	2.8366	51	715	9.48%

The test set of 787 genomic regions picked 538 (3%) of all 21,395 genes.
Mouse Phenotype has 9,575 terms covering 9,654 (45%) of all 21,395 genes, and 705,265 term - gene associations.
9,575 ontology terms (100%) were tested using an annotation count range of [1, Inf].
