# Supplementary material for: AP-2α and AP-2β cooperatively function in the craniofacial surface ectoderm to regulate chromatin and gene expression dynamics during facial development
Source: eLife. 2022 Mar 25;11:e70511. doi: 10.7554/eLife.70511 (PMC9038197; doi:10.7554/eLife.70511)
Supplement: Figure 3—source data 5. [file elife-70511-fig3-data5.pdf]

Job Description

Region-Gene Association Graphs

What do these graphs illustrate?

Number of associated genes per region

Download as PDF.

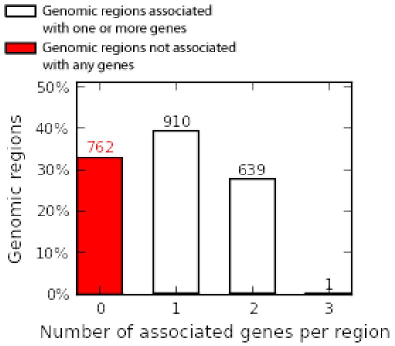

Binned by orientation and distance to TSS

Download as PDF.

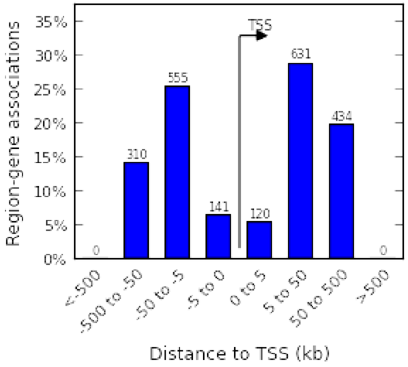

Binned by absolute distance to TSS

Download as PDF.

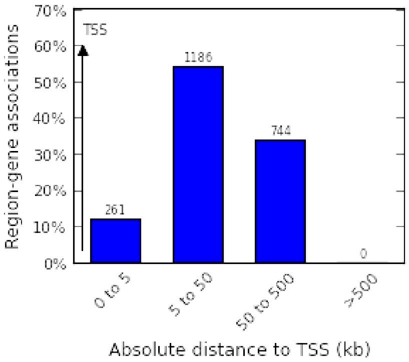

Global Controls

Global Export

Which data is exported by each option?

Ensembl Genes (no terms)

Global controls

GO Biological Process (20+ terms)

Global controls

Table controls: Export | Shown top rows in this table: 20 Set | Term annotation count: Min: 1 Max: Inf Set | Visualize this table: [select one]

Term Name	Binom Rank	Binom Raw P-Value	*Binom FDR Q-Val*	Binom Fold Enrichment	Binom Observed Region Hits	Binom Region Set Coverage	Hyper Rank	*Hyper FDR Q-Val*	Hyper Fold Enrichment	Hyper Observed Gene Hits	Hyper Total Genes	Hyper Gene Set Coverage
defense response to virus	50	**6.3478e-20**	**1.6618e-17**	4.7083	54	2.34%	231	**1.9948e-3**	2.0991	33	183	1.80%
response to virus	64	**1.9447e-18**	**3.9775e-16**	3.6989	65	2.81%	201	**8.5003e-4**	1.9706	43	254	2.34%
negative regulation of lipid biosynthetic process	82	**3.6241e-16**	**5.7853e-14**	5.8598	35	1.51%	376	**1.5700e-2**	2.7161	14	60	0.76%
liver development	87	**6.6572e-16**	**1.0016e-13**	3.7143	55	2.38%	461	**2.8844e-2**	1.9674	24	142	1.31%
hepaticobiliary system development	92	**1.7900e-15**	**2.5469e-13**	3.6250	55	2.38%	494	**3.6248e-2**	1.9267	24	145	1.31%
adult walking behavior	96	**5.0391e-15**	**6.8710e-13**	6.3877	30	1.30%	534	**4.9346e-2**	2.8391	10	41	0.54%
regulation of lipid biosynthetic process	97	**6.1487e-15**	**8.2975e-13**	3.7329	51	2.21%	230	**1.9070e-3**	2.2634	28	144	1.52%
immune effector process	101	**1.4329e-14**	**1.8571e-12**	2.6208	83	3.59%	321	**7.2370e-3**	1.6190	58	417	3.16%
gland development	116	**6.5626e-14**	**7.4056e-12**	2.1780	113	4.89%	114	**2.9502e-5**	1.8393	73	462	3.97%
regulation of autophagy	126	**3.4782e-13**	**3.6134e-11**	3.0576	58	2.51%	375	**1.5728e-2**	1.8238	34	217	1.85%
response to other organism	134	**8.2929e-13**	**8.1011e-11**	2.0455	119	5.15%	522	**4.4609e-2**	1.3498	93	802	5.06%

Term Name	Binom Rank	Binom Raw P-Value	*Binom FDR Q-Val*	Binom Fold Enrichment	Binom Observed Region Hits	Binom Region Set Coverage	Hyper Rank	*Hyper FDR Q-Val*	Hyper Fold Enrichment	Hyper Observed Gene Hits	Hyper Total Genes	Hyper Gene Set Coverage
striated muscle tissue development	149	**3.2438e-12**	**2.8498e-10**	2.3707	82	3.55%	168	**4.2732e-4**	1.9089	51	311	2.77%
natural killer cell activation involved in immune response	153	**4.9574e-12**	**4.2414e-10**	13.8653	14	0.61%	174	**4.9830e-4**	4.0899	13	37	0.71%
regulation of lipid metabolic process	161	**1.2273e-11**	**9.9786e-10**	2.4652	72	3.11%	316	**6.4589e-3**	1.7519	45	299	2.45%
muscle structure development	163	**1.4794e-11**	**1.1880e-9**	2.0539	105	4.54%	111	**1.8046e-5**	1.8457	75	473	4.08%
positive regulation of peptidyl-serine phosphorylation of STAT protein	166	**1.8209e-11**	**1.4359e-9**	12.5467	14	0.61%	159	**2.6610e-4**	4.3236	13	35	0.71%
muscle tissue development	186	**8.8044e-11**	**6.1962e-9**	2.2120	82	3.55%	223	**1.5694e-3**	1.8044	51	329	2.77%
regulation of peptidyl-serine phosphorylation of STAT protein	188	**1.0504e-10**	**7.3139e-9**	10.9525	14	0.61%	174	**4.9830e-4**	4.0899	13	37	0.71%
muscle cell differentiation	190	**1.1610e-10**	**7.9989e-9**	2.3244	73	3.16%	206	**1.0305e-3**	1.9055	46	281	2.50%
developmental growth	197	**1.5658e-10**	**1.0404e-8**	2.0672	93	4.02%	48	**2.2512e-7**	2.1033	73	404	3.97%

The test set of 2,312 genomic regions picked 1,838 (9%) of all 21,395 genes.
GO Biological Process has 13,090 terms covering 17,925 (84%) of all 21,395 genes, and 1,163,819 term - gene associations.
13,090 ontology terms (100%) were tested using an annotation count range of [1, Inf].

GO Cellular Component (3 terms)

Global controls

Table controls: Export Shown top rows in this table: 20 Set Term annotation count: Min: 1 Max: Inf Set Visualize this table: [select one]

Term Name	Binom Rank	Binom Raw P-Value	*Binom FDR Q-Val*	Binom Fold Enrichment	Binom Observed Region Hits	Binom Region Set Coverage	Hyper Rank	*Hyper FDR Q-Val*	Hyper Fold Enrichment	Hyper Observed Gene Hits	Hyper Total Genes	Hyper Gene Set Coverage
endoplasmic reticulum lumen	19	**3.8820e-11**	**3.4611e-9**	3.8713	35	1.51%	13	**4.8844e-3**	2.4790	23	108	1.25%
cell-cell adherens junction	59	**9.6164e-5**	**2.7610e-3**	2.3327	26	1.12%	20	**1.8338e-2**	2.3756	20	98	1.09%
dynein complex	60	**9.6318e-5**	**2.7194e-3**	2.9761	17	0.74%	32	**3.9765e-2**	2.7022	13	56	0.71%

The test set of 2,312 genomic regions picked 1,838 (9%) of all 21,395 genes.
GO Cellular Component has 1,694 terms covering 19,074 (89%) of all 21,395 genes, and 371,380 term - gene associations.
1,694 ontology terms (100%) were tested using an annotation count range of [1, Inf].

GO Molecular Function (1 term)

Global controls

Table controls: Export Shown top rows in this table: 20 Set Term annotation count: Min: 1 Max: Inf Set Visualize this table: [select one]

Term Name	Binom Rank	Binom Raw P-Value	*Binom FDR Q-Val*	Binom Fold Enrichment	Binom Observed Region Hits	Binom Region Set Coverage	Hyper Rank	*Hyper FDR Q-Val*	Hyper Fold Enrichment	Hyper Observed Gene Hits	Hyper Total Genes	Hyper Gene Set Coverage
type I interferon receptor binding	6	**1.3170e-15**	**9.0676e-13**	25.7455	14	0.61%	3	**8.6258e-4**	4.8814	13	31	0.71%

The test set of 2,312 genomic regions picked 1,838 (9%) of all 21,395 genes.
GO Molecular Function has 4,131 terms covering 17,189 (80%) of all 21,395 genes, and 227,341 term - gene associations.
4,131 ontology terms (100%) were tested using an annotation count range of [1, Inf].

Human Phenotype (1 term)

Global controls

Table controls: Export Shown top rows in this table: 20 Set Term annotation count: Min: 1 Max: Inf Set Visualize this table: [select one]

Term Name	Binom Rank	Binom Raw P-Value	*Binom FDR Q-Val*	Binom Fold Enrichment	Binom Observed Region Hits	Binom Region Set Coverage	Hyper Rank	*Hyper FDR Q-Val*	Hyper Fold Enrichment	Hyper Observed Gene Hits	Hyper Total Genes	Hyper Gene Set Coverage
Cleft lip	85	**2.1187e-4**	**1.6449e-2**	2.2154	26	1.12%	1	**1.5987e-2**	2.8219	24	99	1.31%

The test set of 2,312 genomic regions picked 1,838 (9%) of all 21,395 genes.
Human Phenotype has 6,599 terms covering 3,215 (15%) of all 21,395 genes, and 244,972 term - gene associations.
6,599 ontology terms (100%) were tested using an annotation count range of [1, Inf].

Mouse Phenotype Single KO (20+ terms)

Global controls

Table controls: Export Shown top rows in this table: 20 Set Term annotation count: Min: 1 Max: Inf Set Visualize this table: [select one]

Term Name	Binom Rank	Binom Raw P-Value	*Binom FDR Q-Val*	Binom Fold Enrichment	Binom Observed Region Hits	Binom Region Set Coverage	Hyper Rank	*Hyper FDR Q-Val*	Hyper Fold Enrichment	Hyper Observed Gene Hits	Hyper Total Genes	Hyper Gene Set Coverage
decreased triglyceride level	71	**4.2967e-6**	**5.5494e-4**	2.0607	48	2.08%	117	**3.0291e-3**	1.9023	42	257	2.29%
dilated capillary	113	**5.2157e-5**	**4.2326e-3**	6.3357	8	0.35%	95	**1.7719e-3**	6.7902	7	12	0.38%
abnormal renal glomerulus basement membrane morphology	121	**7.5670e-5**	**5.7346e-3**	3.4961	14	0.61%	77	**7.8882e-4**	4.0899	13	37	0.71%
increased energy expenditure	134	**9.8587e-5**	**6.7466e-3**	2.1767	30	1.30%	159	**7.7384e-3**	2.2046	25	132	1.36%
abnormal vitreous body morphology	138	**1.0392e-4**	**6.9052e-3**	2.5321	22	0.95%	47	**7.6026e-5**	3.3951	21	72	1.14%
decreased heart rate	140	**1.1955e-4**	**7.8307e-3**	2.1851	29	1.25%	121	**3.3115e-3**	2.4562	23	109	1.25%
abnormal cornea morphology	142	**1.2133e-4**	**7.8355e-3**	2.0147	35	1.51%	84	**1.0998e-3**	2.2575	32	165	1.74%
abnormal cranial suture morphology	145	**1.2788e-4**	**8.0875e-3**	2.8018	18	0.78%	48	**1.5940e-4**	3.8055	17	52	0.92%
abnormal Harderian gland morphology	149	**1.4167e-4**	**8.7191e-3**	4.8236	9	0.39%	115	**2.9353e-3**	6.2679	7	13	0.38%
cataract	172	**2.4368e-4**	**1.2992e-2**	2.0916	29	1.25%	175	**1.0164e-2**	2.1556	25	135	1.36%
increased mean systemic arterial blood pressure	182	**3.2917e-4**	**1.6585e-2**	3.0218	14	0.61%	133	**4.6648e-3**	3.5817	12	39	0.65%
abnormal aortic valve morphology	192	**3.8090e-4**	**1.8192e-2**	2.7354	16	0.69%	256	**2.2398e-2**	2.7514	13	55	0.71%
abnormal bronchiole morphology	196	**4.0788e-4**	**1.9083e-2**	3.5165	11	0.48%	172	**9.3771e-3**	3.7550	10	31	0.54%
cachexia	213	**5.4542e-4**	**2.3481e-2**	2.1506	24	1.04%	180	**1.0439e-2**	2.3281	21	105	1.14%
thick epidermis	215	**5.4711e-4**	**2.3335e-2**	2.4748	18	0.78%	107	**2.3473e-3**	2.9101	18	72	0.98%
abnormal mammary gland lobule morphology	227	**6.1783e-4**	**2.4958e-2**	3.5981	10	0.43%	224	**1.8089e-2**	4.0488	8	23	0.44%

Term Name	Binom Rank	Binom Raw P-Value	Binom FDR Q-Val	Binom Fold Enrichment	Binom Observed Region Hits	Binom Region Set Coverage	Hyper Rank	Hyper FDR Q-Val	Hyper Fold Enrichment	Hyper Observed Gene Hits	Hyper Total Genes	Hyper Gene Set Coverage
increased fatty acid level	229	**6.2289e-4**	**2.4943e-2**	2.2148	22	0.95%	264	**2.2044e-2**	2.1963	20	106	1.09%
abnormal vein development	235	**6.5484e-4**	**2.5553e-2**	2.5116	17	0.74%	204	**1.3946e-2**	2.8097	14	58	0.76%
decreased ventral retina size	237	**6.6314e-4**	**2.5658e-2**	10.4300	4	0.17%	257	**2.2589e-2**	11.6404	3	3	0.16%
dilated vasculature	240	**6.7314e-4**	**2.5720e-2**	3.3063	11	0.48%	318	**4.1559e-2**	3.1746	9	33	0.49%

The test set of 2,312 genomic regions picked 1,838 (9%) of all 21,395 genes.
Mouse Phenotype Single KO has 9,170 terms covering 9,466 (44%) of all 21,395 genes, and 551,620 term - gene associations.
9,170 ontology terms (100%) were tested using an annotation count range of [1, Inf].

Mouse Phenotype (20+ terms)

Global controls

Table controls: Export Shown top rows in this table: 20 Set Term annotation count: Min: 1 Max: Inf Set Visualize this table: [select one]

Term Name	Binom Rank	Binom Raw P-Value	Binom FDR Q-Val	Binom Fold Enrichment	Binom Observed Region Hits	Binom Region Set Coverage	Hyper Rank	Hyper FDR Q-Val	Hyper Fold Enrichment	Hyper Observed Gene Hits	Hyper Total Genes	Hyper Gene Set Coverage
increased regulatory T cell number	30	**1.6666e-15**	**5.3191e-13**	5.5609	35	1.51%	360	**1.0307e-2**	2.6455	15	66	0.82%
curly tail	33	**2.7617e-15**	**8.0132e-13**	5.1480	37	1.60%	283	**5.5265e-3**	2.7389	16	68	0.87%
increased T cell number	51	**1.0786e-13**	**2.0250e-11**	2.1969	109	4.71%	274	**5.2424e-3**	1.5520	70	525	3.81%
abnormal regulatory T cell number	54	**1.9140e-13**	**3.3939e-11**	3.5094	49	2.12%	262	**4.6308e-3**	2.1380	27	147	1.47%
decreased bone marrow cell number	63	**4.4227e-13**	**6.7219e-11**	3.3268	51	2.21%	548	**2.7943e-2**	1.8775	25	155	1.36%
abnormal regulatory T cell morphology	64	**4.8605e-13**	**7.2717e-11**	3.4183	49	2.12%	293	**5.8651e-3**	2.0953	27	150	1.47%
abnormal brainstem morphology	71	**1.6188e-12**	**2.1831e-10**	2.5798	72	3.11%	91	**6.4357e-5**	2.1645	45	242	2.45%
increased astrocyte number	72	**1.6201e-12**	**2.1546e-10**	3.6492	43	1.86%	403	**1.4262e-2**	2.0754	23	129	1.25%
abnormal circulating amino acid level	75	**2.5385e-12**	**3.2408e-10**	2.7605	63	2.72%	589	**3.4803e-2**	1.6562	35	246	1.90%
abnormal astrocyte number	76	**3.4342e-12**	**4.3267e-10**	3.5635	43	1.86%	455	**1.7569e-2**	2.0282	23	132	1.25%
astrocytosis	77	**3.6121e-12**	**4.4916e-10**	3.6243	42	1.82%	468	**1.9101e-2**	2.0487	22	125	1.20%
hair follicle degeneration	84	**6.7679e-12**	**7.7146e-10**	7.1901	21	0.91%	168	**8.3775e-4**	4.4153	11	29	0.60%
abnormal midbrain morphology	87	**1.5788e-11**	**1.7376e-9**	2.8984	54	2.34%	153	**5.9117e-4**	2.2984	31	157	1.69%
abnormal bone marrow cell number	105	**6.6438e-11**	**6.0585e-9**	2.6678	58	2.51%	586	**3.4553e-2**	1.7009	32	219	1.74%
abnormal amino acid level	114	**1.0618e-10**	**8.9178e-9**	2.3965	69	2.98%	705	**4.7832e-2**	1.5445	41	309	2.23%
increased cell proliferation	116	**1.4946e-10**	**1.2337e-8**	2.4516	65	2.81%	321	**7.5403e-3**	1.7944	39	253	2.12%
decreased B cell number	120	**1.8557e-10**	**1.4807e-8**	2.0331	96	4.15%	248	**3.8275e-3**	1.5972	66	481	3.59%
abnormal tail morphology	130	**5.0542e-10**	**3.7226e-8**	2.0556	89	3.85%	158	**6.5438e-4**	1.7475	62	413	3.37%
small hair follicles	142	**1.1134e-9**	**7.5075e-8**	9.0952	14	0.61%	416	**1.5040e-2**	4.9887	6	14	0.33%
neuron degeneration	147	**1.7857e-9**	**1.1631e-7**	2.0803	81	3.50%	282	**5.3582e-3**	1.6501	55	388	2.99%

The test set of 2,312 genomic regions picked 1,838 (9%) of all 21,395 genes.
Mouse Phenotype has 9,575 terms covering 9,654 (45%) of all 21,395 genes, and 705,265 term - gene associations.
9,575 ontology terms (100%) were tested using an annotation count range of [1, Inf].
