# Supplementary material for: AP-2α and AP-2β cooperatively function in the craniofacial surface ectoderm to regulate chromatin and gene expression dynamics during facial development
Source: eLife. 2022 Mar 25;11:e70511. doi: 10.7554/eLife.70511 (PMC9038197; doi:10.7554/eLife.70511)
Supplement: Figure 3—source data 6. [file elife-70511-fig3-data6.pdf]

Homer Known Motif Enrichment Results (/Users/vanottee/output/)

Homer *de novo* Motif Results
Gene Ontology Enrichment Results
Known Motif Enrichment Results (txt file)
Total Target Sequences = 1454, Total Background Sequences = 47894

Rank	Motif	Name	P-value	log P-pvalue	q-value (Benjamini)	# Target Sequences with Motif	% of Targets Sequences with Motif	# Background Sequences with Motif	% of Background Sequences with Motif	Motif File	SVG
1	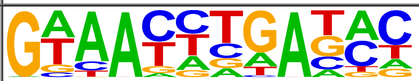	Six2(Homeobox)/NephronProgenitor-Six2-ChIP-Seq(GSE39837)/Homer	1e-97	-2.248e+02	0.0000	529.0	36.38%	6796.1	14.19%	motif file (matrix)	svg
2	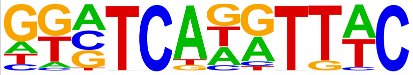	Six1(Homeobox)/Myoblast-Six1-ChIP-Chip(GSE20150)/Homer	1e-78	-1.818e+02	0.0000	227.0	15.61%	1644.2	3.43%	motif file (matrix)	svg
3	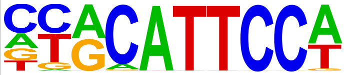	TEAD1(TEAD)/HepG2-TEAD1-ChIP-Seq(Encode)/Homer	1e-48	-1.111e+02	0.0000	417.0	28.68%	6629.1	13.84%	motif file (matrix)	svg
4	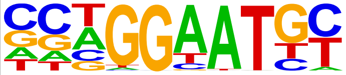	TEAD4(TEA)/Tropoblast-Tead4-ChIP-Seq(GSE37350)/Homer	1e-44	-1.020e+02	0.0000	378.0	26.00%	5929.0	12.38%	motif file (matrix)	svg
5	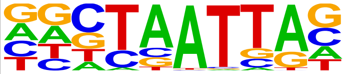	En1(Homeobox)/SUM149-EN1-ChIP-Seq(GSE120957)/Homer	1e-43	-9.969e+01	0.0000	624.0	42.92%	12486.3	26.07%	motif file (matrix)	svg
6	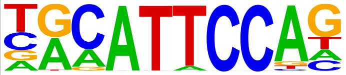	TEAD3(TEA)/HepG2-TEAD3-ChIP-Seq(Encode)/Homer	1e-43	-9.954e+01	0.0000	450.0	30.95%	7779.1	16.24%	motif file (matrix)	svg
7	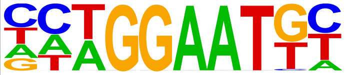	TEAD(TEA)/Fibroblast-PU.1-ChIP-Seq(Unpublished)/Homer	1e-38	-8.836e+01	0.0000	295.0	20.29%	4337.0	9.05%	motif file (matrix)	svg
8	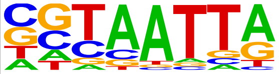	DLX5(Homeobox)/BasalGanglia-Dlx5-ChIP-seq(GSE124936)/Homer	1e-36	-8.342e+01	0.0000	323.0	22.21%	5114.0	10.68%	motif file (matrix)	svg
9	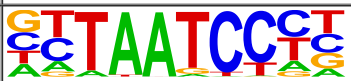	Otx2(Homeobox)/EpiLC-Otx2-ChIP-Seq(GSE56098)/Homer	1e-35	-8.289e+01	0.0000	361.0	24.83%	6047.9	12.63%	motif file (matrix)	svg
10	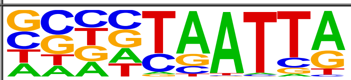	DLX1(Homeobox)/BasalGanglia-Dlx1-ChIP-seq(GSE124936)/Homer	1e-34	-7.918e+01	0.0000	468.0	32.19%	8930.5	18.64%	motif file (matrix)	svg
11	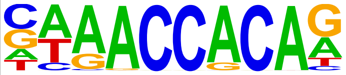	RUNX(Runt)/HPC7-Runx1-ChIP-Seq(GSE22178)/Homer	1e-33	-7.606e+01	0.0000	323.0	22.21%	5320.1	11.11%	motif file (matrix)	svg
12	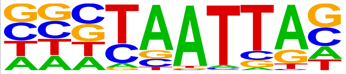	DLX2(Homeobox)/BasalGanglia-Dlx2-ChIP-seq(GSE124936)/Homer	1e-32	-7.508e+01	0.0000	500.0	34.39%	9958.8	20.79%	motif file (matrix)	svg
13	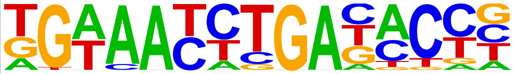	Six4(Homeobox)/MCF7-SIX4-ChIP-Seq(Encode)/Homer	1e-31	-7.279e+01	0.0000	67.0	4.61%	339.8	0.71%	motif file (matrix)	svg
14	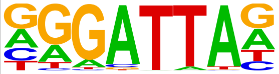	GSC(Homeobox)/FrogEmbryos-GSC-ChIP-Seq(DRA000576)/Homer	1e-30	-7.057e+01	0.0000	443.0	30.47%	8581.3	17.91%	motif file (matrix)	svg
15		Dlx3(Homeobox)/Kerainocytes-Dlx3-ChIP-Seq(GSE89884)/Homer	1e-29	-6.721e+01	0.0000	266.0	18.29%	4213.1	8.80%	motif file	svg

	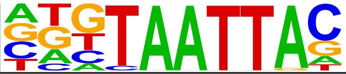									(matrix)	
16	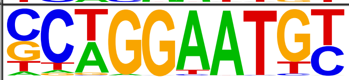	TEAD2(TEA)/Py2T-Tead2-ChIP-Seq(GSE55709)/Homer	1e-29	-6.704e+01	0.0000	241.0	16.57%	3641.0	7.60%	motif file (matrix)	svg
17	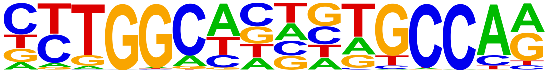	NF1(CTF)/LNCAP-NF1-ChIP-Seq(Unpublished)/Homer	1e-27	-6.240e+01	0.0000	218.0	14.99%	3238.3	6.76%	motif file (matrix)	svg
18	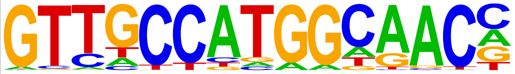	Rfx2(HTH)/LoVo-RFX2-ChIP-Seq(GSE49402)/Homer	1e-26	-6.136e+01	0.0000	100.0	6.88%	906.5	1.89%	motif file (matrix)	svg
19	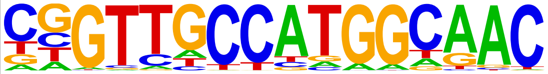	RFX(HTH)/K562-RFX3-ChIP-Seq(SRA012198)/Homer	1e-26	-6.124e+01	0.0000	95.0	6.53%	826.2	1.72%	motif file (matrix)	svg
20	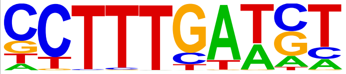	LEF1(HMG)/H1-LEF1-ChIP-Seq(GSE64758)/Homer	1e-26	-6.066e+01	0.0000	271.0	18.64%	4522.3	9.44%	motif file (matrix)	svg
21	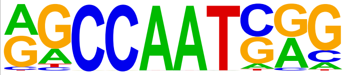	NFY(CCAAT)/Promoter/Homer	1e-24	-5.725e+01	0.0000	385.0	26.48%	7551.9	15.77%	motif file (matrix)	svg
22	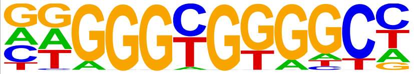	KLF1(Zf)/HUDEP2-KLF1-CutnRun(GSE136251)/Homer	1e-24	-5.706e+01	0.0000	583.0	40.10%	13151.1	27.45%	motif file (matrix)	svg
23	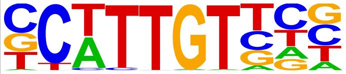	Sox10(HMG)/SciaticNerve-Sox3-ChIP-Seq(GSE35132)/Homer	1e-24	-5.677e+01	0.0000	527.0	36.24%	11528.2	24.07%	motif file (matrix)	svg
24	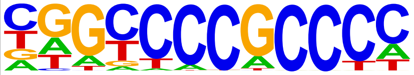	Sp2(Zf)/HEK293-Sp2.eGFP-ChIP-Seq(Encode)/Homer	1e-24	-5.543e+01	0.0000	769.0	52.89%	18963.0	39.59%	motif file (matrix)	svg
25	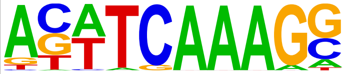	Tcf3(HMG)/mES-Tcf3-ChIP-Seq(GSE11724)/Homer	1e-23	-5.388e+01	0.0000	135.0	9.28%	1661.0	3.47%	motif file (matrix)	svg
26	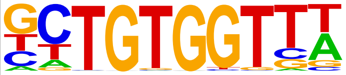	RUNX-AML(Runt)/CD4+-PolII-ChIP-Seq(Barski_et_al.)/Homer	1e-23	-5.341e+01	0.0000	280.0	19.26%	4977.8	10.39%	motif file (matrix)	svg
27	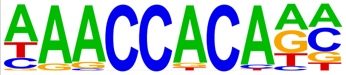	RUNX1(Runt)/Jurkat-RUNX1-ChIP-Seq(GSE29180)/Homer	1e-22	-5.265e+01	0.0000	383.0	26.34%	7689.9	16.05%	motif file (matrix)	svg
28	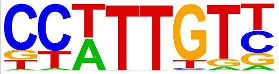	Sox3(HMG)/NPC-Sox3-ChIP-Seq(GSE33059)/Homer	1e-22	-5.241e+01	0.0000	539.0	37.07%	12098.7	25.26%	motif file (matrix)	svg
29	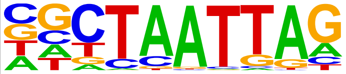	LXH9(Homeobox)/Hct116-LXH9.V5-ChIP-Seq(GSE116822)/Homer	1e-22	-5.197e+01	0.0000	450.0	30.95%	9567.3	19.97%	motif file (matrix)	svg
30	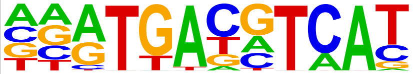	CREB5(bZIP)/LNCaP-CREB5.V5-ChIP-Seq(GSE137775)/Homer	1e-22	-5.173e+01	0.0000	169.0	11.62%	2416.2	5.04%	motif file (matrix)	svg
31	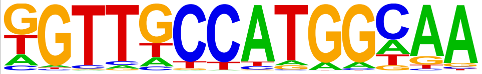	X-box(HTH)/NPC-H3K4me1-ChIP-Seq(GSE16256)/Homer	1e-22	-5.148e+01	0.0000	92.0	6.33%	898.4	1.88%	motif file (matrix)	svg
32	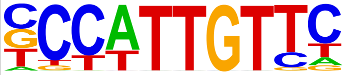	Sox2(HMG)/mES-Sox2-ChIP-Seq(GSE11431)/Homer	1e-22	-5.098e+01	0.0000	328.0	22.56%	6295.3	13.14%	motif file (matrix)	svg
33	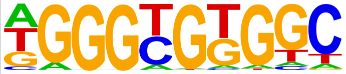	KLF5(Zf)/LoVo-KLF5-ChIP-Seq(GSE49402)/Homer	1e-21	-5.051e+01	0.0000	702.0	48.28%	17140.3	35.78%	motif file (matrix)	svg

34	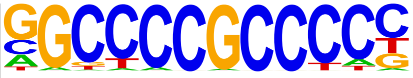	Sp1(Zf)/Promoter/Homer	1e-21	-4.988e+01	0.0000	345.0	23.73%	6788.8	14.17%	motif file (matrix)	svg
35	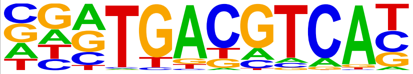	Atf7(bZIP)/3T3L1-Atf7-ChIP-Seq(GSE56872)/Homer	1e-21	-4.952e+01	0.0000	208.0	14.31%	3357.4	7.01%	motif file (matrix)	svg
36	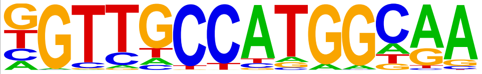	Rfx1(HTH)/NPC-H3K4me1-ChIP-Seq(GSE16256)/Homer	1e-20	-4.687e+01	0.0000	131.0	9.01%	1719.6	3.59%	motif file (matrix)	svg
37	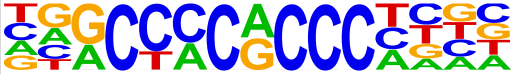	KLF3(Zf)/MEF-Klf3-ChIP-Seq(GSE44748)/Homer	1e-20	-4.653e+01	0.0000	394.0	27.10%	8265.4	17.25%	motif file (matrix)	svg
38	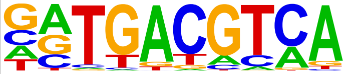	Atf1(bZIP)/K562-ATF1-ChIP-Seq(GSE31477)/Homer	1e-20	-4.613e+01	0.0000	259.0	17.81%	4708.4	9.83%	motif file (matrix)	svg
39	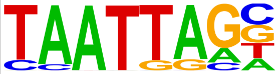	Lhx2(Homeobox)/HFSC-Lhx2-ChIP-Seq(GSE48068)/Homer	1e-19	-4.545e+01	0.0000	346.0	23.80%	7005.9	14.63%	motif file (matrix)	svg
40	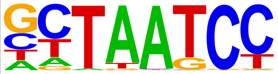	CRX(Homeobox)/Retina-Crx-ChIP-Seq(GSE20012)/Homer	1e-19	-4.511e+01	0.0000	671.0	46.15%	16506.4	34.46%	motif file (matrix)	svg
41	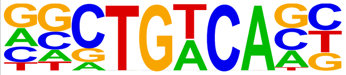	Meis1(Homeobox)/MastCells-Meis1-ChIP-Seq(GSE48085)/Homer	1e-19	-4.457e+01	0.0000	567.0	39.00%	13363.2	27.90%	motif file (matrix)	svg
42	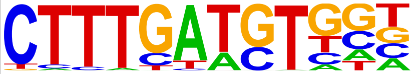	Tcf7(HMG)/GM12878-TCF7-ChIP-Seq(Encode)/Homer	1e-19	-4.388e+01	0.0000	149.0	10.25%	2170.5	4.53%	motif file (matrix)	svg
43	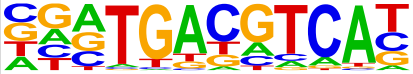	Atf2(bZIP)/3T3L1-Atf2-ChIP-Seq(GSE56872)/Homer	1e-18	-4.192e+01	0.0000	155.0	10.66%	2353.4	4.91%	motif file (matrix)	svg
44	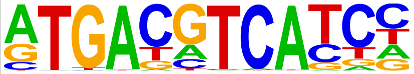	c-Jun-CRE(bZIP)/K562-cJun-ChIP-Seq(GSE31477)/Homer	1e-17	-4.092e+01	0.0000	142.0	9.77%	2090.3	4.36%	motif file (matrix)	svg
45	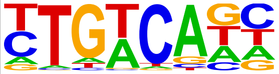	Tgif1(Homeobox)/mES-Tgif1-ChIP-Seq(GSE55404)/Homer	1e-17	-4.089e+01	0.0000	792.0	54.47%	20634.1	43.07%	motif file (matrix)	svg
46	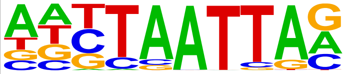	Lhx3(Homeobox)/Neuron-Lhx3-ChIP-Seq(GSE31456)/Homer	1e-17	-4.073e+01	0.0000	477.0	32.81%	10928.0	22.81%	motif file (matrix)	svg
47	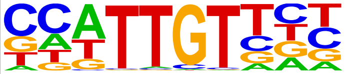	Sox6(HMG)/Myotubes-Sox6-ChIP-Seq(GSE32627)/Homer	1e-17	-4.052e+01	0.0000	478.0	32.87%	10968.9	22.90%	motif file (matrix)	svg
48	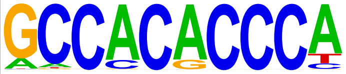	Klf4(Zf)/mES-Klf4-ChIP-Seq(GSE11431)/Homer	1e-17	-3.930e+01	0.0000	260.0	17.88%	4996.6	10.43%	motif file (matrix)	svg
49	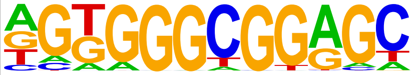	Sp5(Zf)/mES-Sp5.Flag-ChIP-Seq(GSE72989)/Homer	1e-17	-3.921e+01	0.0000	612.0	42.09%	15064.9	31.45%	motif file (matrix)	svg
50	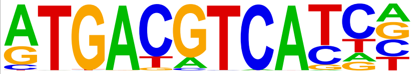	JunD(bZIP)/K562-JunD-ChIP-Seq/Homer	1e-16	-3.864e+01	0.0000	72.0	4.95%	729.2	1.52%	motif file (matrix)	svg
51	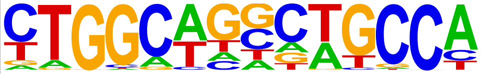	Tlx?(NR)/NPC-H3K4me1-ChIP-Seq(GSE16256)/Homer	1e-16	-3.815e+01	0.0000	196.0	13.48%	3429.9	7.16%	motif file (matrix)	svg
52	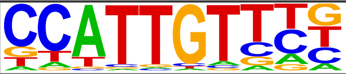	Sox17(HMG)/Endoderm-Sox17-ChIP-Seq(GSE61475)/Homer	1e-16	-3.743e+01	0.0000	260.0	17.88%	5074.9	10.59%	motif file	svg

										(matrix)	
53	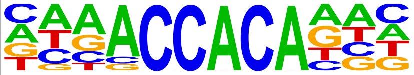	RUNX2(Runt)/PCa-RUNX2-ChIP-Seq(GSE33889)/Homer	1e-15	-3.589e+01	0.0000	298.0	20.50%	6151.1	12.84%	motif file (matrix)	svg
54	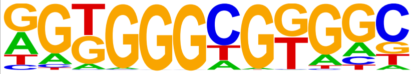	KLF14(Zf)/HEK293-KLF14.GFP-ChIP-Seq(GSE58341)/Homer	1e-15	-3.489e+01	0.0000	811.0	55.78%	21695.0	45.29%	motif file (matrix)	svg
55	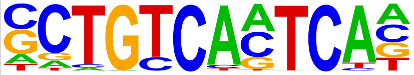	Pbx3(Homeobox)/GM12878-PBX3-ChIP-Seq(GSE32465)/Homer	1e-15	-3.455e+01	0.0000	127.0	8.73%	1923.4	4.02%	motif file (matrix)	svg
56	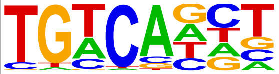	Tgif2(Homeobox)/mES-Tgif2-ChIP-Seq(GSE55404)/Homer	1e-14	-3.451e+01	0.0000	830.0	57.08%	22348.1	46.65%	motif file (matrix)	svg
57	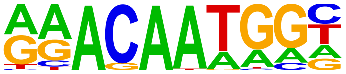	Sox15(HMG)/CPA-Sox15-ChIP-Seq(GSE62909)/Homer	1e-14	-3.400e+01	0.0000	338.0	23.25%	7342.1	15.33%	motif file (matrix)	svg
58	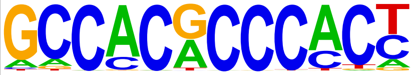	Klf9(Zf)/GBM-Klf9-ChIP-Seq(GSE62211)/Homer	1e-14	-3.349e+01	0.0000	319.0	21.94%	6843.6	14.29%	motif file (matrix)	svg
59	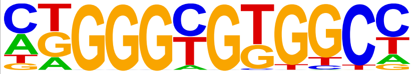	KLF6(Zf)/PDAC-KLF6-ChIP-Seq(GSE64557)/Homer	1e-14	-3.287e+01	0.0000	577.0	39.68%	14425.6	30.11%	motif file (matrix)	svg
60	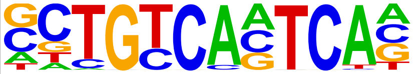	Pknox1(Homeobox)/ES-Prep1-ChIP-Seq(GSE63282)/Homer	1e-12	-2.849e+01	0.0000	120.0	8.25%	1935.4	4.04%	motif file (matrix)	svg
61	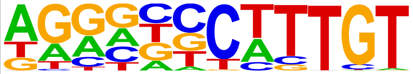	Sox9(HMG)/Limb-SOX9-ChIP-Seq(GSE73225)/Homer	1e-12	-2.784e+01	0.0000	288.0	19.81%	6292.0	13.13%	motif file (matrix)	svg
62	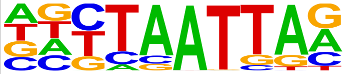	Lhx1(Homeobox)/EmbryoCarcinoma-Lhx1-ChIP-Seq(GSE70957)/Homer	1e-12	-2.770e+01	0.0000	326.0	22.42%	7361.1	15.37%	motif file (matrix)	svg
63	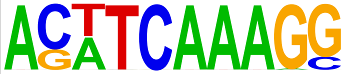	TCFL2(HMG)/K562-TCF7L2-ChIP-Seq(GSE29196)/Homer	1e-11	-2.686e+01	0.0000	52.0	3.58%	548.7	1.15%	motif file (matrix)	svg
64	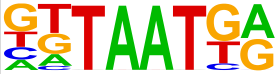	Nkx6.1(Homeobox)/Islet-Nkx6.1-ChIP-Seq(GSE40975)/Homer	1e-11	-2.649e+01	0.0000	621.0	42.71%	16282.1	33.99%	motif file (matrix)	svg
65	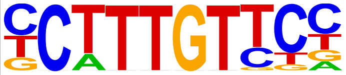	Sox4(HMG)/proB-Sox4-ChIP-Seq(GSE50066)/Homer	1e-11	-2.552e+01	0.0000	271.0	18.64%	5952.0	12.43%	motif file (matrix)	svg
66	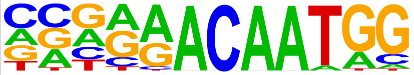	Sox7(HMG)/ESC-Sox7-ChIP-Seq(GSE133899)/Homer	1e-10	-2.322e+01	0.0000	120.0	8.25%	2106.6	4.40%	motif file (matrix)	svg
67	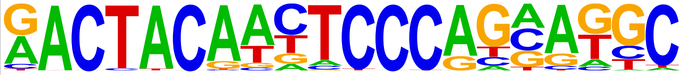	Ronin(THAP)/ES-Thap11-ChIP-Seq(GSE51522)/Homer	1e-9	-2.302e+01	0.0000	36.0	2.48%	326.3	0.68%	motif file (matrix)	svg
68	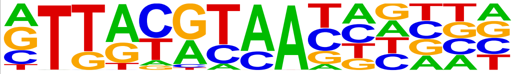	NFIL3(bZIP)/HepG2-NFIL3-ChIP-Seq(Encode)/Homer	1e-9	-2.268e+01	0.0000	189.0	13.00%	3882.0	8.10%	motif file (matrix)	svg
69	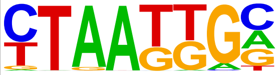	Isl1(Homeobox)/Neuron-Isl1-ChIP-Seq(GSE31456)/Homer	1e-9	-2.169e+01	0.0000	511.0	35.14%	13277.8	27.72%	motif file (matrix)	svg
70	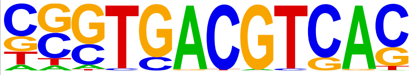	CRE(bZIP)/Promoter/Homer	1e-9	-2.149e+01	0.0000	125.0	8.60%	2292.2	4.79%	motif file (matrix)	svg

71	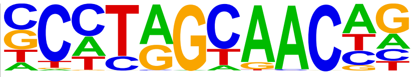	Rfx5(HTH)/GM12878-Rfx5-ChIP-Seq(GSE31477)/Homer	1e-8	-2.002e+01	0.0000	151.0	10.39%	3013.3	6.29%	motif file (matrix)	svg
72	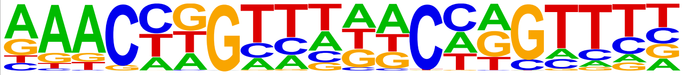	GRHL2(CP2)/HBE-GRHL2-ChIP-Seq(GSE46194)/Homer	1e-8	-1.851e+01	0.0000	132.0	9.08%	2592.6	5.41%	motif file (matrix)	svg
73	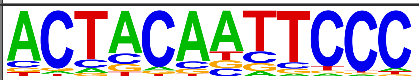	GFY(?)/Promoter/Homer	1e-7	-1.829e+01	0.0000	46.0	3.16%	583.1	1.22%	motif file (matrix)	svg
74	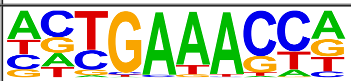	IRF4(IRF)/GM12878-IRF4-ChIP-Seq(GSE32465)/Homer	1e-7	-1.776e+01	0.0000	138.0	9.49%	2782.3	5.81%	motif file (matrix)	svg
75	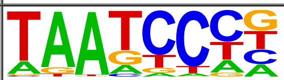	Pitx1(Homeobox)/Chicken-Pitx1-ChIP-Seq(GSE38910)/Homer	1e-7	-1.720e+01	0.0000	910.0	62.59%	26617.9	55.57%	motif file (matrix)	svg
76	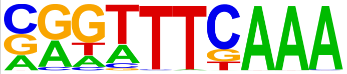	CHR(?)/Hela-CellCycle-Expression/Homer	1e-7	-1.679e+01	0.0000	171.0	11.76%	3710.9	7.75%	motif file (matrix)	svg
77	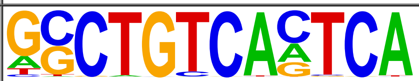	PBX1(Homeobox)/MCF7-PBX1-ChIP-Seq(GSE28007)/Homer	1e-6	-1.585e+01	0.0000	48.0	3.30%	677.8	1.41%	motif file (matrix)	svg
78	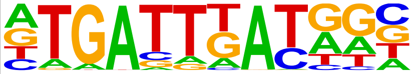	PBX2(Homeobox)/K562-PBX2-ChIP-Seq(Encode)/Homer	1e-6	-1.560e+01	0.0000	223.0	15.34%	5230.9	10.92%	motif file (matrix)	svg
79	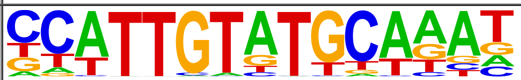	Oct4:Sox17(POU,Homeobox,HMG)/F9-Sox17-ChIP-Seq(GSE44553)/Homer	1e-6	-1.524e+01	0.0000	50.0	3.44%	735.9	1.54%	motif file (matrix)	svg
80	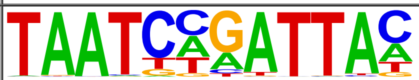	Pax7(Paired,Homeobox),long/Myoblast-Pax7-ChIP-Seq(GSE25064)/Homer	1e-6	-1.489e+01	0.0000	17.0	1.17%	121.7	0.25%	motif file (matrix)	svg
81	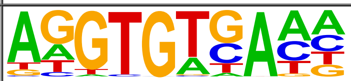	Tbet(T-box)/CD8-Tbet-ChIP-Seq(GSE33802)/Homer	1e-6	-1.474e+01	0.0000	277.0	19.05%	6851.2	14.30%	motif file (matrix)	svg
82	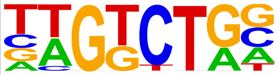	Smad3(MAD)/NPC-Smad3-ChIP-Seq(GSE36673)/Homer	1e-6	-1.438e+01	0.0000	774.0	53.23%	22428.9	46.82%	motif file (matrix)	svg
83	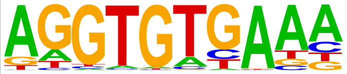	Tbx21(T-box)/GM12878-TBX21-ChIP-Seq(Encode)/Homer	1e-6	-1.409e+01	0.0000	259.0	17.81%	6379.9	13.32%	motif file (matrix)	svg
84	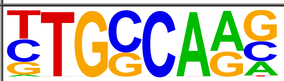	NF1-halfsite(CTF)/LNCaP-NF1-ChIP-Seq(Unpublished)/Homer	1e-6	-1.395e+01	0.0000	509.0	35.01%	13977.0	29.18%	motif file (matrix)	svg
85	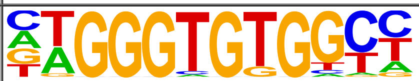	EKLF(Zf)/Erythrocyte-Klf1-ChIP-Seq(GSE20478)/Homer	1e-5	-1.359e+01	0.0000	97.0	6.67%	1919.0	4.01%	motif file (matrix)	svg
86	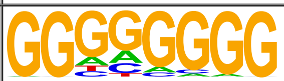	Maz(Zf)/HepG2-Maz-ChIP-Seq(GSE31477)/Homer	1e-5	-1.339e+01	0.0000	635.0	43.67%	18038.3	37.66%	motif file (matrix)	svg
87	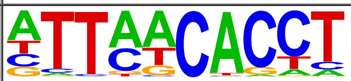	Eomes(T-box)/H9-Eomes-ChIP-Seq(GSE26097)/Homer	1e-5	-1.338e+01	0.0000	506.0	34.80%	13947.4	29.12%	motif file (matrix)	svg
88	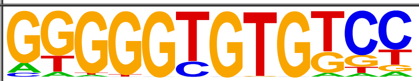	KLF10(Zf)/HEK293-KLF10.GFP-ChIP-Seq(GSE58341)/Homer	1e-5	-1.335e+01	0.0000	309.0	21.25%	7918.3	16.53%	motif file (matrix)	svg
89	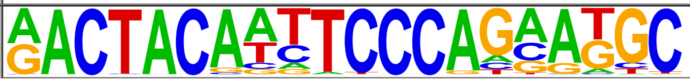	GFY-Staf(?,Zf)/Promoter/Homer	1e-5	-1.326e+01	0.0000	42.0	2.89%	612.3	1.28%	motif file	svg

										(matrix)	
90	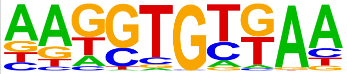	Tbr1(T-box)/Cortex-Tbr1-ChIP-Seq(GSE71384)/Homer	1e-5	-1.198e+01	0.0000	340.0	23.38%	8984.4	18.76%	motif file (matrix)	svg
91	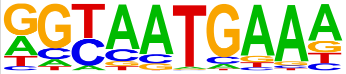	Hoxa10(Homeobox)/ChickenMSG-Hoxa10.Flag-ChIP-Seq(GSE86088)/Homer	1e-5	-1.163e+01	0.0000	150.0	10.32%	3453.6	7.21%	motif file (matrix)	svg
92	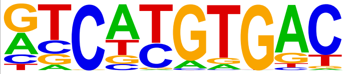	MITF(bHLH)/MastCells-MITF-ChIP-Seq(GSE48085)/Homer	1e-4	-1.118e+01	0.0001	277.0	19.05%	7172.0	14.97%	motif file (matrix)	svg
93	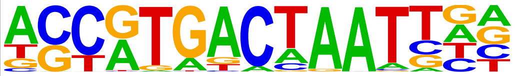	PAX3:FKHR-fusion(Paired,Homeobox)/Rh4-PAX3:FKHR-ChIP-Seq(GSE19063)/Homer	1e-4	-1.097e+01	0.0001	73.0	5.02%	1426.2	2.98%	motif file (matrix)	svg
94	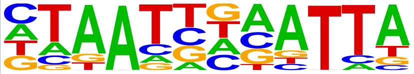	Prop1(Homeobox)/GHFT1-PROP1.biotin-ChIP-Seq(GSE77302)/Homer	1e-4	-1.090e+01	0.0001	152.0	10.45%	3560.2	7.43%	motif file (matrix)	svg
95	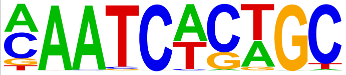	Gfi1b(Zf)/HPC7-Gfi1b-ChIP-Seq(GSE22178)/Homer	1e-4	-1.078e+01	0.0001	186.0	12.79%	4537.3	9.47%	motif file (matrix)	svg
96	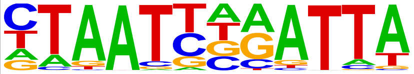	Phox2a(Homeobox)/Neuron-Phox2a-ChIP-Seq(GSE31456)/Homer	1e-4	-1.009e+01	0.0002	101.0	6.95%	2206.6	4.61%	motif file (matrix)	svg
97	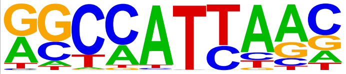	Nanog(Homeobox)/mES-Nanog-ChIP-Seq(GSE11724)/Homer	1e-4	-1.007e+01	0.0002	911.0	62.65%	27570.4	57.55%	motif file (matrix)	svg
98	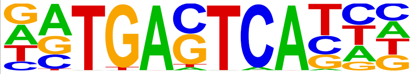	Atf3(bZIP)/GBM-ATF3-ChIP-Seq(GSE33912)/Homer	1e-4	-9.384e+00	0.0004	159.0	10.94%	3875.1	8.09%	motif file (matrix)	svg
99	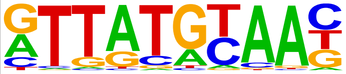	HLF(bZIP)/HSC-HLF.Flag-ChIP-Seq(GSE69817)/Homer	1e-4	-9.212e+00	0.0004	195.0	13.41%	4934.7	10.30%	motif file (matrix)	svg
100	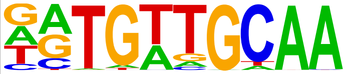	CEBP:AP1(bZIP)/ThioMac-CEBPb-ChIP-Seq(GSE21512)/Homer	1e-3	-9.123e+00	0.0005	179.0	12.31%	4475.8	9.34%	motif file (matrix)	svg
101	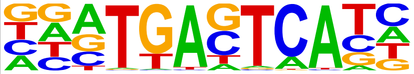	Fra1(bZIP)/BT549-Fra1-ChIP-Seq(GSE46166)/Homer	1e-3	-9.097e+00	0.0005	135.0	9.28%	3215.0	6.71%	motif file (matrix)	svg
102	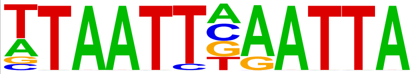	Phox2b(Homeobox)/CLBGA-PHOX2B-ChIP-Seq(GSE90683)/Homer	1e-3	-8.764e+00	0.0007	58.0	3.99%	1144.5	2.39%	motif file (matrix)	svg
103	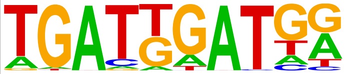	HOXA1(Homeobox)/mES-Hoxa1-ChIP-Seq(SRP084292)/Homer	1e-3	-8.608e+00	0.0008	74.0	5.09%	1568.6	3.27%	motif file (matrix)	svg
104	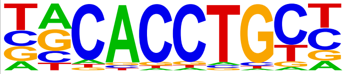	Snail1(Zf)/LS174T-SNAIL1.HA-ChIP-Seq(GSE127183)/Homer	1e-3	-8.573e+00	0.0008	311.0	21.39%	8485.4	17.71%	motif file (matrix)	svg
105	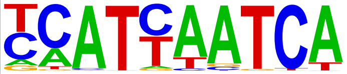	Pdx1(Homeobox)/Islet-Pdx1-ChIP-Seq(SRA008281)/Homer	1e-3	-8.445e+00	0.0009	222.0	15.27%	5808.9	12.13%	motif file (matrix)	svg
106	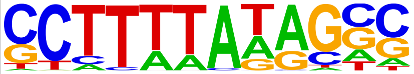	TATA-Box(TBP)/Promoter/Homer	1e-3	-8.307e+00	0.0010	335.0	23.04%	9256.1	19.32%	motif file (matrix)	svg
107		Smad2(MAD)/ES-SMAD2-ChIP-Seq(GSE29422)/Homer	1e-3	-8.275e+00	0.0010	476.0	32.74%	13669.8	28.54%	motif file	svg

	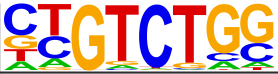									(matrix)	
108	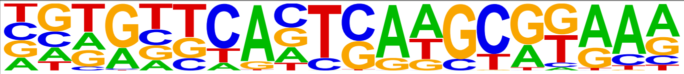	PAX6(Paired,Homeobox)/Forebrain-Pax6-ChIP-Seq(GSE66961)/Homer	1e-3	-7.372e+00	0.0025	36.0	2.48%	653.6	1.36%	motif file (matrix)	svg
109	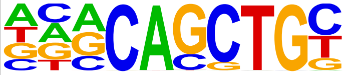	E2A(bHLH)/proBcell-E2A-ChIP-Seq(GSE21978)/Homer	1e-3	-7.055e+00	0.0035	471.0	32.39%	13702.2	28.60%	motif file (matrix)	svg
110	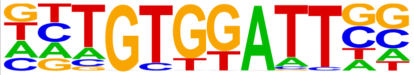	Foxh1(Forkhead)/hESC-FOXH1-ChIP-Seq(GSE29422)/Homer	1e-3	-6.977e+00	0.0037	149.0	10.25%	3798.1	7.93%	motif file (matrix)	svg
111	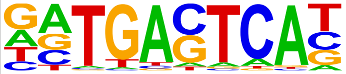	JunB(bZIP)/DendriticCells-Junb-ChIP-Seq(GSE36099)/Homer	1e-3	-6.919e+00	0.0039	130.0	8.94%	3250.3	6.79%	motif file (matrix)	svg
112	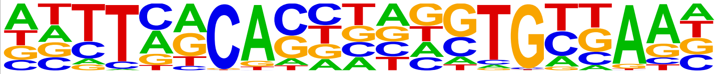	Brachyury(T-box)/Mesoendoderm-Brachyury-ChIP-exo(GSE54963)/Homer	1e-2	-6.811e+00	0.0043	79.0	5.43%	1812.2	3.78%	motif file (matrix)	svg
113	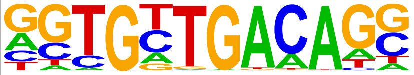	Tbx20(T-box)/Heart-Tbx20-ChIP-Seq(GSE29636)/Homer	1e-2	-6.791e+00	0.0044	72.0	4.95%	1621.1	3.38%	motif file (matrix)	svg
114	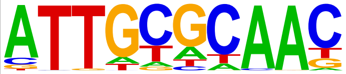	CEBP(bZIP)/ThioMac-CEBPb-ChIP-Seq(GSE21512)/Homer	1e-2	-6.722e+00	0.0046	152.0	10.45%	3911.3	8.17%	motif file (matrix)	svg
115	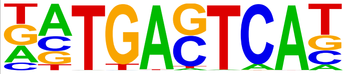	BATF(bZIP)/Th17-BATF-ChIP-Seq(GSE39756)/Homer	1e-2	-6.470e+00	0.0059	153.0	10.52%	3966.4	8.28%	motif file (matrix)	svg
116	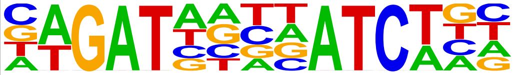	GATA(Zf),IR4/iTreg-Gata3-ChIP-Seq(GSE20898)/Homer	1e-2	-6.444e+00	0.0060	26.0	1.79%	446.8	0.93%	motif file (matrix)	svg
117	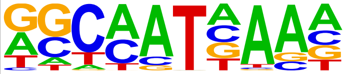	Hoxa9(Homeobox)/ChickenMSG-Hoxa9.Flag-ChIP-Seq(GSE86088)/Homer	1e-2	-6.320e+00	0.0067	599.0	41.20%	17938.4	37.45%	motif file (matrix)	svg
118	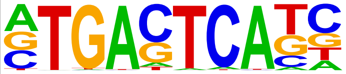	AP-1(bZIP)/ThioMac-PU.1-ChIP-Seq(GSE21512)/Homer	1e-2	-6.273e+00	0.0070	168.0	11.55%	4432.4	9.25%	motif file (matrix)	svg
119	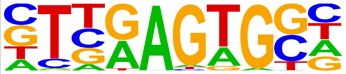	Nkx2.2(Homeobox)/NPC-Nkx2.2-ChIP-Seq(GSE61673)/Homer	1e-2	-6.203e+00	0.0074	525.0	36.11%	15575.1	32.51%	motif file (matrix)	svg
120	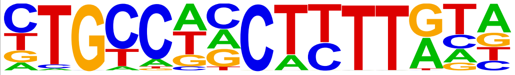	ZNF7(Zf)/HepG2-ZNF7.Flag-ChIP-Seq(Encode)/Homer	1e-2	-6.148e+00	0.0078	141.0	9.70%	3645.1	7.61%	motif file (matrix)	svg
121	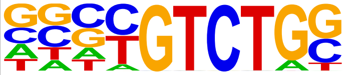	Smad4(MAD)/ESC-SMAD4-ChIP-Seq(GSE29422)/Homer	1e-2	-6.123e+00	0.0079	481.0	33.08%	14181.2	29.60%	motif file (matrix)	svg
122	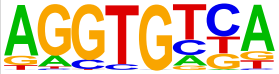	Tbx5(T-box)/HL1-Tbx5.biotin-ChIP-Seq(GSE21529)/Homer	1e-2	-5.998e+00	0.0089	772.0	53.09%	23653.3	49.38%	motif file (matrix)	svg
123	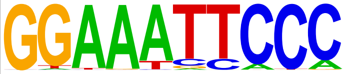	NFkB-p65-Rel(RHD)/ThioMac-LPS-Expression(GSE23622)/Homer	1e-2	-5.917e+00	0.0096	21.0	1.44%	346.9	0.72%	motif file (matrix)	svg
124	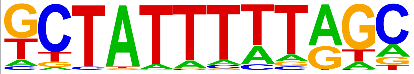	Mef2d(MADS)/Retina-Mef2d-ChIP-Seq(GSE61391)/Homer	1e-2	-5.824e+00	0.0104	47.0	3.23%	1004.0	2.10%	motif file (matrix)	svg
125	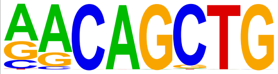	MyoG(bHLH)/C2C12-MyoG-ChIP-Seq(GSE36024)/Homer	1e-2	-5.783e+00	0.0108	332.0	22.83%	9527.9	19.89%	motif file (matrix)	svg

126	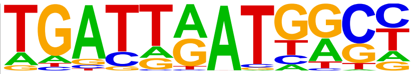	Hoxb4(Homeobox)/ES-Hoxb4-ChIP-Seq(GSE34014)/Homer	1e-2	-5.733e+00	0.0113	54.0	3.71%	1197.4	2.50%	motif file (matrix)	svg
127	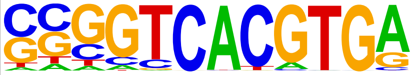	E-box(bHLH)/Promoter/Homer	1e-2	-5.509e+00	0.0140	43.0	2.96%	915.3	1.91%	motif file (matrix)	svg
128	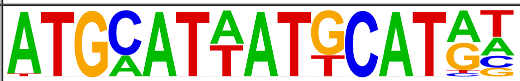	OCT:OCT-short(POU,Homeobox)/NPC-OCT6-ChIP-Seq(GSE43916)/Homer	1e-2	-5.494e+00	0.0141	152.0	10.45%	4042.9	8.44%	motif file (matrix)	svg
129	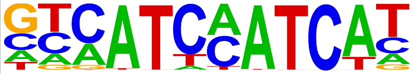	HOXA2(Homeobox)/mES-Hoxa2-ChIP-Seq(Donaldson_et_al.)/Homer	1e-2	-5.333e+00	0.0164	32.0	2.20%	637.7	1.33%	motif file (matrix)	svg
130	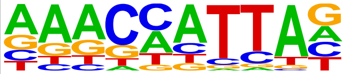	Barx1(Homeobox)/Stomach-Barx1.3xFlag-ChIP-Seq(GSE69483)/Homer	1e-2	-5.318e+00	0.0165	117.0	8.05%	3023.8	6.31%	motif file (matrix)	svg
131	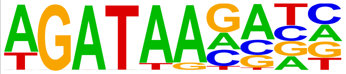	TRPS1(Zf)/MCF7-TRPS1-ChIP-Seq(GSE107013)/Homer	1e-2	-5.252e+00	0.0175	419.0	28.82%	12366.3	25.82%	motif file (matrix)	svg
132	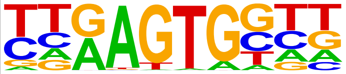	Bapx1(Homeobox)/VertebralCol-Bapx1-ChIP-Seq(GSE36672)/Homer	1e-2	-5.219e+00	0.0180	544.0	37.41%	16378.7	34.19%	motif file (matrix)	svg
133	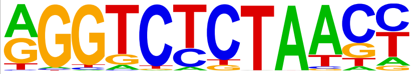	PRDM14(Zf)/H1-PRDM14-ChIP-Seq(GSE22767)/Homer	1e-2	-5.146e+00	0.0192	96.0	6.60%	2426.8	5.07%	motif file (matrix)	svg
134	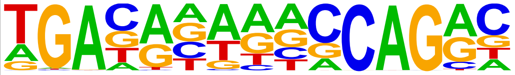	Hand2(bHLH)/Mesoderm-Hand2-ChIP-Seq(GSE61475)/Homer	1e-2	-5.094e+00	0.0200	135.0	9.28%	3580.2	7.47%	motif file (matrix)	svg
135	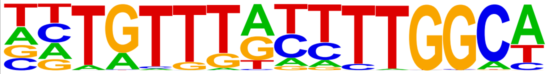	NF1:FOXA1(CTF,Forkhead)/LNCAP-FOXA1-ChIP-Seq(GSE27824)/Homer	1e-2	-5.044e+00	0.0209	17.0	1.17%	280.9	0.59%	motif file (matrix)	svg
136	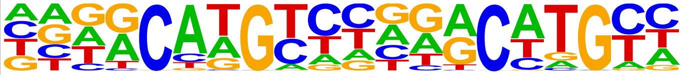	p63(p53)/Keratinocyte-p63-ChIP-Seq(GSE17611)/Homer	1e-2	-5.038e+00	0.0209	111.0	7.63%	2875.5	6.00%	motif file (matrix)	svg
137	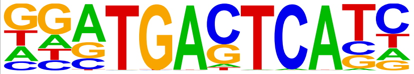	Fra2(bZIP)/Striatum-Fra2-ChIP-Seq(GSE43429)/Homer	1e-2	-5.023e+00	0.0210	107.0	7.36%	2759.2	5.76%	motif file (matrix)	svg
138	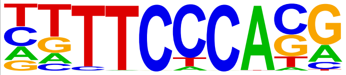	Rbpj1(?)/Panc1-Rbpj1-ChIP-Seq(GSE47459)/Homer	1e-2	-5.008e+00	0.0212	368.0	25.31%	10794.2	22.53%	motif file (matrix)	svg
139	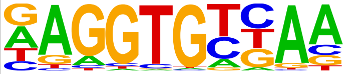	Tbx6(T-box)/ESC-Tbx6-ChIP-Seq(GSE93524)/Homer	1e-2	-4.919e+00	0.0230	268.0	18.43%	7670.8	16.01%	motif file (matrix)	svg
140	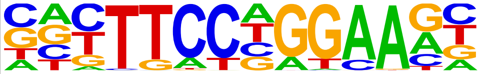	Stat3+il21(Stat)/CD4-Stat3-ChIP-Seq(GSE19198)/Homer	1e-2	-4.889e+00	0.0235	193.0	13.27%	5359.2	11.19%	motif file (matrix)	svg
141	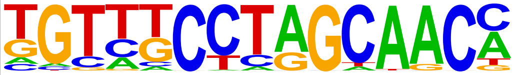	Rfx6(HTH)/Min6b1-Rfx6.HA-ChIP-Seq(GSE62844)/Homer	1e-2	-4.840e+00	0.0246	341.0	23.45%	9972.5	20.82%	motif file (matrix)	svg
142	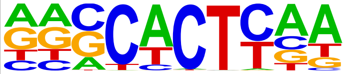	Nkx2.5(Homeobox)/HL1-Nkx2.5.biotin-ChIP-Seq(GSE21529)/Homer	1e-2	-4.803e+00	0.0253	556.0	38.24%	16854.9	35.19%	motif file (matrix)	svg
143	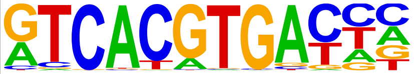	TFE3(bHLH)/MEF-TFE3-ChIP-Seq(GSE75757)/Homer	1e-2	-4.763e+00	0.0262	35.0	2.41%	743.1	1.55%	motif file (matrix)	svg
144	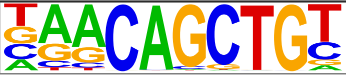	Myf5(bHLH)/GM-Myf5-ChIP-Seq(GSE24852)/Homer	1e-2	-4.753e+00	0.0262	216.0	14.86%	6085.2	12.70%	motif file	svg

											(matrix)	
