# Supplementary material for: AP-2α and AP-2β cooperatively function in the craniofacial surface ectoderm to regulate chromatin and gene expression dynamics during facial development
Source: eLife. 2022 Mar 25;11:e70511. doi: 10.7554/eLife.70511 (PMC9038197; doi:10.7554/eLife.70511)
Supplement: Transparent reporting form [file elife-70511-transrepform1.docx]

***eLife’s* transparent reporting form**

We encourage authors to provide detailed information *within their submission* to facilitate the interpretation and replication of experiments. Authors can upload supporting documentation to indicate the use of appropriate reporting guidelines for health-related research (see [EQUATOR Network](http://www.equator-network.org/%20)), life science research (see the [BioSharing Information Resource](https://biosharing.org/)), or the [ARRIVE guidelines](http://www.plosbiology.org/article/info:doi/10.1371/journal.pbio.1000412) for reporting work involving animal research. Where applicable, authors should refer to any relevant reporting standards documents in this form.

If you have any questions, please consult our Journal Policies and/or contact us: [editorial@elifesciences.org](mailto:editorial@elifesciences.org).

**Sample-size estimation**

- You should state whether an appropriate sample size was computed when the study was being designed
- You should state the statistical method of sample size computation and any required assumptions
- If no explicit power analysis was used, you should describe how you decided what sample (replicate) size (number) to use

Please outline where this information can be found within the submission (e.g., sections or figure legends), or explain why this information doesn’t apply to your submission:

► Sample size for embryo analysis involved a minimum of 3 for each assay performed.

o	SEM analysis

o	In situ hybridization

o	ß-galactosidase staining

o	Gross morphology

o	Bone and cartilage/Cartilage staining

► Sample size for next-generation sequencing datasets was based on standard practice within the field (e.g., biological triplicate for RNA-seq, biological duplicate for ATAC-seq).

► No explicit power analysis was performed as consistent phenotypic differences were seen between mutant and control phenotypes identified by genotype analysis across all experiments.

**Replicates**

- You should report how often each experiment was performed
- You should include a definition of biological versus technical replication
- The data obtained should be provided and sufficient information should be provided to indicate the number of independent biological and/or technical replicates
- If you encountered any outliers, you should describe how these were handled
- Criteria for exclusion/inclusion of data should be clearly stated
- High-throughput sequence data should be uploaded before submission, with a private link for reviewers provided (these are available from both GEO and ArrayExpress)

Please outline where this information can be found within the submission (e.g., sections or figure legends), or explain why this information doesn’t apply to your submission:

► RNA-seq (Figure 1, Figure 7) – 3 biological replicates (information found in figure legend)

► ATAC-seq (Figure 2 and 3) was conducted in duplicate in wild-type or mutant embryos (information found in ‘Results’ and ‘Methods’ section).

► Embryos (information is included within the ‘Methods’ and/or ‘Results’ section, including figure legend for some)

► A minimum of three embryos per genotype were used for: gross morphology including SEM (Figures 1 and 5); each probe for in situ hybridization (Figure 4, Figure 7—figure supplement 2); skeletal analysis (Figure 6); Axin2-lacZ ß-gal staining (Figure 8); and proliferation analysis (Figure 4—figure supplement 5).

► Wnt1 gain-of-function rescue analysis (Figure 8), a minimum of 7 embryos/genotype

► Real-time PCR (Figure 4) – 2 biological replicates per genotype, with technical triplicates for real-time analysis (information is included within the Figure legend).

► Real-time PCR (Figure 4—figure supplement 4; Figure 7—figure supplement 2 and 3) – 1 biological replicate with technical triplicates (information is included within the Figure legend).

**Statistical reporting**

- Statistical analysis methods should be described and justified
- Raw data should be presented in figures whenever informative to do so (typically when N per group is less than 10)
- For each experiment, you should identify the statistical tests used, exact values of N, definitions of center, methods of multiple test correction, and dispersion and precision measures (e.g., mean, median, SD, SEM, confidence intervals; and, for the major substantive results, a measure of effect size (e.g., Pearson's r, Cohen's d)
- Report exact p-values wherever possible alongside the summary statistics and 95% confidence intervals. These should be reported for all key questions and not only when the p-value is less than 0.05.

Please outline where this information can be found within the submission (e.g., sections or figure legends), or explain why this information doesn’t apply to your submission:

► Cumulative distribution plot (Figure 2, Figure 2—figure supplement 11), Kolmogorov-Smirnov test (information in ‘Methods’ section).

► Boxplots (Figure 7), standard two-tailed t-test (information in Figure legend).

► Proliferation analysis (Figure 4—figure supplement 5), standard two-tailed t-test (information in ‘Methods’ section)

(For large datasets, or papers with a very large number of statistical tests, you may upload a single table file with tests, Ns, etc., with reference to sections in the manuscript.)

**Group allocation**

- Indicate how samples were allocated into experimental groups (in the case of clinical studies, please specify allocation to treatment method); if randomization was used, please also state if restricted randomization was applied
- Indicate if masking was used during group allocation, data collection and/or data analysis

Please outline where this information can be found within the submission (e.g., sections or figure legends), or explain why this information doesn’t apply to your submission:

► Group allocation was based on the genotypes of the embryos analysed.

► Proliferation analysis (Figure 4—figure supplement 5) was scored by an observer blind to sample groups.

**Additional data files (“source data”)**

- We encourage you to upload relevant additional data files, such as numerical data that are represented as a graph in a figure, or as a summary table
- Where provided, these should be in the most useful format, and they can be uploaded as “Source data” files linked to a main figure or table
- Include model definition files including the full list of parameters used
- Include code used for data analysis (e.g., R, MatLab)
- Avoid stating that data files are “available upon request”

Please indicate the figures or tables for which source data files have been provided:

► Complete output of motif enrichment analysis for the associated dataset (Figure 2—figure supplement 1, 2, 5, 7, 9 and Figure 3—figure supplement 1, 2, 6)

► Complete output of pathway enrichment analysis for the associated dataset (Figure 2—figure supplement 3, 4, 6, 8, 10 and Figure 3—figure supplement 3, 4, 5)

► Entire list of gene expression and ATAC-seq peaks presented in Figure 2 (Supplementary File 1)

► Entire list of AP-2 dependent ATAC-seq peaks presented in Figure 3 (Supplementary File 2)

► Entire gene expression output for Figure 7 datasets (Supplementary File 3)
